# Supplementary material for: Healthcare usage and expenditure among people with type 2 diabetes and/or hypertension in Cambodia: results from a cross-sectional survey
Source: BMJ Open. 2023 Jan 12;13(1):e061959. doi: 10.1136/bmjopen-2022-061959 (PMC9843177; doi:10.1136/bmjopen-2022-061959)

Annexure 1. Locations of the five studied ODs in Cambodia, 2020.

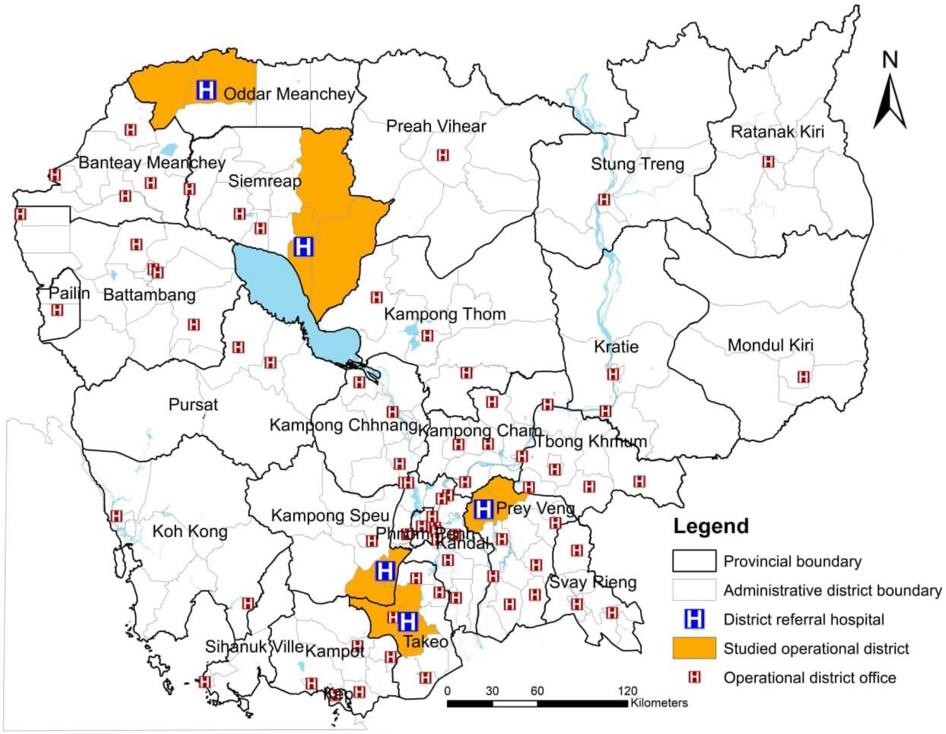

Supplement: Supplementary data [file bmjopen-2022-061959supp001.pdf]
