# Supplementary material for: Healthcare usage and expenditure among people with type 2 diabetes and/or hypertension in Cambodia: results from a cross-sectional survey
Source: BMJ Open. 2023 Jan 12;13(1):e061959. doi: 10.1136/bmjopen-2022-061959 (PMC9843177; doi:10.1136/bmjopen-2022-061959)
Supplement: Supplementary data [file bmjopen-2022-061959supp003.pdf]

Annexure 3. Individual Survey Questionnaire

INTRO:
Hello, my name is ____and I am from the National Institute of Public Health. You has been randomly selected to participate in this study [on the scaling up of diabetes and hypertension in Cambodia] based on information from your household. The information you give will be kept confidential and no personal details will appear in any record. This interview will take approximately 60 minutes. You do not have to answer any question you don't want to and you can stop the interview at any time. There will also be measurements of your blood pressure, weight and height, waist and hip circumferences during and after the interview, and for tomorrow early morning we would like to test your fasting blood glucose [HbA1c and creatinine level for known and suspected diabetes cases]. We very appreciate your participation and information.

**ELIGIBILITY & RECRUITMENT CHECK**<br>(Instructions: To be filled in by data collectors before participants sign informed consent)				
**Q.N**	**Description & questions**	**RESPONSE**		**Ref:**
Q1	Has the individual been a usual member of the household and stayed in the household the night before the interview or had not been absent for more than 6 months?	0 = No	1 = Yes	CDHS
Q2	Is the individual 40 or more than 40 of age?<br><br>*IF POSSIBLE, CHECK ID CARD*	0 = No	1 = Yes	WHO PEN SOP
	If NO, DO NOT continue.			
Q3	Is the individual physically and mentally capable to answer the questions?	0 = No	1 = Yes	
	If NO, DO NOT continue			
Q4	Are you willing to participate in the study?	0 = No	1 = Yes	
	If NO, DO NOT continue<br>If YES, CONSENT obtained			
**Q5. Consent obtained**		0 = No	1 = Yes	

**SECTION 1: SOCIO-DEMOGRAPHIC INFORMATION**					
**Q.N**	**Description & questions**	**RESPONSE**	**Type of Variable**	**Field**	**Ref:**
Q6	How old are you?<br>*-Record in years as stated by the participant*<br>*-Record 99 if don't know*	____________Years	Quantitative discrete	Age	WHO STEPS
Q7	Sex of participant<br>*Record sex of the participant as observed*	1 = Male<br>2 = Female	Categorical binary	Sex	WHO STEPS
Q8	What is your marital status?	1=Married or living together<br>2=Divorced or separated<br>3=Widowed	Categorical nominal	Marital Status	CDHS

Chhim S, *et al. BMJ Open* 2023; 13:e061959. doi: 10.1136/bmjopen-2022-061959

	-Record 88 if refuse to answer	4=Never married and never lived together			
Q9	What is your highest educational level?<br><br>*Record 99 if don't know*	1=No formal schooling<br>2=Less than primary school<br>3=Primary school<br>4=Secondary school<br>5=High school<br>6=College/University<br>7=Post graduate degree	Categorical ordinal	Educatio nal level	WHO STEPS
Q10	What is your ethnic group?	1 = Khmer<br>2 = Vietnamese<br>3 = Chinese<br>4 = Cham (Muslim)<br>5 = Other	Categorical nominal	Ethnicity	WHO STEPS
Q11	Which of the following best describes your main work status within the past 12 months?<br><br>*Record 88 if refuse to answer*	1=Household tasks<br>2=Civil servant<br>3=Employee of private company/NGO<br>4=Self-employed farmer<br>5=Large-scale farmer with employees<br>6=Self-employed in small business<br>7=Running a big business with employees<br>8=Casual worker<br>9=Working abroad<br>10=At school (pupil/student)<br>11=Unemployed or not eligible<br>12=Retired	Categorical nominal	Occupati on	CDHS
Q12	Taking the past year, can you give an estimate of your annual income if I read some options to you?<br><br>*Record 88 if refuse to answer*	1 = no earnings<br>2 = less than or 250 USD<br>3 = more than 251- 1500 USD<br>4 = more than 1501 – 3500 USD<br>5 = more than 3501 USD	Categorical ordinal	Individua l income	WHO STEPS

SECTION 2: HEALTH STATUS AND QUALITY OF LIFE

**Q.N**	**Description and Questions**	**Response**	**Type of Variable**	**Field**	**Ref:**

Chhim S, *et al. BMJ Open* 2023; 13:e061959. doi: 10.1136/bmjopen-2022-061959

Q13	At this point of time in your life, how would you describe:<br><br>Q 13.1. Your home situation [____]<br>Q 13.2. Your family relationships [____]<br>Q 13.3. Your finances [____]<br>Q 13.4. Your work situation [____]<br><br>*-Record 99 if don't know and 88 if refuse*	1 = Excellent<br>2 = Very good<br>3 = Good<br>4 = Fair<br>5 = Poor	Categorical Ordinal	QoLife	GACD book
Q14	How good or bad is your health today?<br><br>*-The scale is numbered from 0 to 100.*<br>*-100 means the best health you can imagine.*<br>*-0 means the worst health you can imagine.*<br>*-Please mark an X on the scale to indicate how your health is today.*<br><br>*-Record 99 if don't know and 88 if refuse*	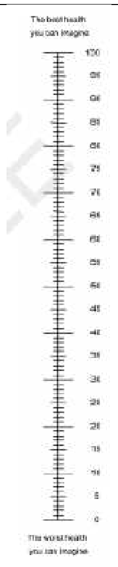	Quantitative discrete	Gen. Health	EuroQoL
Q15	Have you ever been told by a doctor or other health worker that you have hypertension?<br><br>*-Record 99 if don't know/unsure*	0 = No<br>1 = Yes	Categorical binary	HT_self-report diagnosis	WHO STEPS
Q16	Have you ever been told by a doctor or other health worker that you have diabetes?<br><br>*-Record 99 if don't know/unsure*	0 = No<br>1 = Yes	Categorical binary	DM_self-report diagnosis	WHO STEPS

Chhim S, *et al. BMJ Open* 2023; 13:e061959. doi: 10.1136/bmjopen-2022-061959

Q17	Have you ever been told by a doctor or other health worker that you have heart problems?<br><br>*-Record 99 if don't know/unsure*	0 = No<br>1 = Yes	Categorical binary	Co-morbidity (CVD)	WHO STEPS
Q18	Have you ever been told by a doctor or other health worker that you have symptoms suggestive of a stroke?<br><br>*-Record 99 if don't know/unsure*	0 = No<br>1 = Yes	Categorical binary	Co-morbidity (CVD)	WHO STEPS
Q19	Have you ever been told by a doctor or other health worker that you have chronic kidney disease?<br><br>*-Record 99 if don't know/unsure*	0 = No<br>1 = Yes	Categorical binary	Co-morbidity (CKD)	WHO STEPS
Q20	How many natural teeth do you have?<br><br>*-Record 99 if don't know*	0 = None<br>1 = 1-9 teeth<br>2 = 10-19 teeth<br>3 = 20 teeth or more	Categorical ordinal	Dental health	WHO Oral Health Questionnaire
Q21	During the past 12 months, did your teeth or mouth cause any pain or comfort?<br><br>*-Record 99 if don't know*	0 = No<br>1 = Yes	Categorical binary	Dental health	WHO Oral Health Questionnaire
Q22	Over the last 2 weeks, how often have you been bothered by any of the following problems?<br><br>Q 22.1. Little interest or pleasure in doing things [____]<br>Q 22.2. Feeling down, depressed, or hopeless. [____]<br>Q 22.3. Trouble falling or staying asleep, or sleeping too much. [____]	0 = Not at all<br>1 = Several days<br>2 = More than half the days<br>3 = Nearly everyday	Categorical ordinal	Mental health	PHQ-9

Chhim S, *et al. BMJ Open* 2023; 13:e061959. doi: 10.1136/bmjopen-2022-061959

	Q 22.4. Feeling tired or having little energy. [____]<br>Q 22.5. Poor appetite or overeating [____]<br>Q 22.6. Feeling bad about yourself – or that you are a failure or make yourself or down your family [____]<br>Q 22.7. Trouble concentrating on things, such as reading the newspaper or watching television [____]<br>Q 22.8. Moving or speaking so slowly that other people could have noticed? Or the oppositebeing so fidgety or restless that you have been moving around a lot more than usual [____]<br>Q 22.9. Thoughts that you would be better off dead or of hurting yourself in some ways [____]				
Q42	We would like to confirm that:<br><br>*-This question is for categorizing respondents for the following sections and it is also important to ask respondents to confirm their main conditions in this survey.*<br><br>*-IF the answer is "0", go to Section 3*<br>*-IF the answer is "1", go to Section 3a*<br>*-IF the answer is "2", go to Section 3b*<br>*-IF the answer is "3", go to Section 3c*	0 = Neither hypertension nor diabetes<br><br>1 = Only hypertension<br><br>2 = Only diabetes<br><br>3 = Both diabetes and hypertension			

Chhim S, *et al. BMJ Open* 2023; 13:e061959. doi: 10.1136/bmjopen-2022-061959

SECTION 3: HEALTH CARE UTILIZATION					
**Q.N**	**Description and Questions**	**Response**	**Type of Variable**	**Field**	**Ref:**
Q23	Have you sought medical treatment or advice as an outpatient from anyone in the past 3 months?<br><br>*-If No, go to Question 40.*	0 = No<br>1 = Yes	Categorical variable	Medic al advice	GACD book
Q24	Where did you seek medical advice or treatment for illness in the past 3 months?<br><br>*-More than one answer can be selected.*<br>*-Data collectors can use probes to help respondents determine the types of health facilities in the Response Column.*<br>*-Record 99 if don't know and 88 if refuse*	1= National hospital (PP)<br>2= Provincial hospital (RH)<br>3= District hospital (RH)<br>4= Health centre<br>5= Health post<br>6= Provincial rehabilitation centre (PRC) or Community-based rehabilitation (CBR)<br>7= Other public; specify:<br>8= Private hospital<br>9= Private clinic<br>10= Private pharmacy<br>11= Home/Office of trained health worker/nurse<br>12= Visit of trained health worker/nurse<br>13= Other private medical; specify:<br>14= Shop selling drugs/market<br>15= Kru Khmer/ Magician	Categorical Nominal	Care provid er_3M	CDHS and GACD book

		16= Monk/religious leader<br>17= Traditional birth attendant<br>18= Oversee medical service<br>19= Other; specify:			
From Q25-Q39, it is a set of questions that are asked following choices selected in Q24. If 2 or 3 choices were selected in Q24, Q25-Q39 would appear 2 or 3 times, accordingly.					
Q25	How many times did you visit the selected place**(s)** in Q24 in the past three months?	_______ times	Quantitative discrete	Number of visits	GACD
Q26	How much in total was spent on the treatment at the selected place(s) in Q24?<br><br>*-Record 99 if don't know and 88 if refuse*	0 = free/no cost<br>1 = in kind<br>2 = ________Riels	-Categorical nominal<br>-<br>Quantitative Continuous	Cost of treatment	CDHS
Q27	How did you pay for the treatment cost at the selected place(s) in Q24?<br><br>*-Record 99 if don't know and 88 if refuse*	1= Health Equity Fund<br>2= Voucher<br>3= Fee Exemption<br>4= NGO<br>5= National Social Security Fund<br>6= Community-Based Health Insurance<br>7= Health Insurance through Employer<br>8= Other Privately Purchased Commercial Health Insurance	-Categorical nominal	Payment method	GADC adapted to context specific

		9= Wage/income<br>10= Loan/ Ton Tin<br>11= Sale of Assets<br>12= Gift from Relative<br>13= Savings<br>14= Other			
Q28	How much in total was spent on transport to go to and return from the selected place(s) in Q24?<br><br>*-Record 99 if don't know and 88 if refuse*	0 = free/no cost<br>1 = in kind<br>2 = ________Riels	-Categorical nominal<br>-Quantitative Continuous	Cost of transport	CDHS
Q29	On average how many hours do you spend to get treatment/advices from the selected place(s) in Q24?<br><br>*-Record 99 if don't know and 88 if refuse*	________Hours	Quantitative continuous	Time spending	GACD book (Adapted)
Q30	How satisfied are you with the effect of your {treatment/care} at the selected place(s) in Q24?<br><br>*-Record 99 if don't know/unsure*	0 = Very satisfied<br>1 = Satisfied<br>2 = Neither satisfied nor dissatisfied<br>3 = Dissatisfied<br>4 = Very dissatisfied	Categorical ordinal	Patient satisfaction of care services	SAPS 2006
Q31	How satisfied are you with the explanations the {doctor/other health professional} has given you about the results of your {treatment/care} at the selected place(s) in Q24?<br><br>*-Record 99 if don't know/unsure*	0 = Very satisfied<br>1 = Satisfied<br>2 = Neither satisfied nor dissatisfied<br>3 = Dissatisfied<br>4 = Very dissatisfied	Categorical ordinal	Patient satisfaction of care services	SAPS 2006

Chhim S, *et al. BMJ Open* 2023; 13:e061959. doi: 10.1136/bmjopen-2022-061959

Q32	The {doctor/other health professional} at the selected place(s) in Q24 was very careful to check everything when examining you.<br><br>*-Record 99 if don't know/unsure*	0 = Strongly agree<br>1 = Agree<br>2 = Not sure<br>3 = Disagree<br>4 = Strongly disagree	Categorical ordinal	Patient satisfaction of care services	SAPS 2006
Q33	At the selected place(s) in Q24, how satisfied were you with the choices you had in decisions affecting your health care?<br><br>*-Record 99 if don't know/unsure*	0 = Very satisfied<br>1 = Satisfied<br>2 = Neither satisfied nor dissatisfied<br>3 = Dissatisfied<br>4 = Very dissatisfied	Categorical ordinal	Patient satisfaction of care services	SAPS 2006
Q34	How much of the time did you feel respected by the {doctor/other health professional} at the selected place(s) in Q24?<br><br>*-Record 99 if don't know/unsure*	0 = All of the time<br>1 = Most of the time<br>2 = About half the time<br>3 = Some of the time<br>4 = None of the time	Categorical ordinal	Patient satisfaction of care services	SAPS 2006
Q35	At the selected place(s) in Q24, the time you had with the {doctor/other health professional} was too short.<br><br>*-Record 99 if don't know/unsure*	0 = Strongly agree<br>1 = Agree<br>2 = Not sure<br>3 = Disagree<br>4 = Strongly disagree	Categorical ordinal	Patient satisfaction of care services	SAPS 2006
Q36	Are you satisfied with the care you received in the selected place(s) in Q24?<br><br>*-Record 99 if don't know/unsure*	0 = Very satisfied<br>1 = Satisfied<br>2 = Neither satisfied nor dissatisfied<br>3 = Dissatisfied<br>4 = Very dissatisfied	Categorical ordinal	Patient satisfaction of care services	SAPS 2006

Chhim S, *et al. BMJ Open* 2023; 13:e061959. doi: 10.1136/bmjopen-2022-061959

Q37	Did you get your blood pressure measured at the selected place(s) in Q24?<br><br>*-Record 99 if don't know/unsure*	0 = No<br>1 = Yes	Categorical variable	Acces s to blood pressu re testing	Opinion
Q38	Did you get your blood glucose tested at the selected place(s) in Q24?<br><br>*-Record 99 if don't know/unsure*	0 = No<br>1 = Yes	Categorical variable	Acces s to blood glucos e testing	Opinion
Q39	Would you recommend the selected place(s) in Q24 to others?<br><br>*-Record 99 if don't know/unsure*	1 = Not recommended<br>2 = Recommend with reservations<br>3 = Recommend<br>4 = Highly recommend	Categorical ordinal	Patien t satisfa ction of care servic es	Opinion
Q40	Have you ever had your blood glucose tested in the last three years?<br><br>*-Record 99 if don't know/unsure*	0 = No<br>1 = Yes	Categorical binary	Testin g DM (CoC)	Opinion
Q41	Have you ever had your blood pressure measured in the last three years?<br><br>*-Record 99 if don't know/unsure*	0 = No<br>1 = Yes	Categorical binary	Testin g HT (CoC)	Opinion

SECTION 3a: HEALTH CARE UTILIZATION FOR HYPERTENSION

Q.N	Description and Questions	Response	Type of Variable	Field	Ref:
Q43a	How long have you lived with hypertension?<br><br>*-Record 99 if don't know/unsure and 88 if refuse*<br>*-Less than a year is rounded up to one year*<br>*-Standard rounded up formula is applied.*	________Years	Quantitative concrete	Durati on HT	Opinion
Q44a	Where were you first diagnosed as having hypertension?<br><br>*-Record 99 if don't know and 88 if refuse*	1= National hospital (PP)<br>2= Provincial hospital (RH)<br>3= District hospital (RH)<br>4= Health centre<br>5= Health post<br>6= Provincial rehabilitation centre (PRC) or Community-based rehabilitation (CBR)<br>7= Other public; specify:<br>8= Private hospital<br>9= Private clinic<br>10= Private pharmacy<br>11= Home/Office of trained health worker/nurse<br>12= Visit of trained health worker/nurse<br>13= Other private medical; specify:<br>14= Shop selling drugs/market<br>15= Kru Khmer/ Magician	Categorical Nominal	Diagn osis HT (CoC)	Opinion

Chhim S, *et al. BMJ Open* 2023; 13:e061959. doi: 10.1136/bmjopen-2022-061959

		16= Monk/religious leader<br>17= Traditional birth attendant<br>18= Oversee medical service<br>19= MoPoTsyo<br>20= Other; specify:			
Q45a	Where did you first seek advice or treatment for hypertension after being diagnosed?<br><br>*-Record 99 if don't know and 88 if refuse*	1= National hospital (PP)<br>2= Provincial hospital (RH)<br>3= District hospital (RH)<br>4= Health centre<br>5= Health post<br>6= Provincial rehabilitation centre (PRC) or Community-based rehabilitation (CBR)<br>7= Other public; specify:<br>8= Private hospital<br>9= Private clinic<br>10= Private pharmacy<br>11= Home/Office of trained health worker/nurse<br>12= Visit of trained health worker/nurse<br>13= Other private medical; specify:<br>14= Shop selling drugs/market<br>15= Kru Khmer/ Magician<br>16= Monk/religious leader<br>17= Traditional birth attendant	Categorical Nominal	Link to care HT (CoC)	CDHS (Adapted for disease specific)

		18= Oversee medical service<br>19= MoPoTsyo<br>20= Other; specify:			
Q46a	Did you go to other places for follow up treatment/care for your hypertensive conditions?<br><br>*-Record 88 if refuse*<br>*-If NO, please skip Q47a*	0 = No<br>1 = Yes	Categorical binary	Trust HT	CDHS
Q47a	If yes to Q46a, where else did you go to get follow up treatment/care for your hypertensive conditions?	1= National hospital (PP)<br>2= Provincial hospital (RH)<br>3= District hospital (RH)<br>4= Health centre<br>5= Health post<br>6= Provincial rehabilitation centre (PRC) or Community-based rehabilitation (CBR)<br>7= Other public; specify:<br>8= Private hospital<br>9= Private clinic<br>10= Private pharmacy<br>11= Home/Office of trained health worker/nurse<br>12= Visit of trained health worker/nurse<br>13= Other private medical; specify:<br>14= Shop selling drugs/market	Categorical Nominal	Link to care HT (CoC)	CDHS (Adapted for disease specific)

Chhim S, *et al. BMJ Open* 2023; 13:e061959. doi: 10.1136/bmjopen-2022-061959

		15= Kru Khmer/ Magician<br>16= Monk/religious leader<br>17= Traditional birth attendant<br>18= Oversee medical service<br>19= MoPoTsyo<br>20= Other; specify:			
Q48a	Did you get treatment/care for your hypertensive conditions in the past **12 months?**<br><br>*-Record 88 if refuse*<br>*-If NO, please skip Q49a-64a*	0 = No<br>1 = Yes	Categorical binary	Retain in care HT (CoC)	CDHS (Adapted for disease specific)
Q49a	Are you currently receiving any of the following treatment/advices for your hypertensive conditions prescribed by a doctor or other health care worker?<br><br>Q 49.1a. Drugs (medication) that you have taken in the past two weeks [____]<br>Q 49.2a. Advice to reduce salt intake [___]<br>Q 49.3a. Advice or treatment to lose weight [____]<br>Q 49.4a. Advice or treatment to stop smoking [____]<br>Q 49.5a. Advice to start or do more physical exercise [____]<br><br>*-Record 99 if don't know and 88 if refuse*	0 = No 1= Yes	Categorical ordinal	In treatm ent for HT (CoC)	STEPS Survey
Q50a	Have you had your blood cholesterol measured in the past 12 months?<br><br>*-Record 99 if don't know and 88 if refuse*	0 = No 1= Yes	Categorical ordinal	In treatm ent for HT (CoC)	Veerle's suggestion

Chhim S, *et al. BMJ Open* 2023; 13:e061959. doi: 10.1136/bmjopen-2022-061959

Q51a	Where did you seek medical advice or treatment for your hypertensive condition in **the past 3 months**?<br><br>-***More than one answer can be selected***.<br>*-Data collectors can use probes to help respondents determine the types of health facilities in the Response Column.*<br>*-Record 99 if don't know and 88 if refuse*<br>*-If (21=no wehere), go to Q65a*	1= National hospital (PP)<br>2= Provincial hospital (RH)<br>3= District hospital (RH)<br>4= Health centre<br>5= Health post<br>6= Provincial rehabilitation centre (PRC) or Community-based rehabilitation (CBR)<br>7= Other public; specify:<br>8= Private hospital<br>9= Private clinic<br>10= Private pharmacy<br>11= Home/Office of trained health worker/nurse<br>12= Visit of trained health worker/nurse<br>13= Other private medical; specify:<br>14= Shop selling drugs/market<br>15= Kru Khmer/ Magician<br>16= Monk/religious leader<br>17= Traditional birth attendant<br>18= Oversee medical service<br>19= MoPoTsyo<br>20= Other; specify:	Categorical Nominal	Care provid er_3M	CDHS and GACD book

		21= No where			
From Q52a-Q64a, it is a set of questions that are asked following choices selected in Q51a. If 2 or 3 choices were selected in Q51a, Q52a-Q64a would appear 2 or 3 times, accordingly.					
Q52a	How many times did you visit the selected place**(s)** in Q51a in the past three months?	_______ times	Quantitative discrete	Number of visits	GACD
Q53a	How much in total was spent on the treatment at the selected place(s) in Q51a?<br><br>*-Record 99 if don't know and 88 if refuse*	0 = free/no cost<br>1 = in kind<br>2 = ________Riels	-Categorical nominal<br>-<br>Quantitative Continuous	Cost of treatment	CDHS
Q54a	How did you pay for the treatment cost at the selected place(s) in Q51a?<br><br>*-Record 99 if don't know and 88 if refuse*	1= Health Equity Fund<br>2= Voucher<br>3= Fee Exemption<br>4= NGO<br>5= National Social Security Fund<br>6= Community-Based Health Insurance<br>7= Health Insurance through Employer<br>8= Other Privately Purchased Commercial Health Insurance	-Categorical nominal	Payment method	GADC adapted to context specific

		9= Wage/income<br>10= Loan/ Ton Tin<br>11= Sale of Assets<br>12= Gift from Relative<br>13= Savings<br>14= Other			
Q55a	How much in total was spent on transport to go to and return from the selected place(s) in Q51a?<br><br>*-Record 99 if don't know and 88 if refuse*	0 = free/no cost<br>1 = in kind<br>2 = ________Riels	-Categorical nominal<br>-<br>Quantitative Continuous	Cost of transport	CDHS
Q56a	On average how many hours do you spend to get treatment/advices from the selected place(s) in Q51a?<br><br>*-Record 99 if don't know and 88 if refuse*	________Hours	Quantitative continuous	Time spending	GACD book (Adapted)
Q57a	How satisfied are you with the effect of your {treatment/care} at the selected place(s) in Q51a?<br><br>*-Record 99 if don't know/unsure*	0 = Very satisfied<br>1 = Satisfied<br>2 = Neither satisfied nor dissatisfied<br>3 = Dissatisfied<br>4 = Very dissatisfied	Categorical ordinal	Patient satisfaction of care services	SAPS 2006
Q58a	How satisfied are you with the explanations the {doctor/other health professional} has given you about the results of your {treatment/care} at the selected place(s) in Q51a?<br><br>*-Record 99 if don't know/unsure*	0 = Very satisfied<br>1 = Satisfied<br>2 = Neither satisfied nor dissatisfied<br>3 = Dissatisfied<br>4 = Very d issatisfied	Categorical ordinal	Patient satisfaction of care services	SAPS 2006

Chhim S, *et al. BMJ Open* 2023; 13:e061959. doi: 10.1136/bmjopen-2022-061959

Q59a	The {doctor/other health professional} at the selected place(s) in Q51a was very careful to check everything when examining you.<br><br>*-Record 99 if don't know/unsure*	0 = Strongly agree<br>1 = Agree<br>2 = Not sure<br>3 = Disagree<br>4 = Strongly disagree	Categorical ordinal	Patient satisfaction of care services	SAPS 2006
Q60a	At the selected place(s) in Q51a, how satisfied were you with the choices you had in decisions affecting your health care?<br><br>*-Record 99 if don't know/unsure*	0 = Very satisfied<br>1 = Satisfied<br>2 = Neither satisfied nor dissatisfied<br>3 = Dissatisfied<br>4 = Very dissatisfied	Categorical ordinal	Patient satisfaction of care services	SAPS 2006
Q61a	How much of the time did you feel respected by the {doctor/other health professional} at the selected place(s) in Q51a?<br><br>*-Record 99 if don't know/unsure*	0 = All of the time<br>1 = Most of the time<br>2 = About half the time<br>3 = Some of the time<br>4 = None of the time	Categorical ordinal	Patient satisfaction of care services	SAPS 2006
Q62a	At the selected place(s) in Q51a, the time you had with the {doctor/other health professional} was too short.<br><br>*-Record 99 if don't know/unsure*	0 = Strongly agree<br>1 = Agree<br>2 = Not sure<br>3 = Disagree<br>4 = Strongly disagree	Categorical ordinal	Patient satisfaction of care services	SAPS 2006
Q63a	Are you satisfied with the care you received in the selected place(s) in Q51a?<br><br>*-Record 99 if don't know/unsure*	0 = Very satisfied<br>1 = Satisfied<br>2 = Neither satisfied nor dissatisfied<br>3 = Dissatisfied<br>4 = Very dissatisfied	Categorical ordinal	Patient satisfaction of care services	SAPS 2006

Chhim S, *et al. BMJ Open* 2023; 13:e061959. doi: 10.1136/bmjopen-2022-061959

Q64a	Would you recommend the selected place(s) in Q51a to others?<br><br>*-Record 99 if don't know/unsure*	1 = Not recommend<br>2 = Recommend with reservations<br>3 = Recommend<br>4 = Highly recommend	Categorical ordinal	Patient satisfaction of care services	Opinion
Q65a	Have you ever been told by a doctor that you have eyes problems?<br><br>*-Record 99 if don't know/unsure*	0 = No<br>1 = Yes	Quantitative discrete	Complication-HT	Cambodia national guideline
Q66a	Have you ever been told by a doctor that you have kidney problems?<br><br>*-Record 99 if don't know/unsure*	0 = No<br>1 = Yes	Quantitative discrete	Complication-HT	Cambodia national guideline
Q67a	Have you ever been told by a doctor that you have lost the sensation of your peripheral membrane, such as foot, hand, arm?<br><br>*-Record 99 if don't know/unsure*	0 = No<br>1 = Yes	Quantitative discrete	Complication-HT	Cambodia national guideline
	In **the past 3 months**, besides seeking medical advice or treatment for your hypertensive condition, have you sought medical treatment or advice for other illnesses or conditions?<br><br>If No, go to Section 4.	0 = No<br>1 = Yes			

Chhim S, *et al. BMJ Open* 2023; 13:e061959. doi: 10.1136/bmjopen-2022-061959

Q24	Where did you seek medical advice or treatment for that illness in the past 3 months?<br><br>*-More than one answer can be selected.*<br>*-Data collectors can use probes to help respondents determine the types of health facilities in the Response Column.*<br>*-Record 99 if don't know and 88 if refuse*	1= National hospital (PP)<br>2= Provincial hospital (RH)<br>3= District hospital (RH)<br>4= Health centre<br>5= Health post<br>6= Provincial rehabilitation centre (PRC) or Community-based rehabilitation (CBR)<br>7= Other public; specify:<br>8= Private hospital<br>9= Private clinic<br>10= Private pharmacy<br>11= Home/Office of trained health worker/nurse<br>12= Visit of trained health worker/nurse<br>13= Other private medical; specify:<br>14= Shop selling drugs/market<br>15= Kru Khmer/ Magician<br>16= Monk/religious leader<br>17= Traditional birth attendant<br>18= Oversee medical service<br>19= Other; specify:	Categorical Nominal	Care provider_3M	CDHS and GACD book
From Q25-Q39, it is a set of questions that are asked following choices selected in Q24. If 2 or 3 choices were selected in Q24, Q25-Q39 would appear 2 or 3 times, accordingly.					

Q25	How many times did you visit the selected place**(s)** in Q24 in the past three months?	_______ times	Quantitative discrete	Number of visits	GACD
Q26	How much in total was spent on the treatment at the selected place(s) in Q24?<br><br>*-Record 99 if don't know and 88 if refuse*	0 = free/no cost<br>1 = in kind<br>2 = ________Riels	-Categorical nominal<br>-<br>Quantitative Continuous	Cost of treatment	CDHS
Q27	How did you pay for the treatment cost at the selected place(s) in Q24?<br><br>*-Record 99 if don't know and 88 if refuse*	1= Health Equity Fund<br>2= Voucher<br>3= Fee Exemption<br>4= NGO<br>5= National Social Security Fund<br>6= Community-Based Health Insurance<br>7= Health Insurance through Employer<br>8= Other Privately Purchased Commercial Health Insurance<br>9= Wage/income<br>10= Loan/ Ton Tin<br>11= Sale of Assets<br>12= Gift from Relative<br>13= Savings<br>14= Other	-Categorical nominal	Payment method	GADC adapted to context specific

Chhim S, *et al. BMJ Open* 2023; 13:e061959. doi: 10.1136/bmjopen-2022-061959

Q28	How much in total was spent on transport to go to and return from the selected place(s) in Q24?<br><br>*-Record 99 if don't know and 88 if refuse*	0 = free/no cost<br>1 = in kind<br>2 = ________Riels	-Categorical nominal<br>-<br>Quantitative Continuous	Cost of transp ort	CDHS
Q29	On average how many hours do you spend to get treatment/advices from the selected place(s) in Q24?<br><br>*-Record 99 if don't know and 88 if refuse*	________Hours	Quantitative continuous	Time spendi ng	GACD book (Adapted)
Q30	How satisfied are you with the effect of your {treatment/care} at the selected place(s) in Q24?<br><br>*-Record 99 if don't know/unsure*	0 = Very satisfied<br>1 = Satisfied<br>2 = Neither satisfied nor dissatisfied<br>3 = Dissatisfied<br>4 = Very dissatisfied	Categorical ordinal	Patien t satisfa ction of care servic es	SAPS 2006
Q31	How satisfied are you with the explanations the {doctor/other health professional} has given you about the results of your {treatment/care} at the selected place(s) in Q24?<br><br>*-Record 99 if don't know/unsure*	0 = Very satisfied<br>1 = Satisfied<br>2 = Neither satisfied nor dissatisfied<br>3 = Dissatisfied<br>4 = Very dissatisfied	Categorical ordinal	Patien t satisfa ction of care servic es	SAPS 2006
Q32	The {doctor/other health professional} at the selected place(s) in Q24 was very careful to check everything when examining you.<br><br>*-Record 99 if don't know/unsure*	0 = Strongly agree<br>1 = Agree<br>2 = Not sure<br>3 = Disagree<br>4 = Strongly disagree	Categorical ordinal	Patien t satisfa ction of care servic es	SAPS 2006

Chhim S, *et al. BMJ Open* 2023; 13:e061959. doi: 10.1136/bmjopen-2022-061959

Q33	At the selected place(s) in Q24, how satisfied were you with the choices you had in decisions affecting your health care?<br><br>*-Record 99 if don't know/unsure*	0 = Very satisfied<br>1 = Satisfied<br>2 = Neither s atisfied nor dissatisfied<br>3 = Dissatisfied<br>4 = Very dissatisfied	Categorical ordinal	Patien t satisfa ction of care servic es	SAPS 2006
Q34	How much of the time did you feel respected by the {doctor/other health professional} at the selected place(s) in Q24?<br><br>*-Record 99 if don't know/unsure*	0 = All of the time<br>1 = Most of the time<br>2 = About half the time<br>3 = Some of the time<br>4 = None of the time	Categorical ordinal	Patien t satisfa ction of care servic es	SAPS 2006
Q35	At the selected place(s) in Q24, the time you had with the {doctor/other health professional} was too short.<br><br>*-Record 99 if don't know/unsure*	0 = Strongly agree<br>1 = Agree<br>2 = Not sure<br>3 = Disagree<br>4 = Strongly disagree	Categorical ordinal	Patien t satisfa ction of care servic es	SAPS 2006
Q36	Are you satisfied with the care you received in the selected place(s) in Q24?<br><br>*-Record 99 if don't know/unsure*	0 = Very satisfied<br>1 = Satisfied<br>2 = Neither s atisfied nor dissatisfied<br>3 = Dissatisfied<br>4 = Very dissatisfied	Categorical ordinal	Patien t satisfa ction of care servic es	SAPS 2006
Q37	Did you get your blood pressure measured at the selected place(s) in Q24?<br><br>*-Record 99 if don't know/unsure*	0 = No<br>1 = Yes	Categorical variable	Acces s to blood pressu re testing	Opinion

Chhim S, *et al. BMJ Open* 2023; 13:e061959. doi: 10.1136/bmjopen-2022-061959

Q38	Did you get your blood glucose tested at the selected place(s) in Q24?<br><br>*-Record 99 if don't know/unsure*	0 = No<br>1 = Yes	Categorical variable	Access to blood glucose testing	Opinion
Q39	Would you recommend the selected place(s) in Q24 to others?<br><br>*-Record 99 if don't know/unsure*	1 = Not recommended<br>2 = Recommend with reservations<br>3 = Recommend<br>4 = Highly recommend	Categorical ordinal	Patient satisfaction of care services	Opinion

Chhim S, *et al. BMJ Open* 2023; 13:e061959. doi: 10.1136/bmjopen-2022-061959

SECTION 3b: HEALTH CARE UTILIZATION FOR DIABETES					
**Q.N**	**Description and Questions**	**Response**	**Type of Variable**	**Field**	**Ref:**
Q43b	How long have you lived with diabetes?<br><br>*-Record 99 if don't know/unsure and 88 if refuse*<br>*-Less than a year is rounded up to one year*<br>*-Standard rounded up formula is applied.*	________Years	Quantitative concrete	Durati on DM	Opinion
Q44b	Where were you first diagnosed as having diabetes?<br><br>*-Record 99 if don't know and 88 if refuse*	1= National hospital (PP)<br>2= Provincial hospital (RH)<br>3= District hospital (RH)<br>4= Health centre<br>5= Health post<br>6= Provincial rehabilitation centre (PRC) or Community-based rehabilitation (CBR)<br>7= Other public; specify:<br>8= Private hospital<br>9= Private clinic<br>10= Private pharmacy<br>11= Home/Office of trained health worker/nurse<br>12= Visit of trained health worker/nurse<br>13= Other private medical; specify:<br>14= Shop selling drugs/market<br>15= Kru Khmer/ Magician	Categorical Nominal	Diagn osis DM (CoC)	Opinion

Chhim S, *et al. BMJ Open* 2023; 13:e061959. doi: 10.1136/bmjopen-2022-061959

		16= Monk/religious leader<br>17= Traditional birth attendant<br>18= Oversee medical service<br>19= MoPoTsyo<br>20= Other; specify:			
Q45b	Where did you first seek advice or treatment for diabetes after being diagnosed?<br><br>*-Record 99 if don't know and 88 if refuse*	1= National hospital (PP)<br>2= Provincial hospital (RH)<br>3= District hospital (RH)<br>4= Health centre<br>5= Health post<br>6= Provincial rehabilitation centre (PRC) or Community-based rehabilitation (CBR)<br>7= Other public; specify:<br>8= Private hospital<br>9= Private clinic<br>10= Private pharmacy<br>11= Home/Office of trained health worker/nurse<br>12= Visit of trained health worker/nurse<br>13= Other private medical; specify:<br>14= Shop selling drugs/market<br>15= Kru Khmer/ Magician<br>16= Monk/religious leader<br>17= Traditional birth attendant	Categorical Nominal	Link to care DM (CoC)	CDHS (Adapted for disease specific)

		18= Oversee medical service<br>19= MoPoTsyo<br>20= Other; specify:			
Q46b	Did you go to other places for follow up treatment/care for your diabetes conditions?<br><br>*-Record 88 if refuse*<br>*-If NO, please skip Q51b*	0 = No<br>1 = Yes	Categorical binary	Trust DM	CDHS
Q47b	If yes to Q46b, where else did you go to get follow up treatment/care for your diabetes conditions?	1= National hospital (PP)<br>2= Provincial hospital (RH)<br>3= District hospital (RH)<br>4= Health centre<br>5= Health post<br>6= Provincial rehabilitation centre (PRC) or Community-based rehabilitation (CBR)<br>7= Other public; specify:<br>8= Private hospital<br>9= Private clinic<br>10= Private pharmacy<br>11= Home/Office of trained health worker/nurse<br>12= Visit of trained health worker/nurse<br>13= Other private medical; specify:<br>14= Shop selling drugs/market	Categorical Nominal	Link to care DM (CoC)	CDHS (Adapted for disease specific)

Chhim S, *et al. BMJ Open* 2023; 13:e061959. doi: 10.1136/bmjopen-2022-061959

		15= Kru Khmer/ Magician<br>16= Monk/religious leader<br>17= Traditional birth attendant<br>18= Oversee medical service<br>19= MoPoTsyo<br>20= Other; specify:			
Q48b	Did you get treatment/care for your diabetes conditions in the past 12 months?<br><br>*-Record 88 if refuse*<br>*-If NO, please skip Q49b-65b*	0 = No<br>1 = Yes	Categorical binary	Retain in care DM (CoC)	CDHS (Adapted for disease specific)
Q49b	Are you currently receiving any of the following treatment/advices for your diabetes conditions prescribed by a doctor or other health care worker?<br><br>Q. 49b.1. Insulin [____]<br>Q. 49b.2. Drugs (medication) that you have taken in the past two weeks [____]<br>Q. 49b.3. Special prescribed diet [____]<br>Q. 49b.4. Advice or treatment to lose weight [____]<br>Q. 49b.5. Advice or treatment to stop smoking<br>Q. 49b.6. Advice to start or do more physical exercise	0 = No 1= Yes	Categorical ordinal	In treatm ent for DM (CoC)	STEPS Survey
Q50b	Have you had your blood glucose measured in the past 12 months?<br><br>*-Record 99 if don't know and 88 if refuse*	0 = No 1= Yes	Categorical ordinal	In treatm ent for DM (CoC)	Veerle's suggestion

Chhim S, *et al. BMJ Open* 2023; 13:e061959. doi: 10.1136/bmjopen-2022-061959

Q51b	Have you had your HbA1c tested in the past 12 months?<br><br>*-Record 99 if don't know and 88 if refuse*	0 = No 1= Yes	Categorical ordinal	In treatment for DM (CoC)	Veerle's suggestion
Q52b	Where did you seek medical advice or treatment for illness in the **past 3 months**?<br><br>***-More than one answer can be selected.***<br>*-Data collectors can use probes to help respondents determine the types of health facilities in the Response Column.*<br>*-Record 99 if don't know and 88 if refuse*<br>*-If 21=Nowhere, go to Q66b.*	1= National hospital (PP)<br>2= Provincial hospital (RH)<br>3= District hospital (RH)<br>4= Health centre<br>5= Health post<br>6= Provincial rehabilitation centre (PRC) or Community-based rehabilitation (CBR)<br>7= Other public; specify:<br>8= Private hospital<br>9= Private clinic<br>10= Private pharmacy<br>11= Home/Office of trained health worker/nurse<br>12= Visit of trained health worker/nurse<br>13= Other private medical; specify:<br>14= Shop selling drugs/market<br>15= Kru Khmer/ Magician<br>16= Monk/religious leader<br>17= Traditional birth attendant<br>18= Oversee medical service	Categorical Nominal	Care provider_3M	CDHS and GACD book

Chhim S, *et al. BMJ Open* 2023; 13:e061959. doi: 10.1136/bmjopen-2022-061959

		19= MoPoTsyo<br>20= Other; specify:<br>21= No where			
From Q53b-Q65b, it is a set of questions that are asked following choices selected in Q52b. If 2 or 3 choices were selected in Q52b, Q53b-Q65b would appear 2 or 3 times, accordingly.					
Q53b	How many times did you visit the selected place**(s)** in Q52b in the past three months?	_______ times	Quantitative discrete	Number of visits	GACD
Q54b	How much in total was spent on the treatment at the selected place(s) in Q52b?<br><br>*-Record 99 if don't know and 88 if refuse*	0 = free/no cost<br>1 = in kind<br>2 = ________Riels<br>OR<br>________ USD	-Categorical nominal<br>-<br>Quantitative Continuous	Cost of treatment	CDHS
Q55b	How did you pay for the treatment cost at the selected place(s) in Q52b?<br><br>*-Record 99 if don't know and 88 if refuse*	1= Health Equity Fund<br>2= Voucher<br>3= Fee Exemption<br>4= NGO<br>5= National Social Security Fund<br>6= Community-Based Health Insurance<br>7= Health Insurance through Employer<br>8= Other Privately	-Categorical nominal	Payment method	GADC adapted to context specific

		Purchased Commercial Health Insurance<br>9= Wage/income<br>10= Loan/ Ton Tin<br>11= Sale of Assets<br>12= Gift from Relative<br>13= Savings<br>14= Other			
Q56b	How much in total was spent on transport to go to and return from the selected place(s) in Q52b?<br><br>*-Record 99 if don't know and 88 if refuse*	0 = free/no cost<br>1 = in kind<br>2 = ________Riels OR ________ USD	-Categorical nominal<br>-<br>Quantitative Continuous	Cost of transport	CDHS
Q57b	On average how many hours do you spend to get treatment/advices from the selected place(s) in Q52b?<br><br>*-Record 99 if don't know and 88 if refuse*	________Hours	Quantitative continuous	Time spending	GACD book (Adapted)
Q58b	How satisfied are you with the effect of your {treatment/care} at the selected place(s) in Q52b?<br><br>*-Record 99 if don't know/unsure*	0 = Very satisfied<br>1 = Satisfied<br>2 = Neither satisfied nor dissatisfied<br>3 = Dissatisfied<br>4 = Very dissatisfied	Categorical ordinal	Patient satisfaction of care service	SAPS 2006
Q59b	How satisfied are you with the explanations the {doctor/other health professional} has given you about the results of your {treatment/care} at the selected place(s) in Q52b?<br><br>*-Record 99 if don't know/unsure*	0 = Very satisfied<br>1 = Satisfied<br>2 = Neither satisfied nor dissatisfied<br>3 = Dissatisfied<br>4 = Very d issatisfied	Categorical ordinal	Patient satisfaction of care service	SAPS 2006

Q60b	The {doctor/other health professional} at the selected place(s) in Q52b was very careful to check everything when examining you.<br><br>*-Record 99 if don't know/unsure*	0 = Strongly agree<br>1 = Agree<br>2 = Not sure<br>3 = Disagree<br>4 = Strongly disagree	Categorical ordinal	Patient satisfaction of care service	SAPS 2006
Q61b	At the selected place(s) in Q52b, how satisfied were you with the choices you had in decisions affecting your health care?<br><br>*-Record 99 if don't know/unsure*	0 = Very satisfied<br>1 = Satisfied<br>2 = Neither satisfied nor dissatisfied<br>3 = Dissatisfied<br>4 = Very dissatisfied	Categorical ordinal	Patient satisfaction of care service	SAPS 2006
Q62b	How much of the time did you feel respected by the {doctor/other health professional} at the selected place(s) in Q52b?<br><br>*-Record 99 if don't know/unsure*	0 = All of the time<br>1 = Most of the time<br>2 = About half the time<br>3 = Some of the time<br>4 = None of the time	Categorical ordinal	Patient satisfaction of care service	SAPS 2006
Q63b	At the selected place(s) in Q52b, the time you had with the {doctor/other health professional} was too short.<br><br>*-Record 99 if don't know/unsure*	0 = Strongly agree<br>1 = Agree<br>2 = Not sure<br>3 = Disagree<br>4 = Strongly disagree	Categorical ordinal	Patient satisfaction of care service	SAPS 2006
Q64b	Are you satisfied with the care you received in the selected place(s) in Q52b?<br><br>*-Record 99 if don't know/unsure*	0 = Very satisfied<br>1 = Satisfied<br>2 = Neither satisfied nor dissatisfied<br>3 = Dissatisfied<br>4 = Very dissatisfied	Categorical ordinal	Patient satisfaction of care service	SAPS 2006

Chhim S, *et al. BMJ Open* 2023; 13:e061959. doi: 10.1136/bmjopen-2022-061959

Q65b	Would you recommend the selected place(s) in Q52b to others?<br><br>*-Record 99 if don't know/unsure*	1 = Not recommended<br>2 = Recommend with reservations<br>3 = Recommend<br>4 = Highly recommend	Categorical ordinal	Patient satisfaction of care services	Opinion
Q66b	Have you ever been told by a doctor that you have eyes problems?<br><br>*-Record 99 if don't know/unsure*	0 = No<br>1 = Yes	Quantitative discrete	Complication-DM	Cambodia national guideline
Q67b	Have you ever been told by a doctor that you have kidney problems?<br><br>*-Record 99 if don't know/unsure*	0 = No<br>1 = Yes	Quantitative discrete	Complication-DM	Cambodia national guideline
Q68b	Have you ever been told by a doctor that you have lost the sensation of your peripheral membrane, such as foot, hand, arm?<br><br>*-Record 99 if don't know/unsure*	0 = No<br>1 = Yes	Quantitative discrete	Complication-DM	Cambodia national guideline
	In **the past 3 months**, besides seeking medical advice or treatment for your diabetes condition, have you sought medical treatment or advice for other illnesses or conditions?<br><br>If No, go to Section 4.	0 = No<br>1 = Yes			

Q24	Where did you seek medical advice or treatment for that illness in the past 3 months?<br><br>*-More than one answer can be selected.*<br>*-Data collectors can use probes to help respondents determine the types of health facilities in the Response Column.*<br>*-Record 99 if don't know and 88 if refuse*	1= National hospital (PP)<br>2= Provincial hospital (RH)<br>3= District hospital (RH)<br>4= Health centre<br>5= Health post<br>6= Provincial rehabilitation centre (PRC) or Community-based rehabilitation (CBR)<br>7= Other public; specify:<br>8= Private hospital<br>9= Private clinic<br>10= Private pharmacy<br>11= Home/Office of trained health worker/nurse<br>12= Visit of trained health worker/nurse<br>13= Other private medical; specify:<br>14= Shop selling drugs/market<br>15= Kru Khmer/ Magician<br>16= Monk/religious leader<br>17= Traditional birth attendant<br>18= Oversee medical service<br>19= Other; specify:	Categorical Nominal	Care provider_3M	CDHS and GACD book
From Q25-Q39, it is a set of questions that are asked following choices selected in Q24. If 2 or 3 choices were selected in Q24, Q25-Q39 would appear 2 or 3 times, accordingly.					

Q25	How many times did you visit the selected place**(s)** in Q24 in the past three months?	_______ times	Quantitative discrete	Numb er of visits	GACD
Q26	How much in total was spent on the treatment at the selected place(s) in Q24?<br><br>*-Record 99 if don't know and 88 if refuse*	0 = free/no cost<br>1 = in kind<br>2 = ________Riels	-Categorical nominal<br>-<br>Quantitative Continuous	Cost of treatm ent	CDHS
Q27	How did you pay for the treatment cost at the selected place(s) in Q24?<br><br>*-Record 99 if don't know and 88 if refuse*	1= Health Equity Fund<br>2= Voucher<br>3= Fee Exemption<br>4= NGO<br>5= National Social Security Fund<br>6= Community-Based Health Insurance<br>7= Health Insurance through Employer<br>8= Other Privately Purchased Commercial Health Insurance<br>9= Wage/income<br>10= Loan/ Ton Tin<br>11= Sale of Assets<br>12= Gift from Relative<br>13= Savings<br>14= Other	-Categorical nominal	Paym ent metho d	GADC adapted to context specific

Chhim S, *et al. BMJ Open* 2023; 13:e061959. doi: 10.1136/bmjopen-2022-061959

Q28	How much in total was spent on transport to go to and return from the selected place(s) in Q24?<br><br>*-Record 99 if don't know and 88 if refuse*	0 = free/no cost<br>1 = in kind<br>2 = ________Riels	-Categorical nominal<br>-<br>Quantitative Continuous	Cost of transp ort	CDHS
Q29	On average how many hours do you spend to get treatment/advices from the selected place(s) in Q24?<br><br>*-Record 99 if don't know and 88 if refuse*	________Hours	Quantitative continuous	Time spendi ng	GACD book (Adapted)
Q30	How satisfied are you with the effect of your {treatment/care} at the selected place(s) in Q24?<br><br>*-Record 99 if don't know/unsure*	0 = Very satisfied<br>1 = Satisfied<br>2 = Neither satisfied nor dissatisfied<br>3 = Dissatisfied<br>4 = Very dissatisfied	Categorical ordinal	Patien t satisfa ction of care servic es	SAPS 2006
Q31	How satisfied are you with the explanations the {doctor/other health professional} has given you about the results of your {treatment/care} at the selected place(s) in Q24?<br><br>*-Record 99 if don't know/unsure*	0 = Very satisfied<br>1 = Satisfied<br>2 = Neither satisfied nor dissatisfied<br>3 = Dissatisfied<br>4 = Very dissatisfied	Categorical ordinal	Patien t satisfa ction of care servic es	SAPS 2006
Q32	The {doctor/other health professional} at the selected place(s) in Q24 was very careful to check everything when examining you.<br><br>*-Record 99 if don't know/unsure*	0 = Strongly agree<br>1 = Agree<br>2 = Not sure<br>3 = Disagree<br>4 = Strongly disagree	Categorical ordinal	Patien t satisfa ction of care servic es	SAPS 2006

Chhim S, *et al. BMJ Open* 2023; 13:e061959. doi: 10.1136/bmjopen-2022-061959

Q33	At the selected place(s) in Q24, how satisfied were you with the choices you had in decisions affecting your health care?<br><br>*-Record 99 if don't know/unsure*	0 = Very satisfied<br>1 = Satisfied<br>2 = Neither s atisfied nor dissatisfied<br>3 = Dissatisfied<br>4 = Very dissatisfied	Categorical ordinal	Patient satisfaction of care services	SAPS 2006
Q34	How much of the time did you feel respected by the {doctor/other health professional} at the selected place(s) in Q24?<br><br>*-Record 99 if don't know/unsure*	0 = All of the time<br>1 = Most of the time<br>2 = About half the time<br>3 = Some of the time<br>4 = None of the time	Categorical ordinal	Patient satisfaction of care services	SAPS 2006
Q35	At the selected place(s) in Q24, the time you had with the {doctor/other health professional} was too short.<br><br>*-Record 99 if don't know/unsure*	0 = Strongly agree<br>1 = Agree<br>2 = Not sure<br>3 = Disagree<br>4 = Strongly disagree	Categorical ordinal	Patient satisfaction of care services	SAPS 2006
Q36	Are you satisfied with the care you received in the selected place(s) in Q24?<br><br>*-Record 99 if don't know/unsure*	0 = Very satisfied<br>1 = Satisfied<br>2 = Neither s atisfied nor dissatisfied<br>3 = Dissatisfied<br>4 = Very dissatisfied	Categorical ordinal	Patient satisfaction of care services	SAPS 2006
Q37	Did you get your blood pressure measured at the selected place(s) in Q24?<br><br>*-Record 99 if don't know/unsure*	0 = No<br>1 = Yes	Categorical variable	Access to blood pressure testing	Opinion

Q38	Did you get your blood glucose tested at the selected place(s) in Q24?<br><br>*-Record 99 if don't know/unsure*	0 = No<br>1 = Yes	Categorical variable	Acces s to blood glucos e testing	Opinion
Q39	Would you recommend the selected place(s) in Q24 to others?<br><br>*-Record 99 if don't know/unsure*	1 = Not recommended<br>2 = Recommend with reservations<br>3 = Recommend<br>4 = Highly recommend	Categorical ordinal	Patien t satisfa ction of care servic es	Opinion

SECTION 3c: HEALTH CARE UTILIZATION FOR DIABETES AND HYPERTENSION					
**Q.N**	**Description and Questions**	**Response**	**Type of Variable**	**Field**	**Ref:**
Q43c	How long have you lived with diabetes?<br><br>*-Record 99 if don't know/unsure and 88 if refuse*<br>*-Less than a year is rounded up to one year*<br>*-Standard rounded up formula is applied.*	________Years	Quantitative concrete	Duration DM and HT	Opinion
Q44c	Where were you first diagnosed as having diabetes?<br><br>*-Record 99 if don't know and 88 if refuse*	1= National hospital (PP)<br>2= Provincial hospital (RH)<br>3= District hospital (RH)<br>4= Health centre<br>5= Health post<br>6= Provincial rehabilitation centre (PRC) or Community-based rehabilitation (CBR)<br>7= Other public; specify:<br>8= Private hospital<br>9= Private clinic<br>10= Private pharmacy<br>11= Home/Office of trained health worker/nurse<br>12= Visit of trained health worker/nurse<br>13= Other private medical; specify:<br>14= Shop selling drugs/market<br>15= Kru Khmer/ Magician	Categorical Nominal	Diagnosis DM (CoC)	Opinion

Chhim S, *et al. BMJ Open* 2023; 13:e061959. doi: 10.1136/bmjopen-2022-061959

		16= Monk/religious leader<br>17= Traditional birth attendant<br>18= Oversee medical service<br>19= MoPoTsyo<br>20= Other; specify:			
Q45c	Where did you first seek advice or treatment for diabetes after being diagnosed?<br><br>*-Record 99 if don't know and 88 if refuse*	1= National hospital (PP)<br>2= Provincial hospital (RH)<br>3= District hospital (RH)<br>4= Health centre<br>5= Health post<br>6= Provincial rehabilitation centre (PRC) or Community-based rehabilitation (CBR)<br>7= Other public; specify:<br>8= Private hospital<br>9= Private clinic<br>10= Private pharmacy<br>11= Home/Office of trained health worker/nurse<br>12= Visit of trained health worker/nurse<br>13= Other private medical; specify:<br>14= Shop selling drugs/market<br>15= Kru Khmer/ Magician<br>16= Monk/religious leader<br>17= Traditional birth attendant	Categorical Nominal	Link to care DM (CoC)	CDHS (Adapted for disease specific)

		18= Oversee medical service<br>19= MoPoTsyo<br>20= Other; specify:			
Q46c	How long have you lived with hypertension?<br><br>*-Record 99 if don't know/unsure and 88 if refuse*<br>*-Less than a year is rounded up to one year*<br>*-Standard rounded up formula is applied.*	________Years	Quantitative concrete	Duration DM and HT	Opinion
Q47c	Where were you first diagnosed as having hypertension?<br><br>*-Record 99 if don't know and 88 if refuse*	1= National hospital (PP)<br>2= Provincial hospital (RH)<br>3= District hospital (RH)<br>4= Health centre<br>5= Health post<br>6= Provincial rehabilitation centre (PRC) or Community-based rehabilitation (CBR)<br>7= Other public; specify:<br>8= Private hospital<br>9= Private clinic<br>10= Private pharmacy<br>11= Home/Office of trained health worker/nurse<br>12= Visit of trained health worker/nurse<br>13= Other private medical; specify:<br>14= Shop selling drugs/market	Categorical Nominal	Diagnosis HT (CoC)	Opinion

		15= Kru Khmer/ Magician<br>16= Monk/religious leader<br>17= Traditional birth attendant<br>18= Oversee medical service<br>19= MoPoTsyo<br>20= Other; specify:			
Q48c	Where did you first seek advice or treatment for hypertension after being diagnosed?<br><br>*-Record 99 if don't know and 88 if refuse*	1= National hospital (PP)<br>2= Provincial hospital (RH)<br>3= District hospital (RH)<br>4= Health centre<br>5= Health post<br>6= Provincial rehabilitation centre (PRC) or Community-based rehabilitation (CBR)<br>7= Other public; specify:<br>8= Private hospital<br>9= Private clinic<br>10= Private pharmacy<br>11= Home/Office of trained health worker/nurse<br>12= Visit of trained health worker/nurse<br>13= Other private medical; specify:<br>14= Shop selling drugs/market<br>15= Kru Khmer/ Magician<br>16= Monk/religious leader	Categorical Nominal	Link to care HT (CoC)	CDHS (Adapted for disease specific)

		17= Traditional birth attendant<br>18= Oversee medical service<br>19= MoPoTsyo<br>20= Other; specify:			
Q49c	Did you go to other places for follow up treatment/care for your hypertensive and diabetes conditions?<br><br>*-Record 88 if refuse*<br>*-If NO, please skip Q50c*	0 = No<br>1 = Yes	Categorical binary	Trust DM and HT	CDHS
Q50c	If yes to Q49c, where else did you go to get follow up treatment/care for both conditions?	1= National hospital (PP)<br>2= Provincial hospital (RH)<br>3= District hospital (RH)<br>4= Health centre<br>5= Health post<br>6= Provincial rehabilitation centre (PRC) or Community-based rehabilitation (CBR)<br>7= Other public; specify:<br>8= Private hospital<br>9= Private clinic<br>10= Private pharmacy<br>11= Home/Office of trained health worker/nurse<br>12= Visit of trained health worker/nurse<br>13= Other private medical; specify:<br>14= Shop selling drugs/market	Categorical Nominal	Link to care DM and HT (CoC)	CDHS (Adapted for disease specific)

		15= Kru Khmer/ Magician<br>16= Monk/religious leader<br>17= Traditional birth attendant<br>18= Oversee medical service<br>19= MoPoTsyo<br>20= Other; specify:			
Q51c	Did you get treatment/care for both conditions in the past 12 months?<br><br>*-Record 88 if refuse*<br>*-If NO, please skip Q52c-69c*	0 = No<br>1 = Yes	Categorical binary	Retain in care DM and HT (CoC)	CDHS (Adapted for disease specific)
Q52c	Are you currently receiving any of the following treatment/advices for both conditions prescribed by a doctor or other health care worker?<br><br>Q. 52c.1. Insulin [____]<br>Q. 52c.2. Drugs (medication) that you have taken in the past two weeks [____]<br>Q. 52c.3. Special prescribed diet [____]<br>Q. 52c.4. Advice or treatment to lose weight [____]<br>Q. 52c.5. Advice or treatment to stop smoking<br>Q. 52c.6. Advice to reduce salt intake<br>Q. 52c.7. Advice to start or do more physical exercise	0 = No 1= Yes	Categorical ordinal	In treatment for DM and HT (CoC)	STEPS Survey
Q53c	Have you had your blood glucose measured in the past 12 months?<br><br>*-Record 99 if don't know and 88 if refuse*	0 = No 1= Yes	Categorical ordinal	In treatment for DM (CoC)	Veerle's suggestion

Chhim S, *et al. BMJ Open* 2023; 13:e061959. doi: 10.1136/bmjopen-2022-061959

Q54c	Have you had your HbA1c tested in the past 12 months?<br><br>*-Record 99 if don't know and 88 if refuse*	0 = No 1= Yes	Categorical ordinal	In treatment for DM (CoC)	Veerle's suggestion
Q55c	Have you had your blood cholesterol measured in the past 12 months?<br><br>*-Record 99 if don't know and 88 if refuse*	0 = No 1= Yes	Categorical ordinal	In treatment for HT (CoC)	Veerle's suggestion
Q56c	Where did you seek medical advice or treatment for your conditions in **the past 3 months?**<br><br>-***More than one answer can be selected***.<br>*-Data collectors can use probes to help respondents determine the types of health facilities in the Response Column.*<br>*-Record 99 if don't know and 88 if refuse*<br>*-If 21=Nowhere, go to Q70c.*	1= National hospital (PP)<br>2= Provincial hospital (RH)<br>3= District hospital (RH)<br>4= Health centre<br>5= Health post<br>6= Provincial rehabilitation centre (PRC) or Community-based rehabilitation (CBR)<br>7= Other public; specify:<br>8= Private hospital<br>9= Private clinic<br>10= Private pharmacy<br>11= Home/Office of trained health worker/nurse<br>12= Visit of trained health worker/nurse<br>13= Other private medical; specify:<br>14= Shop selling drugs/market	Categorical Nominal	Care provider_3M	CDHS and GACD book

		15= Kru Khmer/ Magician<br>16= Monk/religious leader<br>17= Traditional birth attendant<br>18= Oversee medical service<br>19= MoPoTsyo<br>20= Other; specify:<br>21= Nowhere			
From Q57c-Q69c, it is a set of questions that are asked following choices selected in Q56c. If 2 or 3 choices were selected in Q56c, Q57c-Q69c would appear 2 or 3 times, accordingly.					
Q57c	How many times did you visit the selected place(**s**) in Q56c in the past three months?	_______ times	Quantitative discrete	Numb er of visits	GACD
Q58c	How much in total was spent on the treatment at the selected place(s) in Q56c?<br><br>*-Record 99 if don't know and 88 if refuse*	0 = free/no cost<br>1 = in kind<br>2 = ________Riels<br>OR<br>________ USD	-Categorical nominal<br>-<br>Quantitative Continuous	Cost of treatm ent	CDHS
Q59c	How did you pay for the treatment cost at the selected place(s) in Q56c?<br><br>*-Record 99 if don't know and 88 if refuse*	1= Health Equity Fund<br>2= Voucher<br>3= Fee Exemption<br>4= NGO<br>5= National Social Security Fund<br>6= Community-Based Health Insurance<br>7= Health Insurance through Employer<br>8= Other Privately	-Categorical nominal	Paym ent metho d	GADC adapted to context specific

		Purchased Commercial Health Insurance<br>9= Wage/income<br>10= Loan/ Ton Tin<br>11= Sale of Assets<br>12= Gift from Relative<br>13= Savings<br>14= Other			
Q60c	How much in total was spent on transport to go to and return from the selected place(s) in Q56c?<br><br>*-Record 99 if don't know and 88 if refuse*	0 = free/no cost<br>1 = in kind<br>2 = ________Riels<br>OR<br>________ USD	-Categorical nominal<br>-<br>Quantitative Continuous	Cost of transp ort	CDHS
Q61c	On average how many hours do you spend to get treatment/advices from the selected place(s) in Q56c?<br><br>*-Record 99 if don't know and 88 if refuse*	________Hours	Quantitative continuous	Time spendi ng	GACD book (Adapted)
Q62c	How satisfied are you with the effect of your {treatment/care} at the selected place(s) in Q56c?<br><br>*-Record 99 if don't know/unsure*	0 = Very satisfied<br>1 = Satisfied<br>2 = Neither satisfied nor dissatisfied<br>3 = Dissatisfied<br>4 = Very dissatisfied	Categorical ordinal	Patien t satisfa ction of care servic e	SAPS 2006
Q63c	How satisfied are you with the explanations the {doctor/other health professional} has given you about the results of your {treatment/care} at the selected place(s) in Q56c?<br><br>*-Record 99 if don't know/unsure*	0 = Very satisfied<br>1 = Satisfied<br>2 = Neither satisfied nor dissatisfied<br>3 = Dissatisfied<br>4 = Very d issatisfied	Categorical ordinal	Patien t satisfa ction of care servic e	SAPS 2006

Chhim S, *et al. BMJ Open* 2023; 13:e061959. doi: 10.1136/bmjopen-2022-061959

Q64c	The {doctor/other health professional} at the selected place(s) in Q56c was very careful to check everything when examining you.<br><br>*-Record 99 if don't know/unsure*	0 = Strongly agree<br>1 = Agree<br>2 = Not sure<br>3 = Disagree<br>4 = Strongly disagree	Categorical ordinal	Patient satisfaction of care service	SAPS 2006
Q65c	At the selected place(s) in Q56c, how satisfied were you with the choices you had in decisions affecting your health care?<br><br>*-Record 99 if don't know/unsure*	0 = Very satisfied<br>1 = Satisfied<br>2 = Neither satisfied nor dissatisfied<br>3 = Dissatisfied<br>4 = Very dissatisfied	Categorical ordinal	Patient satisfaction of care service	SAPS 2006
Q66c	How much of the time did you feel respected by the {doctor/other health professional} at the selected place(s) in Q56c?<br><br>*-Record 99 if don't know/unsure*	0 = All of the time<br>1 = Most of the time<br>2 = About half the time<br>3 = Some of the time<br>4 = None of the time	Categorical ordinal	Patient satisfaction of care service	SAPS 2006
Q67c	At the selected place(s) in Q56c, the time you had with the {doctor/other health professional} was too short.<br><br>*-Record 99 if don't know/unsure*	0 = Strongly agree<br>1 = Agree<br>2 = Not sure<br>3 = Disagree<br>4 = Strongly disagree	Categorical ordinal	Patient satisfaction of care service	SAPS 2006
Q68c	Are you satisfied with the care you received in the selected place(s) in Q56c?<br><br>*-Record 99 if don't know/unsure*	0 = Very satisfied<br>1 = Satisfied<br>2 = Neither satisfied nor dissatisfied<br>3 = Dissatisfied<br>4 = Very dissatisfied	Categorical ordinal	Patient satisfaction of care service	SAPS 2006

Chhim S, *et al. BMJ Open* 2023; 13:e061959. doi: 10.1136/bmjopen-2022-061959

Q69c	Would you recommend the selected place(s) in Q56c to others?<br><br>*-Record 99 if don't know/unsure*	1 = Not recommended<br>2 = Recommend with reservations<br>3 = Recommend<br>4 = Highly recommend	Categorical ordinal	Patient satisfaction of care services	Opinion
Q70c	Have you ever been told by a doctor that you have eyes problems?<br><br>*-Record 99 if don't know/unsure*	0 = No<br>1 = Yes	Quantitative discrete	Complication-DM and HT	Cambodia national guideline
Q71c	Have you ever been told by a doctor that you have kidney problems?<br><br>*-Record 99 if don't know/unsure*	0 = No<br>1 = Yes	Quantitative discrete	Complication-DM and HT	Cambodia national guideline
Q72c	Have you ever been told by a doctor that you have lost the sensation of your peripheral membrane, such as foot, hand, arm?<br><br>*-Record 99 if don't know/unsure*	0 = No<br>1 = Yes	Quantitative discrete	Complication-DM and HT	Cambodia national guideline
	In **the past 3 months**, besides seeking medical advice or treatment for your hypertensive and diabetes condition, have you sought medical treatment or advice for other illnesses or conditions?<br><br>If No, go to Section 4.	0 = No<br>1 = Yes			

Chhim S, *et al. BMJ Open* 2023; 13:e061959. doi: 10.1136/bmjopen-2022-061959

Q24	Where did you seek medical advice or treatment for that illness in the past 3 months?<br><br>*-More than one answer can be selected.*<br>*-Data collectors can use probes to help respondents determine the types of health facilities in the Response Column.*<br>*-Record 99 if don't know and 88 if refuse*	1= National hospital (PP)<br>2= Provincial hospital (RH)<br>3= District hospital (RH)<br>4= Health centre<br>5= Health post<br>6= Provincial rehabilitation centre (PRC) or Community-based rehabilitation (CBR)<br>7= Other public; specify:<br>8= Private hospital<br>9= Private clinic<br>10= Private pharmacy<br>11= Home/Office of trained health worker/nurse<br>12= Visit of trained health worker/nurse<br>13= Other private medical; specify:<br>14= Shop selling drugs/market<br>15= Kru Khmer/ Magician<br>16= Monk/religious leader<br>17= Traditional birth attendant<br>18= Oversee medical service<br>19= Other; specify:	Categorical Nominal	Care provider_3M	CDHS and GACD book
From Q25-Q39, it is a set of questions that are asked following choices selected in Q24. If 2 or 3 choices were selected in Q24, Q25-Q39 would appear 2 or 3 times, accordingly.					

Q25	How many times did you visit the selected place**(s)** in Q24 in the past three months?	_______ times	Quantitative discrete	Number of visits	GACD
Q26	How much in total was spent on the treatment at the selected place(s) in Q24?<br><br>*-Record 99 if don't know and 88 if refuse*	0 = free/no cost<br>1 = in kind<br>2 = ________Riels	-Categorical nominal<br>-Quantitative Continuous	Cost of treatment	CDHS
Q27	How did you pay for the treatment cost at the selected place(s) in Q24?<br><br>*-Record 99 if don't know and 88 if refuse*	1= Health Equity Fund<br>2= Voucher<br>3= Fee Exemption<br>4= NGO<br>5= National Social Security Fund<br>6= Community-Based Health Insurance<br>7= Health Insurance through Employer<br>8= Other Privately Purchased Commercial Health Insurance<br>9= Wage/income<br>10= Loan/ Ton Tin<br>11= Sale of Assets<br>12= Gift from Relative<br>13= Savings<br>14= Other	-Categorical nominal	Payment method	GADC adapted to context specific

Chhim S, *et al. BMJ Open* 2023; 13:e061959. doi: 10.1136/bmjopen-2022-061959

Q28	How much in total was spent on transport to go to and return from the selected place(s) in Q24?<br><br>*-Record 99 if don't know and 88 if refuse*	0 = free/no cost<br>1 = in kind<br>2 = ________Riels	-Categorical nominal<br>-<br>Quantitative Continuous	Cost of transport	CDHS
Q29	On average how many hours do you spend to get treatment/advices from the selected place(s) in Q24?<br><br>*-Record 99 if don't know and 88 if refuse*	________Hours	Quantitative continuous	Time spending	GACD book (Adapted)
Q30	How satisfied are you with the effect of your {treatment/care} at the selected place(s) in Q24?<br><br>*-Record 99 if don't know/unsure*	0 = Very satisfied<br>1 = Satisfied<br>2 = Neither satisfied nor dissatisfied<br>3 = Dissatisfied<br>4 = Very dissatisfied	Categorical ordinal	Patient satisfaction of care services	SAPS 2006
Q31	How satisfied are you with the explanations the {doctor/other health professional} has given you about the results of your {treatment/care} at the selected place(s) in Q24?<br><br>*-Record 99 if don't know/unsure*	0 = Very satisfied<br>1 = Satisfied<br>2 = Neither satisfied nor dissatisfied<br>3 = Dissatisfied<br>4 = Very dissatisfied	Categorical ordinal	Patient satisfaction of care services	SAPS 2006
Q32	The {doctor/other health professional} at the selected place(s) in Q24 was very careful to check everything when examining you.<br><br>*-Record 99 if don't know/unsure*	0 = Strongly agree<br>1 = Agree<br>2 = Not sure<br>3 = Disagree<br>4 = Strongly disagree	Categorical ordinal	Patient satisfaction of care services	SAPS 2006

Q33	At the selected place(s) in Q24, how satisfied were you with the choices you had in decisions affecting your health care?<br><br>*-Record 99 if don't know/unsure*	0 = Very satisfied<br>1 = Satisfied<br>2 = Neither s atisfied nor dissatisfied<br>3 = Dissatisfied<br>4 = Very dissatisfied	Categorical ordinal	Patient satisfaction of care services	SAPS 2006
Q34	How much of the time did you feel respected by the {doctor/other health professional} at the selected place(s) in Q24?<br><br>*-Record 99 if don't know/unsure*	0 = All of the time<br>1 = Most of the time<br>2 = About half the time<br>3 = Some of the time<br>4 = None of the time	Categorical ordinal	Patient satisfaction of care services	SAPS 2006
Q35	At the selected place(s) in Q24, the time you had with the {doctor/other health professional} was too short.<br><br>*-Record 99 if don't know/unsure*	0 = Strongly agree<br>1 = Agree<br>2 = Not sure<br>3 = Disagree<br>4 = Strongly disagree	Categorical ordinal	Patient satisfaction of care services	SAPS 2006
Q36	Are you satisfied with the care you received in the selected place(s) in Q24?<br><br>*-Record 99 if don't know/unsure*	0 = Very satisfied<br>1 = Satisfied<br>2 = Neither s atisfied nor dissatisfied<br>3 = Dissatisfied<br>4 = Very dissatisfied	Categorical ordinal	Patient satisfaction of care services	SAPS 2006
Q37	Did you get your blood pressure measured at the selected place(s) in Q24?<br><br>*-Record 99 if don't know/unsure*	0 = No<br>1 = Yes	Categorical variable	Access to blood pressure testing	Opinion

Q38	Did you get your blood glucose tested at the selected place(s) in Q24?<br><br>*-Record 99 if don't know/unsure*	0 = No<br>1 = Yes	Categorical variable	Acces s to blood glucos e testing	Opinion
Q39	Would you recommend the selected place(s) in Q24 to others?<br><br>*-Record 99 if don't know/unsure*	1 = Not recommended<br>2 = Recommend with reservations<br>3 = Recommend<br>4 = Highly recommend	Categorical ordinal	Patien t satisfa ction of care servic es	Opinion

Chhim S, *et al. BMJ Open* 2023; 13:e061959. doi: 10.1136/bmjopen-2022-061959
