# Supplementary material for: Healthcare usage and expenditure among people with type 2 diabetes and/or hypertension in Cambodia: results from a cross-sectional survey
Source: BMJ Open. 2023 Jan 12;13(1):e061959. doi: 10.1136/bmjopen-2022-061959 (PMC9843177; doi:10.1136/bmjopen-2022-061959)
Supplement: Supplementary data [file bmjopen-2022-061959supp004.pdf]

Annexure 4. Arithmetic and the geometric mean of medical cost by participant characteristics in Cambodia, 2020

Table S1. Arithmetic mean of medical cost by participant characteristics in Cambodia, 2020

Variable	Overall	No T2D/HTN	HTN	T2D	T2D plus HTN
	Mean [95% CI]	Mean [95% CI]	Mean [95% CI]	Mean [95% CI]	Mean [95% CI]
Sex					
Male	16.8 [15.2–18.5]	16.8 [14.7–18.9]	15.5 [12.3–18.6]	25.5 [15.8–35.2]	17.0 [10.2–23.7]
Female	19.5 [18.3–20.6]	22.6 [20.8–24.4]	14.3 [12.7–15.8]	21.1 [16.0–26.2]	22.2 [18.9–25.5]
P-value	0.015	<0.001	0.480	0.393	0.212
Age in years					
40–49	19.0 [16.8–21.2]	20.6 [18.1–23.2]	20.6 [18.1–23.2]	10.3 [6.5–14.0]	19.7 [7.1–32.4]
50–59	19.1 [17.5–20.7]	82.8 [73.0–92.6]	20.7 [18.3–23.2]	14.2 [12.0–16.5]	24.5 [17.6–31.3]
60+	18.2 [16.8–19.7]	82.1 [72.8–91.4]	20.5 [18.2–22.9]	15.4 [13.4–17.4]	21.1 [13.8–28.4]
P-value	0.698	0.995	0.120	0.704	0.850
Educational level					
No schooling	19.1 [17.4–20.9]	20.6 [17.9–23.3]	16.2 [13.8–18.7]	22.8 [14.8–30.7]	22.1 [15.8–28.5]
Primary	17.9 [16.7–19.1]	20.3 [18.5–22.1]	12.9 [11.1–14.7]	20.5 [15.1–26.0]	19.9 [16.7–23.2]
Secondary/higher	21.1 [18.0–24.3]	22.0 [17.9–26.2]	16.9 [11.5–22.2]	28.6 [8.8–48.4]	26.8 [14.3–39.2]
P-value	0.091	0.726	0.054	0.506	0.365
Having NSSF membership					
No	18.8 [17.8–19.7]	20.7 [19.2–22.1]	14.5 [13.1–16.0]	22.9 [18.1–27.6]	21.9 [18.8–25.0]
Yes	17.5 [13.2–21.8]	20.4 [13.9–26.9]	14.7 [7.0–22.4]	13.8 [1.6–26.0]	11.8 [4.7–18.8]
P-value	0.5604	0.937	0.961	0.339	0.076
Having HEF membership					
No	19.4 [18.3–20.5]	21.4 [19.8–23.0]	14.9 [13.3–16.5]	24.1 [18.8–29.3]	22.6 [19.4–25.9]
Yes	15.8 [13.8–17.8]	17.6 [14.6–20.5]	13.2 [10.2–16.2]	14.3 [11.0–18.6]	14.9 [7.5–22.3]
P-value	0.004	0.035	0.360	0.096	0.058
Household socio-economic class					
Poorest	20.2 [17.7–22.8]	23.9 [20.1–27.6]	13.6 [10.3–16.9]	18.0 [6.6–29.4]	23.6 [14.8–32.4]
Poor	16.9 [14.8–18.9]	17.8 [15.0–20.6]	14.7 [11.4–18.0]	23.2 [11.3–35.1]	14.8 [6.0–23.7]
Medium	18.2 [16.2–20.2]	19.8 [16.9–22.6]	14.7 [11.4–18.0]	18.8 [12.5–25.0]	21.2 [14.4–28.1]
Rich	20.0 [17.8–22.2]	23.0 [19.4–26.5]	15.6 [12.5–18.6]	23.4 [10.3–36.6]	21.5 [16.8–26.3]
Richest	18.2 [16.2–20.2]	19.2 [16.4–22.0]	14.0 [11.0–17.0]	26.8 [15.8–37.7]	24.1 [17.4–30.8]
P-value	0.171	0.034	0.919	0.687	0.427
Sector					
Private	20.5 [19.3–21.6]	23.0 [21.4–24.7]	15.7 [14.0–17.3]	24.9 [18.3–31.6]	22.0 [18.0–26.0]
Public	12.0 [10.3–13.7]	9.8 [7.4–12.2]	10.6 [7.7–13.6]	18.5 [13.2–23.8]	20.4 [15.6–25.2]
Both	21.7 [17.4–26.1]	24.8 [19.5–30.1]	9.4 [5.7–13.1]	__	20.5 [10.6–30.4]

P-value	<0.001	<0.001	0.013	0.164	0.878
Care model					
Coexisting	16.7 [14.7–18.7]	17.9 [15.1–20.6]	13.8 [10.8–16.9]	20.0 [11.8–28.2]	23.3 [12.1–34.6]
Community-based	22.6 [20.3–25.0]	24.5 [21.1–27.9]	18.9 [15.1–22.7]	22.3 [12.7–32.0]	27.2 [19.2–35.2]
Health center-based (high)	18.6 [16.4–20.7]	20.7 [17.5–24.0]	14.8 [11.7–17.8]	19.7 [9.2–30.1]	22.8 [14.6–30.9]
Health center-based (low)	15.7 [13.9–17.5]	17.6 [14.8–20.4]	11.0 [8.8–13.2]	24.1 [13.1–35.2]	19.0 [15.1–22.9]
Hospital-based	20.3 [17.9–22.7]	22.8 [19.4–26.2]	14.8 [10.9–18.7]	23.3 [12.1–34.6]	18.1 [12.0–24.1]
P-value	<0.001	0.006	0.011	0.896	0.2695

Abbreviation CI, confidence interval

Note: Health center-based (high) means the OD with high coverage (six out of nine) of health centers with the WHO PEN; Health center-based (low) means the OD with low coverage (six out of 25) of health centers with the WHO PEN

Table S2. Geometric mean of medical cost by participant characteristics in Cambodia, 2020

Variable	**Overall**	**No T2D/HTN**	**HTN**	**T2D**	**T2D plus HTN**
	Mean [95% CI]	Mean [95% CI]	Mean [95% CI]	Mean [95% CI]	Mean [95% CI]
Sex					
Male	6.9 [6.2–7.8]	6.5 [5.6–7.5]	7.0 [5.7–8.6]	14.7 [8.8–24.7]	8.5 [4.2–17.0]
Female	8.5 [7.9–9.1]	8.9 [8.0–10.0]	6.8 [6.1–7.6]	12.5 [9.4–16.5]	13.9 [11.1–17.5]
P-value	**0.004**	**<0.001**	0.79	0.548	**0.104**
Age in years					
40–49	7.9 [6.8–9.0]	8.2 [7.0–9.7]	8.2 [7.0–9.7]	5.0 [3.7–6.8]	13.2 [8.3–20.9]
50–59	8.4 [7.6–9.4]	28.2 [23.6–33.6]	7.9 [6.7–9.2]	7.6 [6.4–8.9]	15.2 [10.6–21.9]
60+	7.7 [7.0–8.5]	28.4 [23.7–33.9]	7.9 [6.8–9.3]	6.9 [6.0–7.8]	11.1 [7.1–17.3]
P-value	0.480	0.91	**0.09**	0.509	0.384
Educational level					
No schooling	7.9 [7.0–8.8]	7.4 [6.2–8.8]	7.6 [6.4–8.9]	13.0 [8.1–20.8]	11.6 [7.4–18.2]
Primary	8.0 [7.4–8.7]	8.3 [7.4–9.4]	6.4 [5.7–7.3]	12.6 [9.2–17.3]	12.9 [9.8–17.0]
Secondary/higher	8.2 [6.8–9.9]	8.0 [6.2–10.4]	6.6 [4.7–9.4]	15.7 [7.0–35.1]	17.2 [9.2–32.2]
P-value	0.920	0.510	0.299	0.842	0.600
Having NSSF membership					
No	8.1 [7.6–8.6]	8.0 [7.3–8.8]	7.0 [6.3–7.7]	13.6 [10.6–17.4]	13.5 [10.8–16.9]
Yes	6.8 [5.0–9.2]	7.9 [5.2–12.0]	5.3 [3.0–9.2]	7.6 [1.5–37.8]	6.3 [2.2–18.1]
P-value	**0.2351**	0.937	0.234	0.261	**0.071**
Having HEF membership					
No	8.8 [8.2–9.4]	8.7 [7.9–9.7]	7.4 [6.6–8.2]	14.1 [7.4–20.9]	16.1 [13.5–19.3]
Yes	5.4 [4.6–6.3]	5.5 [4.4–6.9]	5.1 [4.0–6.7]	8.6 [4.5–16.3]	4.1 [1.7–9.9]
P-value	**<0.001**	**<0.001**	**0.004**	**0.114**	**<0.001**
Household socio-economic class					

Poorest	6.8 [5.8–8.0]	7.3 [5.8–9.2]	5.4 [4.1–6.9]	8.4 [3.9–18.3]	11.1 [5.5–22.4]
Poor	7.4 [6.5–8.5]	7.4 [6.1–8.9]	7.1 [5.7–8.8]	13.6 [7.2–25.9]	6.6 [3.2–13.7]
Medium	8.4 [7.3–9.6]	8.2 [6.7–10.0]	7.4 [6.0–9.1]	12.6 [7.9–20.1]	12.5 [7.5–20.8]
Rich	9.4 [8.2–10.8]	9.6 [7.8–11.9]	7.9 [6.5–9.6]	14.1 [6.8–28.9]	15.8 [11.1–22.5]
Richest	8.2 [7.2–9.3]	7.8 [6.5–9.4]	6.7 [5.5–8.3]	16.5 [10.5–25.7]	19.0 [14.4–25.1]
P-value	**0.022**	0.366	**0.133**	0.538	**0.054**
Sector					
Private	9.8 [9.2–10.5]	10.2 [9.2–11.2]	8.4 [7.6–9.2]	15.0 [11.2–20.2]	15.5 [12.6–19.0]
Public	3.5 [3.0–4.1]	2.5 [2.0–3.1]	3.1 [2.4–4.1]	10.7 [7.0–16.3]	9.5 [5.9–15.2]
Both	13.9 [10.8–17.9]	16.2 [12.2–21.5]	6.9 [4.0–12.1]	—	20.3 [12.7–32.4]
P-value	**<0.001**	**<0.001**	**<0.001**	**0.176**	**0.084**
Care model					
Coexisting	7.3 [6.3–8.5]	7.5 [6.2–9.2]	6.0 [4.7–7.6]	12.8 [6.9–23.5]	17.5 [10.8–28.3]
Community-based	10.8 [9.4–12.3]	10.4 [8.5–12.8]	9.7 [7.9–11.8]	16.5 [10.8–25.2]	20.5 [14.3–29.2]
Health center-based (high)	8.5 [7.5–9.7]	8.6 [7.0–10.5]	7.4 [6.1–9.0]	11.4 [7.1–18.2]	15.0 [9.5–23.9]
Health center-based (low)	6.4 [5.6–7.3]	5.9 [4.8–7.2]	5.6 [4.6–6.9]	13.7 [6.9–27.4]	13.9 [9.5–20.1]
Hospital-based	7.9 [6.7–9.2]	8.5 [6.9–10.5]	6.3 [4.8–8.1]	17.5 [10.8–28.3]	7.3 [4.2–12.8]
P-value	**<0.001**	**0.002**	**0.003**	0.911	**0.015**

Abbreviation CI, confidence interval

Note: Health center-based (high) means the OD with high coverage (six out of nine) of health centers with the WHO PEN; Health center-based (low) means the OD with low coverage (six out of 25) of health centers with the WHO PEN
