# Supplementary material for: Healthcare usage and expenditure among people with type 2 diabetes and/or hypertension in Cambodia: results from a cross-sectional survey
Source: BMJ Open. 2023 Jan 12;13(1):e061959. doi: 10.1136/bmjopen-2022-061959 (PMC9843177; doi:10.1136/bmjopen-2022-061959)
Supplement: Author's manuscript [file bmjopen-2022-061959.draft_revisions.pdf]

BMJ Open is committed to open peer review. As part of this commitment we make the peer review history of every article we publish publicly available.

When an article is published we post the peer reviewers' comments and the authors' responses online. We also post the versions of the paper that were used during peer review. These are the versions that the peer review comments apply to.

The versions of the paper that follow are the versions that were submitted during the peer review process. They are not the versions of record or the final published versions. They should not be cited or distributed as the published version of this manuscript.

BMJ Open is an open access journal and the full, final, typeset and author-corrected version of record of the manuscript is available on our site with no access controls, subscription charges or pay-per-view fees (http://bmjopen.bmj.com).

If you have any questions on BMJ Open's open peer review process please email info.bmjopen@bmj.com

BMJ Open

Healthcare utilization and expenditure among people with type 2 diabetes and/or hypertension in Cambodia: results from a cross-sectional survey

Journal:	*BMJ Open*
Manuscript ID	bmjopen-2022-061959
Article Type:	Original research
Date Submitted by the Author:	15-Feb-2022

Complete List of Authors:	Chhim, Srean; National Institute of Public Health, Technical Office<br>Te, Vannarath; National Institute of Public Health, School of Public Health<br>buffel, veerle; University of Antwerp, Sociology<br>van Olmen, Josefien; University of Antwerp, ELIZA<br>Long, Sereyraksmey; National Institute of Public Health, School of Public Health<br>Yem, Sokunthea; National Institute of Public Health, Technical Office<br>Van Damme, Wim; Institute of Tropical Medicine, Public Health; Vrije Universiteit Brussel, Gerontology<br>Wouters, Edwin; University of Antwerp, Centre for Longitudinal & Life Course Studies; University of the Free State, Centre for Health Systems Research & Development<br>Por, Ir; National Institute of Public Health

Keywords:	HEALTH SERVICES ADMINISTRATION & MANAGEMENT, HEALTH ECONOMICS, Hypertension < CARDIOLOGY

SCHOLARONE™
Manuscripts

I, the Submitting Author has the right to grant and does grant on behalf of all authors of the Work (as defined in the below author licence), an exclusive licence and/or a non-exclusive licence for contributions from authors who are: i) UK Crown employees; ii) where BMJ has agreed a CC-BY licence shall apply, and/or iii) in accordance with the terms applicable for US Federal Government officers or employees acting as part of their official duties; on a worldwide, perpetual, irrevocable, royalty-free basis to BMJ Publishing Group Ltd ("BMJ") its licensees and where the relevant Journal is co-owned by BMJ to the co-owners of the Journal, to publish the Work in this journal and any other BMJ products and to exploit all rights, as set out in our licence.

The Submitting Author accepts and understands that any supply made under these terms is made by BMJ to the Submitting Author unless you are acting as an employee on behalf of your employer or a postgraduate student of an affiliated institution which is paying any applicable article publishing charge ("APC") for Open Access articles. Where the Submitting Author wishes to make the Work available on an Open Access basis (and intends to pay the relevant APC), the terms of reuse of such Open Access shall be governed by a Creative Commons licence – details of these licences and which Creative Commons licence will apply to this Work are set out in our licence referred to above.

Other than as permitted in any relevant BMJ Author's Self Archiving Policies, I confirm this Work has not been accepted for publication elsewhere, is not being considered for publication elsewhere and does not duplicate material already published. I confirm all authors consent to publication of this Work and authorise the granting of this licence.

For peer review only

Healthcare utilization and expenditure among people with type 2 diabetes and/or hypertension in Cambodia: results from a cross-sectional survey

Srean Chhim[1,2*], Vannarath Te[2,3,4], Veerle Buffel[5], Josefien Van Olmen[4], Savina Chham[1,5], Sereyraksmey Long[2], Sokunthea Yem[1], Wim Van Damme[3], Edwin Wouters[5,6], Por Ir[1,2]

Affiliations

[1] Technical Bureau, National Institute of Public Health, Phnom Penh, Cambodia

[2] School of Public Health, National Institute of Public Health, Phnom Penh, Cambodia

[3] Health Policy Unit, Department of Public Health, Institute of Tropical Medicine, Antwerp, Belgium

[4] Department of Family Medicine and Population Health (FAMPOP), Faculty of Medicine and Health Sciences, University of Antwerp, Belgium

[5] Centre for Population, Family, and Health, Department of Social Sciences, University of Antwerp, Belgium

[6] Centre for Health Systems Research and Development, University of the Free State, South Africa

* ***Corresponding author:*** Srean Chhim (#109, street 01, Krang Thnong, Saensokh, Phnom Penh, postal 120804), srean168@gmail.com

Keywords: healthcare, utilization, expenditure, Cambodia

Word count 5082/4000

ABSTRACT

Objective: To assess utilization of public and private healthcare, related healthcare expenditure, and associated factors for people with type 2 diabetes (T2D) and/or hypertension (HTN) and for people without those conditions in Cambodia.

Methods: A cross-sectional household survey.

Settings: Five operational districts in Cambodia.

Participants: Data was from 2,360 participants aged ≥ 40 years who had used healthcare services at least once in the three months preceding the survey.

Primary and secondary outcome: The main variables of interest were the number of healthcare visits and healthcare expenditure in the last three months.

Results: The majority of healthcare visits took place in the private sector. Only 22.0% of healthcare visits took place in public healthcare facilities: 21.7% in people with HTN, 37.2% in people with T2D, 34.7% in people with T2D plus HTN, and 18.9% in people without the two conditions (*P*-value<0.01). In people with T2D and/or HTN, increased public healthcare use was significantly associated with Health Equity Fund (HEF) membership and living in operational districts with *community-based care*. Furthermore, significant healthcare expenditure reduction was associated with HEF membership and using public healthcare facilities in these populations.

Conclusion: Overall public healthcare utilization was relatively low; however, it was higher in people with chronic conditions. HEF membership and *community-based care* contributed to higher public healthcare utilization among people with chronic conditions. Using public healthcare services regardless of HEF status reduced healthcare expenditure, but the reduction in spending was more noticeable in people with HEF membership. To increase public healthcare utilization among people with T2D and/or HTN, the public healthcare system should further

For peer review only

improve care quality, expand social health protection, and expand *community-based care* for this population. However, these may require more time and resources. One potential strategy in the short run is to partner the private sector with the public sector.

For peer review only

STRENGTHS AND LIMITATIONS OF THIS STUDY

- Our study is among the few to examine healthcare utilization and expenditure among people with two major chronic conditions type 2 diabetes (T2D) and/or hypertension (HTN) in Cambodia, in comparison with the people without the two conditions.
- The findings in this study provides could inform decision-makers about strategies for T2D and HTN management in Cambodia and similar settings.
- Our study is not representative for the national level as most of the study sites we selected were rural or semi-rural.
- We only calculated the healthcare expenditure for those who used the service in the three months preceding the study, which cannot be generalized to the target population in the study (people aged 40 years or older).

For peer review only

INTRODUCTION

Type 2 diabetes (T2D) and hypertension (HTN) are global public health concerns. They are major risk factors for cardiovascular diseases, causing about 31% (17.9 million) of all deaths worldwide annually.[1] The prevalence of people with T2D and/or HTN will likely continue to increase.[2 3] These two diseases disproportionately affect low- and middle-income countries and account for around 75% of all deaths in these countries.[1]

In Cambodia, large-scale population-based studies such as STEPS Surveys have shed light on the prevalence and risk factors of chronic diseases. The prevalence of T2D and HTN rose noticeably over recent years from 2.9% and 11.2% in the population aged 25–64 years in 2010 to 9.6% and 14.2% in the population aged 18–69 years in 2016, respectively.[4]

Previous nationally representative surveys have shown that a majority of the population seeks outpatient curative care in private facilities, but knowledge on the related healthcare uptake and expenditure among those with T2D and/or HTN is scarce.[5] Additionally, these surveys do not cover topics related to the management of the diseases, such as healthcare utilization and expenditure.[6 7] There is only one study by Bigdeli *et al.* which examines access to care for people with T2D and/or HTN concerning social health protection schemes in Cambodia.[8] This study shows that 61% of the people with T2D and/or HTN who knew their status were diagnosed in private facilities.[8] However, this study collected data in 2013, before key interventions were introduced or expanded in public healthcare facilities.[9] Also, it provides limited information about which types of health facilities were used, and what differences exist between people with one or both conditions compared to those without.

In the last decade, the Cambodian Ministry of Health (MoH), in collaboration with development partners, made significant efforts to improve the quality of public healthcare and initiated a few

For peer review only - http://bmjopen.bmj.com/site/about/guidelines.xhtml

84 healthcare delivery models for people with T2D and/or HTN in public facilities.[9] These models include *hospital-based care, health center-based care, community-based care* and a combination of all three models (*coexisting care*) (Box 1). These efforts might have changed the pattern of healthcare utilization and related expenditure, especially among people with T2D and/or HTN.

Box 1. Overview of different care models in Cambodia in 2021

- The *hospital-based care* model is standard care, which means an operational district (OD) has a government-run Non-communicable Disease (NCD) clinic at the district referral hospitals.[9] By 2021, 31 out of 117 referral hospitals had implemented this model.
- The *health center-based care* model adopts the World Health Organization Package of Essential Non-communicable Disease Interventions (WHO PEN).[9] In this model, the MoH added the function of a health center to *hospital-based care*. However, the coverage of health centers with the WHO PEN varies in each OD, which can be divided into low coverage (<50% of all health centers implement the WHO PEN; *health center-based care (low)*) and high coverage (≥50% of all health centers implement the WHO PEN; *health center-based care (high)*). The referral flow is slightly different between T2D and HTN. For T2D, this model identifies cases in the health centers through a screening test. If the patients are suspected of having T2D, health centers refer them to a diabetes clinic at a district referral hospital for confirmation of diagnosis. Once diagnosed, severe cases are treated in the hospital clinic, and stable or mild cases are followed up regularly at the health centers. For HTN, the health centers treat mild patients and refer the severe cases to the referral hospital. By 2021, *health center-based care* was implemented in 137 of 1,221 health centers.

For peer review only

- The *community-based care* model or peer education network established and run by MoPoTsyo, a local non-governmental organization.[9] In this model, peer educators (PEs) are added to hospital-based care. MoPoTsyo trained people with T2D and/or HTN to be PEs. These PEs play a role in screening and referring those suspected of having T2D and/or HTN to seek medical consultation and treatment at the referral hospitals that MoPoTsyo has partnered with. The PEs also provide counseling on lifestyle changes and support self-management to registered network members. By 2019, this *community-based care* model had been implemented in 20 of 102 ODs in 8 of 25 provinces in Cambodia.[9 10] It had 225 PEs to serve 40,000 people with T2D.[10]
- The *coexisting care* model comprises a combination of the above three models. At the time of the study until 2021, only one OD (Daunkeo) had this model.

A better understanding of the current patterns of healthcare utilization and expenditure among people with T2D and/or HTN is critical for better targeting resources and strategies to improve the management of T2D and HTN. The main objectives of this study are two-fold. First, it evaluates usage and determines the factors associated with public healthcare use in four groups: (1) people without T2D or HTN, (2) people with T2D alone, (3) people with HTN alone, and (4) people with T2D plus HTN. Second, it assesses the healthcare expenditure in the three months preceding the survey for all services used by the four patient groups in public and private facilities and determines factors associated with (reducing or increasing) healthcare expenditure.

METHODS AND CONTEXT

Context

The health system in Cambodia is pluralistic, meaning healthcare services are provided by both public and private healthcare providers.[5 11]

Public healthcare services in Cambodia dominate preventive services (reproductive, maternal, neonatal, and child health), control of primary disease (tuberculosis, malaria, and HIV/AIDS control), and inpatient treatment.[5] The facilities include health posts, health centers, district referral hospitals, provincial referral hospitals, and national hospitals.[5] Public healthcare is organized per operational district (OD)—the third and last administrative level in Cambodia's health system management.[5] An OD covers a population of 100,000–200,000 people while a health center covers a population of 10,000–20,000 people.[5] Remote areas with a small population can be covered by a health post.[5] The health post provides similar services to a health center, but it is smaller than a health center.[5] Each OD usually has one district referral hospital with a few ODs having two district hospitals.[5] The district referral hospital receives self-referred patients or those referred by the health centers.

Alongside this public sector, a large *private healthcare sector,* which is more accessible than the public sector, dominates outpatient curative care.[5] Since 1994, the Cambodian government started economic liberalization, permitting staff to work outside their government's working hours and own healthcare facilities.[5] Since then, the private healthcare sector and dual practice, meaning public healthcare workers also have private practices, have grown rapidly. In 2015, over 50% of the healthcare workforce in private healthcare facilities were government personnel.[5] The private healthcare facilities range from cabinets, laboratories, pharmacies, clinics, and polyclinics to hospitals.[5] Cabinets are the smallest facilities with less than two beds and mainly provide medical

For peer review only

consultation services.[12 13] According to the MoH Progress Report in 2018, over 90% of private healthcare facilities were cabinets.[12 13] The second most frequent facilities were clinics (3.2%), providing medical specialties, laboratories, radiology services, and pharmacies.[12] A clinic has between 10 and 20 beds.[12] In addition, buying medication in pharmacies or drugstores for self-treatment without a doctor's prescription is common in Cambodia, although not permitted by law.[14]

In terms of health expenditure, the public healthcare sector did not charge user fees until 1996.[5] In that year, the government also introduced a user-fee scheme for the public sector with fees approved by the local community to increase healthcare quality at public healthcare facilities.[5 15] The revenue from the user-fee scheme could be used to incentivize staff and support ongoing operations. However, the user-fee posed challenges for the poor to access public healthcare. To address this, the MoH established the Health Equity Fund (HEF) in 2000, a pro-poor social health protection scheme.[16] The HEF is linked to the implementation of identification of the poor (known as "IDPoor").[17] It is intended for the "extremely poor" or "poor" category, which is assessed and verified by the local authorities.[17] People with IDPoor are entitled to the HEF, meaning that they receive free healthcare services at public healthcare facilities and transportation expenditure reimbursement.[17] By 2019, the HEF covered approximately three million or about 20% of Cambodia's population.[16] Another scheme was the National Social Security Fund (NSSF), established in 2007.[18] The NSSF covers work and non-work-related illnesses and injuries for formally employed people.[18] Formal employers are mandated to pay for their staff's NSSF membership. The NSSF had enrolled over 1.7 million employees or about 11% of the population by 2019.[19]

For peer review only

However, it is important to note that several studies have indicated that the private sector constitutes a significant source for out-of-pocket expenditure (OOPE).[20 21] Between 2009 and 2016, around 60% of health expenditure was OOPE while the rest was a combination of the government's and development partners' budgets. The OOPE per capita increased slightly from USD 40.6 in 2009 to USD 48.1 in 2016.[21] In 2016, 76.6% of the total OOPE was linked to private healthcare.[20 21]

Data sources

This study is part of a larger cross-sectional household survey, with the primary aim of developing a care cascade for T2D and HTN.

Settings

The study purposively selected five ODs. The selection was made to include different T2D and/or HTN care models piloted in Cambodia: *coexisting care*, *community-based care*, *health-center based care (high)*, *health-center based care (low),* and *hospital-based care* (Box 1). The five ODs in which the study took place are out of 103 ODs in the country and located in five different provinces. The map of ODs is presented in Annexure 1. These ODs have similar road infrastructure improvements, in which poor road conditions are no longer a barrier to accessing healthcare.

- *OD Daunkeo, Takeo province*: This OD had the *"coexisting care"* model. At the time of the study, it was the only OD in which the three care models coexisted. The catchment area included Takeo town and a large rural area. Its NCD clinic was established in 2002, and the peer educator network was initiated in 2007 and handed over to the MoH in

For peer review only

2015.[22] The WHO PEN was implemented in 5 out of 14 health centers since 2015. The private services for people with T2D and/or HTN may also be easily accessible.

- *OD Kong Pisey, Kampong Speu province*: This OD had the *"community-based care"* model. It has a strong MoPoTsyo network to provide T2D and HTN care to patients. Located about 54 kilometers from the capital of Phnom Penh, this OD is semi-urban with a variety of private facilities.
- *OD Pearaing, Prey Veng province*: This OD had the *"health center-based (high)"* model, and was the OD with high coverage of the WHO PEN. Six out of nine health centers in this OD have been piloting the WHO PEN since 2015. Due to dual practice, the high coverage of the WHO PEN also facilitates accessible private services for people with T2D and/or HTN.
- *OD Sot Nikum, Siem Reap province*: This OD had the *"health center-based (low*)" model, and was the OD with low coverage of the WHO PEN (6/25 of the health centers started the WHO PEN in 2018). This OD has been historically and significantly influenced by the financial support of various development partners, and services for people with T2D and/or HTN have been well arranged at its NCD clinic.[23]
- *OD Samrong, Oddar Meanchey province*: This OD had a "*hospital-based care"* model. It had an NCD clinic without the WHO PEN and peer educator network. A large part of the catchment is a remote area bordering Thailand, approximately 470 kilometers from the capital. Therefore, the private services for people with T2D and/or HTN may not be broadly accessible.

For peer review only

Samples

The larger household survey recruited 5,072 individuals aged 40 years or older to participate in the study using a multi-stage cluster sampling method. Initially, it purposively chose five ODs with different care models for T2D and HTN. Second, 44 villages per OD were randomly selected, regardless of the population size of each OD. The purpose of this equal probability selection was to over-sample participants in ODs with a smaller population so that they would have an adequate sample for each care model. Third, 24 households in each village were selected by probability systematic sampling, and finally, one person aged 40 years or older per household was selected at random. To minimize the non-response rate, which can unintentionally exclude a certain group of the target population from the survey, the selected participants were called back or followed-up three times when they were absent from their household. If the attempt failed, another household in the next row in the sampling list was selected. Then, the procedure described above was repeated. The equal probability selection at the village and household levels were used with the OD level's same purpose.

To correspond to our analytical objective, we used a subset of this sample: we only retained those who reported using healthcare services at least once in the three months preceding the survey (Figure 1). A total of 2,360/5,072 participants met this criterion. The 2,360-participant sample subset included four patient groups: 1,331 people without T2D and HTN, 761 people with HTN alone, 109 people with T2D alone, and 159 people with T2D plus HTN.

For peer review only

Figure 1. Data flow from household selection to final dataset in this study, Cambodia, 2020

Data collection

The data collection took place between July and October 2020. The data collection was conducted in three steps following the WHO's STEPS Survey approach: (1) interviews with a structured questionnaire, (2) anthropometric measurements, and (3) biochemical measurements.[6, 7] Since our study only focuses on healthcare utilization and expenditure, we only used information from step 1—interviews with a structured questionnaire. The questionnaire was tablet-based and comprised of 11 sections, including socio-demographic information, health status and quality of life, healthcare utilization, social support, lifestyle or behavior measures, physical activity, diabetes and hypertension knowledge, medication adherence, self-management support, and decision-making power on food. However, we only used two sections in our analysis: socio-demographic information and healthcare utilization.

The tablet-based questionnaire was installed using the Kobo Toolbox (https://kf.kobotoolbox.org), an open-source software with a free-of-charge server and online storage.[24]

Measures

This study's primary variable of interest is the number of visits to public and private facilities. By public healthcare facilities, we refer to government-run facilities that provide medical services, and include national hospitals, provincial referral hospitals, district referral hospitals, health centers, and health posts. A health post is similar to a health center, and only a few exist in remote areas. Therefore, we grouped them with health centers. Private healthcare services are non-government organizations that provide medical and non-medical services, and include

private hospitals, private clinics, pharmacies, homes of trained health workers, and visits of health workers to the patients' homes. Traditional healing/medicine and using healthcare services abroad have also been included in this category.

The secondary variable of interest was healthcare expenditure, the lump sum expenditure of medical consultation, treatment, and medication. These data were obtained from the interview with the participants. They were asked about their use of health services in the three months preceding the survey (where they went, how often they went to a particular type of healthcare facility, and how much they spent in each facility in those three months). We include the questionnaire in Annexure 2. The Cambodian currency (riels) was converted into USD at an exchange rate of 4,000 riels per USD. The expenditure does not include other spendings such as on transport, food, or guesthouses/hotels.

To better understand the profile of people using public or private healthcare facilities, we estimated associations between the use of public and private healthcare services and patient characteristics such as sex (male, female), age (40–49, 50–59, 60+ years old), educational level (none, primary, secondary or higher), social protection status (NSSF (yes, no), HEF (yes, no)), wealth quintile (poorest, poor, medium, rich, richest), type of care models (*hospital-based*, *health center-based (high)*, *health center-based (low)*, and *community-based*). The details on wealth quintile calculation (socio-economic class) are described in Annexure 3.

Analysis

Healthcare utilization

For peer review only

Taking the number of visits as a dependent variable, we report the healthcare visit rate to public and private facilities over the three months preceding the survey, then stratified by patient groups.

To identify the independent factors associated with healthcare utilization (defined by the number of visits), we first used bivariate negative binomial regression to identify the potential factors in the five groups—overall and four patient groups—separately. Variables with a P-value<0.25 in at least one of the four patient groups or overall group were included in the multiple negative binomial regression. The exposure variable (total healthcare visits of each participant) was incorporated into this model. Variables with a P-value<0.05 were considered statistically significant in this final model. The negative binomial regression was chosen over Poisson regression because the number of visits was over-dispersed.

Medical expenditure

We took healthcare expenditure in the three months preceding the survey as the dependent variable. Due to the limitation of our data, we focused more on assessing the factors associated with healthcare expenditure and did not explore the overall medical expenditure. We reported the overall arithmetic mean and then stratified the mean by patient groups. The expenditure was calculated separately for each patient group. Because arithmetic means can be easily affected by extremely high values, we removed the values above the 90th percentile, which we believe were too high in our sample.

Our analysis was carried out in three steps to separately identify the independent factors associated with healthcare expenditure in the four patient groups. First, a logarithmic transformation of the medical expenditure was performed as the data was skewed to the right.

For peer review only

Second, in the bivariate analysis, we compared the geometric mean of healthcare expenditure by characteristics of the participants. This analysis identified the variables potentially associated with the healthcare expenditure at a *P*-value<0.25. During this phase, the Student's t-test for binary explanatory variables and the one-way ANOVA test for non-binary explanatory variables were used. Variables with a significant level at a *P*-value<0.25 in any patient group were included in the multiple linear regression. Third, multiple linear regression was performed and the coefficient and 95% confidence interval (CI) values were exponentiated to a risk ratio (RR) for better interpretation.

Data were analyzed using Stata 16.0 (Stata Corp LLC, College Station, Texas 77, USA), and R programing's GGPLOT2 package was used to produce the graphs.

Ethical approval

The protocol was approved by the National Ethics Committee for Human Research (NECHR) on April 29, 2019 (No. 105 NECHR) and by the Institutional Review Board of Institute of Tropical Medicine (Antwerp) on October 25, 2019 (No. 1,323/19). All participants provided their consent and volunteered to take part in the study.

Patient and public involvement

No patient was involved in the development of the research question and outcome measures, study design, and study participant recruitment. The findings are not disseminated to the study participants.

For peer review only

RESULTS

Characteristics of participants

Our analysis included 2,360 participants, including 1,331 people without T2D or HTN, 761 people with HTN alone, 109 with T2D alone, and 159 with T2D plus HTN. The other participants were excluded because they had not used healthcare services in the three months preceding the survey (N=2,703) or had a missing response to the primary variable of interest (N=9).

Females were more prevalent in all patient groups, especially in the T2D plus HTN group. The age range was between 40 and 96 years, with people with HTN and T2D plus HTN having a significantly higher average age than those without the two conditions. The majority of participants did not attend school or attended only primary school.

Regarding the social health protection scheme, a small proportion of participants in all groups had the NSSF membership (4.8% overall). A larger proportion of patients across all groups had the HEF membership (18.4% overall).

Table 1. Demographic and socio-economic characteristics of participant, 2020, Cambodia

Variable	Overall (N=2360)	No T2D/HTN (N=1331)	HTN (N=761)	T2D (N=109)	T2D plus HTN (N=159)	*P*-value
		n (%)	n (%)	n (%)	n (%)	
Sex of participant						
Male	689 (29.2)	457 (34.3)	179 (23.5)	29 (26.6)	24 (15.1)	<0.001
Female	1671 (70.8)	874 (65.7)	582 (76.5)	80 (73.4)	135 (84.9)	
Age in years						
Range	40–96	40–96	40–90	40–81	40–82	
Mean (±SD)	58.5 (± 10.4)	56.0 (±10.3)	62.4 (±10.0)	57.6 (±8.4)	61.7 (±8.2)	<0.001
40–49	497 (21.1)	398 (29.9)	75 (9.9)	17 (15.6)	7 (4.4)	<0.001
50–59	803 (34.0)	464 (34.9)	231 (30.4)	46 (42.2)	62 (39.0)	
60 or older	1060 (44.9)	469 (35.2)	455 (59.8)	46 (42.2)	90 (56.6)	
Educational level						
No formal schooling	757 (32.1)	393 (29.5)	283 (37.2)	35 (32.1)	46 (28.9)	<0.016
Primary school	1308 (55.4)	755 (56.7)	398 (52.3)	61 (56.0)	94 (59.1)	
Secondary school or higher	295 (12.5)	183 (13.7)	80 (10.5)	13 (11.9)	19 (11.9)	

For peer review only

Having NSSF membership (yes)	114 (4.8)	62 (4.7)	36 (4.7)	6 (5.5)	10 (6.3)	0.806
Having HEF membership (yes)	434 (18.4)	247 (18.6)	143 (18.8)	18 (16.5)	26 (16.4)	0.849
Wealth quintile						
Poorest	441 (18.7)	261 (19.6)	140 (18.4)	16 (14.7)	24 (15.1)	0.050
Poor	447 (18.9)	263 (19.8)	139 (18.3)	18 (16.5)	27 (17.0)	
Medium	467 (19.8)	262 (19.7)	144 (18.9)	27 (24.8)	34 (21.4)	
Rich	480 (20.3)	244 (18.3)	176 (23.1)	16 (14.7)	44 (27.7)	
Richest	525 (22.2)	301 (22.6)	162 (21.3)	32 (29.4)	30 (18.9)	
Care model						
Coexisting	432 (18.3)	248 (18.6)	147 (19.3)	20 (18.3)	17 (10.7)	0.015
Community-based	480 (20.3)	276 (20.7)	153 (20.1)	18 (16.5)	33 (20.8)	
Health center-based (high)	486 (20.6)	257 (19.3)	174 (22.9)	27 (24.8)	28 (17.6)	
Health center-based (low)	518 (22.0)	292 (21.9)	170 (22.3)	18 (16.5)	38 (23.9)	
Hospital-based	444 (18.8)	258 (19.4)	117 (15.4)	26 (23.9)	43 (27.0)	

Abbreviations: SD, standard deviation; NSSF, National Social Security Fund; HEF, Health Equity Fund

Note: Health center-based (high) means the OD with high coverage (six out of nine) of health centers with the WHO PEN; Health center-based (low) means the OD with low coverage (six out of 25) of health centers with the WHO PEN

Public and private healthcare utilization

The 2,360 individuals reported 6,645 visits to the healthcare facilities in the three months preceding the survey, averaging 2.8 visits per person over three months.

Figure 2 presents the proportion of visits to public and private healthcare facilities. At the facility level, as shown in Figure 2A, the largest share was accounted for by private clinics (28.5%), followed by visits to the private homes of nurses or doctors (15.6%), private pharmacies (15.3%), health centers (12.6%), and private hospitals (11.6%).

The common public healthcare facilities used by participants with T2D and T2D plus HTN were provincial/national and district referral hospitals (Figure 2A). Approximately 29.7% of visits from people with T2D and 29.6% from people with T2D plus HTN went to provincial/national and district hospitals (Figure 2A). These proportions were higher than 6.9% for people without T2D or HTN and 6.1% for HTN only.

Overall, the private sector occupied about 78.0% of the total visits, and the public sector occupied 22.0% (Figure 2B). All groups visited private healthcare facilities more frequently than public healthcare facilities (Figure 2). However, the frequency of visiting public facilities was

For peer review only

statistically higher in people with T2D and T2D plus HTN. As shown in Figure 2B, 37.2% of visits from people with T2D and 34.7% of visits from people with T2D plus HTN were to public healthcare facilities, compared with 18.9% of visits from people without the two conditions and 21.7% of the visits from people with HTN (*P*-value<0.001).

Figure 2. Proportion of visits to public and private facilities in Cambodia, 2020

Public healthcare utilization by participant characteristics

Table 2 compares the public healthcare facility user rates defined as the proportion of public healthcare visits over total visits (public visits plus private visits). The user rates were disaggregated by participant characteristics. In this bivariate analysis, age, NSSF, HEF, wealth quintile, and care model were significantly associated with public healthcare utilization at a *P*-value<0.25 in at least one patient group. Therefore, we included these variables in the multiple negative binomial regressions.

Table 2. Proportions of visits to public facilities by participant characteristics, 2020, Cambodia

Variable	**Overall (N=6645)**	**No T2D/HTN (N=3467)**	**HTN (N=2345)**	**T2D (N=320)**	**T2D plus HTN (N=513)**
	**User rate (%)**	**User rate (%)**	**User rate (%)**	**User rate (%)**	**User rate (%)**
Sex					
Male	20.9	17.9	21.9	47.2	26.4
Female	23.7	20.7	23.2	33.3	37.6
P-value	0.936	0.803	0.879	0.552	0.847
Age in years					
40–49	17.5	14.8	27.4	23.8	32.1
50–59	24.3	22.4	19.2	35	51.4
60+	24.0	21.1	24.2	43.5	26.3
P-value	0.442	**0.096**	0.601	0.708	0.254
Educational level					
No schooling	21.9	20.1	19.3	37.5	39.6
Primary	22.9	18.8	23.2	45	34.6
Secondary/higher	25.5	22.6	33.1	—	29.5

For peer review only

P-value	0.998	0.981	0.606	0.92	0.932
Having NSSF membership					
No	22.2	19.9	21.2	37.5	33.3
Yes	35.9	17.1	52.4	31.6	66.7
P-value	0.512	0.4505	**0.165**	0.808	0.343
Having HEF membership					
No	19.8	18	18.3	34.2	29.5
Yes	35.7	27.5	39.5	55.6	65.1
P-value	**<0.001**	**0.01**	**0.014**	0.346	**0.04**
Household socio-economic class					
Poorest	28.2	20.4	33.5	54.5	58
Poor	24.9	23.5	23.3	27.3	42.7
Medium	22.3	15.2	25.7	36.6	37.5
Rich	22.1	20.5	19.9	50	30
Richest	18.1	18.9	14.8	29.9	16.5
P-value	0.029	0.419	**0.083**	0.966	**0.218**
Care model					
Coexisting	31.1	25.8	36.6	53.0	20.0
Community-based	19.5	18.0	13.6	56.8	39.2
Health center-based (high)	14.7	13.2	14.8	19.0	23.8
Health center-based (low)	21.4	21.5	16.6	46.7	32.7
Hospital-based	24.4	16.3	29.7	22.5	42.9
P-value	**0.002**	**0.007**	**0.018**	**0.172**	**0.162**

Abbreviation: NSSF, National Social Security Fund; HEF, Health Equity Fund

Note:

- *The % of private healthcare is not presented in this table but can it be calculated by subtracting the % of the public healthcare from 100%.*
- *Health center-based (high) means the OD with high coverage (six out of nine) of health centers with the WHO PEN; Health center-based (low) means the OD with low coverage (six out of 25) of health centers with the WHO PEN*
- *"N" denotes the total of visits.*
- *P-values<0.25 are in bold, indicating a significant level at 0.25. Variables with P-value<0.25 were included in multivariate analysis.*

Table 3 presents the results of the multiple negative binomial regressions. Overall, the significant increase in public healthcare use was associated with having T2D and T2D plus HTN, living in the OD with *coexisting care,* and HEF membership.

In people without T2D or HTN, HEF membership was significantly associated with public healthcare use: adjusted incidence rate ratio (AIRR) of 1.4 [95% CI 1.0–2.0], *P*-value<0.05). We did not observe the same association in the other three groups.

For peer review only

In people with HTN, the poorest category was significantly associated with increasing public healthcare use with an AIRR of 2.1 [95% CI 1.1–4.0], *P*-value=0.02), compared to those in the richest category. Nevertheless, the same association was not seen in other patient groups.

Regarding the care model, in people with T2D, the OD with *community-based care* (AIRR 3.7 [95% CI 1.2–11.3], *P*-value=0.019) and the OD with low coverage of *health center-based care* (AIRR 3.3 [95% CI 1.1–9.8], *P*-value=0.036) were significantly higher in public healthcare use than in the OD with *hospital-based care*.

In people with T2D plus HTN, the OD with *coexisting care* was associated with higher public healthcare use (AIRR 4.0 [95% CI 1.2–12.9], *P*-value=0.020).

Table 3. Factors associated with public healthcare use, 2020, Cambodia

	**Overall (N=2360)**	**No T2D/HTN (N=1331)**	**HTN (N=161)**	**T2D (N=109)**	**T2D plus HTN (N=759)**
Disease group	AIRR [95% CI]	AIRR [95% CI]	AIRR [95% CI]	AIRR [95% CI]	AIRR [95% CI]
No T2D/HTN	Ref.				
HTN	1.0 [0.8–1.2]	—	—	—	—
T2D	**1.9 [1.3–2.9]****	—	—	—	—
T2D plus HTN	**1.9 [1.3–2.7]*****	—	—	—	—
Age in year					
40–49	Ref.	Ref.	Ref.	Ref.	
50–59	1.2 [0.9–1.5]	1.4 [1.0–1.9]	0.6 [0.3–1.2]	1.5 [0.5–4.5]	1.2 [0.3–4.2]
60+	1.1 [0.8–1.4]	1.2 [0.9–1.7]	0.7 [0.4–1.3]	1.5 [0.5–4.5]	0.6 [0.2–2.2]
Having NSSF membership					
No	Ref.	Ref.	Ref.	Ref.	
Yes	1.4 [0.9–2.1]	1.0 [0.5–1.8]	2.0 [0.9–4.6]	2.3 [0.5–10.1]	1.9 [0.7–4.8]
Having HEF membership					
No		Ref.	Ref.	Ref.	
Yes	**1.4 [1.1–1.8]***	**1.4 [1.0–2.0]***	1.4 [0.9–2.3]	2.1 [0.8–5.1]	**1.9 [1.0–3.7]**
Household socio-economic class					
Poorest	**1.4 [1.0–2.0]***	1.0 [0.7–1.6]	**2.1 [1.1–4.0]***	1.2 [0.4–3.5]	2.6 [0.9–7.1]
Poor	1.2 [0.9–1.7]	1.1 [0.7–1.7]	1.2 [0.7–2.3]	0.7 [0.2–2.1]	2.6 [1.0–7.3]
Medium	1.1 [0.8–1.5]	0.9 [0.6–1.4]	1.2 [0.7–2.3]	0.9 [0.4–2.2]	3.0 [1.2–7.7]
Rich	1.1 [0.8–1.4]	1.1 [0.7–1.7]	1.0 [0.6–1.8]	1.2 [0.4–3.5]	1.7 [0.7–4.1]
Richest	Ref.	Ref.	Ref.	Ref.	Ref.
Care model					
Coexisting	**1.4 [1.0–1.9]***	1.5 [1.0–2.3]	1.3 [0.7–2.3]	2.5 [0.8–7.6]	**4.0 [1.2–12.9]***
Community-based	0.9 [0.7–1.2]	1.0 [0.6–1.5]	0.5 [0.3–1.0]	**3.7 [1.2–11.3]***	1.7 [0.5–6.1]

For peer review only

Health center-based (high)	0.8 [0.6–1.1]	0.8 [0.5–1.3]	0.7 [0.4–1.3]	1.3 [0.4–3.9]	2.7 [0.9–8.8]
Health center-based (low)	1.0 [0.8–1.4]	1.4 [1.0–2.1]	0.6 [0.3–1.0]	**3.3 [1.1–9.8]***	3.0 [1.0–9.1]
Hospital-based	Ref.	Ref.	Ref.	Ref.	Ref.

(*) P-value<0.05, (**) P-value<0.01, (***) P-value<0.001

Abbreviation: AIRR, adjusted incidence rate ratio; CI, confidence interval; Ref., reference group; OD, operational district

Note: Health center-based (high) means the OD with high coverage (six out of nine) of health centers with the WHO PEN; Health center-based (low) means the OD with low coverage (six out of 25) of health centers with the WHO PEN

Healthcare expenditure

Medical cost per year, overall and by facility

Overall, those who used healthcare spent an average of USD 25.3 [95% CI 22.9–27.6] for all healthcare services in the three months preceding the survey (Figure 3).

When comparing patient groups, people with T2D plus HTN had the highest healthcare expenditure with an average of USD 43.6 [95% CI 29.7–57.2], followed by people with T2D with an average of USD 34.0 [95% CI 25.5–42.6]. These expenditures were statistically higher than the average of USD 17.1 [95% CI 13.1–21.1] in people with HTN and the average of USD 26.9 [95% CI 23.9–29.9] in people without the two conditions with a *P*-value<0.001.

Figure 3. Healthcare expenditure by patient groups in the three months preceding the survey in 2020, Cambodia

Table S1 shows the arithmetic mean of healthcare expenditure (Annexure 4). The arithmetic mean is the mean before the data log-transformation. Since our model's Risk Ratio (RR) in Table 4is the geometric mean (after log-transformation) ratio, we presented the geometric mean in Table S2 (Annexure 4). In the bivariate analysis, sex, age, NSSF, HEF, wealth quintile, sector

For peer review only

(public vs. private), and care model were statistically associated with healthcare expenditure in one or more patient groups with a *P*-value<0.25. These variables were included in the multiple linear regression.

Table 5presents results from multiple linear regression. Overall, having T2D or T2D plus HTN, being female, having reported using both private and public healthcare, and living in the OD with *community-based care* was significantly associated with increased healthcare expenditure. In contrast, holding HEF membership and using public healthcare was significantly associated with healthcare expenditure reduction.

At the group level, in people without the two conditions, HEF membership was significantly associated with a reduction in healthcare expenditure with an adjusted risk ratio (ARR) of 0.7 [95% CI 0.5–0.8], *P*-value<0.001). The same association was seen in people with HTN (ARR of 0.8 [95% CI 0.6- 1.0], *P*-value<0.01), and in T2D plus HTN (ARR of 0.3 [95% CI 0.2–0.6], *P*-value<0.001). However, the association was not observed in people with T2D.

In people without the two conditions, using public healthcare was significantly associated with a reduction in the expenditure (ARR of 0.3 [95% CI 0.2–0.3], *P*-value<0.001). The association was also found in people with HTN (ARR 0.4 [95% CI 0.3–0.5], *P*-value<0.001).

People with T2D plus HTN who resided in the OD with *community-based care* were significantly associated with a higher expenditure with an ARR of 2.0 [95% CI 1.1–3.8], *P*-value<0.01) than those with *hospital-based care*.

Table 4 Factors associated with reducing or increasing healthcare expenditure in 2020, Cambodia

Variable	**Overall [N=2142]**	**No T2D/HTN [N=1187]**	**HTN [N=726]**	**T2D [N=98]**	**T2D plus HTN [N=139]**
	ARR [95% CI]	ARR [95% CI]	ARR [95% CI]	ARR [95% CI]	ARR [95% CI]

For peer review only

Disease group					
No T2D/HTN	Ref.	—	—	—	—
HTN	0.9 [0.7–1.0]	—	—	—	—
T2D	2.1 [1.6–2.7]***	—	—	—	—
T2D plus HTN	1.9 [1.5–2.4]***	—	—	—	—
Sex					
Male		Ref.	Ref.	Ref.	Ref.
Female	1.2 [1.1–1.4]**	1.4 [1.2–1.7]***	1.0 [0.8–1.2]	0.7 [0.4–1.5]	1.5 [0.8–2.9]
Age in years					
40–49	Ref.	Ref.	Ref.	Ref.	
50–59	1.1 [0.9–1.3]	1.0 [0.8–1.2]	1.4 [1.0–2.0]	1.1 [0.5–2.4]	1.4 [0.5–3.7]
60+	1.0 [0.9–1.2]	1.1 [0.8–1.3]	1.3 [1.0–1.9]	0.9 [0.4–1.9]	1.0 [0.4–2.7]
Educational level					
No schooling	Ref.	Ref.	Ref.	Ref.	Ref.
Primary	1.0 [0.9–1.1]	1.1 [0.9–1.4]	0.8 [0.7–1.0]	0.9 [0.5–1.7]	1.1 [0.7–1.8]
Secondary/higher	1.1 [0.8–1.3]	1.2 [0.9–1.6]	0.9 [0.6–1.3]	—	1.6 [0.7–3.7]
Having NSSF membership					
No	Ref.	Ref.	Ref.	Ref.	Ref.
Yes	0.9 [0.6–1.2]	0.9 [0.6–1.4]	0.9 [0.6–1.4]	0.6 [0.2–2.0]	0.4 [0.2–0.9]*
Having HEF membership					
No	Ref.	Ref.	Ref.	Ref.	Ref.
Yes	0.7 [0.6–0.8]***	0.7 [0.5–0.8]***	0.8 [0.6–1.0]***	0.7 [0.3–1.6]	0.3 [0.2–0.6]***
Household wealth quintile					
Poorest	1.1 [0.9–1.4]	1.3 [0.9–1.7]	1.1 [0.8–1.5]	0.5 [0.2–1.2]	1.1 [0.5–2.2]
Poor	1.1 [0.9–1.3]	1.1 [0.8–1.4]	1.2 [0.9–1.7]	0.8 [0.3–1.8]	0.6 [0.3–1.3]
Medium	1.1 [0.9–1.3]	1.1 [0.8–1.4]	1.2 [0.9–1.6]	0.8 [0.4–1.6]	0.9 [0.4–1.7]
Rich	1.2 [1.0–1.4]	1.3 [1.0–1.7]	1.2 [0.9–1.6]	0.7 [0.3–1.7]	0.9 [0.5–1.7]
Richest	Ref.	Ref.	Ref.	Ref.	Ref.
Healthcare sector					
Private	Ref.	Ref.	Ref.	Ref.	Ref.
Public	0.3 [0.3–0.4]***	0.3 [0.2–0.3]***	0.4 [0.3–0.5]***	0.7 [0.4–1.2]	0.8 [0.5–1.3]
Both	1.5 [1.1–1.9]***	1.6 [1.1–2.4]**	0.9 [0.4–1.7]	—	1.2 [0.3–5.4]
OD with different care					
Coexisting	1.0 [0.9–1.3]	1.0 [0.8–1.3]	1.0 [0.7–1.4]	1.0 [0.4–2.6]	1.7 [0.8–3.9]
Community-based	1.4 [1.1–1.6]**	1.2 [0.9–1.6]	1.3 [1.0–1.9]	1.3 [0.6–3.2]	2.0 [1.1–3.8]**
Health center-based [high]	1.0 [0.8–1.2]	1.0 [0.7–1.3]	1.1 [0.8–1.5]	0.9 [0.4–1.9]	1.3 [0.7–2.6]
Health center-based [low]	0.8 [0.7–1.0]	0.8 [0.6–1.0]	0.8 [0.6–1.1]	1.3 [0.5–3.0]	1.5 [0.8–2.6]
Hospital-based	Ref.	Ref.	Ref.	Ref.	Ref.

(*) P-value<0.05, (**) P-value<0.01, (***) P-value<0.001

Abbreviation: ARR, adjusted risk ratio; CI: confidence interval; Ref., reference group; OD, operational district

Note: Health center-based (high) means the OD with high coverage (six out of nine) of health centers with the WHO PEN; Health center-based (low) means the OD with low coverage (six out of 25) of health centers with the WHO PEN; P-values<0.05, <0.01, <0.001 are in bold, indicating the variables are significantly associated with expenditure

For peer review only

DISCUSSION

The results show that the use of the public healthcare system remains low for all groups in our study, with about one in every five healthcare visits taking place in the public sector overall. People with chronic conditions, HEF membership, living in the OD with *community-based care* contributed to public healthcare uptake. The healthcare expenditure was significantly reduced when patients used public healthcare services, regardless of HEF membership. However, the reduction in spending was more noticeable in people with HEF membership. In contrast, expenditure was higher among patients living in the OD with *community-based care.*

People in Cambodia predominantly used healthcare in private facilities for outpatient curative care.[21] Our study showed that this is also the case for people with chronic conditions such as T2D and HTN although this group had a slightly higher rate of using public healthcare services. This result is congruent with earlier findings that approximately 61% of T2D and/or HTN patients received their initial diagnosis in private settings.[8] A qualitative study in Cambodia suggested that people with T2D did not prefer diabetes services at public facilities because they were less accessible due to geographical factors or distance and limited medication supply.[25] Our findings suggest that healthcare quality and access to public healthcare services is still below the expectations of people. To increase public healthcare utilization, efforts need to be made to expand the coverage and quality of T2D and HTN services in public healthcare facilities. However, this may require time and resources, which will have budget implications, while Cambodia may not be able to increase the budget for health more than its economic growth rate of about 7% per year.[26] Another approach could be to recognize the role of private healthcare providers and include them in the coverage schemes. This would mean that they are required to

For peer review only

comply with government policies and regulations. The practical, financial, legal, and political implications of such strategies need to be examined.

Previous studies revealed that HEF membership contributed to the health service uptake at public facilities and reduced healthcare expenditure in general users.[16 27] Our findings extended the understanding that HEF membership has also increased public healthcare use and substantially reduced healthcare spending among people with T2D and/or HTN. Since HEF benefits are only available in public healthcare facilities, it is not surprising that it also contributes to increasing service uptake in public facilities. These findings suggest that HEF membership should be expanded among people with chronic conditions, alongside with improving service coverage and quality. By doing so, the health services with quality for people with T2D and/or HTN will be more accessible with fewer financial barriers.

Community-based care contributed to the higher public service uptake among T2D and/or HTN, but it also contributed to the higher expenditure for the users. In ODs with this model, PEs refer patients to the public referral hospitals, so it is not surprising that the public service uptake is slightly higher than other ODs.[28] However, it is somewhat surprising that people with T2D and/or HTN in the OD with *community-based care* spent more on their health services. This may be explained by higher unit costs spent by the supply side in *community-based care* to operate their services, so the patients are charged a higher fee than other models. Our team had conducted a costing study in 2020 to examine the costs to operate services by different care models. The study found that the unit costs were higher for T2D and HTN patients in the *community-based care* than the *hospital-based care* (USD 101 vs. USD 77 for a T2D patient and USD 83 vs. USD 55 for an HTN patient). The higher unit costs in the *community-based care* were driven by adding PE components and field activities to the model. The investment in

For peer review only

community-based care leads to better treatment outcomes, but it is not explored in our study. A previous study provided limited information that a significant proportion of patients in the *community-based care* network had achieved fasting blood glucose goals of 126 mg/dl, from 10% to 45%, and blood pressure goals of 140/90 mm Hg, from 58% to 67%, after a 12-month follow-up.[22] This study, however, did not have a control group (patients outside the network). From this, we can learn two things. First, the adapting and scaling up of PEs should be done with a careful budget plan as PEs incur operational costs. Second, a study investigating the treatment outcomes between different care models and cost-effectiveness remains the gap in Cambodia and should be conducted for a better decision.

There are several strengths in our study. First, our study is among the few to examine healthcare utilization and expenditure both among people with T2D and/or HTN and people without the two conditions. It furthermore covers both the public and private sectors. This broad scope renders the results useful to inform T2D and HTN interventions in Cambodia. Second, we covered a wide range of ODs with different care models and geographically dispersed, which means that our participants are heterogeneous. Third, the data collection was robust and ensured a reliable data set.

Our study also had its limitations. First, it may not represent the national level as most of the study sites (villages) we selected were rural or semi-rural, which may lead to overestimating the healthcare utilization in public facilities. Second, the ODs were purposively selected with oversampling the OD with interventions, increasing the service uptake in public facilities. This may lead to overestimating the public healthcare use in our study. Third, we only calculated the healthcare expenditure for those who used the service in the three months preceding the survey, which cannot be generalized to the target population in the study (people aged 40 years or older).

For peer review only

However, it is unlikely to be significant because we focused more on factors associated with increasing or reducing healthcare expenditure. Fourth, the sample size may be relatively small when we stratified them into four groups. Therefore, variables that were not significantly associated with the dependent variables in our study may be due to the insufficient sample size.

CONCLUSION

Healthcare utilization at public healthcare facilities is relatively low for all groups; however, it is higher in people with chronic conditions. HEF membership and *community-based care* contributed the higher public healthcare utilization in people with chronic conditions. Using public healthcare services regardless of HEF status reduced the healthcare expenditure, but the reduction in spending was more noticeable in people with HEF membership. To increase public healthcare utilization among people with T2D and/or HTN, the public healthcare system should further improve care quality, expand social health protection, and expand community-based care for this population. However, these may require more time and resources. One potential strategy in the short run is to partner the private sector with the public sector. Future research should link healthcare use and expenditure across different healthcare models to actual treatment outcomes to denote areas for further investment.

For peer review only

ACKNOWLEDGMENTS

We would like to thank all the participants in the study and all data collectors for carefully following the instructions.

CONFLICT OF INTERESTS

All authors declare no conflict of interest.

AUTHOR CONTRIBUTIONS

SC, PI, VT, EW designed the study; SC analyzed data; all authors wrote the manuscript.

DATA AVAILABILITY STATEMENT

Data are available on reasonable request. Data are available on reasonable request to Dr. Por Ir (ipor@niph.org.kh).

FUNDING

This work was funded by the Belgian Government through the VLIR-UOS project, the Fourth Funding Agreement (FA4) Programme (2017–2021) between the Belgian Directorate of Development Cooperation and the Institute of Tropical Medicine, Antwerp, and the Horizon2020 Framework Program of the European Union, grant no 825432.

For peer review only

REFERENCES

1. World Health Organization. Cardiovascular diseases, 2020. Available: http://origin.who.int/cardiovascular_diseases/en/ [Accessed January 16, 2022].

2. World Health Organization. Global report on diabetes, 2016. Available: https://www.who.int/publications/i/item/9789241565257 [Accessed January 16, 2022].

3. World Health Organization. Hypertension, 2019. Available: https://www.who.int/news-room/fact-sheets/detail/hypertension [Accessed January 16, 2022].

4. University of Health Sciences. National noncommunicable disease risk factor survey: STEPS survey 2016. Phnom Penh: University of Health Sciences, 2016.

5. World Health Organization. The Kingdom of Cambodia health system review, 2015. Available: https://apps.who.int/iris/bitstream/handle/10665/208213/9789290616917_eng.pdf?sequence=1&isAllowed=y [Accessed January 16, 2022].

6. University of Health Sciences (UHS) and the Preventive Medicine Department (DPM) of the Ministry of Health and World Health Organization (WHO). Prevalence of non-communicable disease risk factors in Cambodia: STEPS Survey, 2010. Available: https://www.who.int/ncds/surveillance/steps/2010_STEPS_Report_Cambodia.pdf [Accessed January 16, 2022].

7. University of Health Sciences (UHS) and the Preventive Medicine Department (DPM) of the Ministry of Health and World Health Organization (WHO). Prevalence of non-communicable disease risk factors in Cambodia: STEPS Survey, 2016.

8. Bigdeli M, Jacobs B, Men CR, et al. Access to Treatment for Diabetes and Hypertension in Rural Cambodia: Performance of Existing Social Health Protection Schemes. *PLOS One* 2016;11(1):e0146147.^

9. Ministry of Health - Cambodia. National standard operating procedure for diabetes and hypertension management in primary care 2019. Phnom Penh: MoH, 2019.

10. Rao M, van Pelt M, LoGerfo J, et al. Utilization of diabetes management health care services and its association with glycemic control among patients participating in a peer educator-based program in Cambodia. *PloS One* 2020;15(6):e0235037–e37.^

11. Department of Planning and Health Information. Health information system master plan 2016–2020. Phnom Penh: Cambodian Ministry of Health, 2017.

12. Health Policy Plus. A Legal and Regulatory Assessment of Private Healthcare Provision in Cambodia. Washington, DC, USA: Health Policy Plus, 2019.

13. Ministry of Health - Cambodia. Health Progress Report, 2018. Available: http://moh.gov.kh/content/uploads/2017/05/2019_MoH-Final01-Low.pdf [Accessed January 16, 2022].

14. Bureau-Point E, Baxerres C, Chheang S. Self-Medication and the Pharmaceutical System in Cambodia. *Med Anthropol* 2020;39(8):765–81.^

15. Jacobs B, Price N. The impact of the introduction of user fees at a district hospital in Cambodia. *Health Policy Plan* 2004;19(5):310–21.^

16. Annear PL, Tayu Lee J, Khim K, et al. Protecting the poor? Impact of the national health equity fund on utilization of government health services in Cambodia, 2006–2013. *BMJ Glob Health* 2019;4(6):e001679.^

For peer review only

17. Kaba MW, Baesel K, Poch B, et al. IDPoor: a poverty identification programme that enables collaboration across sectors for maternal and child health in Cambodia. *BMJ* 2018;363:k4698.^

18. National Social Security Fund (NSSF). Health Insurance Scheme, 2021. Available: https://www.nssf.gov.kh/default/health-care-scheme-2/ [Accessed January 16, 2022].

19. Kolesar RJ, Pheakdey S, Jacobs B, et al. Expanding social health protection in Cambodia: An assessment of the current coverage potential and gaps, and social equity considerations. *Int Soc Secur Rev* 2020;73(1):35–63.^

20. World Health Organization. Regional Office for the Western/Pacific. Cambodia national health accounts (2012–2016): health expenditure report, 2019. Available: http://iris.wpro.who.int/handle/10665.1/14362 [Accessed January 16, 2022].

21. World Health Organization. Regional Office for the Western/Pacific. Financial health protection in Cambodia (2009–2016): analysis of data from the Cambodia Socioeconomic Survey, 2019. Available: http://iris.wpro.who.int/handle/10665.1/14436 https://apps.who.int/iris/handle/10665/330701 [Accessed January 16, 2022].

22. Taniguchi D, LoGerfo J, van Pelt M, et al. Evaluation of a multi-faceted diabetes care program including community-based peer educators in Takeo province, Cambodia, 2007-2013. *PloS One* 2017;12(9):e0181582.^

23. Janssens B, Van Damme W, Raleigh B, et al. Offering integrated care for HIV/AIDS, diabetes and hypertension within chronic disease clinics in Cambodia. *Bulletin of the World Health Organization* 2007;85(11):880-85.^

24. KoBoToolbox at the Harvard Humanitarian Initiative. KoBoToolbox, Available: https://www.kobotoolbox.org/#home [Accessed January 16, 2022].

For peer review only

25. Nang EEK, Dary C, Hsu LY, et al. Patients' and healthcare providers' perspectives of diabetes management in Cambodia: a qualitative study. *BMJ Open* 2019;9(11):e032578–e78.^

26. Thin K, Sokbunthoeun S. Assessing government's fiscal space for moving towards universal health coverage in Cambodia. *Global Journal of Medicine and Public Health* 2017;6(3):11.^

27. Jithitikulchai T, Feldhaus I, Bauhoff S, et al. Health equity funds as the pathway to universal coverage in Cambodia: care seeking and financial risk protection. *Health Policy Plan* 2021;36(1):26–34.^

28. van Olmen J, Eggermont N, van Pelt M, et al. Patient-centred innovation to ensure access to diabetes care in Cambodia: the case of MoPoTsyo. *J Pharm Policy Pract* 2016;9(1):1.^

For peer review only

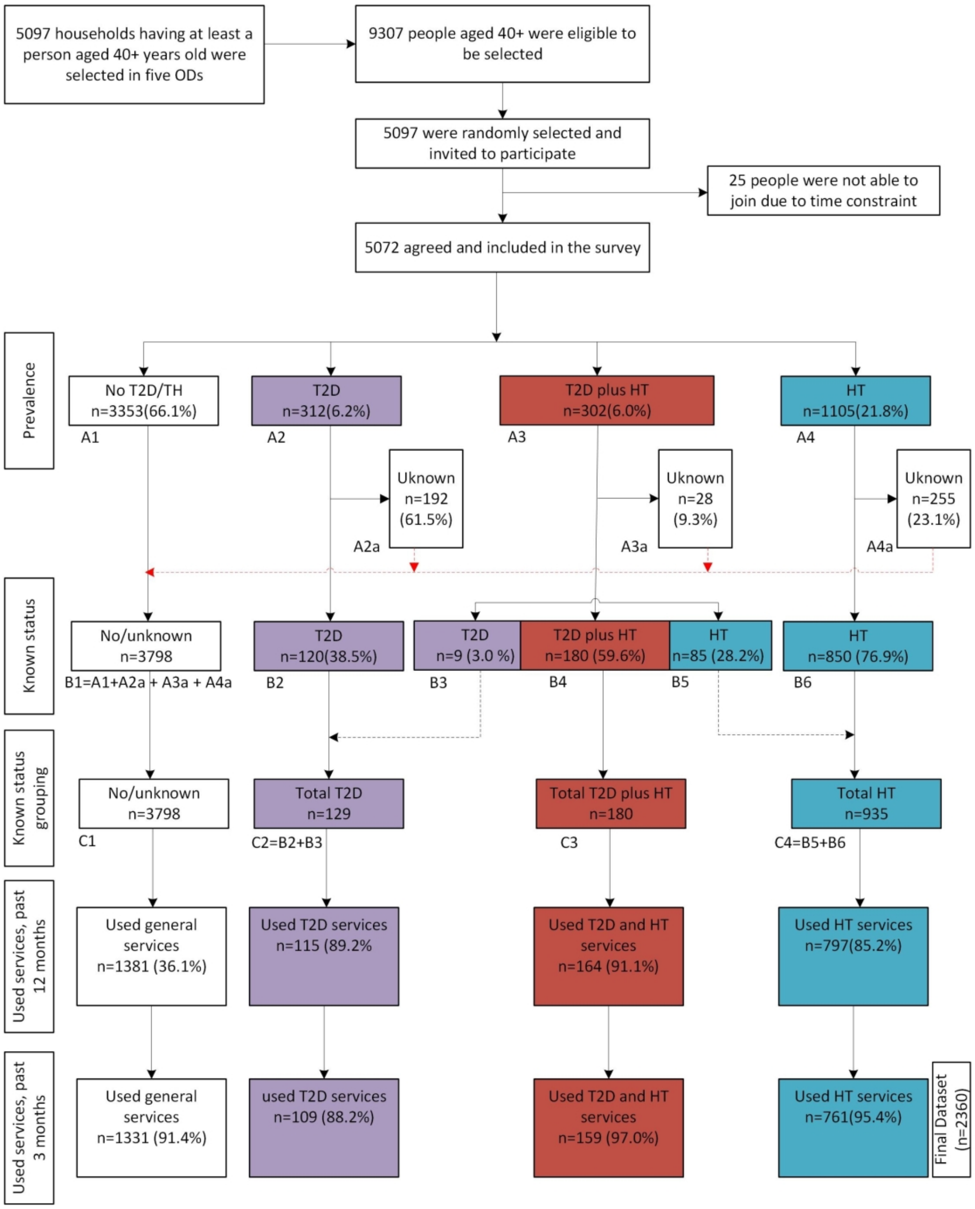

Figure 1. Data flow from household selection to final dataset in this study, Cambodia, 2020

199x244mm (300 x 300 DPI)

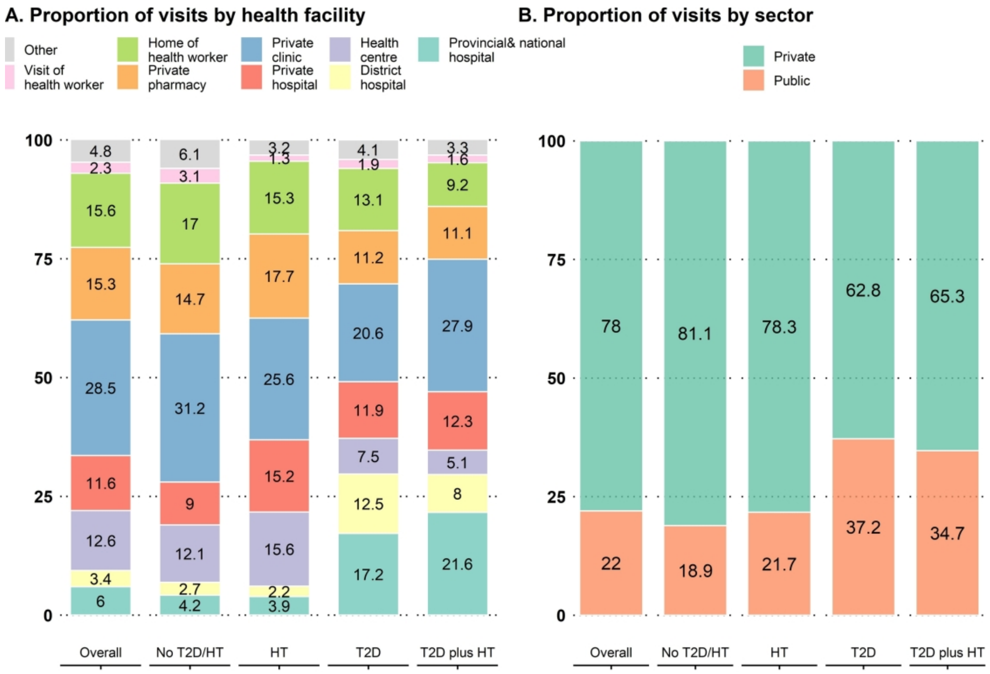

Figure 2. Proportion of visits to public and private facilities in Cambodia, 2020

254x177mm (300 x 300 DPI)

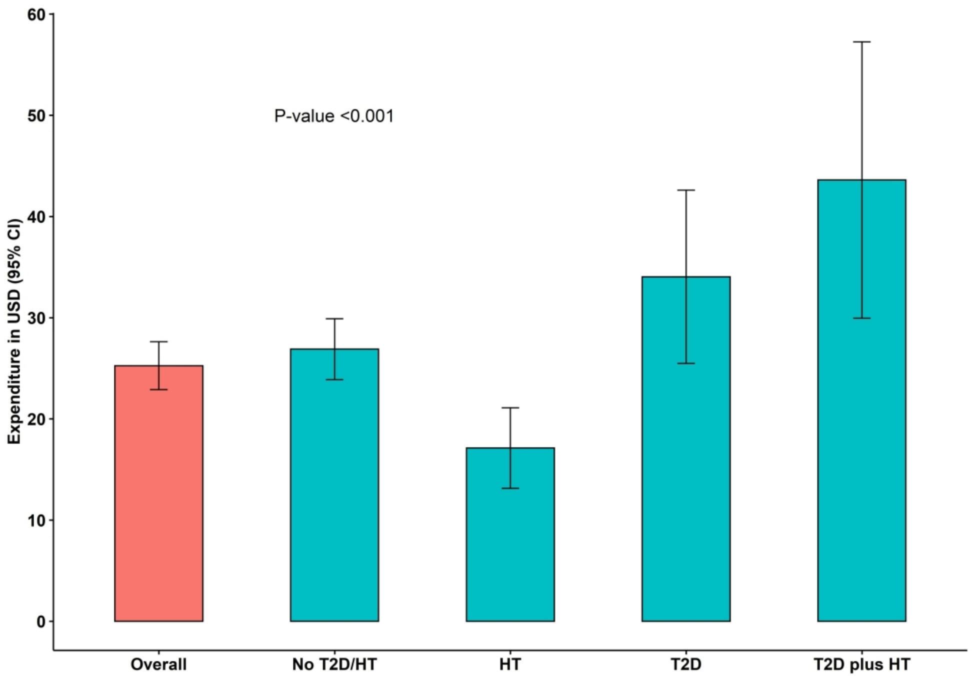

Figure 3. Healthcare expenditure by patient groups in the three months preceding the survey in 2020, Cambodia

254x177mm (300 x 300 DPI)

Annexure 1. Locations of the five studied ODs in Cambodia, 2020.

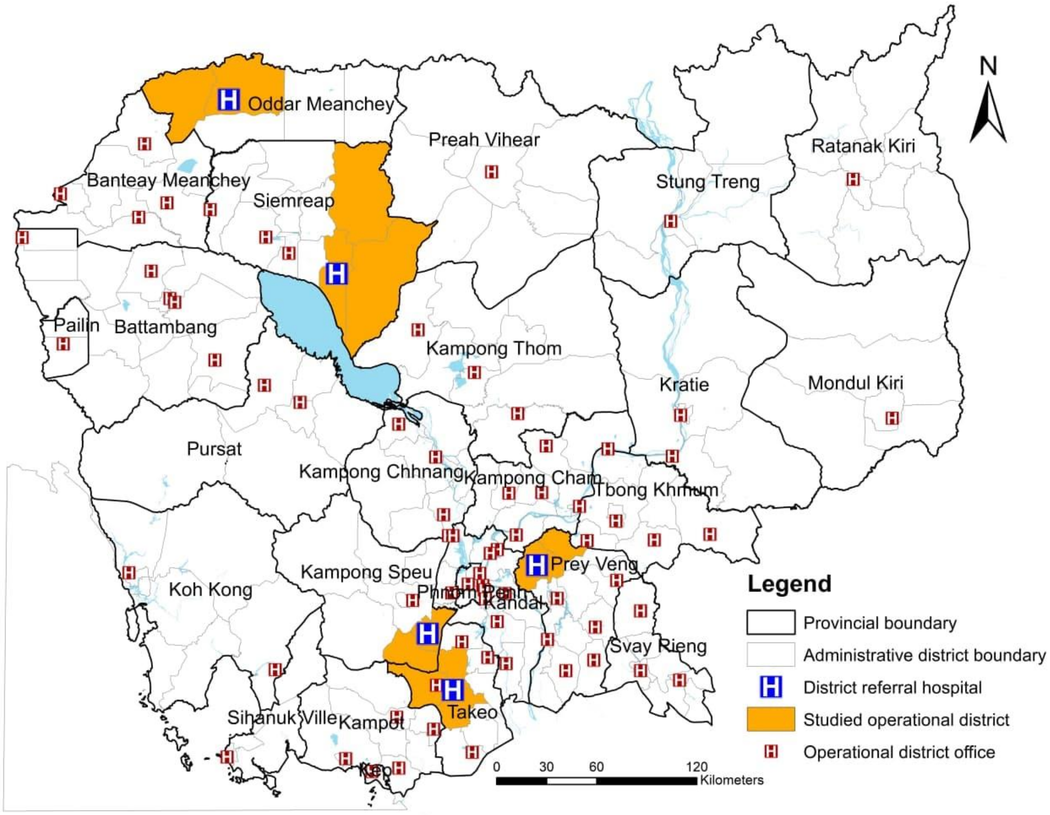

Annexure 2. Principal Component Analysis

The wealth quintile was used to classify participants into five socio-economic classes: poorest, poor, medium, rich, and richest. The wealth quintile was constructed using principal component analysis (PCA). The head of the family or family representative was interviewed using a 30-item tool that was taken from the 2014 Cambodia Demographic Health Survey. The tool is designed to classify households' wealth based on their properties. The binary answer questions were coded with 0 for no and 1 for yes. The questions with non-binary-answer were split into binary questions with a code of 0 for no and 1 for yes [21]. The first score component was used to classify the wealth into five quintiles [21]. The lower score represents the poorer status, and the higher score represents the richer status. The list of questions used for PCA is in the table below.

For peer review only

HOUSEHOLD SOCIO-ECONOMIC STATUS

QH3. Does your household have? [CDHS]		
301. electricity	1=Yes	0=No
302. a radio?	1=Yes	0=No
303. a television?	1=Yes	0=No
304. a mobile telephone?	1=Yes	0=No
305. If yes, is it a smartphone?	1=Yes	0=No
306. a refrigerator?	1=Yes	0=No
307. a wardrobe?	1=Yes	0=No
308. a sewing machine or loom?	1=Yes	0=No
309. CD/DVD player?	1=Yes	0=No
310. a generator/battery/solar panel?	1=Yes	0=No

Does any member of your household own/have ? [CDHS]		
311. a watch?	1=Yes	0=No

312. a bicycle or cyclo?	1=Yes	0=No
313. a motorcycle or motor-scooter?	1=Yes	0=No
314. a motorcycle-cart	1=Yes	0=No
315. a oxcart or horsecart?	1=Yes	0=No
316. a car or truck, tractor or van?	1=Yes	0=No
317. a boat with a motor?	1=Yes	0=No
318. a boat without a motor?	1=Yes	0=No
319. any agricultural land?	1=Yes	0=No
320. any livestock, herds, other farm animals, or poultry?	1=Yes	0=No
321. a bank account?	1=Yes	0=No

322	What is the main material of the floor?<br>*Record observation*	1. Earth/sand/clay<br>2. Dung<br>3. Wood planks<br>4. Palm/bamboo<br>5. Parquet or polished wood<br>6. Vinyl or Asphalt strips<br>7. Ceramic tiles<br>8. Cement tiles<br>9. Cement<br>10. Floating house<br>11. Other (specify)
323	What is the main material of the roof?<br>*Record observation*	1. Bamboo/thatch/palm leaf<br>2. Rustic mat<br>3. Wood planks<br>4. Cardboard<br>5. Plastic sheet<br>6. Metal<br>7. Wood<br>8. Calamine/cement fiber<br>9. Ceramic tiles<br>10. Clay tiles<br>11. Cement<br>12. Other (specify)
324	What is the main material of the exterior walls?<br>*Record observation*	1. Palm/bamboo/thach<br>2. Dirt<br>3. Bamboo with mud<br>4. Straw with mud<br>5. Stone with mud<br>6. Uncovered adobe

For peer review only

		7. Plywood<br>8. Cardboard<br>9. Reused wood<br>10. Metal<br>11. Cement<br>12. Stone with lime/cement<br>13. Bricks<br>14. Cement blocks<br>15. Covered adobe<br>16. Wood planks/shingles<br>17. Other (specify)
325	How many rooms in this household are used for sleeping?	Rooms:…………………
326	What is the main source of drinking water during the wet season for members of your household?	1. Piped into dwelling<br>2. Piped to yard/plot<br>3. Public tap/standpipe<br>4. Tube well or borehole<br>5. Protected well<br>6. Unprotected well<br>7. Protected spring<br>8. Unprotected spring<br>9. Rainwater<br>10. Tanker truck<br>11. Cart with small tank<br>12. Surface water (river/dam/lake/pond/stream/canal/irrigation channel<br>13. Bottled water<br>14. Other (specify)
327	What is the main source of drinking water during the dry season for members of your household?	1. Piped into dwelling<br>2. Piped to yard/plot<br>3. Public tap/standpipe<br>4. Tube well or borehole<br>5. Protected well<br>6. Unprotected well<br>7. Protected spring<br>8. Unprotected spring<br>9. Rainwater<br>10. Tanker truck<br>11. Cart with small tank<br>12. Surface water (river/dam/lake/pond/stream/canal/irrigation channel<br>13. Bottled water<br>14. Other (specify)

For peer review only

328	Do you do anything to the water to make it safer to drink?	1. Yes, always<br>2. Yes, sometime<br>3. No<br>4. Don't know
329	What do you usually do to make the water safer to drink?<br>*Record all mentioned.*	1. Boil<br>2. Add bleach/chlorine<br>3. Strain through a cloth<br>4. Use water filter (ceramic/sand/composite/etc.)<br>5. Solar disinfection<br>6. Let it stand and settle<br>7. Other (specify)
330	What kind of toilet facility do members of your household usually use?	1. Flush to piped sewer system (not shared with other households)<br>2. Flush to septic tank (not shared with other households)<br>3. No facility/bush/field<br>4. Other type of toilet (specify)

For peer review only

Annexure 3. Individual Survey Questionnaire

INTRO:
Hello, my name is ____and I am from the National Institute of Public Health. You has been randomly selected to participate in this study [on the scaling up of diabetes and hypertension in Cambodia] based on information from your household. The information you give will be kept confidential and no personal details will appear in any record. This interview will take approximately 60 minutes. You do not have to answer any question you don't want to and you can stop the interview at any time. There will also be measurements of your blood pressure, weight and height, waist and hip circumferences during and after the interview, and for tomorrow early morning we would like to test your fasting blood glucose [HbA1c and creatinine level for known and suspected diabetes cases]. We very appreciate your participation and information.

ELIGIBILITY & RECRUITMENT CHECK
(Instructions: To be filled in by data collectors before participants sign informed consent)

Q.N	Description & questions	RESPONSE		Ref:
Q1	Has the individual been a usual member of the household and stayed in the household the night before the interview or had not been absent for more than 6 months?	0 = No	1 = Yes	CDHS
Q2	Is the individual 40 or more than 40 of age?<br><br>*IF POSSIBLE, CHECK ID CARD*	0 = No	1 = Yes	WHO PEN SOP
	If NO, DO NOT continue.			
Q3	Is the individual physically and mentally capable to answer the questions?	0 = No	1 = Yes	
	If NO, DO NOT continue			
Q4	Are you willing to participate in the study?	0 = No	1 = Yes	
	If NO, DO NOT continue<br>If YES, CONSENT obtained			
**Q5. Consent obtained**		0 = No	1 = Yes	

SECTION 1: SOCIO-DEMOGRAPHIC INFORMATION

Q.N	Description & questions	RESPONSE	Type of Variable	Field	Ref:
Q6	How old are you?<br>*-Record in years as stated by the participant*<br>*-Record 99 if don't know*	____________Years	Quantitative discrete	Age	WHO STEPS
Q7	Sex of participant<br>*Record sex of the participant as observed*	1 = Male<br>2 = Female	Categorical binary	Sex	WHO STEPS
Q8	What is your marital status?	1=Married or living together<br>2=Divorced or separated<br>3=Widowed	Categorical nominal	Marital Status	CDHS

For peer review only - http://bmjopen.bmj.com/site/about/guidelines.xhtml

	-Record 88 if refuse to answer	4=Never married and never lived together			
Q9	What is your highest educational level?<br><br>*Record 99 if don't know*	1=No formal schooling<br>2=Less than primary school<br>3=Primary school<br>4=Secondary school<br>5=High school<br>6=College/University<br>7=Post graduate degree	Categorical ordinal	Educational level	WHO STEPS
Q10	What is your ethnic group?	1 = Khmer<br>2 = Vietnamese<br>3 = Chinese<br>4 = Cham (Muslim)<br>5 = Other	Categorical nominal	Ethnicity	WHO STEPS
Q11	Which of the following best describes your main work status within the past 12 months?<br><br>*Record 88 if refuse to answer*	1=Household tasks<br>2=Civil servant<br>3=Employee of private company/NGO<br>4=Self-employed farmer<br>5=Large-scale farmer with employees<br>6=Self-employed in small business<br>7=Running a big business with employees<br>8=Casual worker<br>9=Working abroad<br>10=At school (pupil/student)<br>11=Unemployed or not eligible<br>12=Retired	Categorical nominal	Occupation	CDHS
Q12	Taking the past year, can you give an estimate of your annual income if I read some options to you?<br><br>*Record 88 if refuse to answer*	1 = no earnings<br>2 = less than or 250 USD<br>3 = more than 251- 1500 USD<br>4 = more than 1501 – 3500 USD<br>5 = more than 3501 USD	Categorical ordinal	Individual income	WHO STEPS

**SECTION 2: HEALTH STATUS AND QUALITY OF LIFE**					
**Q.N**	**Description and Questions**	**Response**	**Type of Variable**	**Field**	**Ref:**

For peer review only - http://bmjopen.bmj.com/site/about/guidelines.xhtml

Q13	At this point of time in your life, how would you describe:<br><br>Q 13.1. Your home situation [_____]<br>Q 13.2. Your family relationships [_____]<br>Q 13.3. Your finances [_____]<br>Q 13.4. Your work situation [_____]<br><br>*-Record 99 if don't know and 88 if refuse*	1 = Excellent<br>2 = Very good<br>3 = Good<br>4 = Fair<br>5 = Poor	Categorical Ordinal	QoLife	GACD book
Q14	How good or bad is your health today?<br><br>*-The scale is numbered from 0 to 100.*<br>*-100 means the best health you can imagine.*<br>*-0 means the worst health you can imagine.*<br>*-Please mark an X on the scale to indicate how your health is today.*<br><br>*-Record 99 if don't know and 88 if refuse*	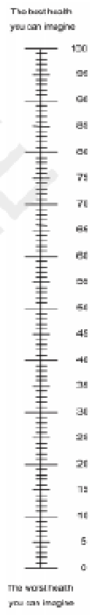	Quantitative discrete	Gen. Health	EuroQoL
Q15	Have you ever been told by a doctor or other health worker that you have hypertension?<br><br>*-Record 99 if don't know/unsure*	0 = No<br>1 = Yes	Categorical binary	HT_self-report diagnosis	WHO STEPS
Q16	Have you ever been told by a doctor or other health worker that you have diabetes?<br><br>*-Record 99 if don't know/unsure*	0 = No<br>1 = Yes	Categorical binary	DM_self-report diagnosis	WHO STEPS

For peer review only

For peer review only - http://bmjopen.bmj.com/site/about/guidelines.xhtml

Q17	Have you ever been told by a doctor or other health worker that you have heart problems?<br><br>*-Record 99 if don't know/unsure*	0 = No<br>1 = Yes	Categorical binary	Co-morbidity (CVD)	WHO STEPS
Q18	Have you ever been told by a doctor or other health worker that you have symptoms suggestive of a stroke?<br><br>*-Record 99 if don't know/unsure*	0 = No<br>1 = Yes	Categorical binary	Co-morbidity (CVD)	WHO STEPS
Q19	Have you ever been told by a doctor or other health worker that you have chronic kidney disease?<br><br>*-Record 99 if don't know/unsure*	0 = No<br>1 = Yes	Categorical binary	Co-morbidity (CKD)	WHO STEPS
Q20	How many natural teeth do you have?<br><br>*-Record 99 if don't know*	0 = None<br>1 = 1-9 teeth<br>2 = 10-19 teeth<br>3 = 20 teeth or more	Categorical ordinal	Dental health	WHO Oral Health Questionnaire
Q21	During the past 12 months, did your teeth or mouth cause any pain or comfort?<br><br>*-Record 99 if don't know*	0 = No<br>1 = Yes	Categorical binary	Dental health	WHO Oral Health Questionnaire
Q22	Over the last 2 weeks, how often have you been bothered by any of the following problems?<br><br>Q 22.1. Little interest or pleasure in doing things [____]<br>Q 22.2. Feeling down, depressed, or hopeless. [____]<br>Q 22.3. Trouble falling or staying asleep, or sleeping too much. [____]	0 = Not at all<br>1 = Several days<br>2 = More than half the days<br>3 = Nearly everyday	Categorical ordinal	Mental health	PHQ-9

For peer review only - http://bmjopen.bmj.com/site/about/guidelines.xhtml

	Q 22.4. Feeling tired or having little energy. [____]<br>Q 22.5. Poor appetite or overeating [____]<br>Q 22.6. Feeling bad about yourself – or that you are a failure or make yourself or down your family [____]<br>Q 22.7. Trouble concentrating on things, such as reading the newspaper or watching television [____]<br>Q 22.8. Moving or speaking so slowly that other people could have noticed? Or the oppositebeing so fidgety or restless that you have been moving around a lot more than usual [____]<br>Q 22.9. Thoughts that you would be better off dead or of hurting yourself in some ways [____]				
Q42	We would like to confirm that:<br><br>*-This question is for categorizing respondents for the following sections and it is also important to ask respondents to confirm their main conditions in this survey.*<br><br>*-IF the answer is "0", go to Section 3*<br>*-IF the answer is "1", go to Section 3a*<br>*-IF the answer is "2", go to Section 3b*<br>*-IF the answer is "3", go to Section 3c*	0 = Neither hypertension nor diabetes<br><br>1 = Only hypertension<br><br>2 = Only diabetes<br><br>3 = Both diabetes and hypertension			

For peer review only

For peer review only - http://bmjopen.bmj.com/site/about/guidelines.xhtml

SECTION 3: HEALTH CARE UTILIZATION					
**Q.N**	**Description and Questions**	**Response**	**Type of Variable**	**Field**	**Ref:**
Q23	Have you sought medical treatment or advice as an outpatient from anyone in the past 3 months?<br><br>*-If No, go to Question 40.*	0 = No<br>1 = Yes	Categorical variable	Medical advice	GACD book
Q24	Where did you seek medical advice or treatment for illness in the past 3 months?<br><br>*-More than one answer can be selected.*<br>*-Data collectors can use probes to help respondents determine the types of health facilities in the Response Column.*<br>*-Record 99 if don't know and 88 if refuse*	1= National hospital (PP)<br>2= Provincial hospital (RH)<br>3= District hospital (RH)<br>4= Health centre<br>5= Health post<br>6= Provincial rehabilitation centre (PRC) or Community-based rehabilitation (CBR)<br>7= Other public; specify:<br>8= Private hospital<br>9= Private clinic<br>10= Private pharmacy<br>11= Home/Office of trained health worker/nurse<br>12= Visit of trained health worker/nurse<br>13= Other private medical; specify:<br>14= Shop selling drugs/market<br>15= Kru Khmer/ Magician	Categorical Nominal	Care provider_3M	CDHS and GACD book

For peer review only - http://bmjopen.bmj.com/site/about/guidelines.xhtml

		16= Monk/religious leader<br>17= Traditional birth attendant<br>18= Oversee medical service<br>19= Other; specify:			
From Q25-Q39, it is a set of questions that are asked following choices selected in Q24. If 2 or 3 choices were selected in Q24, Q25-Q39 would appear 2 or 3 times, accordingly.					
Q25	How many times did you visit the selected place**(s)** in Q24 in the past three months?	_______ times	Quantitative discrete	Number of visits	GACD
Q26	How much in total was spent on the treatment at the selected place(s) in Q24?<br><br>*-Record 99 if don't know and 88 if refuse*	0 = free/no cost<br>1 = in kind<br>2 = ________Riels	-Categorical nominal<br>-<br>Quantitative Continuous	Cost of treatment	CDHS
Q27	How did you pay for the treatment cost at the selected place(s) in Q24?<br><br>*-Record 99 if don't know and 88 if refuse*	1= Health Equity Fund<br>2= Voucher<br>3= Fee Exemption<br>4= NGO<br>5= National Social Security Fund<br>6= Community-Based Health Insurance<br>7= Health Insurance through Employer<br>8= Other Privately Purchased Commercial Health Insurance	-Categorical nominal	Payment method	GADC adapted to context specific

For peer review only

		9= Wage/income<br>10= Loan/ Ton Tin<br>11= Sale of Assets<br>12= Gift from Relative<br>13= Savings<br>14= Other			
Q28	How much in total was spent on transport to go to and return from the selected place(s) in Q24?<br><br>*-Record 99 if don't know and 88 if refuse*	0 = free/no cost<br>1 = in kind<br>2 = ________Riels	-Categorical nominal<br>-<br>Quantitative Continuous	Cost of transp ort	CDHS
Q29	On average how many hours do you spend to get treatment/advices from the selected place(s) in Q24?<br><br>*-Record 99 if don't know and 88 if refuse*	________Hours	Quantitative continuous	Time spendi ng	GACD book (Adapted)
Q30	How satisfied are you with the effect of your {treatment/care} at the selected place(s) in Q24?<br><br>*-Record 99 if don't know/unsure*	0 = Very satisfied<br>1 = Satisfied<br>2 = Neither satisfied nor dissatisfied<br>3 = Dissatisfied<br>4 = Very dissatisfied	Categorical ordinal	Patien t satisfa ction of care servic es	SAPS 2006
Q31	How satisfied are you with the explanations the {doctor/other health professional} has given you about the results of your {treatment/care} at the selected place(s) in Q24?<br><br>*-Record 99 if don't know/unsure*	0 = Very satisfied<br>1 = Satisfied<br>2 = Neither satisfied nor dissatisfied<br>3 = Dissatisfied<br>4 = Very dissatisfied	Categorical ordinal	Patien t satisfa ction of care servic es	SAPS 2006

For peer review only - http://bmjopen.bmj.com/site/about/guidelines.xhtml

Q32	The {doctor/other health professional} at the selected place(s) in Q24 was very careful to check everything when examining you.<br><br>*-Record 99 if don't know/unsure*	0 = Strongly agree<br>1 = Agree<br>2 = Not sure<br>3 = Disagree<br>4 = Strongly disagree	Categorical ordinal	Patient satisfaction of care services	SAPS 2006
Q33	At the selected place(s) in Q24, how satisfied were you with the choices you had in decisions affecting your health care?<br><br>*-Record 99 if don't know/unsure*	0 = Very satisfied<br>1 = Satisfied<br>2 = Neither satisfied nor dissatisfied<br>3 = Dissatisfied<br>4 = Very dissatisfied	Categorical ordinal	Patient satisfaction of care services	SAPS 2006
Q34	How much of the time did you feel respected by the {doctor/other health professional} at the selected place(s) in Q24?<br><br>*-Record 99 if don't know/unsure*	0 = All of the time<br>1 = Most of the time<br>2 = About half the time<br>3 = Some of the time<br>4 = None of the time	Categorical ordinal	Patient satisfaction of care services	SAPS 2006
Q35	At the selected place(s) in Q24, the time you had with the {doctor/other health professional} was too short.<br><br>*-Record 99 if don't know/unsure*	0 = Strongly agree<br>1 = Agree<br>2 = Not sure<br>3 = Disagree<br>4 = Strongly disagree	Categorical ordinal	Patient satisfaction of care services	SAPS 2006
Q36	Are you satisfied with the care you received in the selected place(s) in Q24?<br><br>*-Record 99 if don't know/unsure*	0 = Very satisfied<br>1 = Satisfied<br>2 = Neither satisfied nor dissatisfied<br>3 = Dissatisfied<br>4 = Very dissatisfied	Categorical ordinal	Patient satisfaction of care services	SAPS 2006

For peer review only - http://bmjopen.bmj.com/site/about/guidelines.xhtml

Q37	Did you get your blood pressure measured at the selected place(s) in Q24?<br><br>-*Record 99 if don't know/unsure*	0 = No<br>1 = Yes	Categorical variable	Access to blood pressure testing	Opinion
Q38	Did you get your blood glucose tested at the selected place(s) in Q24?<br><br>-*Record 99 if don't know/unsure*	0 = No<br>1 = Yes	Categorical variable	Access to blood glucose testing	Opinion
Q39	Would you recommend the selected place(s) in Q24 to others?<br><br>-*Record 99 if don't know/unsure*	1 = Not recommended<br>2 = Recommend with reservations<br>3 = Recommend<br>4 = Highly recommend	Categorical ordinal	Patient satisfaction of care services	Opinion
Q40	Have you ever had your blood glucose tested in the last three years?<br><br>-*Record 99 if don't know/unsure*	0 = No<br>1 = Yes	Categorical binary	Testing DM (CoC)	Opinion
Q41	Have you ever had your blood pressure measured in the last three years?<br><br>-*Record 99 if don't know/unsure*	0 = No<br>1 = Yes	Categorical binary	Testing HT (CoC)	Opinion

For peer review only - http://bmjopen.bmj.com/site/about/guidelines.xhtml

SECTION 3a: HEALTH CARE UTILIZATION FOR HYPERTENSION					
**Q.N**	**Description and Questions**	**Response**	**Type of Variable**	**Field**	**Ref:**
Q43a	How long have you lived with hypertension?<br><br>*-Record 99 if don't know/unsure and 88 if refuse*<br>*-Less than a year is rounded up to one year*<br>*-Standard rounded up formula is applied.*	________Years	Quantitative concrete	Durati on HT	Opinion
Q44a	Where were you first diagnosed as having hypertension?<br><br>*-Record 99 if don't know and 88 if refuse*	1= National hospital (PP)<br>2= Provincial hospital (RH)<br>3= District hospital (RH)<br>4= Health centre<br>5= Health post<br>6= Provincial rehabilitation centre (PRC) or Community-based rehabilitation (CBR)<br>7= Other public; specify:<br>8= Private hospital<br>9= Private clinic<br>10= Private pharmacy<br>11= Home/Office of trained health worker/nurse<br>12= Visit of trained health worker/nurse<br>13= Other private medical; specify:<br>14= Shop selling drugs/market<br>15= Kru Khmer/ Magician	Categorical Nominal	Diagn osis HT (CoC)	Opinion

For peer review only - http://bmjopen.bmj.com/site/about/guidelines.xhtml

		16= Monk/religious leader<br>17= Traditional birth attendant<br>18= Oversee medical service<br>19= MoPoTsyo<br>20= Other; specify:			
Q45a	Where did you first seek advice or treatment for hypertension after being diagnosed?<br><br>*-Record 99 if don't know and 88 if refuse*	1= National hospital (PP)<br>2= Provincial hospital (RH)<br>3= District hospital (RH)<br>4= Health centre<br>5= Health post<br>6= Provincial rehabilitation centre (PRC) or Community-based rehabilitation (CBR)<br>7= Other public; specify:<br>8= Private hospital<br>9= Private clinic<br>10= Private pharmacy<br>11= Home/Office of trained health worker/nurse<br>12= Visit of trained health worker/nurse<br>13= Other private medical; specify:<br>14= Shop selling drugs/market<br>15= Kru Khmer/ Magician<br>16= Monk/religious leader<br>17= Traditional birth attendant	Categorical Nominal	Link to care HT (CoC)	CDHS (Adapted for disease specific)

For peer review only - http://bmjopen.bmj.com/site/about/guidelines.xhtml

		18= Oversee medical service<br>19= MoPoTsyo<br>20= Other; specify:			
Q46a	Did you go to other places for follow up treatment/care for your hypertensive conditions?<br><br>*-Record 88 if refuse*<br>*-If NO, please skip Q47a*	0 = No<br>1 = Yes	Categorical binary	Trust HT	CDHS
Q47a	If yes to Q46a, where else did you go to get follow up treatment/care for your hypertensive conditions?	1= National hospital (PP)<br>2= Provincial hospital (RH)<br>3= District hospital (RH)<br>4= Health centre<br>5= Health post<br>6= Provincial rehabilitation centre (PRC) or Community-based rehabilitation (CBR)<br>7= Other public; specify:<br>8= Private hospital<br>9= Private clinic<br>10= Private pharmacy<br>11= Home/Office of trained health worker/nurse<br>12= Visit of trained health worker/nurse<br>13= Other private medical; specify:<br>14= Shop selling drugs/market	Categorical Nominal	Link to care HT (CoC)	CDHS (Adapted for disease specific)

For peer review only - http://bmjopen.bmj.com/site/about/guidelines.xhtml

		15= Kru Khmer/ Magician<br>16= Monk/religious leader<br>17= Traditional birth attendant<br>18= Oversee medical service<br>19= MoPoTsyo<br>20= Other; specify:			
Q48a	Did you get treatment/care for your hypertensive conditions in the past **12 months?**<br><br>*-Record 88 if refuse*<br>*-If NO, please skip Q49a-64a*	0 = No<br>1 = Yes	Categorical binary	Retain in care HT (CoC)	CDHS (Adapted for disease specific)
Q49a	Are you currently receiving any of the following treatment/advices for your hypertensive conditions prescribed by a doctor or other health care worker?<br><br>Q 49.1a. Drugs (medication) that you have taken in the past two weeks [____]<br>Q 49.2a. Advice to reduce salt intake [___]<br>Q 49.3a. Advice or treatment to lose weight [____]<br>Q 49.4a. Advice or treatment to stop smoking [____]<br>Q 49.5a. Advice to start or do more physical exercise [____]<br><br>*-Record 99 if don't know and 88 if refuse*	0 = No 1= Yes	Categorical ordinal	In treatment for HT (CoC)	STEPS Survey
Q50a	Have you had your blood cholesterol measured in the past 12 months?<br><br>*-Record 99 if don't know and 88 if refuse*	0 = No 1= Yes	Categorical ordinal	In treatment for HT (CoC)	Veerle's suggestion

For peer review only - http://bmjopen.bmj.com/site/about/guidelines.xhtml

Q51a	Where did you seek medical advice or treatment for your hypertensive condition in **the past 3 months**?<br><br>-***More than one answer can be selected***.<br>-*Data collectors can use probes to help respondents determine the types of health facilities in the Response Column.*<br>-*Record 99 if don't know and 88 if refuse*<br>-*If (21=no wehere), go to Q65a*	1= National hospital (PP)<br>2= Provincial hospital (RH)<br>3= District hospital (RH)<br>4= Health centre<br>5= Health post<br>6= Provincial rehabilitation centre (PRC) or Community-based rehabilitation (CBR)<br>7= Other public; specify:<br>8= Private hospital<br>9= Private clinic<br>10= Private pharmacy<br>11= Home/Office of trained health worker/nurse<br>12= Visit of trained health worker/nurse<br>13= Other private medical; specify:<br>14= Shop selling drugs/market<br>15= Kru Khmer/ Magician<br>16= Monk/religious leader<br>17= Traditional birth attendant<br>18= Oversee medical service<br>19= MoPoTsyo<br>20= Other; specify:	Categorical Nominal	Care provider_3M	CDHS and GACD book

For peer review only - http://bmjopen.bmj.com/site/about/guidelines.xhtml

		21= No where			
From Q52a-Q64a, it is a set of questions that are asked following choices selected in Q51a. If 2 or 3 choices were selected in Q51a, Q52a-Q64a would appear 2 or 3 times, accordingly.					
Q52a	How many times did you visit the selected place**(s)** in Q51a in the past three months?	________ times	Quantitative discrete	Numb er of visits	GACD
Q53a	How much in total was spent on the treatment at the selected place(s) in Q51a?<br><br>*-Record 99 if don't know and 88 if refuse*	0 = free/no cost<br>1 = in kind<br>2 = _________Riels	-Categorical nominal<br>-<br>Quantitative Continuous	Cost of treatm ent	CDHS
Q54a	How did you pay for the treatment cost at the selected place(s) in Q51a?<br><br>*-Record 99 if don't know and 88 if refuse*	1= Health Equity Fund<br>2= Voucher<br>3= Fee Exemption<br>4= NGO<br>5= National Social Security Fund<br>6= Community-Based Health Insurance<br>7= Health Insurance through Employer<br>8= Other Privately Purchased Commercial Health Insurance	-Categorical nominal	Paym ent metho d	GADC adapted to context specific

For peer review only - http://bmjopen.bmj.com/site/about/guidelines.xhtml

		9= Wage/income<br>10= Loan/ Ton Tin<br>11= Sale of Assets<br>12= Gift from Relative<br>13= Savings<br>14= Other			
Q55a	How much in total was spent on transport to go to and return from the selected place(s) in Q51a?<br><br>*-Record 99 if don't know and 88 if refuse*	0 = free/no cost<br>1 = in kind<br>2 = ________Riels	-Categorical nominal<br>-<br>Quantitative Continuous	Cost of transport	CDHS
Q56a	On average how many hours do you spend to get treatment/advices from the selected place(s) in Q51a?<br><br>*-Record 99 if don't know and 88 if refuse*	________Hours	Quantitative continuous	Time spending	GACD book (Adapted)
Q57a	How satisfied are you with the effect of your {treatment/care} at the selected place(s) in Q51a?<br><br>*-Record 99 if don't know/unsure*	0 = Very satisfied<br>1 = Satisfied<br>2 = Neither satisfied nor dissatisfied<br>3 = Dissatisfied<br>4 = Very dissatisfied	Categorical ordinal	Patient satisfaction of care services	SAPS 2006
Q58a	How satisfied are you with the explanations the {doctor/other health professional} has given you about the results of your {treatment/care} at the selected place(s) in Q51a?<br><br>*-Record 99 if don't know/unsure*	0 = Very satisfied<br>1 = Satisfied<br>2 = Neither satisfied nor dissatisfied<br>3 = Dissatisfied<br>4 = Very d issatisfied	Categorical ordinal	Patient satisfaction of care services	SAPS 2006

For peer review only

For peer review only - http://bmjopen.bmj.com/site/about/guidelines.xhtml

Q59a	The {doctor/other health professional} at the selected place(s) in Q51a was very careful to check everything when examining you.<br><br>*-Record 99 if don't know/unsure*	0 = Strongly agree<br>1 = Agree<br>2 = Not sure<br>3 = Disagree<br>4 = Strongly disagree	Categorical ordinal	Patient satisfaction of care services	SAPS 2006
Q60a	At the selected place(s) in Q51a, how satisfied were you with the choices you had in decisions affecting your health care?<br><br>*-Record 99 if don't know/unsure*	0 = Very satisfied<br>1 = Satisfied<br>2 = Neither satisfied nor dissatisfied<br>3 = Dissatisfied<br>4 = Very dissatisfied	Categorical ordinal	Patient satisfaction of care services	SAPS 2006
Q61a	How much of the time did you feel respected by the {doctor/other health professional} at the selected place(s) in Q51a?<br><br>*-Record 99 if don't know/unsure*	0 = All of the time<br>1 = Most of the time<br>2 = About half the time<br>3 = Some of the time<br>4 = None of the time	Categorical ordinal	Patient satisfaction of care services	SAPS 2006
Q62a	At the selected place(s) in Q51a, the time you had with the {doctor/other health professional} was too short.<br><br>*-Record 99 if don't know/unsure*	0 = Strongly agree<br>1 = Agree<br>2 = Not sure<br>3 = Disagree<br>4 = Strongly disagree	Categorical ordinal	Patient satisfaction of care services	SAPS 2006
Q63a	Are you satisfied with the care you received in the selected place(s) in Q51a?<br><br>*-Record 99 if don't know/unsure*	0 = Very satisfied<br>1 = Satisfied<br>2 = Neither satisfied nor dissatisfied<br>3 = Dissatisfied<br>4 = Very dissatisfied	Categorical ordinal	Patient satisfaction of care services	SAPS 2006

For peer review only - http://bmjopen.bmj.com/site/about/guidelines.xhtml

Q64a	Would you recommend the selected place(s) in Q51a to others?<br><br>*-Record 99 if don't know/unsure*	1 = Not recommend<br>2 = Recommend with reservations<br>3 = Recommend<br>4 = Highly recommend	Categorical ordinal	Patient satisfaction of care services	Opinion
Q65a	Have you ever been told by a doctor that you have eyes problems?<br><br>*-Record 99 if don't know/unsure*	0 = No<br>1 = Yes	Quantitative discrete	Complication-HT	Cambodia national guideline
Q66a	Have you ever been told by a doctor that you have kidney problems?<br><br>*-Record 99 if don't know/unsure*	0 = No<br>1 = Yes	Quantitative discrete	Complication-HT	Cambodia national guideline
Q67a	Have you ever been told by a doctor that you have lost the sensation of your peripheral membrane, such as foot, hand, arm?<br><br>*-Record 99 if don't know/unsure*	0 = No<br>1 = Yes	Quantitative discrete	Complication-HT	Cambodia national guideline
	In **the past 3 months**, besides seeking medical advice or treatment for your hypertensive condition, have you sought medical treatment or advice for other illnesses or conditions?<br><br>If No, go to Section 4.	0 = No<br>1 = Yes			

For peer review only - http://bmjopen.bmj.com/site/about/guidelines.xhtml

Q24	Where did you seek medical advice or treatment for that illness in the past 3 months?<br><br>*-More than one answer can be selected.*<br>*-Data collectors can use probes to help respondents determine the types of health facilities in the Response Column.*<br>*-Record 99 if don't know and 88 if refuse*	1= National hospital (PP)<br>2= Provincial hospital (RH)<br>3= District hospital (RH)<br>4= Health centre<br>5= Health post<br>6= Provincial rehabilitation centre (PRC) or Community-based rehabilitation (CBR)<br>7= Other public; specify:<br>8= Private hospital<br>9= Private clinic<br>10= Private pharmacy<br>11= Home/Office of trained health worker/nurse<br>12= Visit of trained health worker/nurse<br>13= Other private medical; specify:<br>14= Shop selling drugs/market<br>15= Kru Khmer/ Magician<br>16= Monk/religious leader<br>17= Traditional birth attendant<br>18= Oversee medical service<br>19= Other; specify:	Categorical Nominal	Care provider_3M	CDHS and GACD book
From Q25-Q39, it is a set of questions that are asked following choices selected in Q24. If 2 or 3 choices were selected in Q24, Q25-Q39 would appear 2 or 3 times, accordingly.					

For peer review only - http://bmjopen.bmj.com/site/about/guidelines.xhtml

Q25	How many times did you visit the selected place**(s)** in Q24 in the past three months?	________ times	Quantitative discrete	Number of visits	GACD
Q26	How much in total was spent on the treatment at the selected place(s) in Q24?<br><br>-*Record 99 if don't know and 88 if refuse*	0 = free/no cost<br>1 = in kind<br>2 = ________Riels	-Categorical nominal<br>-Quantitative Continuous	Cost of treatment	CDHS
Q27	How did you pay for the treatment cost at the selected place(s) in Q24?<br><br>-*Record 99 if don't know and 88 if refuse*	1= Health Equity Fund<br>2= Voucher<br>3= Fee Exemption<br>4= NGO<br>5= National Social Security Fund<br>6= Community-Based Health Insurance<br>7= Health Insurance through Employer<br>8= Other Privately Purchased Commercial Health Insurance<br>9= Wage/income<br>10= Loan/ Ton Tin<br>11= Sale of Assets<br>12= Gift from Relative<br>13= Savings<br>14= Other	-Categorical nominal	Payment method	GADC adapted to context specific

For peer review only - http://bmjopen.bmj.com/site/about/guidelines.xhtml

Q28	How much in total was spent on transport to go to and return from the selected place(s) in Q24?<br><br>*-Record 99 if don't know and 88 if refuse*	0 = free/no cost<br>1 = in kind<br>2 = ________Riels	-Categorical nominal<br>-Quantitative Continuous	Cost of transport	CDHS
Q29	On average how many hours do you spend to get treatment/advices from the selected place(s) in Q24?<br><br>*-Record 99 if don't know and 88 if refuse*	________Hours	Quantitative continuous	Time spending	GACD book (Adapted)
Q30	How satisfied are you with the effect of your {treatment/care} at the selected place(s) in Q24?<br><br>*-Record 99 if don't know/unsure*	0 = Very satisfied<br>1 = Satisfied<br>2 = Neither satisfied nor dissatisfied<br>3 = Dissatisfied<br>4 = Very dissatisfied	Categorical ordinal	Patient satisfaction of care services	SAPS 2006
Q31	How satisfied are you with the explanations the {doctor/other health professional} has given you about the results of your {treatment/care} at the selected place(s) in Q24?<br><br>*-Record 99 if don't know/unsure*	0 = Very satisfied<br>1 = Satisfied<br>2 = Neither satisfied nor dissatisfied<br>3 = Dissatisfied<br>4 = Very dissatisfied	Categorical ordinal	Patient satisfaction of care services	SAPS 2006
Q32	The {doctor/other health professional} at the selected place(s) in Q24 was very careful to check everything when examining you.<br><br>*-Record 99 if don't know/unsure*	0 = Strongly agree<br>1 = Agree<br>2 = Not sure<br>3 = Disagree<br>4 = Strongly disagree	Categorical ordinal	Patient satisfaction of care services	SAPS 2006

For peer review only - http://bmjopen.bmj.com/site/about/guidelines.xhtml

Q33	At the selected place(s) in Q24, how satisfied were you with the choices you had in decisions affecting your health care?<br><br>*-Record 99 if don't know/unsure*	0 = Very satisfied<br>1 = Satisfied<br>2 = Neither s atisfied nor dissatisfied<br>3 = Dissatisfied<br>4 = Very dissatisfied	Categorical ordinal	Patien t satisfa ction of care servic es	SAPS 2006
Q34	How much of the time did you feel respected by the {doctor/other health professional} at the selected place(s) in Q24?<br><br>*-Record 99 if don't know/unsure*	0 = All of the time<br>1 = Most of the time<br>2 = About half the time<br>3 = Some of the time<br>4 = None of the time	Categorical ordinal	Patien t satisfa ction of care servic es	SAPS 2006
Q35	At the selected place(s) in Q24, the time you had with the {doctor/other health professional} was too short.<br><br>*-Record 99 if don't know/unsure*	0 = Strongly agree<br>1 = Agree<br>2 = Not sure<br>3 = Disagree<br>4 = Strongly disagree	Categorical ordinal	Patien t satisfa ction of care servic es	SAPS 2006
Q36	Are you satisfied with the care you received in the selected place(s) in Q24?<br><br>*-Record 99 if don't know/unsure*	0 = Very satisfied<br>1 = Satisfied<br>2 = Neither s atisfied nor dissatisfied<br>3 = Dissatisfied<br>4 = Very dissatisfied	Categorical ordinal	Patien t satisfa ction of care servic es	SAPS 2006
Q37	Did you get your blood pressure measured at the selected place(s) in Q24?<br><br>*-Record 99 if don't know/unsure*	0 = No<br>1 = Yes	Categorical variable	Acces s to blood pressu re testing	Opinion

For peer review only - http://bmjopen.bmj.com/site/about/guidelines.xhtml

Q38	Did you get your blood glucose tested at the selected place(s) in Q24?<br><br>*-Record 99 if don't know/unsure*	0 = No<br>1 = Yes	Categorical variable	Access to blood glucose testing	Opinion
Q39	Would you recommend the selected place(s) in Q24 to others?<br><br>*-Record 99 if don't know/unsure*	1 = Not recommended<br>2 = Recommend with reservations<br>3 = Recommend<br>4 = Highly recommend	Categorical ordinal	Patient satisfaction of care services	Opinion

peer review only

For peer review only - http://bmjopen.bmj.com/site/about/guidelines.xhtml

SECTION 3b: HEALTH CARE UTILIZATION FOR DIABETES					
**Q.N**	**Description and Questions**	**Response**	**Type of Variable**	**Field**	**Ref:**
Q43b	How long have you lived with diabetes?<br><br>*-Record 99 if don't know/unsure and 88 if refuse*<br>*-Less than a year is rounded up to one year*<br>*-Standard rounded up formula is applied.*	________Years	Quantitative concrete	Duration DM	Opinion
Q44b	Where were you first diagnosed as having diabetes?<br><br>*-Record 99 if don't know and 88 if refuse*	1= National hospital (PP)<br>2= Provincial hospital (RH)<br>3= District hospital (RH)<br>4= Health centre<br>5= Health post<br>6= Provincial rehabilitation centre (PRC) or Community-based rehabilitation (CBR)<br>7= Other public; specify:<br>8= Private hospital<br>9= Private clinic<br>10= Private pharmacy<br>11= Home/Office of trained health worker/nurse<br>12= Visit of trained health worker/nurse<br>13= Other private medical; specify:<br>14= Shop selling drugs/market<br>15= Kru Khmer/ Magician	Categorical Nominal	Diagnosis DM (CoC)	Opinion

For peer review only - http://bmjopen.bmj.com/site/about/guidelines.xhtml

		16= Monk/religious leader<br>17= Traditional birth attendant<br>18= Oversee medical service<br>19= MoPoTsyo<br>20= Other; specify:			
Q45b	Where did you first seek advice or treatment for diabetes after being diagnosed?<br><br>*-Record 99 if don't know and 88 if refuse*	1= National hospital (PP)<br>2= Provincial hospital (RH)<br>3= District hospital (RH)<br>4= Health centre<br>5= Health post<br>6= Provincial rehabilitation centre (PRC) or Community-based rehabilitation (CBR)<br>7= Other public; specify:<br>8= Private hospital<br>9= Private clinic<br>10= Private pharmacy<br>11= Home/Office of trained health worker/nurse<br>12= Visit of trained health worker/nurse<br>13= Other private medical; specify:<br>14= Shop selling drugs/market<br>15= Kru Khmer/ Magician<br>16= Monk/religious leader<br>17= Traditional birth attendant	Categorical Nominal	Link to care DM (CoC)	CDHS (Adapted for disease specific)

For peer review only - http://bmjopen.bmj.com/site/about/guidelines.xhtml

		18= Oversee medical service<br>19= MoPoTsyo<br>20= Other; specify:			
Q46b	Did you go to other places for follow up treatment/care for your diabetes conditions?<br><br>*-Record 88 if refuse*<br>*-If NO, please skip Q51b*	0 = No<br>1 = Yes	Categorical binary	Trust DM	CDHS
Q47b	If yes to Q46b, where else did you go to get follow up treatment/care for your diabetes conditions?	1= National hospital (PP)<br>2= Provincial hospital (RH)<br>3= District hospital (RH)<br>4= Health centre<br>5= Health post<br>6= Provincial rehabilitation centre (PRC) or Community-based rehabilitation (CBR)<br>7= Other public; specify:<br>8= Private hospital<br>9= Private clinic<br>10= Private pharmacy<br>11= Home/Office of trained health worker/nurse<br>12= Visit of trained health worker/nurse<br>13= Other private medical; specify:<br>14= Shop selling drugs/market	Categorical Nominal	Link to care DM (CoC)	CDHS (Adapted for disease specific)

For peer review only - http://bmjopen.bmj.com/site/about/guidelines.xhtml

		15= Kru Khmer/ Magician<br>16= Monk/religious leader<br>17= Traditional birth attendant<br>18= Oversee medical service<br>19= MoPoTsyo<br>20= Other; specify:			
Q48b	Did you get treatment/care for your diabetes conditions in the past 12 months?<br><br>*-Record 88 if refuse*<br>*-If NO, please skip Q49b-65b*	0 = No<br>1 = Yes	Categorical binary	Retain in care DM (CoC)	CDHS (Adapted for disease specific)
Q49b	Are you currently receiving any of the following treatment/advices for your diabetes conditions prescribed by a doctor or other health care worker?<br><br>Q. 49b.1. Insulin [____]<br>Q. 49b.2. Drugs (medication) that you have taken in the past two weeks [____]<br>Q. 49b.3. Special prescribed diet [____]<br>Q. 49b.4. Advice or treatment to lose weight [____]<br>Q. 49b.5. Advice or treatment to stop smoking<br>Q. 49b.6. Advice to start or do more physical exercise	0 = No 1= Yes	Categorical ordinal	In treatm ent for DM (CoC)	STEPS Survey
Q50b	Have you had your blood glucose measured in the past 12 months?<br><br>*-Record 99 if don't know and 88 if refuse*	0 = No 1= Yes	Categorical ordinal	In treatm ent for DM (CoC)	Veerle's suggestion

For peer review only - http://bmjopen.bmj.com/site/about/guidelines.xhtml

Q51b	Have you had your HbA1c tested in the past 12 months?<br><br>*-Record 99 if don't know and 88 if refuse*	0 = No 1= Yes	Categorical ordinal	In treatment for DM (CoC)	Veerle's suggestion
Q52b	Where did you seek medical advice or treatment for illness in the **past 3 months**?<br><br>***-More than one answer can be selected.***<br>*-Data collectors can use probes to help respondents determine the types of health facilities in the Response Column.*<br>*-Record 99 if don't know and 88 if refuse*<br>*-If 21=Nowhere, go to Q66b.*	1= National hospital (PP)<br>2= Provincial hospital (RH)<br>3= District hospital (RH)<br>4= Health centre<br>5= Health post<br>6= Provincial rehabilitation centre (PRC) or Community-based rehabilitation (CBR)<br>7= Other public; specify:<br>8= Private hospital<br>9= Private clinic<br>10= Private pharmacy<br>11= Home/Office of trained health worker/nurse<br>12= Visit of trained health worker/nurse<br>13= Other private medical; specify:<br>14= Shop selling drugs/market<br>15= Kru Khmer/ Magician<br>16= Monk/religious leader<br>17= Traditional birth attendant<br>18= Oversee medical service	Categorical Nominal	Care provider_3M	CDHS and GACD book

For peer review only - http://bmjopen.bmj.com/site/about/guidelines.xhtml

		19= MoPoTsyo<br>20= Other; specify:<br>21= No where			
From Q53b-Q65b, it is a set of questions that are asked following choices selected in Q52b. If 2 or 3 choices were selected in Q52b, Q53b-Q65b would appear 2 or 3 times, accordingly.					
Q53b	How many times did you visit the selected place**(s)** in Q52b in the past three months?	________ times	Quantitative discrete	Number of visits	GACD
Q54b	How much in total was spent on the treatment at the selected place(s) in Q52b?<br><br>*-Record 99 if don't know and 88 if refuse*	0 = free/no cost<br>1 = in kind<br>2 = _________Riels OR _________ USD	-Categorical nominal<br>-<br>Quantitative Continuous	Cost of treatment	CDHS
Q55b	How did you pay for the treatment cost at the selected place(s) in Q52b?<br><br>*-Record 99 if don't know and 88 if refuse*	1= Health Equity Fund<br>2= Voucher<br>3= Fee Exemption<br>4= NGO<br>5= National Social Security Fund<br>6= Community-Based Health Insurance<br>7= Health Insurance through Employer<br>8= Other Privately	-Categorical nominal	Payment method	GADC adapted to context specific

For peer review only - http://bmjopen.bmj.com/site/about/guidelines.xhtml

		Purchased Commercial Health Insurance<br>9= Wage/income<br>10= Loan/ Ton Tin<br>11= Sale of Assets<br>12= Gift from Relative<br>13= Savings<br>14= Other			
Q56b	How much in total was spent on transport to go to and return from the selected place(s) in Q52b?<br><br>*-Record 99 if don't know and 88 if refuse*	0 = free/no cost<br>1 = in kind<br>2 = ________Riels<br>OR<br>________ USD	-Categorical nominal<br>-<br>Quantitative Continuous	Cost of transp ort	CDHS
Q57b	On average how many hours do you spend to get treatment/advices from the selected place(s) in Q52b?<br><br>*-Record 99 if don't know and 88 if refuse*	________Hours	Quantitative continuous	Time spendi ng	GACD book (Adapted)
Q58b	How satisfied are you with the effect of your {treatment/care} at the selected place(s) in Q52b?<br><br>*-Record 99 if don't know/unsure*	0 = Very satisfied<br>1 = Satisfied<br>2 = Neither satisfied nor dissatisfied<br>3 = Dissatisfied<br>4 = Very dissatisfied	Categorical ordinal	Patien t satisfa ction of care servic e	SAPS 2006
Q59b	How satisfied are you with the explanations the {doctor/other health professional} has given you about the results of your {treatment/care} at the selected place(s) in Q52b?<br><br>*-Record 99 if don't know/unsure*	0 = Very satisfied<br>1 = Satisfied<br>2 = Neither satisfied nor dissatisfied<br>3 = Dissatisfied<br>4 = Very d issatisfied	Categorical ordinal	Patien t satisfa ction of care servic e	SAPS 2006

For peer review only - http://bmjopen.bmj.com/site/about/guidelines.xhtml

Q60b	The {doctor/other health professional} at the selected place(s) in Q52b was very careful to check everything when examining you.<br><br>*-Record 99 if don't know/unsure*	0 = Strongly agree<br>1 = Agree<br>2 = Not sure<br>3 = Disagree<br>4 = Strongly disagree	Categorical ordinal	Patient satisfaction of care service	SAPS 2006
Q61b	At the selected place(s) in Q52b, how satisfied were you with the choices you had in decisions affecting your health care?<br><br>*-Record 99 if don't know/unsure*	0 = Very satisfied<br>1 = Satisfied<br>2 = Neither satisfied nor dissatisfied<br>3 = Dissatisfied<br>4 = Very dissatisfied	Categorical ordinal	Patient satisfaction of care service	SAPS 2006
Q62b	How much of the time did you feel respected by the {doctor/other health professional} at the selected place(s) in Q52b?<br><br>*-Record 99 if don't know/unsure*	0 = All of the time<br>1 = Most of the time<br>2 = About half the time<br>3 = Some of the time<br>4 = None of the time	Categorical ordinal	Patient satisfaction of care service	SAPS 2006
Q63b	At the selected place(s) in Q52b, the time you had with the {doctor/other health professional} was too short.<br><br>*-Record 99 if don't know/unsure*	0 = Strongly agree<br>1 = Agree<br>2 = Not sure<br>3 = Disagree<br>4 = Strongly disagree	Categorical ordinal	Patient satisfaction of care service	SAPS 2006
Q64b	Are you satisfied with the care you received in the selected place(s) in Q52b?<br><br>*-Record 99 if don't know/unsure*	0 = Very satisfied<br>1 = Satisfied<br>2 = Neither satisfied nor dissatisfied<br>3 = Dissatisfied<br>4 = Very dissatisfied	Categorical ordinal	Patient satisfaction of care service	SAPS 2006

For peer review only - http://bmjopen.bmj.com/site/about/guidelines.xhtml

Q65b	Would you recommend the selected place(s) in Q52b to others?<br><br>*-Record 99 if don't know/unsure*	1 = Not recommended<br>2 = Recommend with reservations<br>3 = Recommend<br>4 = Highly recommend	Categorical ordinal	Patient satisfaction of care services	Opinion
Q66b	Have you ever been told by a doctor that you have eyes problems?<br><br>*-Record 99 if don't know/unsure*	0 = No<br>1 = Yes	Quantitative discrete	Complication-DM	Cambodia national guideline
Q67b	Have you ever been told by a doctor that you have kidney problems?<br><br>*-Record 99 if don't know/unsure*	0 = No<br>1 = Yes	Quantitative discrete	Complication-DM	Cambodia national guideline
Q68b	Have you ever been told by a doctor that you have lost the sensation of your peripheral membrane, such as foot, hand, arm?<br><br>*-Record 99 if don't know/unsure*	0 = No<br>1 = Yes	Quantitative discrete	Complication-DM	Cambodia national guideline
	In **the past 3 months**, besides seeking medical advice or treatment for your diabetes condition, have you sought medical treatment or advice for other illnesses or conditions?<br><br>If No, go to Section 4.	0 = No<br>1 = Yes			

For peer review only - http://bmjopen.bmj.com/site/about/guidelines.xhtml

Q24	Where did you seek medical advice or treatment for that illness in the past 3 months?<br><br>*-More than one answer can be selected.*<br>*-Data collectors can use probes to help respondents determine the types of health facilities in the Response Column.*<br>*-Record 99 if don't know and 88 if refuse*	1= National hospital (PP)<br>2= Provincial hospital (RH)<br>3= District hospital (RH)<br>4= Health centre<br>5= Health post<br>6= Provincial rehabilitation centre (PRC) or Community-based rehabilitation (CBR)<br>7= Other public; specify:<br>8= Private hospital<br>9= Private clinic<br>10= Private pharmacy<br>11= Home/Office of trained health worker/nurse<br>12= Visit of trained health worker/nurse<br>13= Other private medical; specify:<br>14= Shop selling drugs/market<br>15= Kru Khmer/ Magician<br>16= Monk/religious leader<br>17= Traditional birth attendant<br>18= Oversee medical service<br>19= Other; specify:	Categorical Nominal	Care provider_3M	CDHS and GACD book
From Q25-Q39, it is a set of questions that are asked following choices selected in Q24. If 2 or 3 choices were selected in Q24, Q25-Q39 would appear 2 or 3 times, accordingly.					

For peer review only - http://bmjopen.bmj.com/site/about/guidelines.xhtml

Q25	How many times did you visit the selected place**(s)** in Q24 in the past three months?	_______ times	Quantitative discrete	Number of visits	GACD
Q26	How much in total was spent on the treatment at the selected place(s) in Q24?<br><br>-*Record 99 if don't know and 88 if refuse*	0 = free/no cost<br>1 = in kind<br>2 = ________Riels	-Categorical nominal<br>-Quantitative Continuous	Cost of treatment	CDHS
Q27	How did you pay for the treatment cost at the selected place(s) in Q24?<br><br>-*Record 99 if don't know and 88 if refuse*	1= Health Equity Fund<br>2= Voucher<br>3= Fee Exemption<br>4= NGO<br>5= National Social Security Fund<br>6= Community-Based Health Insurance<br>7= Health Insurance through Employer<br>8= Other Privately Purchased Commercial Health Insurance<br>9= Wage/income<br>10= Loan/ Ton Tin<br>11= Sale of Assets<br>12= Gift from Relative<br>13= Savings<br>14= Other	-Categorical nominal	Payment method	GADC adapted to context specific

For peer review only - http://bmjopen.bmj.com/site/about/guidelines.xhtml

Q28	How much in total was spent on transport to go to and return from the selected place(s) in Q24?<br><br>*-Record 99 if don't know and 88 if refuse*	0 = free/no cost<br>1 = in kind<br>2 = ________Riels	-Categorical nominal<br>-Quantitative Continuous	Cost of transport	CDHS
Q29	On average how many hours do you spend to get treatment/advices from the selected place(s) in Q24?<br><br>*-Record 99 if don't know and 88 if refuse*	________Hours	Quantitative continuous	Time spending	GACD book (Adapted)
Q30	How satisfied are you with the effect of your {treatment/care} at the selected place(s) in Q24?<br><br>*-Record 99 if don't know/unsure*	0 = Very satisfied<br>1 = Satisfied<br>2 = Neither satisfied nor dissatisfied<br>3 = Dissatisfied<br>4 = Very dissatisfied	Categorical ordinal	Patient satisfaction of care services	SAPS 2006
Q31	How satisfied are you with the explanations the {doctor/other health professional} has given you about the results of your {treatment/care} at the selected place(s) in Q24?<br><br>*-Record 99 if don't know/unsure*	0 = Very satisfied<br>1 = Satisfied<br>2 = Neither satisfied nor dissatisfied<br>3 = Dissatisfied<br>4 = Very dissatisfied	Categorical ordinal	Patient satisfaction of care services	SAPS 2006
Q32	The {doctor/other health professional} at the selected place(s) in Q24 was very careful to check everything when examining you.<br><br>*-Record 99 if don't know/unsure*	0 = Strongly agree<br>1 = Agree<br>2 = Not sure<br>3 = Disagree<br>4 = Strongly disagree	Categorical ordinal	Patient satisfaction of care services	SAPS 2006

For peer review only - http://bmjopen.bmj.com/site/about/guidelines.xhtml

Q33	At the selected place(s) in Q24, how satisfied were you with the choices you had in decisions affecting your health care?<br><br>*-Record 99 if don't know/unsure*	0 = Very satisfied<br>1 = Satisfied<br>2 = Neither s atisfied nor dissatisfied<br>3 = Dissatisfied<br>4 = Very dissatisfied	Categorical ordinal	Patien t satisfa ction of care servic es	SAPS 2006
Q34	How much of the time did you feel respected by the {doctor/other health professional} at the selected place(s) in Q24?<br><br>*-Record 99 if don't know/unsure*	0 = All of the time<br>1 = Most of the time<br>2 = About half the time<br>3 = Some of the time<br>4 = None of the time	Categorical ordinal	Patien t satisfa ction of care servic es	SAPS 2006
Q35	At the selected place(s) in Q24, the time you had with the {doctor/other health professional} was too short.<br><br>*-Record 99 if don't know/unsure*	0 = Strongly agree<br>1 = Agree<br>2 = Not sure<br>3 = Disagree<br>4 = Strongly disagree	Categorical ordinal	Patien t satisfa ction of care servic es	SAPS 2006
Q36	Are you satisfied with the care you received in the selected place(s) in Q24?<br><br>*-Record 99 if don't know/unsure*	0 = Very satisfied<br>1 = Satisfied<br>2 = Neither s atisfied nor dissatisfied<br>3 = Dissatisfied<br>4 = Very dissatisfied	Categorical ordinal	Patien t satisfa ction of care servic es	SAPS 2006
Q37	Did you get your blood pressure measured at the selected place(s) in Q24?<br><br>*-Record 99 if don't know/unsure*	0 = No<br>1 = Yes	Categorical variable	Acces s to blood pressu re testing	Opinion

For peer review only - http://bmjopen.bmj.com/site/about/guidelines.xhtml

Q38	Did you get your blood glucose tested at the selected place(s) in Q24?<br><br>*-Record 99 if don't know/unsure*	0 = No<br>1 = Yes	Categorical variable	Access to blood glucose testing	Opinion
Q39	Would you recommend the selected place(s) in Q24 to others?<br><br>*-Record 99 if don't know/unsure*	1 = Not recommended<br>2 = Recommend with reservations<br>3 = Recommend<br>4 = Highly recommend	Categorical ordinal	Patient satisfaction of care services	Opinion

peer review only

For peer review only - http://bmjopen.bmj.com/site/about/guidelines.xhtml

SECTION 3c: HEALTH CARE UTILIZATION FOR DIABETES AND HYPERTENSION					
**Q.N**	**Description and Questions**	**Response**	**Type of Variable**	**Field**	**Ref:**
Q43c	How long have you lived with diabetes?<br><br>*-Record 99 if don't know/unsure and 88 if refuse*<br>*-Less than a year is rounded up to one year*<br>*-Standard rounded up formula is applied.*	________Years	Quantitative concrete	Duration DM and HT	Opinion
Q44c	Where were you first diagnosed as having diabetes?<br><br>*-Record 99 if don't know and 88 if refuse*	1= National hospital (PP)<br>2= Provincial hospital (RH)<br>3= District hospital (RH)<br>4= Health centre<br>5= Health post<br>6= Provincial rehabilitation centre (PRC) or Community-based rehabilitation (CBR)<br>7= Other public; specify:<br>8= Private hospital<br>9= Private clinic<br>10= Private pharmacy<br>11= Home/Office of trained health worker/nurse<br>12= Visit of trained health worker/nurse<br>13= Other private medical; specify:<br>14= Shop selling drugs/market<br>15= Kru Khmer/ Magician	Categorical Nominal	Diagnosis DM (CoC)	Opinion

For peer review only - http://bmjopen.bmj.com/site/about/guidelines.xhtml

		16= Monk/religious leader<br>17= Traditional birth attendant<br>18= Oversee medical service<br>19= MoPoTsyo<br>20= Other; specify:			
Q45c	Where did you first seek advice or treatment for diabetes after being diagnosed?<br><br>*-Record 99 if don't know and 88 if refuse*	1= National hospital (PP)<br>2= Provincial hospital (RH)<br>3= District hospital (RH)<br>4= Health centre<br>5= Health post<br>6= Provincial rehabilitation centre (PRC) or Community-based rehabilitation (CBR)<br>7= Other public; specify:<br>8= Private hospital<br>9= Private clinic<br>10= Private pharmacy<br>11= Home/Office of trained health worker/nurse<br>12= Visit of trained health worker/nurse<br>13= Other private medical; specify:<br>14= Shop selling drugs/market<br>15= Kru Khmer/ Magician<br>16= Monk/religious leader<br>17= Traditional birth attendant	Categorical Nominal	Link to care DM (CoC)	CDHS (Adapted for disease specific)

For peer review only - http://bmjopen.bmj.com/site/about/guidelines.xhtml

		18= Oversee medical service<br>19= MoPoTsyo<br>20= Other; specify:			
Q46c	How long have you lived with hypertension?<br><br>*-Record 99 if don't know/unsure and 88 if refuse*<br>*-Less than a year is rounded up to one year*<br>*-Standard rounded up formula is applied.*	________Years	Quantitative concrete	Duration DM and HT	Opinion
Q47c	Where were you first diagnosed as having hypertension?<br><br>*-Record 99 if don't know and 88 if refuse*	1= National hospital (PP)<br>2= Provincial hospital (RH)<br>3= District hospital (RH)<br>4= Health centre<br>5= Health post<br>6= Provincial rehabilitation centre (PRC) or Community-based rehabilitation (CBR)<br>7= Other public; specify:<br>8= Private hospital<br>9= Private clinic<br>10= Private pharmacy<br>11= Home/Office of trained health worker/nurse<br>12= Visit of trained health worker/nurse<br>13= Other private medical; specify:<br>14= Shop selling drugs/market	Categorical Nominal	Diagnosis HT (CoC)	Opinion

For peer review only - http://bmjopen.bmj.com/site/about/guidelines.xhtml

		15= Kru Khmer/ Magician<br>16= Monk/religious leader<br>17= Traditional birth attendant<br>18= Oversee medical service<br>19= MoPoTsyo<br>20= Other; specify:			
Q48c	Where did you first seek advice or treatment for hypertension after being diagnosed?<br><br>*-Record 99 if don't know and 88 if refuse*	1= National hospital (PP)<br>2= Provincial hospital (RH)<br>3= District hospital (RH)<br>4= Health centre<br>5= Health post<br>6= Provincial rehabilitation centre (PRC) or Community-based rehabilitation (CBR)<br>7= Other public; specify:<br>8= Private hospital<br>9= Private clinic<br>10= Private pharmacy<br>11= Home/Office of trained health worker/nurse<br>12= Visit of trained health worker/nurse<br>13= Other private medical; specify:<br>14= Shop selling drugs/market<br>15= Kru Khmer/ Magician<br>16= Monk/religious leader	Categorical Nominal	Link to care HT (CoC)	CDHS (Adapted for disease specific)

For peer review only - http://bmjopen.bmj.com/site/about/guidelines.xhtml

		17= Traditional birth attendant<br>18= Oversee medical service<br>19= MoPoTsyo<br>20= Other; specify:			
Q49c	Did you go to other places for follow up treatment/care for your hypertensive and diabetes conditions?<br><br>*-Record 88 if refuse*<br>*-If NO, please skip Q50c*	0 = No<br>1 = Yes	Categorical binary	Trust DM and HT	CDHS
Q50c	If yes to Q49c, where else did you go to get follow up treatment/care for both conditions?	1= National hospital (PP)<br>2= Provincial hospital (RH)<br>3= District hospital (RH)<br>4= Health centre<br>5= Health post<br>6= Provincial rehabilitation centre (PRC) or Community-based rehabilitation (CBR)<br>7= Other public; specify:<br>8= Private hospital<br>9= Private clinic<br>10= Private pharmacy<br>11= Home/Office of trained health worker/nurse<br>12= Visit of trained health worker/nurse<br>13= Other private medical; specify:<br>14= Shop selling drugs/market	Categorical Nominal	Link to care DM and HT (CoC)	CDHS (Adapted for disease specific)

For peer review only - http://bmjopen.bmj.com/site/about/guidelines.xhtml

		15= Kru Khmer/ Magician<br>16= Monk/religious leader<br>17= Traditional birth attendant<br>18= Oversee medical service<br>19= MoPoTsyo<br>20= Other; specify:			
Q51c	Did you get treatment/care for both conditions in the past 12 months?<br><br>*-Record 88 if refuse*<br>*-If NO, please skip Q52c-69c*	0 = No<br>1 = Yes	Categorical binary	Retain in care DM and HT (CoC)	CDHS (Adapted for disease specific)
Q52c	Are you currently receiving any of the following treatment/advices for both conditions prescribed by a doctor or other health care worker?<br><br>Q. 52c.1. Insulin [____]<br>Q. 52c.2. Drugs (medication) that you have taken in the past two weeks [____]<br>Q. 52c.3. Special prescribed diet [____]<br>Q. 52c.4. Advice or treatment to lose weight [____]<br>Q. 52c.5. Advice or treatment to stop smoking<br>Q. 52c.6. Advice to reduce salt intake<br>Q. 52c.7. Advice to start or do more physical exercise	0 = No 1= Yes	Categorical ordinal	In treatment for DM and HT (CoC)	STEPS Survey
Q53c	Have you had your blood glucose measured in the past 12 months?<br><br>*-Record 99 if don't know and 88 if refuse*	0 = No 1= Yes	Categorical ordinal	In treatment for DM (CoC)	Veerle's suggestion

For peer review only - http://bmjopen.bmj.com/site/about/guidelines.xhtml

Q54c	Have you had your HbA1c tested in the past 12 months?<br><br>*-Record 99 if don't know and 88 if refuse*	0 = No 1= Yes	Categorical ordinal	In treatment for DM (CoC)	Veerle's suggestion
Q55c	Have you had your blood cholesterol measured in the past 12 months?<br><br>*-Record 99 if don't know and 88 if refuse*	0 = No 1= Yes	Categorical ordinal	In treatment for HT (CoC)	Veerle's suggestion
Q56c	Where did you seek medical advice or treatment for your conditions in **the past 3 months?**<br><br>***-More than one answer can be selected**.*<br>*-Data collectors can use probes to help respondents determine the types of health facilities in the Response Column.*<br>*-Record 99 if don't know and 88 if refuse*<br>*-If 21=Nowhere, go to Q70c.*	1= National hospital (PP)<br>2= Provincial hospital (RH)<br>3= District hospital (RH)<br>4= Health centre<br>5= Health post<br>6= Provincial rehabilitation centre (PRC) or Community-based rehabilitation (CBR)<br>7= Other public; specify:<br>8= Private hospital<br>9= Private clinic<br>10= Private pharmacy<br>11= Home/Office of trained health worker/nurse<br>12= Visit of trained health worker/nurse<br>13= Other private medical; specify:<br>14= Shop selling drugs/market	Categorical Nominal	Care provider_3M	CDHS and GACD book

For peer review only

For peer review only - http://bmjopen.bmj.com/site/about/guidelines.xhtml

		15= Kru Khmer/ Magician<br>16= Monk/religious leader<br>17= Traditional birth attendant<br>18= Oversee medical service<br>19= MoPoTsyo<br>20= Other; specify:<br>21= Nowhere			
From Q57c-Q69c, it is a set of questions that are asked following choices selected in Q56c. If 2 or 3 choices were selected in Q56c, Q57c-Q69c would appear 2 or 3 times, accordingly.					
Q57c	How many times did you visit the selected place**(s)** in Q56c in the past three months?	________ times	Quantitative discrete	Numb er of visits	GACD
Q58c	How much in total was spent on the treatment at the selected place(s) in Q56c?<br><br>*-Record 99 if don't know and 88 if refuse*	0 = free/no cost<br>1 = in kind<br>2 = _________Riels OR _________ USD	-Categorical nominal<br>-<br>Quantitative Continuous	Cost of treatm ent	CDHS
Q59c	How did you pay for the treatment cost at the selected place(s) in Q56c?<br><br>*-Record 99 if don't know and 88 if refuse*	1= Health Equity Fund<br>2= Voucher<br>3= Fee Exemption<br>4= NGO<br>5= National Social Security Fund<br>6= Community-Based Health Insurance<br>7= Health Insurance through Employer<br>8= Other Privately	-Categorical nominal	Paym ent metho d	GADC adapted to context specific

For peer review only - http://bmjopen.bmj.com/site/about/guidelines.xhtml

		Purchased Commercial Health Insurance<br>9= Wage/income<br>10= Loan/ Ton Tin<br>11= Sale of Assets<br>12= Gift from Relative<br>13= Savings<br>14= Other			
Q60c	How much in total was spent on transport to go to and return from the selected place(s) in Q56c?<br><br>*-Record 99 if don't know and 88 if refuse*	0 = free/no cost<br>1 = in kind<br>2 = ________Riels<br>OR<br>________ USD	-Categorical nominal<br>-<br>Quantitative Continuous	Cost of transp ort	CDHS
Q61c	On average how many hours do you spend to get treatment/advices from the selected place(s) in Q56c?<br><br>*-Record 99 if don't know and 88 if refuse*	________Hours	Quantitative continuous	Time spendi ng	GACD book (Adapted)
Q62c	How satisfied are you with the effect of your {treatment/care} at the selected place(s) in Q56c?<br><br>*-Record 99 if don't know/unsure*	0 = Very satisfied<br>1 = Satisfied<br>2 = Neither satisfied nor dissatisfied<br>3 = Dissatisfied<br>4 = Very dissatisfied	Categorical ordinal	Patien t satisfa ction of care servic e	SAPS 2006
Q63c	How satisfied are you with the explanations the {doctor/other health professional} has given you about the results of your {treatment/care} at the selected place(s) in Q56c?<br><br>*-Record 99 if don't know/unsure*	0 = Very satisfied<br>1 = Satisfied<br>2 = Neither satisfied nor dissatisfied<br>3 = Dissatisfied<br>4 = Very d issatisfied	Categorical ordinal	Patien t satisfa ction of care servic e	SAPS 2006

For peer review only - http://bmjopen.bmj.com/site/about/guidelines.xhtml

Q64c	The {doctor/other health professional} at the selected place(s) in Q56c was very careful to check everything when examining you.<br><br>*-Record 99 if don't know/unsure*	0 = Strongly agree<br>1 = Agree<br>2 = Not sure<br>3 = Disagree<br>4 = Strongly disagree	Categorical ordinal	Patient satisfaction of care service	SAPS 2006
Q65c	At the selected place(s) in Q56c, how satisfied were you with the choices you had in decisions affecting your health care?<br><br>*-Record 99 if don't know/unsure*	0 = Very satisfied<br>1 = Satisfied<br>2 = Neither satisfied nor dissatisfied<br>3 = Dissatisfied<br>4 = Very dissatisfied	Categorical ordinal	Patient satisfaction of care service	SAPS 2006
Q66c	How much of the time did you feel respected by the {doctor/other health professional} at the selected place(s) in Q56c?<br><br>*-Record 99 if don't know/unsure*	0 = All of the time<br>1 = Most of the time<br>2 = About half the time<br>3 = Some of the time<br>4 = None of the time	Categorical ordinal	Patient satisfaction of care service	SAPS 2006
Q67c	At the selected place(s) in Q56c, the time you had with the {doctor/other health professional} was too short.<br><br>*-Record 99 if don't know/unsure*	0 = Strongly agree<br>1 = Agree<br>2 = Not sure<br>3 = Disagree<br>4 = Strongly disagree	Categorical ordinal	Patient satisfaction of care service	SAPS 2006
Q68c	Are you satisfied with the care you received in the selected place(s) in Q56c?<br><br>*-Record 99 if don't know/unsure*	0 = Very satisfied<br>1 = Satisfied<br>2 = Neither satisfied nor dissatisfied<br>3 = Dissatisfied<br>4 = Very dissatisfied	Categorical ordinal	Patient satisfaction of care service	SAPS 2006

For peer review only - http://bmjopen.bmj.com/site/about/guidelines.xhtml

Q69c	Would you recommend the selected place(s) in Q56c to others?<br><br>*-Record 99 if don't know/unsure*	1 = Not recommended<br>2 = Recommend with reservations<br>3 = Recommend<br>4 = Highly recommend	Categorical ordinal	Patient satisfaction of care services	Opinion
Q70c	Have you ever been told by a doctor that you have eyes problems?<br><br>*-Record 99 if don't know/unsure*	0 = No<br>1 = Yes	Quantitative discrete	Complication-DM and HT	Cambodia national guideline
Q71c	Have you ever been told by a doctor that you have kidney problems?<br><br>*-Record 99 if don't know/unsure*	0 = No<br>1 = Yes	Quantitative discrete	Complication-DM and HT	Cambodia national guideline
Q72c	Have you ever been told by a doctor that you have lost the sensation of your peripheral membrane, such as foot, hand, arm?<br><br>*-Record 99 if don't know/unsure*	0 = No<br>1 = Yes	Quantitative discrete	Complication-DM and HT	Cambodia national guideline
	In **the past 3 months**, besides seeking medical advice or treatment for your hypertensive and diabetes condition, have you sought medical treatment or advice for other illnesses or conditions?<br><br>If No, go to Section 4.	0 = No<br>1 = Yes			

For peer review only - http://bmjopen.bmj.com/site/about/guidelines.xhtml

Q24	Where did you seek medical advice or treatment for that illness in the past 3 months?<br><br>*-More than one answer can be selected.*<br>*-Data collectors can use probes to help respondents determine the types of health facilities in the Response Column.*<br>*-Record 99 if don't know and 88 if refuse*	1= National hospital (PP)<br>2= Provincial hospital (RH)<br>3= District hospital (RH)<br>4= Health centre<br>5= Health post<br>6= Provincial rehabilitation centre (PRC) or Community-based rehabilitation (CBR)<br>7= Other public; specify:<br>8= Private hospital<br>9= Private clinic<br>10= Private pharmacy<br>11= Home/Office of trained health worker/nurse<br>12= Visit of trained health worker/nurse<br>13= Other private medical; specify:<br>14= Shop selling drugs/market<br>15= Kru Khmer/ Magician<br>16= Monk/religious leader<br>17= Traditional birth attendant<br>18= Oversee medical service<br>19= Other; specify:	Categorical Nominal	Care provider_3M	CDHS and GACD book
From Q25-Q39, it is a set of questions that are asked following choices selected in Q24. If 2 or 3 choices were selected in Q24, Q25-Q39 would appear 2 or 3 times, accordingly.					

Q25	How many times did you visit the selected place**(s)** in Q24 in the past three months?	_______ times	Quantitative discrete	Number of visits	GACD
Q26	How much in total was spent on the treatment at the selected place(s) in Q24?<br><br>*-Record 99 if don't know and 88 if refuse*	0 = free/no cost<br>1 = in kind<br>2 = ________Riels	-Categorical nominal<br>-Quantitative Continuous	Cost of treatment	CDHS
Q27	How did you pay for the treatment cost at the selected place(s) in Q24?<br><br>*-Record 99 if don't know and 88 if refuse*	1= Health Equity Fund<br>2= Voucher<br>3= Fee Exemption<br>4= NGO<br>5= National Social Security Fund<br>6= Community-Based Health Insurance<br>7= Health Insurance through Employer<br>8= Other Privately Purchased Commercial Health Insurance<br>9= Wage/income<br>10= Loan/ Ton Tin<br>11= Sale of Assets<br>12= Gift from Relative<br>13= Savings<br>14= Other	-Categorical nominal	Payment method	GADC adapted to context specific

For peer review only - http://bmjopen.bmj.com/site/about/guidelines.xhtml

Q28	How much in total was spent on transport to go to and return from the selected place(s) in Q24?<br><br>*-Record 99 if don't know and 88 if refuse*	0 = free/no cost<br>1 = in kind<br>2 = ________Riels	-Categorical nominal<br>-Quantitative Continuous	Cost of transport	CDHS
Q29	On average how many hours do you spend to get treatment/advices from the selected place(s) in Q24?<br><br>*-Record 99 if don't know and 88 if refuse*	________Hours	Quantitative continuous	Time spending	GACD book (Adapted)
Q30	How satisfied are you with the effect of your {treatment/care} at the selected place(s) in Q24?<br><br>*-Record 99 if don't know/unsure*	0 = Very satisfied<br>1 = Satisfied<br>2 = Neither satisfied nor dissatisfied<br>3 = Dissatisfied<br>4 = Very dissatisfied	Categorical ordinal	Patient satisfaction of care services	SAPS 2006
Q31	How satisfied are you with the explanations the {doctor/other health professional} has given you about the results of your {treatment/care} at the selected place(s) in Q24?<br><br>*-Record 99 if don't know/unsure*	0 = Very satisfied<br>1 = Satisfied<br>2 = Neither satisfied nor dissatisfied<br>3 = Dissatisfied<br>4 = Very dissatisfied	Categorical ordinal	Patient satisfaction of care services	SAPS 2006
Q32	The {doctor/other health professional} at the selected place(s) in Q24 was very careful to check everything when examining you.<br><br>*-Record 99 if don't know/unsure*	0 = Strongly agree<br>1 = Agree<br>2 = Not sure<br>3 = Disagree<br>4 = Strongly disagree	Categorical ordinal	Patient satisfaction of care services	SAPS 2006

For peer review only - http://bmjopen.bmj.com/site/about/guidelines.xhtml

Q33	At the selected place(s) in Q24, how satisfied were you with the choices you had in decisions affecting your health care?<br><br>*-Record 99 if don't know/unsure*	0 = Very satisfied<br>1 = Satisfied<br>2 = Neither s atisfied nor dissatisfied<br>3 = Dissatisfied<br>4 = Very dissatisfied	Categorical ordinal	Patien t satisfa ction of care servic es	SAPS 2006
Q34	How much of the time did you feel respected by the {doctor/other health professional} at the selected place(s) in Q24?<br><br>*-Record 99 if don't know/unsure*	0 = All of the time<br>1 = Most of the time<br>2 = About half the time<br>3 = Some of the time<br>4 = None of the time	Categorical ordinal	Patien t satisfa ction of care servic es	SAPS 2006
Q35	At the selected place(s) in Q24, the time you had with the {doctor/other health professional} was too short.<br><br>*-Record 99 if don't know/unsure*	0 = Strongly agree<br>1 = Agree<br>2 = Not sure<br>3 = Disagree<br>4 = Strongly disagree	Categorical ordinal	Patien t satisfa ction of care servic es	SAPS 2006
Q36	Are you satisfied with the care you received in the selected place(s) in Q24?<br><br>*-Record 99 if don't know/unsure*	0 = Very satisfied<br>1 = Satisfied<br>2 = Neither s atisfied nor dissatisfied<br>3 = Dissatisfied<br>4 = Very dissatisfied	Categorical ordinal	Patien t satisfa ction of care servic es	SAPS 2006
Q37	Did you get your blood pressure measured at the selected place(s) in Q24?<br><br>*-Record 99 if don't know/unsure*	0 = No<br>1 = Yes	Categorical variable	Acces s to blood pressu re testing	Opinion

For peer review only - http://bmjopen.bmj.com/site/about/guidelines.xhtml

Q38	Did you get your blood glucose tested at the selected place(s) in Q24?<br><br>*-Record 99 if don't know/unsure*	0 = No<br>1 = Yes	Categorical variable	Access to blood glucose testing	Opinion
Q39	Would you recommend the selected place(s) in Q24 to others?<br><br>*-Record 99 if don't know/unsure*	1 = Not recommended<br>2 = Recommend with reservations<br>3 = Recommend<br>4 = Highly recommend	Categorical ordinal	Patient satisfaction of care services	Opinion

peer review only

For peer review only - http://bmjopen.bmj.com/site/about/guidelines.xhtml

For peer review only

For peer review only - http://bmjopen.bmj.com/site/about/guidelines.xhtml

Annexure 4. Arithmetic and the geometric mean of medical cost by participant characteristics in Cambodia, 2020

Table S1. Arithmetic mean of medical cost by participant characteristics in Cambodia, 2020

Variable	Overall	No T2D/HTN	HTN	T2D	T2D plus HTN
	Mean [95% CI]	Mean [95% CI]	Mean [95% CI]	Mean [95% CI]	Mean [95% CI]
Sex					
Male	16.8 [15.2–18.5]	16.8 [14.7–18.9]	15.5 [12.3–18.6]	25.5 [15.8–35.2]	17.0 [10.2–23.7]
Female	19.5 [18.3–20.6]	22.6 [20.8–24.4]	14.3 [12.7–15.8]	21.1 [16.0–26.2]	22.2 [18.9–25.5]
P-value	0.015	<0.001	0.480	0.393	0.212
Age in years					
40–49	19.0 [16.8–21.2]	20.6 [18.1–23.2]	20.6 [18.1–23.2]	10.3 [6.5–14.0]	19.7 [7.1–32.4]
50–59	19.1 [17.5–20.7]	82.8 [73.0–92.6]	20.7 [18.3–23.2]	14.2 [12.0–16.5]	24.5 [17.6–31.3]
60+	18.2 [16.8–19.7]	82.1 [72.8–91.4]	20.5 [18.2–22.9]	15.4 [13.4–17.4]	21.1 [13.8–28.4]
P-value	0.698	0.995	0.120	0.704	0.850
Educational level					
No schooling	19.1 [17.4–20.9]	20.6 [17.9–23.3]	16.2 [13.8–18.7]	22.8 [14.8–30.7]	22.1 [15.8–28.5]
Primary	17.9 [16.7–19.1]	20.3 [18.5–22.1]	12.9 [11.1–14.7]	20.5 [15.1–26.0]	19.9 [16.7–23.2]
Secondary/higher	21.1 [18.0–24.3]	22.0 [17.9–26.2]	16.9 [11.5–22.2]	28.6 [8.8–48.4]	26.8 [14.3–39.2]
P-value	0.091	0.726	0.054	0.506	0.365
Having NSSF membership					
No	18.8 [17.8–19.7]	20.7 [19.2–22.1]	14.5 [13.1–16.0]	22.9 [18.1–27.6]	21.9 [18.8–25.0]
Yes	17.5 [13.2–21.8]	20.4 [13.9–26.9]	14.7 [7.0–22.4]	13.8 [1.6–26.0]	11.8 [4.7–18.8]
P-value	0.5604	0.937	0.961	0.339	0.076
Having HEF membership					
No	19.4 [18.3–20.5]	21.4 [19.8–23.0]	14.9 [13.3–16.5]	24.1 [18.8–29.3]	22.6 [19.4–25.9]
Yes	15.8 [13.8–17.8]	17.6 [14.6–20.5]	13.2 [10.2–16.2]	14.3 [11.0–18.6]	14.9 [7.5–22.3]
P-value	0.004	0.035	0.360	0.096	0.058
Household socio-economic class					
Poorest	20.2 [17.7–22.8]	23.9 [20.1–27.6]	13.6 [10.3–16.9]	18.0 [6.6–29.4]	23.6 [14.8–32.4]
Poor	16.9 [14.8–18.9]	17.8 [15.0–20.6]	14.7 [11.4–18.0]	23.2 [11.3–35.1]	14.8 [6.0–23.7]
Medium	18.2 [16.2–20.2]	19.8 [16.9–22.6]	14.7 [11.4–18.0]	18.8 [12.5–25.0]	21.2 [14.4–28.1]
Rich	20.0 [17.8–22.2]	23.0 [19.4–26.5]	15.6 [12.5–18.6]	23.4 [10.3–36.6]	21.5 [16.8–26.3]
Richest	18.2 [16.2–20.2]	19.2 [16.4–22.0]	14.0 [11.0–17.0]	26.8 [15.8–37.7]	24.1 [17.4–30.8]
P-value	0.171	0.034	0.919	0.687	0.427
Sector					
Private	20.5 [19.3–21.6]	23.0 [21.4–24.7]	15.7 [14.0–17.3]	24.9 [18.3–31.6]	22.0 [18.0–26.0]
Public	12.0 [10.3–13.7]	9.8 [7.4–12.2]	10.6 [7.7–13.6]	18.5 [13.2–23.8]	20.4 [15.6–25.2]
Both	21.7 [17.4–26.1]	24.8 [19.5–30.1]	9.4 [5.7–13.1]	__	20.5 [10.6–30.4]

For peer review only

P-value	<0.001	<0.001	0.013	0.164	0.878
Care model					
Coexisting	16.7 [14.7–18.7]	17.9 [15.1–20.6]	13.8 [10.8–16.9]	20.0 [11.8–28.2]	23.3 [12.1–34.6]
Community-based	22.6 [20.3–25.0]	24.5 [21.1–27.9]	18.9 [15.1–22.7]	22.3 [12.7–32.0]	27.2 [19.2–35.2]
Health center-based (high)	18.6 [16.4–20.7]	20.7 [17.5–24.0]	14.8 [11.7–17.8]	19.7 [9.2–30.1]	22.8 [14.6–30.9]
Health center-based (low)	15.7 [13.9–17.5]	17.6 [14.8–20.4]	11.0 [8.8–13.2]	24.1 [13.1–35.2]	19.0 [15.1–22.9]
Hospital-based	20.3 [17.9–22.7]	22.8 [19.4–26.2]	14.8 [10.9–18.7]	23.3 [12.1–34.6]	18.1 [12.0–24.1]
P-value	<0.001	0.006	0.011	0.896	0.2695

Abbreviation CI, confidence interval

Note: Health center-based (high) means the OD with high coverage (six out of nine) of health centers with the WHO PEN; Health center-based (low) means the OD with low coverage (six out of 25) of health centers with the WHO PEN

Table S2. Geometric mean of medical cost by participant characteristics in Cambodia, 2020

Variable	**Overall**	**No T2D/HTN**	**HTN**	**T2D**	**T2D plus HTN**
	Mean [95% CI]	Mean [95% CI]	Mean [95% CI]	Mean [95% CI]	Mean [95% CI]
Sex					
Male	6.9 [6.2–7.8]	6.5 [5.6–7.5]	7.0 [5.7–8.6]	14.7 [8.8–24.7]	8.5 [4.2–17.0]
Female	8.5 [7.9–9.1]	8.9 [8.0–10.0]	6.8 [6.1–7.6]	12.5 [9.4–16.5]	13.9 [11.1–17.5]
P-value	**0.004**	**<0.001**	0.79	0.548	**0.104**
Age in years					
40–49	7.9 [6.8–9.0]	8.2 [7.0–9.7]	8.2 [7.0–9.7]	5.0 [3.7–6.8]	13.2 [8.3–20.9]
50–59	8.4 [7.6–9.4]	28.2 [23.6–33.6]	7.9 [6.7–9.2]	7.6 [6.4–8.9]	15.2 [10.6–21.9]
60+	7.7 [7.0–8.5]	28.4 [23.7–33.9]	7.9 [6.8–9.3]	6.9 [6.0–7.8]	11.1 [7.1–17.3]
P-value	0.480	0.91	**0.09**	0.509	0.384
Educational level					
No schooling	7.9 [7.0–8.8]	7.4 [6.2–8.8]	7.6 [6.4–8.9]	13.0 [8.1–20.8]	11.6 [7.4–18.2]
Primary	8.0 [7.4–8.7]	8.3 [7.4–9.4]	6.4 [5.7–7.3]	12.6 [9.2–17.3]	12.9 [9.8–17.0]
Secondary/higher	8.2 [6.8–9.9]	8.0 [6.2–10.4]	6.6 [4.7–9.4]	15.7 [7.0–35.1]	17.2 [9.2–32.2]
P-value	0.920	0.510	0.299	0.842	0.600
Having NSSF membership					
No	8.1 [7.6–8.6]	8.0 [7.3–8.8]	7.0 [6.3–7.7]	13.6 [10.6–17.4]	13.5 [10.8–16.9]
Yes	6.8 [5.0–9.2]	7.9 [5.2–12.0]	5.3 [3.0–9.2]	7.6 [1.5–37.8]	6.3 [2.2–18.1]
P-value	**0.2351**	0.937	0.234	0.261	**0.071**
Having HEF membership					
No	8.8 [8.2–9.4]	8.7 [7.9–9.7]	7.4 [6.6–8.2]	14.1 [7.4–20.9]	16.1 [13.5–19.3]
Yes	5.4 [4.6–6.3]	5.5 [4.4–6.9]	5.1 [4.0–6.7]	8.6 [4.5–16.3]	4.1 [1.7–9.9]
P-value	**<0.001**	**<0.001**	**0.004**	**0.114**	**<0.001**
Household socio-economic class					

For peer review only

Poorest	6.8 [5.8–8.0]	7.3 [5.8–9.2]	5.4 [4.1–6.9]	8.4 [3.9–18.3]	11.1 [5.5–22.4]
Poor	7.4 [6.5–8.5]	7.4 [6.1–8.9]	7.1 [5.7–8.8]	13.6 [7.2–25.9]	6.6 [3.2–13.7]
Medium	8.4 [7.3–9.6]	8.2 [6.7–10.0]	7.4 [6.0–9.1]	12.6 [7.9–20.1]	12.5 [7.5–20.8]
Rich	9.4 [8.2–10.8]	9.6 [7.8–11.9]	7.9 [6.5–9.6]	14.1 [6.8–28.9]	15.8 [11.1–22.5]
Richest	8.2 [7.2–9.3]	7.8 [6.5–9.4]	6.7 [5.5–8.3]	16.5 [10.5–25.7]	19.0 [14.4–25.1]
P-value	**0.022**	0.366	**0.133**	0.538	**0.054**
Sector					
Private	9.8 [9.2–10.5]	10.2 [9.2–11.2]	8.4 [7.6–9.2]	15.0 [11.2–20.2]	15.5 [12.6–19.0]
Public	3.5 [3.0–4.1]	2.5 [2.0–3.1]	3.1 [2.4–4.1]	10.7 [7.0–16.3]	9.5 [5.9–15.2]
Both	13.9 [10.8–17.9]	16.2 [12.2–21.5]	6.9 [4.0–12.1]	—	20.3 [12.7–32.4]
P-value	**<0.001**	**<0.001**	**<0.001**	**0.176**	**0.084**
Care model					
Coexisting	7.3 [6.3–8.5]	7.5 [6.2–9.2]	6.0 [4.7–7.6]	12.8 [6.9–23.5]	17.5 [10.8–28.3]
Community-based	10.8 [9.4–12.3]	10.4 [8.5–12.8]	9.7 [7.9–11.8]	16.5 [10.8–25.2]	20.5 [14.3–29.2]
Health center-based (high)	8.5 [7.5–9.7]	8.6 [7.0–10.5]	7.4 [6.1–9.0]	11.4 [7.1–18.2]	15.0 [9.5–23.9]
Health center-based (low)	6.4 [5.6–7.3]	5.9 [4.8–7.2]	5.6 [4.6–6.9]	13.7 [6.9–27.4]	13.9 [9.5–20.1]
Hospital-based	7.9 [6.7–9.2]	8.5 [6.9–10.5]	6.3 [4.8–8.1]	17.5 [10.8–28.3]	7.3 [4.2–12.8]
P-value	**<0.001**	**0.002**	**0.003**	0.911	**0.015**

Abbreviation CI, confidence interval

Note: Health center-based (high) means the OD with high coverage (six out of nine) of health centers with the WHO PEN; Health center-based (low) means the OD with low coverage (six out of 25) of health centers with the WHO PEN

For peer review only

STROBE Statement—Checklist of items that should be included in reports of ***cross-sectional studies***

	Item No	Recommendation	Page No
**Title and abstract**	1	(*a*) Indicate the study's design with a commonly used term in the title or the abstract	1
		(*b*) Provide in the abstract an informative and balanced summary of what was done and what was found	2-3
**Introduction**			
Background/rationale	2	Explain the scientific background and rationale for the investigation being reported	5
Objectives	3	State specific objectives, including any prespecified hypotheses	7
**Methods**			
Study design	4	Present key elements of study design early in the paper	10
Setting	5	Describe the setting, locations, and relevant dates, including periods of recruitment, exposure, follow-up, and data collection	10-14
Participants	6	(*a*) Give the eligibility criteria, and the sources and methods of selection of participants	12
Variables	7	Clearly define all outcomes, exposures, predictors, potential confounders, and effect modifiers. Give diagnostic criteria, if applicable	13-14
Data sources/ measurement	8*	For each variable of interest, give sources of data and details of methods of assessment (measurement). Describe comparability of assessment methods if there is more than one group	10, 13, 14
Bias	9	Describe any efforts to address potential sources of bias	12
Study size	10	Explain how the study size was arrived at	12-13
Quantitative variables	11	Explain how quantitative variables were handled in the analyses. If applicable, describe which groupings were chosen and why	15-16
Statistical methods	12	(*a*) Describe all statistical methods, including those used to control for confounding	15-16
		(*b*) Describe any methods used to examine subgroups and interactions	15-16
		(*c*) Explain how missing data were addressed	NA
		(*d*) If applicable, describe analytical methods taking account of sampling strategy	NA
		(*e*) Describe any sensitivity analyses	NA
**Results**			
Participants	13*	(a) Report numbers of individuals at each stage of study—eg numbers potentially eligible, examined for eligibility, confirmed eligible, included in the study, completing follow-up, and analysed	17
		(b) Give reasons for non-participation at each stage	17, 12, 13
		(c) Consider use of a flow diagram	13
Descriptive data	14*	(a) Give characteristics of study participants (eg demographic, clinical, social) and information on exposures and potential confounders	17
		(b) Indicate number of participants with missing data for each variable of interest	17
Outcome data	15*	Report numbers of outcome events or summary measures	18-19, 22

For peer review only - http://bmjopen.bmj.com/site/about/guidelines.xhtml

Main results	16	(*a*) Give unadjusted estimates and, if applicable, confounder-adjusted estimates and their precision (eg, 95% confidence interval). Make clear which confounders were adjusted for and why they were included	19-20, 23-24
		(*b*) Report category boundaries when continuous variables were categorized	NA
		(*c*) If relevant, consider translating estimates of relative risk into absolute risk for a meaningful time period	NA
Other analyses	17	Report other analyses done—eg analyses of subgroups and interactions, and sensitivity analyses	NA
**Discussion**			
Key results	18	Summarise key results with reference to study objectives	25
Limitations	19	Discuss limitations of the study, taking into account sources of potential bias or imprecision. Discuss both direction and magnitude of any potential bias	27-28
Interpretation	20	Give a cautious overall interpretation of results considering objectives, limitations, multiplicity of analyses, results from similar studies, and other relevant evidence	25-28
Generalisability	21	Discuss the generalisability (external validity) of the study results	27
**Other information**			
Funding	22	Give the source of funding and the role of the funders for the present study and, if applicable, for the original study on which the present article is based	29

*Give information separately for exposed and unexposed groups.

Note: An Explanation and Elaboration article discusses each checklist item and gives methodological background and published examples of transparent reporting. The STROBE checklist is best used in conjunction with this article (freely available on the Web sites of PLoS Medicine at http://www.plosmedicine.org/, Annals of Internal Medicine at http://www.annals.org/, and Epidemiology at http://www.epidem.com/). Information on the STROBE Initiative is available at www.strobe-statement.org.

For peer review only

For peer review only - http://bmjopen.bmj.com/site/about/guidelines.xhtml

BMJ Open

Healthcare utilization and expenditure among people with type 2 diabetes and/or hypertension in Cambodia: results from a cross-sectional survey

Journal:	*BMJ Open*
Manuscript ID	bmjopen-2022-061959.R1
Article Type:	Original research
Date Submitted by the Author:	25-Aug-2022
Complete List of Authors:	Chhim, Srean; National Institute of Public Health, Technical Office<br>Te, Vannarath; National Institute of Public Health, School of Public Health<br>Buffel, Veerle; University of Antwerp, Sociology<br>van Olmen, Josefien; University of Antwerp, ELIZA<br>Chham, Savina; National Institute of Public Health,<br>Long, Sereyraksmey; National Institute of Public Health, School of Public Health<br>Yem, Sokunthea; National Institute of Public Health, Technical Office<br>Van Damme, Wim; Institute of Tropical Medicine, Public Health; Vrije Universiteit Brussel, Gerontology<br>Wouters, Edwin; University of Antwerp, Centre for Longitudinal & Life Course Studies; University of the Free State, Centre for Health Systems Research & Development<br>Por, Ir; National Institute of Public Health
**Primary Subject Heading**:	Public health
Secondary Subject Heading:	Health services research
Keywords:	HEALTH SERVICES ADMINISTRATION & MANAGEMENT, HEALTH ECONOMICS, Hypertension < CARDIOLOGY, General diabetes < DIABETES & ENDOCRINOLOGY, Organisation of health services < HEALTH SERVICES ADMINISTRATION & MANAGEMENT

SCHOLARONE™
Manuscripts

I, the Submitting Author has the right to grant and does grant on behalf of all authors of the Work (as defined in the below author licence), an exclusive licence and/or a non-exclusive licence for contributions from authors who are: i) UK Crown employees; ii) where BMJ has agreed a CC-BY licence shall apply, and/or iii) in accordance with the terms applicable for US Federal Government officers or employees acting as part of their official duties; on a worldwide, perpetual, irrevocable, royalty-free basis to BMJ Publishing Group Ltd ("BMJ") its licensees and where the relevant Journal is co-owned by BMJ to the co-owners of the Journal, to publish the Work in this journal and any other BMJ products and to exploit all rights, as set out in our licence.

The Submitting Author accepts and understands that any supply made under these terms is made by BMJ to the Submitting Author unless you are acting as an employee on behalf of your employer or a postgraduate student of an affiliated institution which is paying any applicable article publishing charge ("APC") for Open Access articles. Where the Submitting Author wishes to make the Work available on an Open Access basis (and intends to pay the relevant APC), the terms of reuse of such Open Access shall be governed by a Creative Commons licence – details of these licences and which Creative Commons licence will apply to this Work are set out in our licence referred to above.

Other than as permitted in any relevant BMJ Author's Self Archiving Policies, I confirm this Work has not been accepted for publication elsewhere, is not being considered for publication elsewhere and does not duplicate material already published. I confirm all authors consent to publication of this Work and authorise the granting of this licence.

For peer review only

Healthcare utilization and expenditure among people with type 2 diabetes and/or hypertension in Cambodia: results from a cross-sectional survey

Srean Chhim[1,2*], Vannarath Te[2,3,4], Veerle Buffel[5], Josefien Van Olmen[4], Savina Chham[1,5], Sereyraksmey Long[2], Sokunthea Yem[1], Wim Van Damme[3], Edwin Wouters[5,6], Por Ir[1,2]

Affiliations

[1] Technical Bureau, National Institute of Public Health, Phnom Penh, Cambodia

[2] School of Public Health, National Institute of Public Health, Phnom Penh, Cambodia

[3] Health Policy Unit, Department of Public Health, Institute of Tropical Medicine, Antwerp, Belgium

[4] Department of Family Medicine and Population Health (FAMPOP), Faculty of Medicine and Health Sciences, University of Antwerp, Belgium

[5] Centre for Population, Family, and Health, Department of Social Sciences, University of Antwerp, Belgium

[6] Centre for Health Systems Research and Development, University of the Free State, South Africa

*** *Corresponding author:*** Srean Chhim (#109, street 01, Krang Thnong, Saensokh, Phnom Penh, postal 120804), srean168@gmail.com

Keywords: healthcare, utilization, expenditure, Cambodia

Word count 5399/4000

ABSTRACT

Objective: To assess utilization of public and private healthcare, related healthcare expenditure, and associated factors for people with type 2 diabetes (T2D) and/or hypertension (HTN) and for people without those conditions in Cambodia.

Methods: A cross-sectional household survey.

Settings: Five operational districts in Cambodia.

Participants: Data was from 2,360 participants aged ≥ 40 years who had used healthcare services at least once in the three months preceding the survey.

Primary and secondary outcome: The main variables of interest were the number of healthcare visits and healthcare expenditure in the last three months.

Results: The majority of healthcare visits took place in the private sector. Only 22.0% of healthcare visits took place in public healthcare facilities: 21.7% in people with HTN, 37.2% in people with T2D, 34.7% in people with T2D plus HTN, and 18.9% in people without the two conditions (*P*-value<0.01). For people with T2D and/or HTN, increased public healthcare use was significantly associated with Health Equity Fund (HEF) membership and living in operational districts with *community-based care*. Furthermore, significant healthcare expenditure reduction was associated with HEF membership and using public healthcare facilities in these populations.

Conclusion: Overall public healthcare utilization was relatively low; however, it was higher in people with chronic conditions. HEF membership and *community-based care* contributed to higher public healthcare utilization among people with chronic conditions. Using public healthcare services, regardless of HEF status reduced healthcare expenditure, but the reduction in spending was more noticeable in people with HEF membership. To protect people with T2D

For peer review only

and/or HTN from financial risk and move towards the direction of universal health coverage, the public healthcare system should further improve care quality and expand social health protection. Future research should link healthcare use and expenditure across different healthcare models to actual treatment outcomes to denote areas for further investment.

For peer review only

STRENGTHS AND LIMITATIONS OF THIS STUDY

- Our study is among the few to examine healthcare utilization and expenditure among people with type 2 diabetes (T2D) and/or hypertension (HTN) in comparison to people without these two conditions in Cambodia.
- The sampling design--randomizing villages, households, and household members--is robust within its scope, targeting the population in rural or semi-rural settings in Cambodia.
- The data collection is robust and ensures a reliable dataset.
- The fact that the five operational districts were selected purposively limited the generalizability for the national level as most of the study sites we selected were rural or semi-rural.
- The sample size for the group of people with T2D only and people with T2D plus HTN may be relatively small and may have insufficient power to assess the association between outcome variables and the dependent variables.

For peer review only

INTRODUCTION

Type 2 diabetes (T2D) and hypertension (HTN) are global public health concerns. They are major risk factors for cardiovascular diseases, causing about 31% (17.9 million) of all deaths worldwide annually.[1] The prevalence of people with T2D and/or HTN will likely continue to increase.[2] These two diseases disproportionately affect low- and middle-income countries and account for around 75% of all deaths in these countries.[1]

In Cambodia, large-scale population-based studies such as STEPS Surveys have shed light on the prevalence and risk factors of chronic diseases. The prevalence of T2D and HTN rose noticeably over recent years from 2.9% and 11.2% in the population aged 25–64 years in 2010 to 9.6% and 14.2% in the population aged 18–69 years in 2016, respectively.[3]

Previous nationally representative surveys have shown that a majority of the population seeks outpatient curative care in private facilities, but knowledge on the related healthcare uptake and expenditure among those with T2D and/or HTN is scarce.[4] Additionally, these surveys do not cover topics related to the management of the diseases, such as healthcare utilization and expenditure.[5] There is only one study, by Bigdeli *et al,*. which examines access to care for people with T2D and/or HTN concerning social health protection schemes in Cambodia.[6] This study shows that 61% of the people with T2D and/or HTN who knew their status were diagnosed in private facilities.[6] However, this study collected data in 2013, before key interventions were introduced or expanded in public healthcare facilities.[7] Also, it provides limited information about which types of health facilities were used, and what differences exist between people with one or both conditions compared to those without.

In the last decade, the Cambodian Ministry of Health (MoH), in collaboration with development partners, made significant efforts to improve the quality of public healthcare and initiated a few

For peer review only

87 healthcare delivery models for people with T2D and/or HTN in public facilities.[7] These models include *hospital-based care, health center-based care, community-based care* and a combination of all three models (*coexisting care*) (Box 1). These efforts might have changed the pattern of healthcare utilization and related expenditure, especially among people with T2D and/or HTN.

Box 1. Overview of different care models in Cambodia in 2021

- The *hospital-based care* model is standard care, which means an operational district (OD) has a government-run Non-communicable Disease (NCD) clinic at the district referral hospitals.[7] By 2021, 31 out of 117 referral hospitals had implemented this model. the patients refer themselves to the units (and they are thus not transferred by an intermediary unit).
- The *health center-based care* model adopts the World Health Organization Package of Essential Non-communicable Disease Interventions (WHO PEN).[7] In this model, the MoH added the function of a health center to *hospital-based care*. However, the coverage of health centers with the WHO PEN varies in each OD, which can be divided into low coverage (<50% of all health centers implement the WHO PEN; *health center-based care (low)*) and high coverage (≥50% of all health centers implement the WHO PEN; *health center-based care (high)*). The referral flow is slightly different between T2D and HTN. For T2D, this model identifies cases in the health centers through a screening test. If the patients are suspected of having T2D, health centers refer them to a diabetes clinic at a district referral hospital for confirmation of diagnosis. Once diagnosed, severe cases are treated in the hospital clinic, and stable or mild cases are followed up regularly at the health centers. For HTN, the health centers treat mild

For peer review only

patients and refer the severe cases to the referral hospital. By 2021, *health center-based care* was implemented in 137 of 1,221 health centers.

- The *community-based care* model or peer education network established and run by MoPoTsyo, a local non-governmental organization.[7] In this model, peer educators (PEs) are added to hospital-based care. MoPoTsyo trained people with T2D and/or HTN to be PEs. These PEs play a role in screening and referring those suspected of having T2D and/or HTN to seek medical consultation and treatment at the referral hospitals that MoPoTsyo has partnered with. The PEs also provide counseling on lifestyle changes and support self-management to registered network members. By 2019, this *community-based care* model had been implemented in 20 of 102 ODs in 8 of 25 provinces in Cambodia.[7 8] It had 225 PEs to serve 40,000 people with T2D.[8]
- The *coexisting care* model comprises a combination of the above three models. At the time of the study until 2021, only one OD (Daunkeo) had this model.

A better understanding of the current patterns of healthcare utilization and expenditure among people with T2D and/or HTN is critical for better resource allocations and strategies to improve the management of T2D and HTN. The main objectives of this study are two-fold. First, it evaluates usage and determines the factors associated with public healthcare use in four groups: (1) people without T2D or HTN, (2) people with T2D alone, (3) people with HTN alone, and (4) people with T2D plus HTN. Second, it assesses the healthcare expenditure in the three months preceding the survey for all services used by the four patient groups in public and private facilities and determines factors associated with (reducing or increasing) healthcare expenditure.

For peer review only

Context

The health system in Cambodia is pluralistic, meaning healthcare services are provided by both public and private healthcare providers.[4 9]

Public healthcare services in Cambodia dominate preventive services (reproductive, maternal, neonatal, and child health), control of primary disease (tuberculosis, malaria, and HIV/AIDS control), and inpatient treatment.[4] The facilities include health posts, health centers, district referral hospitals, provincial referral hospitals, and national hospitals.[4] Public healthcare is organized per operational district (OD)—the third and last administrative level in Cambodia's health system management.[4] An OD covers a population of 100,000–200,000 people while a health center covers a population of 10,000–20,000 people.[4] Remote areas with a small population can be covered by a health post.[4] The health post provides similar services to a health center, but it is smaller than a health center.[4] Each OD usually has one district referral hospital with a few ODs having two district hospitals.[4] The district referral hospital receives self-referred patients or those referred by the health centers.

Alongside this public sector, a large *private healthcare sector* exists, which is more accessible than the public sector, and dominates outpatient curative care.[4] Since 1994, the Cambodian government started economic liberalization, permitting staff to work outside their government's working hours and own healthcare facilities.[4] Since then, the private healthcare sector and dual practice system, meaning public healthcare workers also have private practices, have grown rapidly. In 2015, over 50% of the healthcare workforce in private healthcare facilities were government personnel.[4] The private healthcare facilities range from cabinets, laboratories, pharmacies, clinics, and polyclinics to hospitals.[4] Cabinets are the smallest facilities with less than two beds and mainly provide medical consultation services.[10 11] According to the MoH

For peer review only

Progress Report in 2018, over 90% of private healthcare facilities were cabinets.[10 11] The second most frequent facilities were clinics (3.2%), providing medical specialties, laboratories, radiology services, and pharmacies.[10] A clinic has between 10 and 20 beds.[10] In addition, buying medication in pharmacies or drugstores for self-treatment without a doctor's prescription is common in Cambodia, although not permitted by law.[12]

In terms of health expenditure, the public healthcare sector did not charge user fees until 1996.[4] In that year, the government introduced a user-fee scheme for the public sector with fees approved by the local community to increase healthcare quality at public healthcare facilities.[4 13] The revenue from the user-fee scheme could be used to incentivize staff and support ongoing operations. However, the user-fee posed challenges for the poor to access public healthcare. To address this, the MoH established the Health Equity Fund (HEF) in 2000, a pro-poor social health protection scheme.[14] The HEF is linked to the implementation of identification of the poor (known as "IDPoor").[15] It is intended for the "extremely poor" or "poor" category, which is assessed and verified by the local authorities.[15] People with IDPoor are entitled to HEF support, meaning that they receive free healthcare services at public healthcare facilities and transportation expenditure reimbursement.[15] By 2019, the HEF covered approximately three million or about 20% of Cambodia's population.[14] Another scheme is the National Social Security Fund (NSSF), established in 2007.[16] The NSSF covers work and non-work-related illnesses and injuries for formally employed people.[16] Formal employers are mandated to pay for their staff's NSSF membership. The NSSF had enrolled over 1.7 million employees or about 11% of the population by 2019.[17]

However, it is important to note that several studies have indicated that the private sector still constitutes a significant source for out-of-pocket expenditure (OOPE).[18 19] Between 2009 and

For peer review only

2016, around 60% of health expenditure was OOPE while the rest was a combination of the government's and development partners' budgets. The OOPE per capita increased slightly from USD 40.6 in 2009 to USD 48.1 in 2016.[19] In 2016, 76.6% of the total OOPE was linked to private healthcare.[18 19]

For peer review only

METHODS

Data sources

This study is part of a larger cross-sectional household survey, with the primary aim of developing a care cascade for T2D and HTN.

Settings

The study purposively selected five ODs. The selection was made to include different T2D and/or HTN care models piloted in Cambodia: *coexisting care*, *community-based care*, *health-center based care (high)*, *health-center based care (low),* and *hospital-based care* (Box 1). The five ODs in which the study took place are out of 103 ODs in the country and located in five different provinces. The map of ODs is presented in Annexure 1. These ODs have similar road infrastructure improvements, in which poor road conditions are no longer a barrier to accessing healthcare.

- *OD Daunkeo, Takeo province*: This OD had the *"coexisting care"* model. At the time of the study, it was the only OD in which the three care models coexisted. The catchment area included Takeo town and a large rural area. Its NCD clinic was established in 2002, and the peer educator network was initiated in 2007 and handed over to the MoH in 2015.[20] The WHO PEN was implemented in 5 out of 14 health centers since 2015. The private services for people with T2D and/or HTN may also be easily accessible.
- *OD Kong Pisey, Kampong Speu province*: This OD had the *"community-based care"* model. It has a strong MoPoTsyo network to provide T2D and HTN care to patients. Located about 54 kilometers from the capital of Phnom Penh, this OD is semi-urban with a variety of private facilities.

For peer review only

- *OD Pearaing, Prey Veng province*: This OD had the *"health center-based (high)"* model, and was the OD with high coverage of the WHO PEN. Six out of nine health centers in this OD have been piloting the WHO PEN since 2015. Due to dual practice, the high coverage of the WHO PEN also facilitates accessible private services for people with T2D and/or HTN.
- *OD Sot Nikum, Siem Reap province*: This OD had the *"health center-based (low)"* model, and was the OD with low coverage of the WHO PEN (6/25 of the health centers started the WHO PEN in 2018). This OD has been historically and significantly influenced by the financial support of various development partners, and services for people with T2D and/or HTN have been well arranged at its NCD clinic.[21]
- *OD Samrong, Oddar Meanchey province*: This OD had a "*hospital-based care"* model. It had an NCD clinic without the WHO PEN and peer educator network. A large part of the catchment area is a remote area bordering Thailand, approximately 470 kilometers from the capital. Therefore, the private services for people with T2D and/or HTN may not be broadly accessible.

Samples

The larger household survey recruited 5,072 individuals aged 40 years or older to participate in the study using a multi-stage cluster sampling method. Initially, it purposively chose five ODs with different care models for T2D and HTN. Second, 44 villages per OD were randomly selected, regardless of the population size of each OD. The purpose of this equal probability selection was to over-sample participants in ODs with a smaller population so that they would have an adequate sample for each care model. Third, 24 households in each village were selected

For peer review only

by probability systematic sampling, and finally, one person aged 40 years or older per household was selected at random. To minimize the non-response rate, which can unintentionally exclude a certain group of the target population from the survey, the selected participants were called back or followed-up three times when they were absent from their household. If the attempt failed, another household in the next row in the sampling list was selected. Then, the procedure described above was repeated. The equal probability selection at the village and household levels were used with the OD level's same purpose.

To correspond to our analytical objective, we used a subset of this sample: we only retained those who reported using healthcare services at least once in the three months preceding the survey (Figure 1). A total of 2,360/5,072 participants met this criterion. The 2,360-participant sample subset included four patient groups: 1,331 people without T2D and HTN, 761 people with HTN alone, 109 people with T2D alone, and 159 people with T2D plus HTN.

For peer review only

Data collection

The data collection took place between July and October 2020. The data collection was conducted in three steps following the WHO's STEPS Survey approach: (1) interviews with a structured questionnaire, (2) anthropometric measurements, and (3) biochemical measurements.[5] Since our study only focuses on healthcare utilization and expenditure, we only used information from step 1—interviews with a structured questionnaire. The questionnaire was tablet-based and comprised of 11 sections, including socio-demographic information, health status and quality of life, healthcare utilization, social support, lifestyle or behavior measures, physical activity, diabetes and hypertension knowledge, medication adherence, self-management support, and decision-making power on food. However, we only used two sections in our analysis: socio-demographic information and healthcare utilization. The tablet-based questionnaire was installed using the Kobo Toolbox (https://kf.kobotoolbox.org), an open-source software with a free-of-charge server and online storage.[22]

Measures

This study's primary variable of interest is the number of visits to public and private facilities. By public healthcare facilities, we refer to government-run facilities that provide medical services, and include national hospitals, provincial referral hospitals, district referral hospitals, health centers, and health posts. A health post is similar to a health center, and only a few exist in remote areas. Therefore, we grouped them with health centers. Private healthcare services are non-government organizations that provide medical and non-medical services, and include private hospitals, private clinics, pharmacies, homes of trained health workers, and visits of

For peer review only

health workers to the patients' homes. Traditional healing/medicine and using healthcare services abroad have also been included in this category.

The secondary variable of interest was healthcare expenditure, the lump sum expenditure of medical consultation, treatment, and medication. These data were obtained from the interview with the participants. They were asked about their use of health services in the three months preceding the survey (where they went, how often they went to a particular type of healthcare facility, and how much they spent in each facility in those three months). We include the questionnaire in Annexure 2. The Cambodian currency (riels) was converted into USD at an exchange rate of 4,000 riels per USD. The expenditure does not include other spendings such as on transport, food, or guesthouses/hotels.

To better understand the profile of people using public or private healthcare facilities, we estimated associations between the use of public and private healthcare services and patient characteristics such as sex (male, female), age (40–49, 50–59, 60+ years old), educational level (none, primary, secondary or higher), social protection status (NSSF (yes, no), HEF (yes, no)), wealth quintile (poorest, poor, medium, rich, richest), type of care models (*hospital-based*, *health center-based (high)*, *health center-based (low)*, and *community-based*). The details on wealth quintile calculation (socio-economic class) are described in Annexure 3.

Analysis

Healthcare utilization

Taking the number of visits as a dependent variable, we report the healthcare visit rate to public and private facilities over the three months preceding the survey, then stratified by patient groups.

For peer review only

To identify the independent factors associated with healthcare utilization (defined by the number of visits), we first used bivariate negative binomial regression to identify the potential factors in the five groups—overall and four patient groups—separately. Variables with a P-value<0.25 in at least one of the four patient groups or overall group were included in the multiple negative binomial regression. The exposure variable (total healthcare visits of each participant) was incorporated into this model. Variables with a P-value<0.05 were considered statistically significant in this final model. The negative binomial regression was chosen over Poisson regression because the number of visits was over-dispersed.

Healthcare expenditure

We took healthcare expenditure in the three months preceding the survey as the dependent variable. Due to the limitation of our data, we focused more on assessing the factors associated with healthcare expenditure and did not explore the overall medical expenditure. We reported the overall arithmetic mean and then stratified the mean by patient groups. The expenditure was calculated separately for each patient group. Because arithmetic means can be easily affected by extremely high values, we removed the values above the 90th percentile, which we believe were too high in our sample.

Our analysis was carried out in three steps to separately identify the independent factors associated with healthcare expenditure in the four patient groups. First, a logarithmic transformation of the healthcare expenditure was performed as the data was skewed to the right. Second, in the bivariate analysis, we compared the geometric mean of healthcare expenditure by characteristics of the participants. This analysis identified the variables potentially associated with the healthcare expenditure at a P-value<0.25. During this phase, the Student's t-test for

For peer review only

binary explanatory variables and the one-way ANOVA test for non-binary explanatory variables were used. Variables with a significant level at a P-value<0.25 in any patient group were included in the multiple linear regression. Third, multiple linear regression was performed and the coefficient and 95% confidence interval (CI) values were exponentiated to a risk ratio (RR) for better interpretation.

Data were analyzed using Stata 16.0 (Stata Corp LLC, College Station, Texas 77, USA), and R programing's GGPLOT2 package was used to produce the graphs.

Ethical approval

The protocol was approved by the National Ethics Committee for Human Research (NECHR) on April 29, 2019 (No. 105 NECHR) and by the Institutional Review Board of Institute of Tropical Medicine (Antwerp) on October 25, 2019 (No. 1,323/19). All participants provided their consent and volunteered to take part in the study.

Patient and public involvement

No patient was involved in the development of the research question and outcome measures, study design, and study participant recruitment. The findings are not disseminated to the study participants.

RESULTS

Characteristics of participants

Our analysis included 2,360 participants, including 1,331 people without T2D or HTN, 761 people with HTN alone, 109 with T2D alone, and 159 with T2D plus HTN (Table 1). The other

For peer review only

participants were excluded because they had not used healthcare services in the three months preceding the survey (N=2,703) or had a missing response to the primary variable of interest (N=9).

As shown in Table 1, females were more prevalent in all patient groups, especially in the T2D plus HTN group. The age range was between 40 and 96 years, with people with HTN and T2D plus HTN having a significantly higher average age than those without the two conditions. The majority of participants did not attend school or attended only primary school.

Regarding the social health protection scheme, a small proportion of participants in all groups had the NSSF membership (4.8% overall). A larger proportion of patients across all groups had the HEF membership (18.4% overall).

Table 1. Demographic and socio-economic characteristics of participant, 2020, Cambodia

**Variable**	**Overall (N=2360)**	**No T2D/HTN (N=1331)**	**HTN (N=761)**	**T2D (N=109)**	**T2D plus HTN (N=159)**	***P*-value**
		**n (%)**	**n (%)**	**n (%)**	**n (%)**	
Sex of participant						
Male	689 (29.2)	457 (34.3)	179 (23.5)	29 (26.6)	24 (15.1)	<0.001
Female	1671 (70.8)	874 (65.7)	582 (76.5)	80 (73.4)	135 (84.9)	
Age in years						
Range	40–96	40–96	40–90	40–81	40–82	
Mean (±SD)	58.5 (± 10.4)	56.0 (±10.3)	62.4 (±10.0)	57.6 (±8.4)	61.7 (±8.2)	<0.001
40–49	497 (21.1)	398 (29.9)	75 (9.9)	17 (15.6)	7 (4.4)	<0.001
50–59	803 (34.0)	464 (34.9)	231 (30.4)	46 (42.2)	62 (39.0)	
60 or older	1060 (44.9)	469 (35.2)	455 (59.8)	46 (42.2)	90 (56.6)	
Educational level						
No formal schooling	757 (32.1)	393 (29.5)	283 (37.2)	35 (32.1)	46 (28.9)	<0.016
Primary school	1308 (55.4)	755 (56.7)	398 (52.3)	61 (56.0)	94 (59.1)	
Secondary school or higher	295 (12.5)	183 (13.7)	80 (10.5)	13 (11.9)	19 (11.9)	
Having NSSF membership (yes)	114 (4.8)	62 (4.7)	36 (4.7)	6 (5.5)	10 (6.3)	0.806
Having HEF membership (yes)	434 (18.4)	247 (18.6)	143 (18.8)	18 (16.5)	26 (16.4)	0.849
Wealth quintile						
Poorest	441 (18.7)	261 (19.6)	140 (18.4)	16 (14.7)	24 (15.1)	0.050
Poor	447 (18.9)	263 (19.8)	139 (18.3)	18 (16.5)	27 (17.0)	
Medium	467 (19.8)	262 (19.7)	144 (18.9)	27 (24.8)	34 (21.4)	
Rich	480 (20.3)	244 (18.3)	176 (23.1)	16 (14.7)	44 (27.7)	
Richest	525 (22.2)	301 (22.6)	162 (21.3)	32 (29.4)	30 (18.9)	
Care model						
Coexisting	432 (18.3)	248 (18.6)	147 (19.3)	20 (18.3)	17 (10.7)	0.015

For peer review only

Community-based	480 (20.3)	276 (20.7)	153 (20.1)	18 (16.5)	33 (20.8)
Health center-based (high)	486 (20.6)	257 (19.3)	174 (22.9)	27 (24.8)	28 (17.6)
Health center-based (low)	518 (22.0)	292 (21.9)	170 (22.3)	18 (16.5)	38 (23.9)
Hospital-based	444 (18.8)	258 (19.4)	117 (15.4)	26 (23.9)	43 (27.0)

Abbreviations: SD, standard deviation; NSSF, National Social Security Fund; HEF, Health Equity Fund

Note: Health center-based (high) means the OD with high coverage (six out of nine) of health centers with the WHO PEN; Health center-based (low) means the OD with low coverage (six out of 25) of health centers with the WHO PEN

Public and private healthcare utilization

The 2,360 individuals reported 6,645 visits to the healthcare facilities in the three months preceding the survey, averaging 2.8 visits per person over three months.

Figure 2 presents the proportion of visits to public and private healthcare facilities. At the facility level, as shown in Figure 2A, the largest share was accounted for by private clinics (28.5%), followed by visits to the private homes of nurses or doctors (15.6%), private pharmacies (15.3%), health centers (12.6%), and private hospitals (11.6%).

The common public healthcare facilities used by participants with T2D and T2D plus HTN were provincial/national and district referral hospitals (Figure 2A). Approximately 29.7% of visits from people with T2D and 29.6% from people with T2D plus HTN went to provincial/national and district hospitals (Figure 2A). These proportions were higher than 6.9% for people without T2D or HTN and 6.1% for HTN only.

Overall, the private sector occupied about 78.0% of the total visits, and the public sector occupied 22.0% (Figure 2B). All groups visited private healthcare facilities more frequently than public healthcare facilities (Figure 2). However, the frequency of visiting public facilities was statistically higher in people with T2D and T2D plus HTN. As shown in Figure 2B, 37.2% of visits from people with T2D and 34.7% of visits from people with T2D plus HTN were to public healthcare facilities, compared with 18.9% of visits from people without the two conditions and 21.7% of the visits from people with HTN (P-value<0.001).

For peer review only

Public healthcare utilization by participant characteristics

Table 2 compares the public healthcare facility user rates defined as the proportion of public healthcare visits over total visits (public visits plus private visits). The user rates were disaggregated by participant characteristics. In this bivariate analysis, age, NSSF, HEF, wealth quintile, and care model were significantly associated with public healthcare utilization at a *P*-value<0.25 in at least one patient group. Therefore, we included these variables in the multiple negative binomial regressions.

Table 2. Proportions of visits to public facilities by participant characteristics, 2020, Cambodia

Variable	**Overall (N=6645)**	**No T2D/HTN (N=3467)**	**HTN (N=2345)**	**T2D (N=320)**	**T2D plus HTN (N=513)**
	**User rate (%)**	**User rate (%)**	**User rate (%)**	**User rate (%)**	**User rate (%)**
Sex					
Male	20.9	17.9	21.9	47.2	26.4
Female	23.7	20.7	23.2	33.3	37.6
P-value	0.936	0.803	0.879	0.552	0.847
Age in years					
40–49	17.5	14.8	27.4	23.8	32.1
50–59	24.3	22.4	19.2	35	51.4
60+	24.0	21.1	24.2	43.5	26.3
P-value	0.442	**0.096**	0.601	0.708	0.254
Educational level					
No schooling	21.9	20.1	19.3	37.5	39.6
Primary	22.9	18.8	23.2	45	34.6
Secondary/higher	25.5	22.6	33.1	—	29.5
P-value	0.998	0.981	0.606	0.92	0.932
Having NSSF membership					
No	22.2	19.9	21.2	37.5	33.3
Yes	35.9	17.1	52.4	31.6	66.7
P-value	0.512	0.4505	**0.165**	0.808	0.343
Having HEF membership					
No	19.8	18	18.3	34.2	29.5
Yes	35.7	27.5	39.5	55.6	65.1
P-value	**<0.001**	**0.01**	**0.014**	0.346	**0.04**
Household socio-economic class					
Poorest	28.2	20.4	33.5	54.5	58
Poor	24.9	23.5	23.3	27.3	42.7
Medium	22.3	15.2	25.7	36.6	37.5
Rich	22.1	20.5	19.9	50	30
Richest	18.1	18.9	14.8	29.9	16.5

For peer review only

P-value	0.029	0.419	**0.083**	0.966	**0.218**
Care model					
Coexisting	31.1	25.8	36.6	53.0	20.0
Community-based	19.5	18.0	13.6	56.8	39.2
Health center-based (high)	14.7	13.2	14.8	19.0	23.8
Health center-based (low)	21.4	21.5	16.6	46.7	32.7
Hospital-based	24.4	16.3	29.7	22.5	42.9
P-value	**0.002**	**0.007**	**0.018**	**0.172**	**0.162**

Abbreviation: NSSF, National Social Security Fund; HEF, Health Equity Fund

Note:

- *The % of private healthcare is not presented in this table but can it be calculated by subtracting the % of the public healthcare from 100%.*
- *Health center-based (high) means the OD with high coverage (six out of nine) of health centers with the WHO PEN; Health center-based (low) means the OD with low coverage (six out of 25) of health centers with the WHO PEN*
- *"N" denotes the total of visits.*
- *P-values<0.25 are in bold, indicating a significant level at 0.25. Variables with P-value<0.25 were included in multivariate analysis.*

Table 3 presents the results of the multiple negative binomial regressions. Overall, the significant increase in public healthcare use was associated with having T2D and T2D plus HTN, living in the OD with *coexisting care,* and HEF membership.

In people without T2D or HTN, HEF membership was significantly associated with public healthcare use: adjusted incidence rate ratio (AIRR) of 1.4 [95% CI 1.0–2.0], *P*-value<0.05). We did not observe the same association in the other three groups.

In people with HTN, the poorest category was significantly associated with increasing public healthcare use with an AIRR of 2.1 [95% CI 1.1–4.0], *P*-value=0.02), compared to those in the richest category. Nevertheless, the same association was not seen in other patient groups.

Regarding the care model, in people with T2D, the OD with *community-based care* (AIRR 3.7 [95% CI 1.2–11.3], *P*-value=0.019) and the OD with low coverage of *health center-based care* (AIRR 3.3 [95% CI 1.1–9.8], *P*-value=0.036) were significantly higher in public healthcare use than in the OD with *hospital-based care*.

For peer review only

In people with T2D plus HTN, the OD with *coexisting care* was associated with higher public healthcare use (AIRR 4.0 [95% CI 1.2–12.9], *P*-value=0.020).

Table 3. Factors associated with public healthcare use, 2020, Cambodia

	**Overall (N=2360)**	**No T2D/HTN (N=1331)**	**HTN (N=161)**	**T2D (N=109)**	**T2D plus HTN (N=759)**
Disease group	AIRR [95% CI]	AIRR [95% CI]	AIRR [95% CI]	AIRR [95% CI]	AIRR [95% CI]
No T2D/HTN	Ref.				
HTN	1.0 [0.8–1.2]	—	—	—	—
T2D	**1.9 [1.3–2.9]****	—	—	—	—
T2D plus HTN	**1.9 [1.3–2.7]*****	—	—	—	—
Age in year					
40–49	Ref.	Ref.	Ref.	Ref.	
50–59	1.2 [0.9–1.5]	1.4 [1.0–1.9]	0.6 [0.3–1.2]	1.5 [0.5–4.5]	1.2 [0.3–4.2]
60+	1.1 [0.8–1.4]	1.2 [0.9–1.7]	0.7 [0.4–1.3]	1.5 [0.5–4.5]	0.6 [0.2–2.2]
Having NSSF membership					
No	Ref.	Ref.	Ref.	Ref.	
Yes	1.4 [0.9–2.1]	1.0 [0.5–1.8]	2.0 [0.9–4.6]	2.3 [0.5–10.1]	1.9 [0.7–4.8]
Having HEF membership					
No		Ref.	Ref.	Ref.	
Yes	**1.4 [1.1–1.8]***	**1.4 [1.0–2.0]***	1.4 [0.9–2.3]	2.1 [0.8–5.1]	**1.9 [1.0–3.7]**
Household socio-economic class					
Poorest	**1.4 [1.0–2.0]***	1.0 [0.7–1.6]	**2.1 [1.1–4.0]***	1.2 [0.4–3.5]	2.6 [0.9–7.1]
Poor	1.2 [0.9–1.7]	1.1 [0.7–1.7]	1.2 [0.7–2.3]	0.7 [0.2–2.1]	2.6 [1.0–7.3]
Medium	1.1 [0.8–1.5]	0.9 [0.6–1.4]	1.2 [0.7–2.3]	0.9 [0.4–2.2]	3.0 [1.2–7.7]
Rich	1.1 [0.8–1.4]	1.1 [0.7–1.7]	1.0 [0.6–1.8]	1.2 [0.4–3.5]	1.7 [0.7–4.1]
Richest	Ref.	Ref.	Ref.	Ref.	Ref.
Care model					
Coexisting	**1.4 [1.0–1.9]***	1.5 [1.0–2.3]	1.3 [0.7–2.3]	2.5 [0.8–7.6]	**4.0 [1.2–12.9]***
Community-based	0.9 [0.7–1.2]	1.0 [0.6–1.5]	0.5 [0.3–1.0]	**3.7 [1.2–11.3]***	1.7 [0.5–6.1]
Health center-based (high)	0.8 [0.6–1.1]	0.8 [0.5–1.3]	0.7 [0.4–1.3]	1.3 [0.4–3.9]	2.7 [0.9–8.8]
Health center-based (low)	1.0 [0.8–1.4]	1.4 [1.0–2.1]	0.6 [0.3–1.0]	**3.3 [1.1–9.8]***	3.0 [1.0–9.1]
Hospital-based	Ref.	Ref.	Ref.	Ref.	Ref.

(*) P-value<0.05, (**) P-value<0.01, (***) P-value<0.001

Abbreviation: AIRR, adjusted incidence rate ratio; CI, confidence interval; Ref., reference group; OD, operational district

Note: Health center-based (high) means the OD with high coverage (six out of nine) of health centers with the WHO PEN; Health center-based (low) means the OD with low coverage (six out of 25) of health centers with the WHO PEN

For peer review only

Healthcare expenditure

Medical cost per year, overall and by facility

Overall, those who used healthcare spent an average of USD 25.3 [95% CI 22.9–27.6] for all healthcare services in the three months preceding the survey (Figure 3).

When comparing patient groups, people with T2D plus HTN had the highest healthcare expenditure with an average of USD 43.6 [95% CI 29.7–57.2], followed by people with T2D with an average of USD 34.0 [95% CI 25.5–42.6]. These expenditures were statistically higher than the average of USD 17.1 [95% CI 13.1–21.1] in people with HTN and the average of USD 26.9 [95% CI 23.9–29.9] in people without the two conditions with a *P*-value<0.001.

Table S1 shows the arithmetic mean of healthcare expenditure (Annexure 4). The arithmetic mean is the mean before the data log-transformation. Since our model's Risk Ratio (RR) in Table 4 is the geometric mean (after log-transformation) ratio, we presented the geometric mean in Table S2 (Annexure 4). In the bivariate analysis, sex, age, NSSF, HEF, wealth quintile, sector (public vs. private), and care model were statistically associated with healthcare expenditure in one or more patient groups with a *P*-value<0.25. These variables were included in the multiple linear regression.

Table 4 presents results from the multiple linear regression analyses. Overall, having T2D or T2D plus HTN, being female, having reported using both private and public healthcare, and living in the OD with *community-based care* was significantly associated with increased healthcare expenditure. In contrast, holding HEF membership and using public healthcare was significantly associated with healthcare expenditure reduction.

At the group level, in people without the two conditions, HEF membership was significantly associated with a reduction in healthcare expenditure with an adjusted risk ratio (ARR) of 0.7

For peer review only

[95% CI 0.5–0.8], *P*-value<0.001). The same association was seen in people with HTN (ARR of 0.8 [95% CI 0.6- 1.0], *P*-value<0.01), and in T2D plus HTN (ARR of 0.3 [95% CI 0.2–0.6], *P*-value<0.001). However, the association was not observed in people with T2D.

In people without the two conditions, using public healthcare was significantly associated with a reduction in the expenditure (ARR of 0.3 [95% CI 0.2–0.3], *P*-value<0.001). The association was also found in people with HTN (ARR 0.4 [95% CI 0.3–0.5], *P*-value<0.001).

People with T2D plus HTN who resided in the OD with *community-based care* were significantly associated with a higher expenditure with an ARR of 2.0 [95% CI 1.1–3.8], *P*-value<0.01) than those with *hospital-based care*.

Table 4 Factors associated with reducing or increasing healthcare expenditure in 2020, Cambodia

Variable	**Overall [N=2142]**	**No T2D/HTN [N=1187]**	**HTN [N=726]**	**T2D [N=98]**	**T2D plus HTN [N=139]**
	ARR [95% CI]	ARR [95% CI]	ARR [95% CI]	ARR [95% CI]	ARR [95% CI]
Disease group					
No T2D/HTN	Ref.	—	—	—	—
HTN	0.9 [0.7–1.0]	—	—	—	—
T2D	**2.1 [1.6–2.7]*** **	—	—	—	—
T2D plus HTN	**1.9 [1.5–2.4]*** **	—	—	—	—
Sex					
Male		Ref.	Ref.	Ref.	Ref.
Female	**1.2 [1.1–1.4]** **	**1.4 [1.2–1.7]*** **	1.0 [0.8–1.2]	0.7 [0.4–1.5]	1.5 [0.8–2.9]
Age in years					
40–49	Ref.	Ref.	Ref.	Ref.	
50–59	1.1 [0.9–1.3]	1.0 [0.8–1.2]	1.4 [1.0–2.0]	1.1 [0.5–2.4]	1.4 [0.5–3.7]
60+	1.0 [0.9–1.2]	1.1 [0.8–1.3]	1.3 [1.0–1.9]	0.9 [0.4–1.9]	1.0 [0.4–2.7]
Educational level					
No schooling	Ref.	Ref.	Ref.	Ref.	Ref.
Primary	1.0 [0.9–1.1]	1.1 [0.9–1.4]	0.8 [0.7–1.0]	0.9 [0.5–1.7]	1.1 [0.7–1.8]
Secondary/higher	1.1 [0.8–1.3]	1.2 [0.9–1.6]	0.9 [0.6–1.3]	—	1.6 [0.7–3.7]
Having NSSF membership					
No	Ref.	Ref.	Ref.	Ref.	Ref.
Yes	0.9 [0.6–1.2]	0.9 [0.6–1.4]	0.9 [0.6–1.4]	0.6 [0.2–2.0]	**0.4 [0.2–0.9]***
Having HEF membership					
No	Ref.	Ref.	Ref.	Ref.	Ref.

For peer review only

Yes	**0.7 [0.6–0.8]*** **	**0.7 [0.5–0.8]*** **	**0.8 [0.6–1.0]*** **	0.7 [0.3–1.6]	**0.3 [0.2–0.6]*** **
Household wealth quintile					
Poorest	1.1 [0.9–1.4]	1.3 [0.9–1.7]	1.1 [0.8–1.5]	0.5 [0.2–1.2]	1.1 [0.5–2.2]
Poor	1.1 [0.9–1.3]	1.1 [0.8–1.4]	1.2 [0.9–1.7]	0.8 [0.3–1.8]	0.6 [0.3–1.3]
Medium	1.1 [0.9–1.3]	1.1 [0.8–1.4]	1.2 [0.9–1.6]	0.8 [0.4–1.6]	0.9 [0.4–1.7]
Rich	1.2 [1.0–1.4]	1.3 [1.0–1.7]	1.2 [0.9–1.6]	0.7 [0.3–1.7]	0.9 [0.5–1.7]
Richest	Ref.	Ref.	Ref.	Ref.	Ref.
Healthcare sector					
Private	Ref.	Ref.	Ref.	Ref.	Ref.
Public	**0.3 [0.3–0.4]*** **	**0.3 [0.2–0.3]*** **	**0.4 [0.3–0.5]*** **	0.7 [0.4–1.2]	0.8 [0.5–1.3]
Both	**1.5 [1.1–1.9]*** **	**1.6 [1.1–2.4]** **	0.9 [0.4–1.7]	—	1.2 [0.3–5.4]
OD with different care					
Coexisting	1.0 [0.9–1.3]	1.0 [0.8–1.3]	1.0 [0.7–1.4]	1.0 [0.4–2.6]	1.7 [0.8–3.9]
Community-based	**1.4 [1.1–1.6]** **	1.2 [0.9–1.6]	1.3 [1.0–1.9]	1.3 [0.6–3.2]	**2.0 [1.1–3.8]** **
Health center-based [high]	1.0 [0.8–1.2]	1.0 [0.7–1.3]	1.1 [0.8–1.5]	0.9 [0.4–1.9]	1.3 [0.7–2.6]
Health center-based [low]	0.8 [0.7–1.0]	0.8 [0.6–1.0]	0.8 [0.6–1.1]	1.3 [0.5–3.0]	1.5 [0.8–2.6]
Hospital-based	Ref.	Ref.	Ref.	Ref.	Ref.

(*) P-value<0.05, (**) P-value<0.01, (***) P-value<0.001
Abbreviation: ARR, adjusted risk ratio; CI: confidence interval; Ref., reference group; OD, operational district
Note: Health center-based (high) means the OD with high coverage (six out of nine) of health centers with the WHO PEN; Health center-based (low) means the OD with low coverage (six out of 25) of health centers with the WHO PEN; P-values<0.05, <0.01, <0.001 are in bold, indicating the variables are significantly associated with expenditure

DISCUSSION

The results show that the use of the public healthcare system remains low for all groups in our study, with about one in every five healthcare visits taking place in the public sector overall. People with chronic conditions, HEF membership, living in the OD with *community-based care* contributed to public healthcare uptake. The healthcare expenditure was significantly reduced when patients used public healthcare services, regardless of HEF membership. However, the reduction in spending was more noticeable in people with HEF membership. In contrast, expenditure was higher among patients living in the OD with *community-based care.*

People in Cambodia predominantly used healthcare in private facilities for outpatient curative care.[19] Our study showed that this is also the case for people with chronic conditions such as

For peer review only

T2D and HTN, although this group had a slightly higher rate of using public healthcare services. This result is congruent with earlier findings that approximately 61% of T2D and/or HTN patients received their initial diagnosis in private settings.[6] A qualitative study in Cambodia suggested that people with T2D did not prefer diabetes services at public facilities because they were less accessible due to geographical factors or distance and limited medication supply.[23] Comparing our findings to other low- and middle-income countries is challenging due to differences in health system organization, government investment in health, and most studies focusing on general services rather than T2D and/or HTN services. Nevertheless, our findings are comparable to those from India, Nigeria, and Nepal, where government accounts for a very small share (<30%) of national health expenditure as well. [24] In India, 75% of outpatient visits were occupied by the private sector, similar to Nigeria (82%) and Napal (65%).[24] Cambodia and these three countries shared similar characteristics as the majority of the population relies on low-cost, and low-quality private services. Our findings clearly suggest that healthcare quality and access to public healthcare services is still below the expectations of people and the private providers have a role in filling the gaps. To move forward in the direction of universal health coverage, meaning that people can access the health services they need without financial hardship, Cambodia should focus on expanding quality service coverage for people with T2D and/or HTN at public healthcare facilities across the country. Expanding quality services at public healthcare facilities may be the best suited approach to the Cambodian context, where dual practice is strong and regulation weak. The literature suggests that the public and private providers are not mutually exclusive and they shape each other's characteristics or sometimes so-called competition for health benefits.[24 25] If public healthcare providers can provide quality services at affordable prices to the poor or those from low-income households, visits to private

For peer review only

healthcare providers, who offer inferior services at higher prices, will decrease . [24 25] The private healthcare providers will change their service provision to target the rich. [24 25] Previous studies revealed that HEF membership contributed to the health service uptake at public facilities and reduced healthcare expenditure in general users.[14 26] Our findings extended the understanding that HEF membership has also increased public healthcare use and substantially reduced healthcare spending among people with T2D and/or HTN. Since HEF benefits are only available in public healthcare facilities, it is not surprising that it also contributes to increasing service uptake in public facilities. The HEF is an important pillar of the Cambodian government's social security system and our findings suggest that HEF membership should be expanded to cover among people with chronic conditions. The Cambodian government recognized that the current social protection system has not yet covered those so-called “missing middle” between the poor, who are covered by the HEF, and those in formal employment, who are covered by the NSSF. Therefore, a new social health protection scheme targeting those in the informal economy and senior population without pensions, which accounts for 90% of people aged 60 years or older in Cambodia, should be created. This social protection scheme must go alongside with improving service coverage and quality. The success of such a model has been demonstrated by Thailand, a neighboring country of Cambodia.[27 28] Thailand focused on improving public healthcare services and introduced three public health insurance schemes. One of them was the Universal Coverage Scheme, which covered 75% of the Thai population.[27] Such a model might be too ambitious for Cambodia, since Thailand is more economically developed than Cambodia. However, this is still a model that Cambodia should be aiming for, so that quality health services for people with T2D and/or HTN will be more accessible.

For peer review only

Community-based care contributed to the higher public service uptake among T2D and/or HTN, but it also contributed to the higher expenditure for the users. In ODs with this model, PEs refer patients to the public referral hospitals, so it is not surprising that the public service uptake is slightly higher than other ODs.[29] However, it is somewhat surprising that people with T2D and/or HTN in the OD with *community-based care* spent more on their health services. It is unclear what the influencing factors are because a large proportion of service users (80%) used private services in this OD. Although, this may be partially explained by higher unit costs spent by the supply side in *community-based care* to operate their services, so the patients are charged a higher fee than other models. Our team had conducted a costing study in 2020 to examine the costs to operate services by different care models. The study found that the annual unit costs were higher for T2D and HTN patients in the *community-based care* than the *hospital-based care* (USD 101 vs. USD 77 for a T2D patient and USD 83 vs. USD 55 for an HTN patient). The higher unit costs in the *community-based care* were driven by adding PE components and field activities to the model while drugs and consultation fee are not subsidized. The investment in *community-based care* leads to better treatment outcomes, but it is not explored in our study. A previous study provided limited information that a significant proportion of patients in the *community-based care* network had achieved fasting blood glucose goals of 126 mg/dl, from 10% to 45%, and blood pressure goals of 140/90 mm Hg, from 58% to 67%, after a 12-month follow-up.[20] This study, however, did not have a control group (patients outside the network). From this, we can learn two things. First, the adapting and scaling up of PEs should be done with a careful budget plan as PEs incur operational costs. Second, a study investigating the treatment outcomes and cost-effectiveness between different care models should be conducted in order to

For peer review only

inform decision-making. We, therefore, cannot make a recommendation from this limited finding.

There are several strengths in our study. First, our study is among the few to examine healthcare utilization and expenditure both among people with T2D and/or HTN and people without the two conditions in Cambodia. It furthermore covers both the public and private sectors. This broad scope renders the results useful to inform T2D and HTN interventions in Cambodia. Second, we covered a wide range of ODs which are geographically diverse and comprising of different care models, which means that our participants are heterogeneous. The sampling design--randomizing villages, households, and household members--is robust within its scope, targeting the population in rural or semi-rural settings in Cambodia. Third, the data collection was robust and ensured a reliable data set.

Our study also had its limitations. First, it may not represent the national level as most of the study sites (villages) we selected were rural or semi-rural, which may lead to overestimating the healthcare utilization in public facilities. Second, the ODs were purposively selected with oversampling the OD with interventions, increasing the service uptake in public facilities. This may lead to overestimating the public healthcare use in our study. Third, we only calculated the healthcare expenditure for those who used the service in the three months preceding the survey, which cannot be generalized outside this period. However, it is unlikely to be significant because we focused more on factors associated with increasing or reducing healthcare expenditure. Fourth, the sample size for people with T2D only and people with T2D plus HTN may be relatively small. Therefore, variables that were not significantly associated with the dependent variables in these groups in our study may be due to the insufficient sample size.

For peer review only

CONCLUSION

Healthcare utilization at public healthcare facilities is relatively low for all groups; however, it is higher in people with chronic conditions. HEF membership and *community-based care* contributed the higher public healthcare utilization in people with chronic conditions. Using public healthcare services, regardless of HEF status, reduced the healthcare expenditure. However, the reduction in spending was more noticeable in people with HEF membership. To protect people with T2D and/or HTN from financial risk and move in the direction of universal health coverage, the public healthcare system should further improve care quality, and expand social health protection. Future research should link healthcare use and expenditure across different healthcare models to actual treatment outcomes to denote areas for further investment.

For peer review only

541 **ACKNOWLEDGMENTS**

542 We would like to thank all the participants in the study and all data collectors for carefully following the instructions.

544 **AUTHOR CONTRIBUTIONS**

545 SC, PI, VT, EW designed the study; SC analyzed data; SC, VT, VB, JO, SaC, SL, SY, WD, EW, PI wrote the manuscript.

547 **FUNDING**

548 This work was funded by the Belgian Government through the VLIR-UOS project (no award/grant number), the Fourth Funding Agreement (FA4) Programme (2017–2021) between the Belgian Directorate of Development Cooperation and the Institute of Tropical Medicine, Antwerp (no award/grant number), and the Horizon2020 Framework Program of the European Union, grant no 825432.

553 **CONFLICT OF INTERESTS**

554 All authors declare no conflict of interest.

555 **ETHICS APPROVAL**

556 The protocol was approved by the National Ethics Committee for Human Research (NECHR) on April 29, 2019 (No. 105 NECHR) and by the Institutional Review Board of Institute of Tropical Medicine (Antwerp) on October 25, 2019 (No. 1,323/19). All participants provided their consent and volunteered to take part in the study.

560 **DATA AVAILABILITY STATEMENT**

561 Data are available on reasonable request. Data are available on reasonable request to Dr. Por Ir (ipor@niph.org.kh).

For peer review only

For peer review only

REFERENCES

1. World Health Organization. Cardiovascular diseases, 2020. Available: http://origin.who.int/cardiovascular_diseases/en/ [Accessed January 16, 2022].

2. World Health Organization. Global report on diabetes, 2016. Available: https://www.who.int/publications/i/item/9789241565257 [Accessed January 16, 2022]; World Health Organization. Hypertension, 2019. Available: https://www.who.int/news-room/fact-sheets/detail/hypertension [Accessed January 16, 2022].

3. University of Health Sciences. National noncommunicable disease risk factor survey: STEPS survey 2016. Phnom Penh: University of Health Sciences, 2016.

4. World Health Organization. The Kingdom of Cambodia health system review, 2015. Available: https://apps.who.int/iris/bitstream/handle/10665/208213/9789290616917_eng.pdf?sequence=1&isAllowed=y [Accessed January 16, 2022].

5. University of Health Sciences (UHS) and the Preventive Medicine Department (DPM) of the Ministry of Health and World Health Organization (WHO). Prevalence of non-communicable disease risk factors in Cambodia: STEPS Survey, 2010. Available: https://www.who.int/ncds/surveillance/steps/2010_STEPS_Report_Cambodia.pdf [Accessed January 16, 2022]; University of Health Sciences (UHS) and the Preventive Medicine Department (DPM) of the Ministry of Health and World Health Organization (WHO). Prevalence of non-communicable disease risk factors in Cambodia: STEPS Survey, 2016.

For peer review only

6. Bigdeli M, Jacobs B, Men CR, et al. Access to Treatment for Diabetes and Hypertension in Rural Cambodia: Performance of Existing Social Health Protection Schemes. *PLoS One* 2016;11(1):e0146147.^

7. Ministry of Health - Cambodia. National standard operating procedure for diabetes and hypertension management in primary care 2019. Phnom Penh: MoH, 2019.

8. Rao M, van Pelt M, LoGerfo J, et al. Utilization of diabetes management health care services and its association with glycemic control among patients participating in a peer educator-based program in Cambodia. *PloS One* 2020;15(6):e0235037–e37.^

9. Department of Planning and Health Information. Health information system master plan 2016–2020. Phnom Penh: Cambodian Ministry of Health, 2017.

10. Health Policy Plus. A Legal and Regulatory Assessment of Private Healthcare Provision in Cambodia. Washington, DC, USA: Health Policy Plus, 2019.

11. Ministry of Health - Cambodia. Health Progress Report, 2018. Available: http://moh.gov.kh/content/uploads/2017/05/2019_MoH-Final01-Low.pdf [Accessed January 16, 2022].

12. Bureau-Point E, Baxerres C, Chheang S. Self-Medication and the Pharmaceutical System in Cambodia. *Med Anthropol* 2020;39(8):765–81.^

13. Jacobs B, Price N. The impact of the introduction of user fees at a district hospital in Cambodia. *Health Policy Plan* 2004;19(5):310–21.^

14. Annear PL, Tayu Lee J, Khim K, et al. Protecting the poor? Impact of the national health equity fund on utilization of government health services in Cambodia, 2006–2013. *BMJ Glob Health* 2019;4(6):e001679.^

For peer review only

15. Kaba MW, Baesel K, Poch B, et al. IDPoor: a poverty identification programme that enables collaboration across sectors for maternal and child health in Cambodia. *BMJ* 2018;363:k4698.^

16. National Social Security Fund (NSSF). Health Insurance Scheme, 2021. Available: https://www.nssf.gov.kh/default/health-care-scheme-2/ [Accessed January 16, 2022].

17. Kolesar RJ, Pheakdey S, Jacobs B, et al. Expanding social health protection in Cambodia: An assessment of the current coverage potential and gaps, and social equity considerations. *Int Soc Secur Rev* 2020;73(1):35–63.^

18. World Health Organization. Regional Office for the Western/Pacific. Cambodia national health accounts (2012–2016): health expenditure report, 2019. Available: http://iris.wpro.who.int/handle/10665.1/14362 [Accessed January 16, 2022].

19. World Health Organization. Regional Office for the Western/Pacific. Financial health protection in Cambodia (2009–2016): analysis of data from the Cambodia Socioeconomic Survey, 2019. Available: http://iris.wpro.who.int/handle/10665.1/14436 https://apps.who.int/iris/handle/10665/330701 [Accessed January 16, 2022].

20. Taniguchi D, LoGerfo J, van Pelt M, et al. Evaluation of a multi-faceted diabetes care program including community-based peer educators in Takeo province, Cambodia, 2007-2013. *PLoS One* 2017;12(9):e0181582.^

21. Janssens B, Van Damme W, Raleigh B, et al. Offering integrated care for HIV/AIDS, diabetes and hypertension within chronic disease clinics in Cambodia. *Bulletin of the World Health Organization* 2007;85(11):880-85.^

22. KoBoToolbox at the Harvard Humanitarian Initiative. KoBoToolbox, Available: https://www.kobotoolbox.org/#home [Accessed January 16, 2022].

For peer review only

23. Nang EEK, Dary C, Hsu LY, et al. Patients' and healthcare providers' perspectives of diabetes management in Cambodia: a qualitative study. *BMJ Open* 2019;9(11):e032578–e78.^

24. Mackintosh M, Channon A, Karan A, et al. What is the private sector? Understanding private provision in the health systems of low-income and middle-income countries. *The Lancet* 2016;388(10044):596-605.^

25. Morgan R, Ensor T, Waters H. Performance of private sector health care: implications for universal health coverage. *The Lancet* 2016;388(10044):606-12.^; McPake B, Hanson K. Managing the public–private mix to achieve universal health coverage. *The Lancet* 2016;388(10044):622-30.^; Horton R, Clark S. The perils and possibilities of the private health sector. *The Lancet* 2016;388(10044):540-41.^

26. Jithitikulchai T, Feldhaus I, Bauhoff S, et al. Health equity funds as the pathway to universal coverage in Cambodia: care seeking and financial risk protection. *Health Policy Plan* 2021;36(1):26–34.^

27. Tangcharoensathien V, Tisayaticom K, Suphanchaimat R, et al. Financial risk protection of Thailand's universal health coverage: results from series of national household surveys between 1996 and 2015. *International journal for equity in health* 2020;19(1):163.^

28. Tangcharoensathien V, Witthayapipopsakul W, Panichkriangkrai W, et al. Health systems development in Thailand: a solid platform for successful implementation of universal health coverage. *The Lancet* 2018;391(10126):1205-23.^

29. van Olmen J, Eggermont N, van Pelt M, et al. Patient-centred innovation to ensure access to diabetes care in Cambodia: the case of MoPoTsyo. *J Pharm Policy Pract* 2016;9(1):1.^

For peer review only

For peer review only

For peer review only - http://bmjopen.bmj.com/site/about/guidelines.xhtml

655 **Figure 1. Data flow from household selection to final dataset in this study,**
656 **Cambodia, 2020**

657

658 **Figure 2. Proportion of visits to public and private facilities in Cambodia, 2020**

659

660 **Figure 3. Healthcare expenditure by patient groups in the three months preceding**
661 **the survey in 2020, Cambodia**

662

663

664

For peer review only

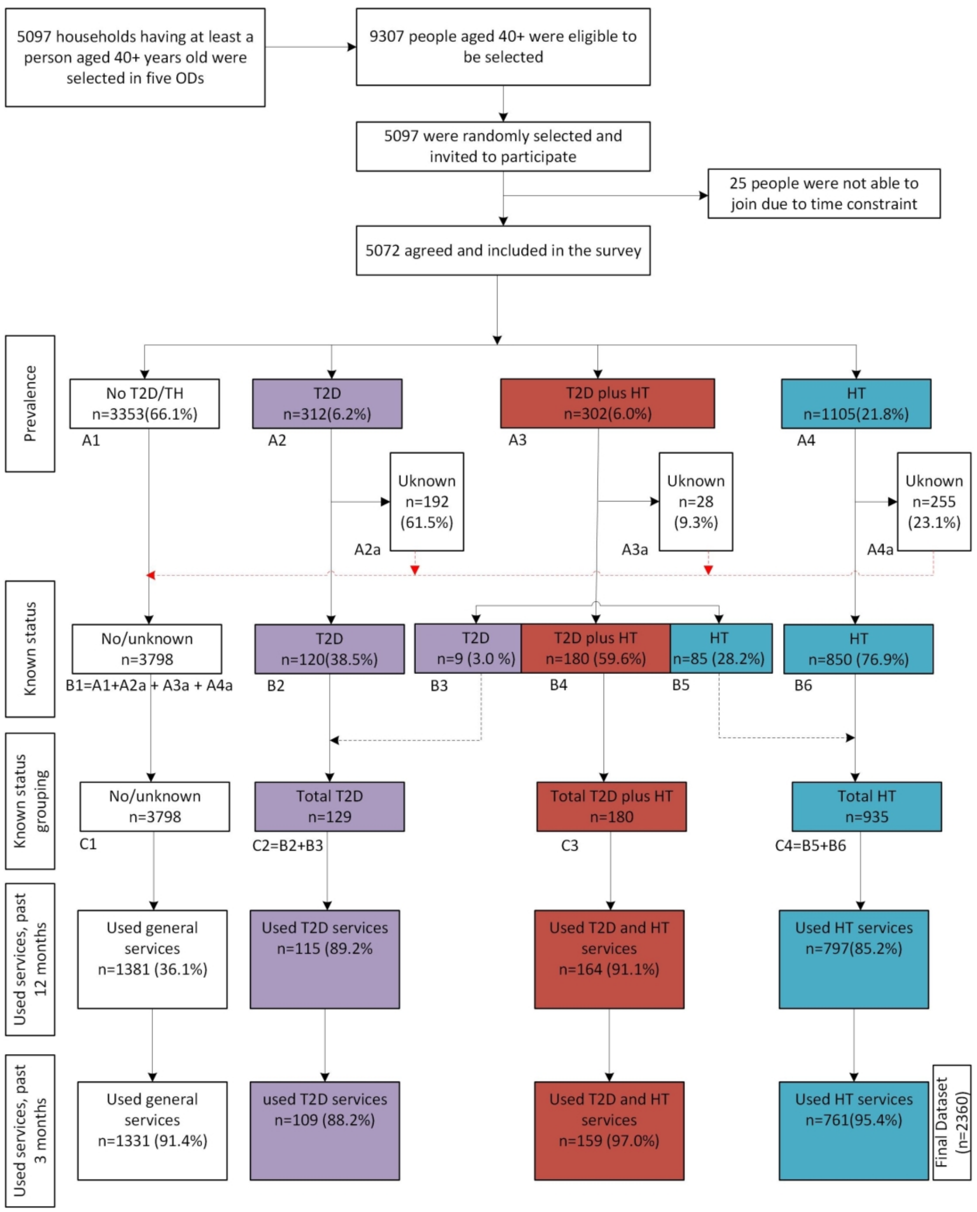

Figure 1. Data flow from household selection to final dataset in this study, Cambodia, 2020

199x244mm (300 x 300 DPI)

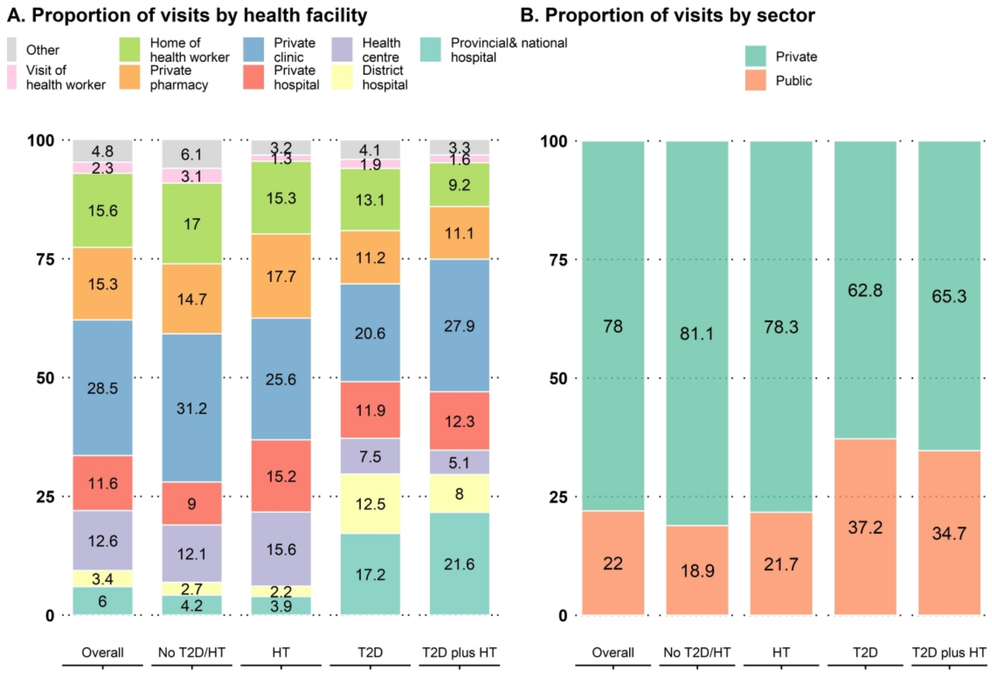

Figure 2. Proportion of visits to public and private facilities in Cambodia, 2020

254x177mm (600 x 600 DPI)

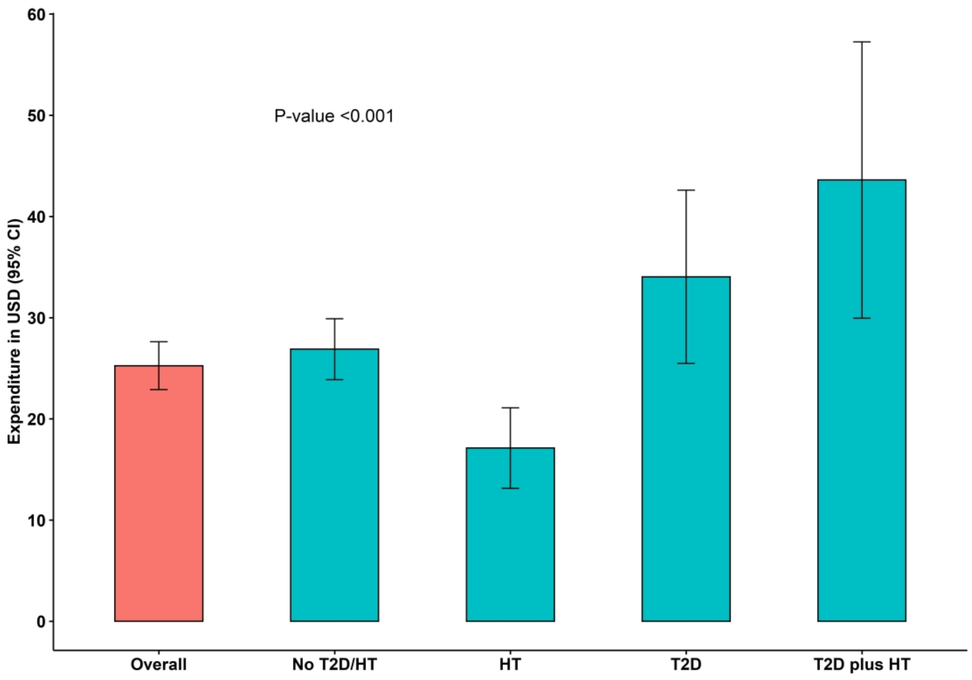

Figure 3. Healthcare expenditure by patient groups in the three months preceding the survey in 2020, Cambodia

254x177mm (600 x 600 DPI)

Annexure 1. Locations of the five studied ODs in Cambodia, 2020.

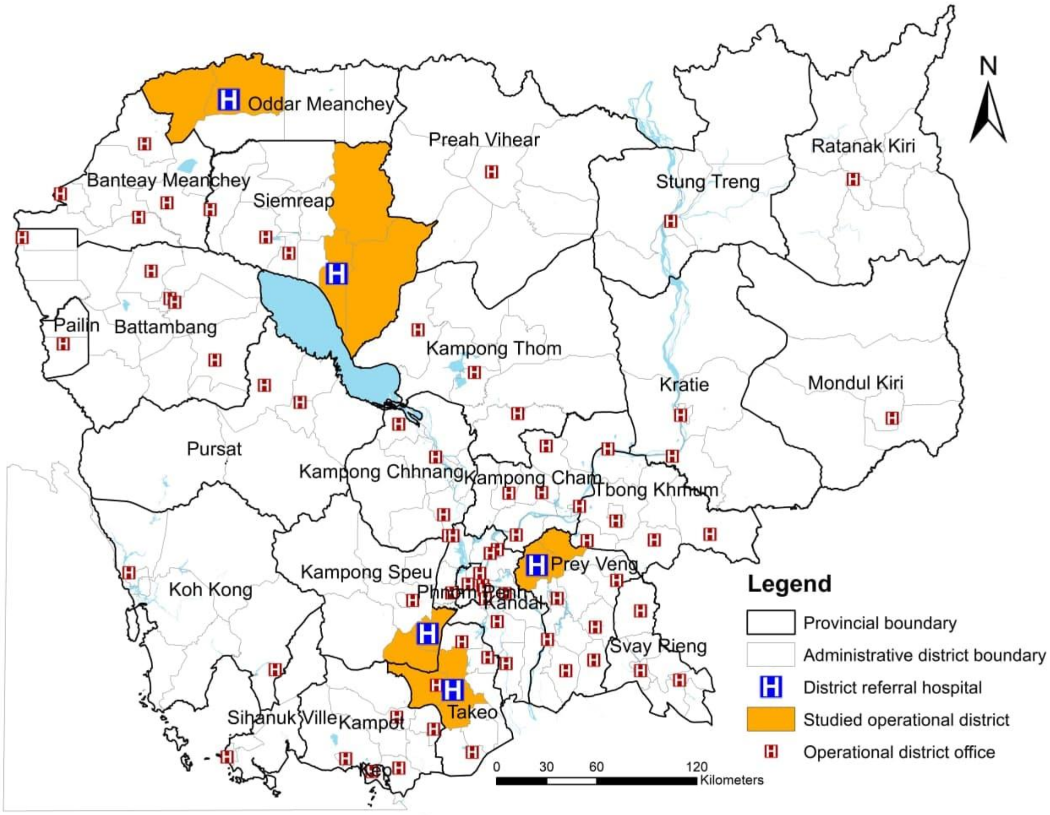

Annexure 2. Principal Component Analysis

The wealth quintile was used to classify participants into five socio-economic classes: poorest, poor, medium, rich, and richest. The wealth quintile was constructed using principal component analysis (PCA). The head of the family or family representative was interviewed using a 30-item tool that was taken from the 2014 Cambodia Demographic Health Survey. The tool is designed to classify households' wealth based on their properties. The binary answer questions were coded with 0 for no and 1 for yes. The questions with non-binary-answer were split into binary questions with a code of 0 for no and 1 for yes [21]. The first score component was used to classify the wealth into five quintiles [21]. The lower score represents the poorer status, and the higher score represents the richer status. The list of questions used for PCA is in the table below.

HOUSEHOLD SOCIO-ECONOMIC STATUS

QH3. Does your household have? [CDHS]		
301. electricity	1=Yes	0=No
302. a radio?	1=Yes	0=No
303. a television?	1=Yes	0=No
304. a mobile telephone?	1=Yes	0=No
305. If yes, is it a smartphone?	1=Yes	0=No
306. a refrigerator?	1=Yes	0=No
307. a wardrobe?	1=Yes	0=No
308. a sewing machine or loom?	1=Yes	0=No
309. CD/DVD player?	1=Yes	0=No
310. a generator/battery/solar panel?	1=Yes	0=No

Does any member of your household own/have ? [CDHS]		
311. a watch?	1=Yes	0=No

For peer review only

312. a bicycle or cyclo?	1=Yes	0=No
313. a motorcycle or motor-scooter?	1=Yes	0=No
314. a motorcycle-cart	1=Yes	0=No
315. a oxcart or horsecart?	1=Yes	0=No
316. a car or truck, tractor or van?	1=Yes	0=No
317. a boat with a motor?	1=Yes	0=No
318. a boat without a motor?	1=Yes	0=No
319. any agricultural land?	1=Yes	0=No
320. any livestock, herds, other farm animals, or poultry?	1=Yes	0=No
321. a bank account?	1=Yes	0=No

322	What is the main material of the floor?<br>*Record observation*	1. Earth/sand/clay<br>2. Dung<br>3. Wood planks<br>4. Palm/bamboo<br>5. Parquet or polished wood<br>6. Vinyl or Asphalt strips<br>7. Ceramic tiles<br>8. Cement tiles<br>9. Cement<br>10. Floating house<br>11. Other (specify)
323	What is the main material of the roof?<br>*Record observation*	1. Bamboo/thatch/palm leaf<br>2. Rustic mat<br>3. Wood planks<br>4. Cardboard<br>5. Plastic sheet<br>6. Metal<br>7. Wood<br>8. Calamine/cement fiber<br>9. Ceramic tiles<br>10. Clay tiles<br>11. Cement<br>12. Other (specify)
324	What is the main material of the exterior walls?<br>*Record observation*	1. Palm/bamboo/thach<br>2. Dirt<br>3. Bamboo with mud<br>4. Straw with mud<br>5. Stone with mud<br>6. Uncovered adobe

For peer review only

		7. Plywood<br>8. Cardboard<br>9. Reused wood<br>10. Metal<br>11. Cement<br>12. Stone with lime/cement<br>13. Bricks<br>14. Cement blocks<br>15. Covered adobe<br>16. Wood planks/shingles<br>17. Other (specify)
325	How many rooms in this household are used for sleeping?	Rooms:…………………
326	What is the main source of drinking water during the wet season for members of your household?	1. Piped into dwelling<br>2. Piped to yard/plot<br>3. Public tap/standpipe<br>4. Tube well or borehole<br>5. Protected well<br>6. Unprotected well<br>7. Protected spring<br>8. Unprotected spring<br>9. Rainwater<br>10. Tanker truck<br>11. Cart with small tank<br>12. Surface water (river/dam/lake/pond/stream/canal/irrigation channel<br>13. Bottled water<br>14. Other (specify)
327	What is the main source of drinking water during the dry season for members of your household?	1. Piped into dwelling<br>2. Piped to yard/plot<br>3. Public tap/standpipe<br>4. Tube well or borehole<br>5. Protected well<br>6. Unprotected well<br>7. Protected spring<br>8. Unprotected spring<br>9. Rainwater<br>10. Tanker truck<br>11. Cart with small tank<br>12. Surface water (river/dam/lake/pond/stream/canal/irrigation channel<br>13. Bottled water<br>14. Other (specify)

For peer review only

328	Do you do anything to the water to make it safer to drink?	1. Yes, always<br>2. Yes, sometime<br>3. No<br>4. Don't know
329	What do you usually do to make the water safer to drink?<br>*Record all mentioned.*	1. Boil<br>2. Add bleach/chlorine<br>3. Strain through a cloth<br>4. Use water filter (ceramic/sand/composite/etc.)<br>5. Solar disinfection<br>6. Let it stand and settle<br>7. Other (specify)
330	What kind of toilet facility do members of your household usually use?	1. Flush to piped sewer system (not shared with other households)<br>2. Flush to septic tank (not shared with other households)<br>3. No facility/bush/field<br>4. Other type of toilet (specify)

For peer review only

Annexure 3. Individual Survey Questionnaire

INTRO:

Hello, my name is ____and I am from the National Institute of Public Health. You has been randomly selected to participate in this study [on the scaling up of diabetes and hypertension in Cambodia] based on information from your household. The information you give will be kept confidential and no personal details will appear in any record. This interview will take approximately 60 minutes. You do not have to answer any question you don't want to and you can stop the interview at any time. There will also be measurements of your blood pressure, weight and height, waist and hip circumferences during and after the interview, and for tomorrow early morning we would like to test your fasting blood glucose [HbA1c and creatinine level for known and suspected diabetes cases]. We very appreciate your participation and information.

ELIGIBILITY & RECRUITMENT CHECK

(Instructions: To be filled in by data collectors before participants sign informed consent)

Q.N	Description & questions	RESPONSE		Ref:
Q1	Has the individual been a usual member of the household and stayed in the household the night before the interview or had not been absent for more than 6 months?	0 = No	1 = Yes	CDHS
Q2	Is the individual 40 or more than 40 of age?<br><br>*IF POSSIBLE, CHECK ID CARD*	0 = No	1 = Yes	WHO PEN SOP
	If NO, DO NOT continue.			
Q3	Is the individual physically and mentally capable to answer the questions?	0 = No	1 = Yes	
	If NO, DO NOT continue			
Q4	Are you willing to participate in the study?	0 = No	1 = Yes	
	If NO, DO NOT continue<br>If YES, CONSENT obtained			
**Q5. Consent obtained**		0 = No	1 = Yes	

SECTION 1: SOCIO-DEMOGRAPHIC INFORMATION

Q.N	Description & questions	RESPONSE	Type of Variable	Field	Ref:
Q6	How old are you?<br>-*Record in years as stated by the participant*<br>-*Record 99 if don't know*	____________Years	Quantitative discrete	Age	WHO STEPS
Q7	Sex of participant<br>*Record sex of the participant as observed*	1 = Male<br>2 = Female	Categorical binary	Sex	WHO STEPS
Q8	What is your marital status?	1=Married or living together<br>2=Divorced or separated<br>3=Widowed	Categorical nominal	Marital Status	CDHS

For peer review only - http://bmjopen.bmj.com/site/about/guidelines.xhtml

	-Record 88 if refuse to answer	4=Never married and never lived together			
Q9	What is your highest educational level?<br><br>*Record 99 if don't know*	1=No formal schooling<br>2=Less than primary school<br>3=Primary school<br>4=Secondary school<br>5=High school<br>6=College/University<br>7=Post graduate degree	Categorical ordinal	Educational level	WHO STEPS
Q10	What is your ethnic group?	1 = Khmer<br>2 = Vietnamese<br>3 = Chinese<br>4 = Cham (Muslim)<br>5 = Other	Categorical nominal	Ethnicity	WHO STEPS
Q11	Which of the following best describes your main work status within the past 12 months?<br><br>*Record 88 if refuse to answer*	1=Household tasks<br>2=Civil servant<br>3=Employee of private company/NGO<br>4=Self-employed farmer<br>5=Large-scale farmer with employees<br>6=Self-employed in small business<br>7=Running a big business with employees<br>8=Casual worker<br>9=Working abroad<br>10=At school (pupil/student)<br>11=Unemployed or not eligible<br>12=Retired	Categorical nominal	Occupation	CDHS
Q12	Taking the past year, can you give an estimate of your annual income if I read some options to you?<br><br>*Record 88 if refuse to answer*	1 = no earnings<br>2 = less than or 250 USD<br>3 = more than 251- 1500 USD<br>4 = more than 1501 – 3500 USD<br>5 = more than 3501 USD	Categorical ordinal	Individual income	WHO STEPS

**SECTION 2: HEALTH STATUS AND QUALITY OF LIFE**					
**Q.N**	**Description and Questions**	**Response**	**Type of Variable**	**Field**	**Ref:**

For peer review only - http://bmjopen.bmj.com/site/about/guidelines.xhtml

Q13	At this point of time in your life, how would you describe:<br><br>Q 13.1. Your home situation [____]<br>Q 13.2. Your family relationships [____]<br>Q 13.3. Your finances [____]<br>Q 13.4. Your work situation [____]<br><br>*-Record 99 if don't know and 88 if refuse*	1 = Excellent<br>2 = Very good<br>3 = Good<br>4 = Fair<br>5 = Poor	Categorical Ordinal	QoLife	GACD book
Q14	How good or bad is your health today?<br><br>*-The scale is numbered from 0 to 100.*<br>*-100 means the best health you can imagine.*<br>*-0 means the worst health you can imagine.*<br>*-Please mark an X on the scale to indicate how your health is today.*<br><br>*-Record 99 if don't know and 88 if refuse*	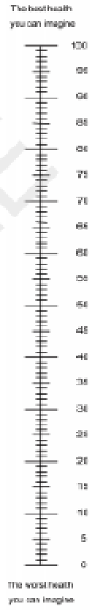	Quantitative discrete	Gen. Health	EuroQoL
Q15	Have you ever been told by a doctor or other health worker that you have hypertension?<br><br>*-Record 99 if don't know/unsure*	0 = No<br>1 = Yes	Categorical binary	HT_self-report diagnosis	WHO STEPS
Q16	Have you ever been told by a doctor or other health worker that you have diabetes?<br><br>*-Record 99 if don't know/unsure*	0 = No<br>1 = Yes	Categorical binary	DM_self-report diagnosis	WHO STEPS

For peer review only - http://bmjopen.bmj.com/site/about/guidelines.xhtml

Q17	Have you ever been told by a doctor or other health worker that you have heart problems?<br><br>*-Record 99 if don't know/unsure*	0 = No<br>1 = Yes	Categorical binary	Co-morbid ity (CVD)	WHO STEPS
Q18	Have you ever been told by a doctor or other health worker that you have symptoms suggestive of a stroke?<br><br>*-Record 99 if don't know/unsure*	0 = No<br>1 = Yes	Categorical binary	Co-morbid ity (CVD)	WHO STEPS
Q19	Have you ever been told by a doctor or other health worker that you have chronic kidney disease?<br><br>*-Record 99 if don't know/unsure*	0 = No<br>1 = Yes	Categorical binary	Co-morbid ity (CKD)	WHO STEPS
Q20	How many natural teeth do you have?<br><br>*-Record 99 if don't know*	0 = None<br>1 = 1-9 teeth<br>2 = 10-19 teeth<br>3 = 20 teeth or more	Categorical ordinal	Dental health	WHO Oral Health Question naire
Q21	During the past 12 months, did your teeth or mouth cause any pain or comfort?<br><br>*-Record 99 if don't know*	0 = No<br>1 = Yes	Categorical binary	Dental health	WHO Oral Health Question naire
Q22	Over the last 2 weeks, how often have you been bothered by any of the following problems?<br><br>Q 22.1. Little interest or pleasure in doing things [____]<br>Q 22.2. Feeling down, depressed, or hopeless. [____]<br>Q 22.3. Trouble falling or staying asleep, or sleeping too much. [____]	0 = Not at all<br>1 = Several days<br>2 = More than half the days<br>3 = Nearly everyday	Categorical ordinal	Mental health	PHQ-9

For peer review only - http://bmjopen.bmj.com/site/about/guidelines.xhtml

	Q 22.4. Feeling tired or having little energy. [____]<br>Q 22.5. Poor appetite or overeating [____]<br>Q 22.6. Feeling bad about yourself – or that you are a failure or make yourself or down your family [____]<br>Q 22.7. Trouble concentrating on things, such as reading the newspaper or watching television [____]<br>Q 22.8. Moving or speaking so slowly that other people could have noticed? Or the oppositebeing so fidgety or restless that you have been moving around a lot more than usual [____]<br>Q 22.9. Thoughts that you would be better off dead or of hurting yourself in some ways [____]				
Q42	We would like to confirm that:<br><br>*-This question is for categorizing respondents for the following sections and it is also important to ask respondents to confirm their main conditions in this survey.*<br><br>*-IF the answer is "0", go to Section 3*<br>*-IF the answer is "1", go to Section 3a*<br>*-IF the answer is "2", go to Section 3b*<br>*-IF the answer is "3", go to Section 3c*	0 = Neither hypertension nor diabetes<br><br>1 = Only hypertension<br><br>2 = Only diabetes<br><br>3 = Both diabetes and hypertension			

For peer review only - http://bmjopen.bmj.com/site/about/guidelines.xhtml

SECTION 3: HEALTH CARE UTILIZATION					
**Q.N**	**Description and Questions**	**Response**	**Type of Variable**	**Field**	**Ref:**
Q23	Have you sought medical treatment or advice as an outpatient from anyone in the past 3 months?<br><br>*-If No, go to Question 40.*	0 = No<br>1 = Yes	Categorical variable	Medical advice	GACD book
Q24	Where did you seek medical advice or treatment for illness in the past 3 months?<br><br>*-More than one answer can be selected.*<br>*-Data collectors can use probes to help respondents determine the types of health facilities in the Response Column.*<br>*-Record 99 if don't know and 88 if refuse*	1= National hospital (PP)<br>2= Provincial hospital (RH)<br>3= District hospital (RH)<br>4= Health centre<br>5= Health post<br>6= Provincial rehabilitation centre (PRC) or Community-based rehabilitation (CBR)<br>7= Other public; specify:<br>8= Private hospital<br>9= Private clinic<br>10= Private pharmacy<br>11= Home/Office of trained health worker/nurse<br>12= Visit of trained health worker/nurse<br>13= Other private medical; specify:<br>14= Shop selling drugs/market<br>15= Kru Khmer/ Magician	Categorical Nominal	Care provider_3M	CDHS and GACD book

For peer review only - http://bmjopen.bmj.com/site/about/guidelines.xhtml

		16= Monk/religious leader<br>17= Traditional birth attendant<br>18= Oversee medical service<br>19= Other; specify:			
From Q25-Q39, it is a set of questions that are asked following choices selected in Q24. If 2 or 3 choices were selected in Q24, Q25-Q39 would appear 2 or 3 times, accordingly.					
Q25	How many times did you visit the selected place**(s)** in Q24 in the past three months?	________ times	Quantitative discrete	Number of visits	GACD
Q26	How much in total was spent on the treatment at the selected place(s) in Q24?<br><br>*-Record 99 if don't know and 88 if refuse*	0 = free/no cost<br>1 = in kind<br>2 = _________Riels	-Categorical nominal<br>-Quantitative Continuous	Cost of treatment	CDHS
Q27	How did you pay for the treatment cost at the selected place(s) in Q24?<br><br>*-Record 99 if don't know and 88 if refuse*	1= Health Equity Fund<br>2= Voucher<br>3= Fee Exemption<br>4= NGO<br>5= National Social Security Fund<br>6= Community-Based Health Insurance<br>7= Health Insurance through Employer<br>8= Other Privately Purchased Commercial Health Insurance	-Categorical nominal	Payment method	GADC adapted to context specific

For peer review only - http://bmjopen.bmj.com/site/about/guidelines.xhtml

		9= Wage/income<br>10= Loan/ Ton Tin<br>11= Sale of Assets<br>12= Gift from Relative<br>13= Savings<br>14= Other			
Q28	How much in total was spent on transport to go to and return from the selected place(s) in Q24?<br><br>-*Record 99 if don't know and 88 if refuse*	0 = free/no cost<br>1 = in kind<br>2 = _________Riels	-Categorical nominal<br>-<br>Quantitative Continuous	Cost of transport	CDHS
Q29	On average how many hours do you spend to get treatment/advices from the selected place(s) in Q24?<br><br>-*Record 99 if don't know and 88 if refuse*	_________Hours	Quantitative continuous	Time spending	GACD book (Adapted)
Q30	How satisfied are you with the effect of your {treatment/care} at the selected place(s) in Q24?<br><br>-*Record 99 if don't know/unsure*	0 = Very satisfied<br>1 = Satisfied<br>2 = Neither satisfied nor dissatisfied<br>3 = Dissatisfied<br>4 = Very dissatisfied	Categorical ordinal	Patient satisfaction of care services	SAPS 2006
Q31	How satisfied are you with the explanations the {doctor/other health professional} has given you about the results of your {treatment/care} at the selected place(s) in Q24?<br><br>-*Record 99 if don't know/unsure*	0 = Very satisfied<br>1 = Satisfied<br>2 = Neither satisfied nor dissatisfied<br>3 = Dissatisfied<br>4 = Very dissatisfied	Categorical ordinal	Patient satisfaction of care services	SAPS 2006

For peer review only - http://bmjopen.bmj.com/site/about/guidelines.xhtml

Q32	The {doctor/other health professional} at the selected place(s) in Q24 was very careful to check everything when examining you.<br><br>*-Record 99 if don't know/unsure*	0 = Strongly agree<br>1 = Agree<br>2 = Not sure<br>3 = Disagree<br>4 = Strongly disagree	Categorical ordinal	Patient satisfaction of care services	SAPS 2006
Q33	At the selected place(s) in Q24, how satisfied were you with the choices you had in decisions affecting your health care?<br><br>*-Record 99 if don't know/unsure*	0 = Very satisfied<br>1 = Satisfied<br>2 = Neither satisfied nor dissatisfied<br>3 = Dissatisfied<br>4 = Very dissatisfied	Categorical ordinal	Patient satisfaction of care services	SAPS 2006
Q34	How much of the time did you feel respected by the {doctor/other health professional} at the selected place(s) in Q24?<br><br>*-Record 99 if don't know/unsure*	0 = All of the time<br>1 = Most of the time<br>2 = About half the time<br>3 = Some of the time<br>4 = None of the time	Categorical ordinal	Patient satisfaction of care services	SAPS 2006
Q35	At the selected place(s) in Q24, the time you had with the {doctor/other health professional} was too short.<br><br>*-Record 99 if don't know/unsure*	0 = Strongly agree<br>1 = Agree<br>2 = Not sure<br>3 = Disagree<br>4 = Strongly disagree	Categorical ordinal	Patient satisfaction of care services	SAPS 2006
Q36	Are you satisfied with the care you received in the selected place(s) in Q24?<br><br>*-Record 99 if don't know/unsure*	0 = Very satisfied<br>1 = Satisfied<br>2 = Neither satisfied nor dissatisfied<br>3 = Dissatisfied<br>4 = Very dissatisfied	Categorical ordinal	Patient satisfaction of care services	SAPS 2006

For peer review only - http://bmjopen.bmj.com/site/about/guidelines.xhtml

Q37	Did you get your blood pressure measured at the selected place(s) in Q24?<br><br>*-Record 99 if don't know/unsure*	0 = No<br>1 = Yes	Categorical variable	Access to blood pressure testing	Opinion
Q38	Did you get your blood glucose tested at the selected place(s) in Q24?<br><br>*-Record 99 if don't know/unsure*	0 = No<br>1 = Yes	Categorical variable	Access to blood glucose testing	Opinion
Q39	Would you recommend the selected place(s) in Q24 to others?<br><br>*-Record 99 if don't know/unsure*	1 = Not recommended<br>2 = Recommend with reservations<br>3 = Recommend<br>4 = Highly recommend	Categorical ordinal	Patient satisfaction of care services	Opinion
Q40	Have you ever had your blood glucose tested in the last three years?<br><br>*-Record 99 if don't know/unsure*	0 = No<br>1 = Yes	Categorical binary	Testing DM (CoC)	Opinion
Q41	Have you ever had your blood pressure measured in the last three years?<br><br>*-Record 99 if don't know/unsure*	0 = No<br>1 = Yes	Categorical binary	Testing HT (CoC)	Opinion

For peer review only - http://bmjopen.bmj.com/site/about/guidelines.xhtml

SECTION 3a: HEALTH CARE UTILIZATION FOR HYPERTENSION

Q.N	Description and Questions	Response	Type of Variable	Field	Ref:
Q43a	How long have you lived with hypertension?<br><br>*-Record 99 if don't know/unsure and 88 if refuse*<br>*-Less than a year is rounded up to one year*<br>*-Standard rounded up formula is applied.*	________Years	Quantitative concrete	Duration HT	Opinion
Q44a	Where were you first diagnosed as having hypertension?<br><br>*-Record 99 if don't know and 88 if refuse*	1= National hospital (PP)<br>2= Provincial hospital (RH)<br>3= District hospital (RH)<br>4= Health centre<br>5= Health post<br>6= Provincial rehabilitation centre (PRC) or Community-based rehabilitation (CBR)<br>7= Other public; specify:<br>8= Private hospital<br>9= Private clinic<br>10= Private pharmacy<br>11= Home/Office of trained health worker/nurse<br>12= Visit of trained health worker/nurse<br>13= Other private medical; specify:<br>14= Shop selling drugs/market<br>15= Kru Khmer/ Magician	Categorical Nominal	Diagnosis HT (CoC)	Opinion

For peer review only - http://bmjopen.bmj.com/site/about/guidelines.xhtml

		16= Monk/religious leader<br>17= Traditional birth attendant<br>18= Oversee medical service<br>19= MoPoTsyo<br>20= Other; specify:			
Q45a	Where did you first seek advice or treatment for hypertension after being diagnosed?<br><br>*-Record 99 if don't know and 88 if refuse*	1= National hospital (PP)<br>2= Provincial hospital (RH)<br>3= District hospital (RH)<br>4= Health centre<br>5= Health post<br>6= Provincial rehabilitation centre (PRC) or Community-based rehabilitation (CBR)<br>7= Other public; specify:<br>8= Private hospital<br>9= Private clinic<br>10= Private pharmacy<br>11= Home/Office of trained health worker/nurse<br>12= Visit of trained health worker/nurse<br>13= Other private medical; specify:<br>14= Shop selling drugs/market<br>15= Kru Khmer/ Magician<br>16= Monk/religious leader<br>17= Traditional birth attendant	Categorical Nominal	Link to care HT (CoC)	CDHS (Adapted for disease specific)

For peer review only - http://bmjopen.bmj.com/site/about/guidelines.xhtml

		18= Oversee medical service<br>19= MoPoTsyo<br>20= Other; specify:			
Q46a	Did you go to other places for follow up treatment/care for your hypertensive conditions?<br><br>*-Record 88 if refuse*<br>*-If NO, please skip Q47a*	0 = No<br>1 = Yes	Categorical binary	Trust HT	CDHS
Q47a	If yes to Q46a, where else did you go to get follow up treatment/care for your hypertensive conditions?	1= National hospital (PP)<br>2= Provincial hospital (RH)<br>3= District hospital (RH)<br>4= Health centre<br>5= Health post<br>6= Provincial rehabilitation centre (PRC) or Community-based rehabilitation (CBR)<br>7= Other public; specify:<br>8= Private hospital<br>9= Private clinic<br>10= Private pharmacy<br>11= Home/Office of trained health worker/nurse<br>12= Visit of trained health worker/nurse<br>13= Other private medical; specify:<br>14= Shop selling drugs/market	Categorical Nominal	Link to care HT (CoC)	CDHS (Adapted for disease specific)

For peer review only - http://bmjopen.bmj.com/site/about/guidelines.xhtml

		15= Kru Khmer/ Magician<br>16= Monk/religious leader<br>17= Traditional birth attendant<br>18= Oversee medical service<br>19= MoPoTsyo<br>20= Other; specify:			
Q48a	Did you get treatment/care for your hypertensive conditions in the past **12 months?**<br><br>*-Record 88 if refuse*<br>*-If NO, please skip Q49a-64a*	0 = No<br>1 = Yes	Categorical binary	Retain in care HT (CoC)	CDHS (Adapted for disease specific)
Q49a	Are you currently receiving any of the following treatment/advices for your hypertensive conditions prescribed by a doctor or other health care worker?<br><br>Q 49.1a. Drugs (medication) that you have taken in the past two weeks [____]<br>Q 49.2a. Advice to reduce salt intake [___]<br>Q 49.3a. Advice or treatment to lose weight [____]<br>Q 49.4a. Advice or treatment to stop smoking [____]<br>Q 49.5a. Advice to start or do more physical exercise [____]<br><br>*-Record 99 if don't know and 88 if refuse*	0 = No 1= Yes	Categorical ordinal	In treatment for HT (CoC)	STEPS Survey
Q50a	Have you had your blood cholesterol measured in the past 12 months?<br><br>*-Record 99 if don't know and 88 if refuse*	0 = No 1= Yes	Categorical ordinal	In treatment for HT (CoC)	Veerle's suggestion

For peer review only - http://bmjopen.bmj.com/site/about/guidelines.xhtml

Q51a	Where did you seek medical advice or treatment for your hypertensive condition in **the past 3 months**?<br><br>*-**More than one answer can be selected**.*<br>*-Data collectors can use probes to help respondents determine the types of health facilities in the Response Column.*<br>*-Record 99 if don't know and 88 if refuse*<br>*-If (21=no wehere), go to Q65a*	1= National hospital (PP)<br>2= Provincial hospital (RH)<br>3= District hospital (RH)<br>4= Health centre<br>5= Health post<br>6= Provincial rehabilitation centre (PRC) or Community-based rehabilitation (CBR)<br>7= Other public; specify:<br>8= Private hospital<br>9= Private clinic<br>10= Private pharmacy<br>11= Home/Office of trained health worker/nurse<br>12= Visit of trained health worker/nurse<br>13= Other private medical; specify:<br>14= Shop selling drugs/market<br>15= Kru Khmer/ Magician<br>16= Monk/religious leader<br>17= Traditional birth attendant<br>18= Oversee medical service<br>19= MoPoTsyo<br>20= Other; specify:	Categorical Nominal	Care provider_3M	CDHS and GACD book

For peer review only

For peer review only - http://bmjopen.bmj.com/site/about/guidelines.xhtml

		21= No where			
From Q52a-Q64a, it is a set of questions that are asked following choices selected in Q51a. If 2 or 3 choices were selected in Q51a, Q52a-Q64a would appear 2 or 3 times, accordingly.					
Q52a	How many times did you visit the selected place**(s)** in Q51a in the past three months?	________ times	Quantitative discrete	Numb er of visits	GACD
Q53a	How much in total was spent on the treatment at the selected place(s) in Q51a?<br><br>*-Record 99 if don't know and 88 if refuse*	0 = free/no cost<br>1 = in kind<br>2 = _________Riels	-Categorical nominal<br>-<br>Quantitative Continuous	Cost of treatm ent	CDHS
Q54a	How did you pay for the treatment cost at the selected place(s) in Q51a?<br><br>*-Record 99 if don't know and 88 if refuse*	1= Health Equity Fund<br>2= Voucher<br>3= Fee Exemption<br>4= NGO<br>5= National Social Security Fund<br>6= Community-Based Health Insurance<br>7= Health Insurance through Employer<br>8= Other Privately Purchased Commercial Health Insurance	-Categorical nominal	Paym ent metho d	GADC adapted to context specific

For peer review only - http://bmjopen.bmj.com/site/about/guidelines.xhtml

		9= Wage/income<br>10= Loan/ Ton Tin<br>11= Sale of Assets<br>12= Gift from Relative<br>13= Savings<br>14= Other			
Q55a	How much in total was spent on transport to go to and return from the selected place(s) in Q51a?<br><br>-*Record 99 if don't know and 88 if refuse*	0 = free/no cost<br>1 = in kind<br>2 = ________Riels	-Categorical nominal<br>-<br>Quantitative Continuous	Cost of transport	CDHS
Q56a	On average how many hours do you spend to get treatment/advices from the selected place(s) in Q51a?<br><br>-*Record 99 if don't know and 88 if refuse*	________Hours	Quantitative continuous	Time spending	GACD book (Adapted)
Q57a	How satisfied are you with the effect of your {treatment/care} at the selected place(s) in Q51a?<br><br>-*Record 99 if don't know/unsure*	0 = Very satisfied<br>1 = Satisfied<br>2 = Neither satisfied nor dissatisfied<br>3 = Dissatisfied<br>4 = Very dissatisfied	Categorical ordinal	Patient satisfaction of care services	SAPS 2006
Q58a	How satisfied are you with the explanations the {doctor/other health professional} has given you about the results of your {treatment/care} at the selected place(s) in Q51a?<br><br>-*Record 99 if don't know/unsure*	0 = Very satisfied<br>1 = Satisfied<br>2 = Neither satisfied nor dissatisfied<br>3 = Dissatisfied<br>4 = Very d issatisfied	Categorical ordinal	Patient satisfaction of care services	SAPS 2006

For peer review only - http://bmjopen.bmj.com/site/about/guidelines.xhtml

Q59a	The {doctor/other health professional} at the selected place(s) in Q51a was very careful to check everything when examining you.<br><br>*-Record 99 if don't know/unsure*	0 = Strongly agree<br>1 = Agree<br>2 = Not sure<br>3 = Disagree<br>4 = Strongly disagree	Categorical ordinal	Patient satisfaction of care services	SAPS 2006
Q60a	At the selected place(s) in Q51a, how satisfied were you with the choices you had in decisions affecting your health care?<br><br>*-Record 99 if don't know/unsure*	0 = Very satisfied<br>1 = Satisfied<br>2 = Neither satisfied nor dissatisfied<br>3 = Dissatisfied<br>4 = Very dissatisfied	Categorical ordinal	Patient satisfaction of care services	SAPS 2006
Q61a	How much of the time did you feel respected by the {doctor/other health professional} at the selected place(s) in Q51a?<br><br>*-Record 99 if don't know/unsure*	0 = All of the time<br>1 = Most of the time<br>2 = About half the time<br>3 = Some of the time<br>4 = None of the time	Categorical ordinal	Patient satisfaction of care services	SAPS 2006
Q62a	At the selected place(s) in Q51a, the time you had with the {doctor/other health professional} was too short.<br><br>*-Record 99 if don't know/unsure*	0 = Strongly agree<br>1 = Agree<br>2 = Not sure<br>3 = Disagree<br>4 = Strongly disagree	Categorical ordinal	Patient satisfaction of care services	SAPS 2006
Q63a	Are you satisfied with the care you received in the selected place(s) in Q51a?<br><br>*-Record 99 if don't know/unsure*	0 = Very satisfied<br>1 = Satisfied<br>2 = Neither satisfied nor dissatisfied<br>3 = Dissatisfied<br>4 = Very dissatisfied	Categorical ordinal	Patient satisfaction of care services	SAPS 2006

For peer review only - http://bmjopen.bmj.com/site/about/guidelines.xhtml

Q64a	Would you recommend the selected place(s) in Q51a to others?<br><br>*-Record 99 if don't know/unsure*	1 = Not recommend<br>2 = Recommend with reservations<br>3 = Recommend<br>4 = Highly recommend	Categorical ordinal	Patient satisfaction of care services	Opinion
Q65a	Have you ever been told by a doctor that you have eyes problems?<br><br>*-Record 99 if don't know/unsure*	0 = No<br>1 = Yes	Quantitative discrete	Complication-HT	Cambodia national guideline
Q66a	Have you ever been told by a doctor that you have kidney problems?<br><br>*-Record 99 if don't know/unsure*	0 = No<br>1 = Yes	Quantitative discrete	Complication-HT	Cambodia national guideline
Q67a	Have you ever been told by a doctor that you have lost the sensation of your peripheral membrane, such as foot, hand, arm?<br><br>*-Record 99 if don't know/unsure*	0 = No<br>1 = Yes	Quantitative discrete	Complication-HT	Cambodia national guideline
	In **the past 3 months**, besides seeking medical advice or treatment for your hypertensive condition, have you sought medical treatment or advice for other illnesses or conditions?<br><br>If No, go to Section 4.	0 = No<br>1 = Yes			

For peer review only - http://bmjopen.bmj.com/site/about/guidelines.xhtml

Q24	Where did you seek medical advice or treatment for that illness in the past 3 months?<br><br>*-More than one answer can be selected.*<br>*-Data collectors can use probes to help respondents determine the types of health facilities in the Response Column.*<br>*-Record 99 if don't know and 88 if refuse*	1= National hospital (PP)<br>2= Provincial hospital (RH)<br>3= District hospital (RH)<br>4= Health centre<br>5= Health post<br>6= Provincial rehabilitation centre (PRC) or Community-based rehabilitation (CBR)<br>7= Other public; specify:<br>8= Private hospital<br>9= Private clinic<br>10= Private pharmacy<br>11= Home/Office of trained health worker/nurse<br>12= Visit of trained health worker/nurse<br>13= Other private medical; specify:<br>14= Shop selling drugs/market<br>15= Kru Khmer/ Magician<br>16= Monk/religious leader<br>17= Traditional birth attendant<br>18= Oversee medical service<br>19= Other; specify:	Categorical Nominal	Care provider_3M	CDHS and GACD book
From Q25-Q39, it is a set of questions that are asked following choices selected in Q24. If 2 or 3 choices were selected in Q24, Q25-Q39 would appear 2 or 3 times, accordingly.					

Q25	How many times did you visit the selected place**(s)** in Q24 in the past three months?	_______ times	Quantitative discrete	Number of visits	GACD
Q26	How much in total was spent on the treatment at the selected place(s) in Q24?<br><br>*-Record 99 if don't know and 88 if refuse*	0 = free/no cost<br>1 = in kind<br>2 = ________Riels	-Categorical nominal<br>-Quantitative Continuous	Cost of treatment	CDHS
Q27	How did you pay for the treatment cost at the selected place(s) in Q24?<br><br>*-Record 99 if don't know and 88 if refuse*	1= Health Equity Fund<br>2= Voucher<br>3= Fee Exemption<br>4= NGO<br>5= National Social Security Fund<br>6= Community-Based Health Insurance<br>7= Health Insurance through Employer<br>8= Other Privately Purchased Commercial Health Insurance<br>9= Wage/income<br>10= Loan/ Ton Tin<br>11= Sale of Assets<br>12= Gift from Relative<br>13= Savings<br>14= Other	-Categorical nominal	Payment method	GADC adapted to context specific

For peer review only - http://bmjopen.bmj.com/site/about/guidelines.xhtml

Q28	How much in total was spent on transport to go to and return from the selected place(s) in Q24?<br><br>*-Record 99 if don't know and 88 if refuse*	0 = free/no cost<br>1 = in kind<br>2 = ________Riels	-Categorical nominal<br>-Quantitative Continuous	Cost of transport	CDHS
Q29	On average how many hours do you spend to get treatment/advices from the selected place(s) in Q24?<br><br>*-Record 99 if don't know and 88 if refuse*	________Hours	Quantitative continuous	Time spending	GACD book (Adapted)
Q30	How satisfied are you with the effect of your {treatment/care} at the selected place(s) in Q24?<br><br>*-Record 99 if don't know/unsure*	0 = Very satisfied<br>1 = Satisfied<br>2 = Neither satisfied nor dissatisfied<br>3 = Dissatisfied<br>4 = Very dissatisfied	Categorical ordinal	Patient satisfaction of care services	SAPS 2006
Q31	How satisfied are you with the explanations the {doctor/other health professional} has given you about the results of your {treatment/care} at the selected place(s) in Q24?<br><br>*-Record 99 if don't know/unsure*	0 = Very satisfied<br>1 = Satisfied<br>2 = Neither satisfied nor dissatisfied<br>3 = Dissatisfied<br>4 = Very dissatisfied	Categorical ordinal	Patient satisfaction of care services	SAPS 2006
Q32	The {doctor/other health professional} at the selected place(s) in Q24 was very careful to check everything when examining you.<br><br>*-Record 99 if don't know/unsure*	0 = Strongly agree<br>1 = Agree<br>2 = Not sure<br>3 = Disagree<br>4 = Strongly disagree	Categorical ordinal	Patient satisfaction of care services	SAPS 2006

For peer review only - http://bmjopen.bmj.com/site/about/guidelines.xhtml

Q33	At the selected place(s) in Q24, how satisfied were you with the choices you had in decisions affecting your health care?<br><br>*-Record 99 if don't know/unsure*	0 = Very satisfied<br>1 = Satisfied<br>2 = Neither s atisfied nor dissatisfied<br>3 = Dissatisfied<br>4 = Very dissatisfied	Categorical ordinal	Patien t satisfa ction of care servic es	SAPS 2006
Q34	How much of the time did you feel respected by the {doctor/other health professional} at the selected place(s) in Q24?<br><br>*-Record 99 if don't know/unsure*	0 = All of the time<br>1 = Most of the time<br>2 = About half the time<br>3 = Some of the time<br>4 = None of the time	Categorical ordinal	Patien t satisfa ction of care servic es	SAPS 2006
Q35	At the selected place(s) in Q24, the time you had with the {doctor/other health professional} was too short.<br><br>*-Record 99 if don't know/unsure*	0 = Strongly agree<br>1 = Agree<br>2 = Not sure<br>3 = Disagree<br>4 = Strongly disagree	Categorical ordinal	Patien t satisfa ction of care servic es	SAPS 2006
Q36	Are you satisfied with the care you received in the selected place(s) in Q24?<br><br>*-Record 99 if don't know/unsure*	0 = Very satisfied<br>1 = Satisfied<br>2 = Neither s atisfied nor dissatisfied<br>3 = Dissatisfied<br>4 = Very dissatisfied	Categorical ordinal	Patien t satisfa ction of care servic es	SAPS 2006
Q37	Did you get your blood pressure measured at the selected place(s) in Q24?<br><br>*-Record 99 if don't know/unsure*	0 = No<br>1 = Yes	Categorical variable	Acces s to blood pressu re testing	Opinion

Q38	Did you get your blood glucose tested at the selected place(s) in Q24?<br><br>*-Record 99 if don't know/unsure*	0 = No<br>1 = Yes	Categorical variable	Acces s to blood glucos e testing	Opinion
Q39	Would you recommend the selected place(s) in Q24 to others?<br><br>*-Record 99 if don't know/unsure*	1 = Not recommended<br>2 = Recommend with reservations<br>3 = Recommend<br>4 = Highly recommend	Categorical ordinal	Patien t satisfa ction of care servic es	Opinion

peer review only

For peer review only - http://bmjopen.bmj.com/site/about/guidelines.xhtml

SECTION 3b: HEALTH CARE UTILIZATION FOR DIABETES					
**Q.N**	**Description and Questions**	**Response**	**Type of Variable**	**Field**	**Ref:**
Q43b	How long have you lived with diabetes?<br><br>*-Record 99 if don't know/unsure and 88 if refuse*<br>*-Less than a year is rounded up to one year*<br>*-Standard rounded up formula is applied.*	________Years	Quantitative concrete	Durati on DM	Opinion
Q44b	Where were you first diagnosed as having diabetes?<br><br>*-Record 99 if don't know and 88 if refuse*	1= National hospital (PP)<br>2= Provincial hospital (RH)<br>3= District hospital (RH)<br>4= Health centre<br>5= Health post<br>6= Provincial rehabilitation centre (PRC) or Community-based rehabilitation (CBR)<br>7= Other public; specify:<br>8= Private hospital<br>9= Private clinic<br>10= Private pharmacy<br>11= Home/Office of trained health worker/nurse<br>12= Visit of trained health worker/nurse<br>13= Other private medical; specify:<br>14= Shop selling drugs/market<br>15= Kru Khmer/ Magician	Categorical Nominal	Diagn osis DM (CoC)	Opinion

For peer review only - http://bmjopen.bmj.com/site/about/guidelines.xhtml

		16= Monk/religious leader<br>17= Traditional birth attendant<br>18= Oversee medical service<br>19= MoPoTsyo<br>20= Other; specify:			
Q45b	Where did you first seek advice or treatment for diabetes after being diagnosed?<br><br>*-Record 99 if don't know and 88 if refuse*	1= National hospital (PP)<br>2= Provincial hospital (RH)<br>3= District hospital (RH)<br>4= Health centre<br>5= Health post<br>6= Provincial rehabilitation centre (PRC) or Community-based rehabilitation (CBR)<br>7= Other public; specify:<br>8= Private hospital<br>9= Private clinic<br>10= Private pharmacy<br>11= Home/Office of trained health worker/nurse<br>12= Visit of trained health worker/nurse<br>13= Other private medical; specify:<br>14= Shop selling drugs/market<br>15= Kru Khmer/ Magician<br>16= Monk/religious leader<br>17= Traditional birth attendant	Categorical Nominal	Link to care DM (CoC)	CDHS (Adapted for disease specific)

For peer review only - http://bmjopen.bmj.com/site/about/guidelines.xhtml

		18= Oversee medical service<br>19= MoPoTsyo<br>20= Other; specify:			
Q46b	Did you go to other places for follow up treatment/care for your diabetes conditions?<br><br>*-Record 88 if refuse*<br>*-If NO, please skip Q51b*	0 = No<br>1 = Yes	Categorical binary	Trust DM	CDHS
Q47b	If yes to Q46b, where else did you go to get follow up treatment/care for your diabetes conditions?	1= National hospital (PP)<br>2= Provincial hospital (RH)<br>3= District hospital (RH)<br>4= Health centre<br>5= Health post<br>6= Provincial rehabilitation centre (PRC) or Community-based rehabilitation (CBR)<br>7= Other public; specify:<br>8= Private hospital<br>9= Private clinic<br>10= Private pharmacy<br>11= Home/Office of trained health worker/nurse<br>12= Visit of trained health worker/nurse<br>13= Other private medical; specify:<br>14= Shop selling drugs/market	Categorical Nominal	Link to care DM (CoC)	CDHS (Adapted for disease specific)

For peer review only - http://bmjopen.bmj.com/site/about/guidelines.xhtml

		15= Kru Khmer/ Magician<br>16= Monk/religious leader<br>17= Traditional birth attendant<br>18= Oversee medical service<br>19= MoPoTsyo<br>20= Other; specify:			
Q48b	Did you get treatment/care for your diabetes conditions in the past 12 months?<br><br>*-Record 88 if refuse*<br>*-If NO, please skip Q49b-65b*	0 = No<br>1 = Yes	Categorical binary	Retain in care DM (CoC)	CDHS (Adapted for disease specific)
Q49b	Are you currently receiving any of the following treatment/advices for your diabetes conditions prescribed by a doctor or other health care worker?<br><br>Q. 49b.1. Insulin [____]<br>Q. 49b.2. Drugs (medication) that you have taken in the past two weeks [____]<br>Q. 49b.3. Special prescribed diet [____]<br>Q. 49b.4. Advice or treatment to lose weight [____]<br>Q. 49b.5. Advice or treatment to stop smoking<br>Q. 49b.6. Advice to start or do more physical exercise	0 = No 1= Yes	Categorical ordinal	In treatment for DM (CoC)	STEPS Survey
Q50b	Have you had your blood glucose measured in the past 12 months?<br><br>*-Record 99 if don't know and 88 if refuse*	0 = No 1= Yes	Categorical ordinal	In treatment for DM (CoC)	Veerle's suggestion

For peer review only - http://bmjopen.bmj.com/site/about/guidelines.xhtml

Q51b	Have you had your HbA1c tested in the past 12 months?<br><br>*-Record 99 if don't know and 88 if refuse*	0 = No 1= Yes	Categorical ordinal	In treatment for DM (CoC)	Veerle's suggestion
Q52b	Where did you seek medical advice or treatment for illness in the **past 3 months**?<br><br>***-More than one answer can be selected.***<br>*-Data collectors can use probes to help respondents determine the types of health facilities in the Response Column.*<br>*-Record 99 if don't know and 88 if refuse*<br>*-If 21=Nowhere, go to Q66b.*	1= National hospital (PP)<br>2= Provincial hospital (RH)<br>3= District hospital (RH)<br>4= Health centre<br>5= Health post<br>6= Provincial rehabilitation centre (PRC) or Community-based rehabilitation (CBR)<br>7= Other public; specify:<br>8= Private hospital<br>9= Private clinic<br>10= Private pharmacy<br>11= Home/Office of trained health worker/nurse<br>12= Visit of trained health worker/nurse<br>13= Other private medical; specify:<br>14= Shop selling drugs/market<br>15= Kru Khmer/ Magician<br>16= Monk/religious leader<br>17= Traditional birth attendant<br>18= Oversee medical service	Categorical Nominal	Care provider_3M	CDHS and GACD book

For peer review only - http://bmjopen.bmj.com/site/about/guidelines.xhtml

		19= MoPoTsyo<br>20= Other; specify:<br>21= No where			
From Q53b-Q65b, it is a set of questions that are asked following choices selected in Q52b. If 2 or 3 choices were selected in Q52b, Q53b-Q65b would appear 2 or 3 times, accordingly.					
Q53b	How many times did you visit the selected place**(s)** in Q52b in the past three months?	________ times	Quantitative discrete	Number of visits	GACD
Q54b	How much in total was spent on the treatment at the selected place(s) in Q52b?<br><br>*-Record 99 if don't know and 88 if refuse*	0 = free/no cost<br>1 = in kind<br>2 = ________Riels<br>OR<br>_________ USD	-Categorical nominal<br>-<br>Quantitative Continuous	Cost of treatment	CDHS
Q55b	How did you pay for the treatment cost at the selected place(s) in Q52b?<br><br>*-Record 99 if don't know and 88 if refuse*	1= Health Equity Fund<br>2= Voucher<br>3= Fee Exemption<br>4= NGO<br>5= National Social Security Fund<br>6= Community-Based Health Insurance<br>7= Health Insurance through Employer<br>8= Other Privately	-Categorical nominal	Payment method	GADC adapted to context specific

For peer review only - http://bmjopen.bmj.com/site/about/guidelines.xhtml

		Purchased Commercial Health Insurance<br>9= Wage/income<br>10= Loan/ Ton Tin<br>11= Sale of Assets<br>12= Gift from Relative<br>13= Savings<br>14= Other			
Q56b	How much in total was spent on transport to go to and return from the selected place(s) in Q52b?<br><br>*-Record 99 if don't know and 88 if refuse*	0 = free/no cost<br>1 = in kind<br>2 = ________Riels<br>OR<br>________ USD	-Categorical nominal<br>-<br>Quantitative Continuous	Cost of transp ort	CDHS
Q57b	On average how many hours do you spend to get treatment/advices from the selected place(s) in Q52b?<br><br>*-Record 99 if don't know and 88 if refuse*	________Hours	Quantitative continuous	Time spendi ng	GACD book (Adapted)
Q58b	How satisfied are you with the effect of your {treatment/care} at the selected place(s) in Q52b?<br><br>*-Record 99 if don't know/unsure*	0 = Very satisfied<br>1 = Satisfied<br>2 = Neither satisfied nor dissatisfied<br>3 = Dissatisfied<br>4 = Very dissatisfied	Categorical ordinal	Patien t satisfa ction of care servic e	SAPS 2006
Q59b	How satisfied are you with the explanations the {doctor/other health professional} has given you about the results of your {treatment/care} at the selected place(s) in Q52b?<br><br>*-Record 99 if don't know/unsure*	0 = Very satisfied<br>1 = Satisfied<br>2 = Neither satisfied nor dissatisfied<br>3 = Dissatisfied<br>4 = Very d issatisfied	Categorical ordinal	Patien t satisfa ction of care servic e	SAPS 2006

For peer review only

For peer review only - http://bmjopen.bmj.com/site/about/guidelines.xhtml

Q60b	The {doctor/other health professional} at the selected place(s) in Q52b was very careful to check everything when examining you.<br><br>*-Record 99 if don't know/unsure*	0 = Strongly agree<br>1 = Agree<br>2 = Not sure<br>3 = Disagree<br>4 = Strongly disagree	Categorical ordinal	Patient satisfaction of care service	SAPS 2006
Q61b	At the selected place(s) in Q52b, how satisfied were you with the choices you had in decisions affecting your health care?<br><br>*-Record 99 if don't know/unsure*	0 = Very satisfied<br>1 = Satisfied<br>2 = Neither satisfied nor dissatisfied<br>3 = Dissatisfied<br>4 = Very dissatisfied	Categorical ordinal	Patient satisfaction of care service	SAPS 2006
Q62b	How much of the time did you feel respected by the {doctor/other health professional} at the selected place(s) in Q52b?<br><br>*-Record 99 if don't know/unsure*	0 = All of the time<br>1 = Most of the time<br>2 = About half the time<br>3 = Some of the time<br>4 = None of the time	Categorical ordinal	Patient satisfaction of care service	SAPS 2006
Q63b	At the selected place(s) in Q52b, the time you had with the {doctor/other health professional} was too short.<br><br>*-Record 99 if don't know/unsure*	0 = Strongly agree<br>1 = Agree<br>2 = Not sure<br>3 = Disagree<br>4 = Strongly disagree	Categorical ordinal	Patient satisfaction of care service	SAPS 2006
Q64b	Are you satisfied with the care you received in the selected place(s) in Q52b?<br><br>*-Record 99 if don't know/unsure*	0 = Very satisfied<br>1 = Satisfied<br>2 = Neither satisfied nor dissatisfied<br>3 = Dissatisfied<br>4 = Very dissatisfied	Categorical ordinal	Patient satisfaction of care service	SAPS 2006

For peer review only - http://bmjopen.bmj.com/site/about/guidelines.xhtml

Q65b	Would you recommend the selected place(s) in Q52b to others?<br><br>*-Record 99 if don't know/unsure*	1 = Not recommended<br>2 = Recommend with reservations<br>3 = Recommend<br>4 = Highly recommend	Categorical ordinal	Patient satisfaction of care services	Opinion
Q66b	Have you ever been told by a doctor that you have eyes problems?<br><br>*-Record 99 if don't know/unsure*	0 = No<br>1 = Yes	Quantitative discrete	Complication-DM	Cambodia national guideline
Q67b	Have you ever been told by a doctor that you have kidney problems?<br><br>*-Record 99 if don't know/unsure*	0 = No<br>1 = Yes	Quantitative discrete	Complication-DM	Cambodia national guideline
Q68b	Have you ever been told by a doctor that you have lost the sensation of your peripheral membrane, such as foot, hand, arm?<br><br>*-Record 99 if don't know/unsure*	0 = No<br>1 = Yes	Quantitative discrete	Complication-DM	Cambodia national guideline
	In **the past 3 months**, besides seeking medical advice or treatment for your diabetes condition, have you sought medical treatment or advice for other illnesses or conditions?<br><br>If No, go to Section 4.	0 = No<br>1 = Yes			

For peer review only - http://bmjopen.bmj.com/site/about/guidelines.xhtml

Q24	Where did you seek medical advice or treatment for that illness in the past 3 months?<br><br>*-More than one answer can be selected.*<br>*-Data collectors can use probes to help respondents determine the types of health facilities in the Response Column.*<br>*-Record 99 if don't know and 88 if refuse*	1= National hospital (PP)<br>2= Provincial hospital (RH)<br>3= District hospital (RH)<br>4= Health centre<br>5= Health post<br>6= Provincial rehabilitation centre (PRC) or Community-based rehabilitation (CBR)<br>7= Other public; specify:<br>8= Private hospital<br>9= Private clinic<br>10= Private pharmacy<br>11= Home/Office of trained health worker/nurse<br>12= Visit of trained health worker/nurse<br>13= Other private medical; specify:<br>14= Shop selling drugs/market<br>15= Kru Khmer/ Magician<br>16= Monk/religious leader<br>17= Traditional birth attendant<br>18= Oversee medical service<br>19= Other; specify:	Categorical Nominal	Care provider_3M	CDHS and GACD book
From Q25-Q39, it is a set of questions that are asked following choices selected in Q24. If 2 or 3 choices were selected in Q24, Q25-Q39 would appear 2 or 3 times, accordingly.					

Q25	How many times did you visit the selected place**(s)** in Q24 in the past three months?	________ times	Quantitative discrete	Numb er of visits	GACD
Q26	How much in total was spent on the treatment at the selected place(s) in Q24?<br><br>*-Record 99 if don't know and 88 if refuse*	0 = free/no cost<br>1 = in kind<br>2 = _________Riels	-Categorical nominal<br>-Quantitative Continuous	Cost of treatm ent	CDHS
Q27	How did you pay for the treatment cost at the selected place(s) in Q24?<br><br>*-Record 99 if don't know and 88 if refuse*	1= Health Equity Fund<br>2= Voucher<br>3= Fee Exemption<br>4= NGO<br>5= National Social Security Fund<br>6= Community-Based Health Insurance<br>7= Health Insurance through Employer<br>8= Other Privately Purchased Commercial Health Insurance<br>9= Wage/income<br>10= Loan/ Ton Tin<br>11= Sale of Assets<br>12= Gift from Relative<br>13= Savings<br>14= Other	-Categorical nominal	Paym ent metho d	GADC adapted to context specific

For peer review only - http://bmjopen.bmj.com/site/about/guidelines.xhtml

Q28	How much in total was spent on transport to go to and return from the selected place(s) in Q24?<br><br>*-Record 99 if don't know and 88 if refuse*	0 = free/no cost<br>1 = in kind<br>2 = ________Riels	-Categorical nominal<br>-Quantitative Continuous	Cost of transport	CDHS
Q29	On average how many hours do you spend to get treatment/advices from the selected place(s) in Q24?<br><br>*-Record 99 if don't know and 88 if refuse*	________Hours	Quantitative continuous	Time spending	GACD book (Adapted)
Q30	How satisfied are you with the effect of your {treatment/care} at the selected place(s) in Q24?<br><br>*-Record 99 if don't know/unsure*	0 = Very satisfied<br>1 = Satisfied<br>2 = Neither satisfied nor dissatisfied<br>3 = Dissatisfied<br>4 = Very dissatisfied	Categorical ordinal	Patient satisfaction of care services	SAPS 2006
Q31	How satisfied are you with the explanations the {doctor/other health professional} has given you about the results of your {treatment/care} at the selected place(s) in Q24?<br><br>*-Record 99 if don't know/unsure*	0 = Very satisfied<br>1 = Satisfied<br>2 = Neither satisfied nor dissatisfied<br>3 = Dissatisfied<br>4 = Very dissatisfied	Categorical ordinal	Patient satisfaction of care services	SAPS 2006
Q32	The {doctor/other health professional} at the selected place(s) in Q24 was very careful to check everything when examining you.<br><br>*-Record 99 if don't know/unsure*	0 = Strongly agree<br>1 = Agree<br>2 = Not sure<br>3 = Disagree<br>4 = Strongly disagree	Categorical ordinal	Patient satisfaction of care services	SAPS 2006

For peer review only - http://bmjopen.bmj.com/site/about/guidelines.xhtml

Q33	At the selected place(s) in Q24, how satisfied were you with the choices you had in decisions affecting your health care?<br><br>*-Record 99 if don't know/unsure*	0 = Very satisfied<br>1 = Satisfied<br>2 = Neither s atisfied nor dissatisfied<br>3 = Dissatisfied<br>4 = Very dissatisfied	Categorical ordinal	Patien t satisfa ction of care servic es	SAPS 2006
Q34	How much of the time did you feel respected by the {doctor/other health professional} at the selected place(s) in Q24?<br><br>*-Record 99 if don't know/unsure*	0 = All of the time<br>1 = Most of the time<br>2 = About half the time<br>3 = Some of the time<br>4 = None of the time	Categorical ordinal	Patien t satisfa ction of care servic es	SAPS 2006
Q35	At the selected place(s) in Q24, the time you had with the {doctor/other health professional} was too short.<br><br>*-Record 99 if don't know/unsure*	0 = Strongly agree<br>1 = Agree<br>2 = Not sure<br>3 = Disagree<br>4 = Strongly disagree	Categorical ordinal	Patien t satisfa ction of care servic es	SAPS 2006
Q36	Are you satisfied with the care you received in the selected place(s) in Q24?<br><br>*-Record 99 if don't know/unsure*	0 = Very satisfied<br>1 = Satisfied<br>2 = Neither s atisfied nor dissatisfied<br>3 = Dissatisfied<br>4 = Very dissatisfied	Categorical ordinal	Patien t satisfa ction of care servic es	SAPS 2006
Q37	Did you get your blood pressure measured at the selected place(s) in Q24?<br><br>*-Record 99 if don't know/unsure*	0 = No<br>1 = Yes	Categorical variable	Acces s to blood pressu re testing	Opinion

For peer review only - http://bmjopen.bmj.com/site/about/guidelines.xhtml

Q38	Did you get your blood glucose tested at the selected place(s) in Q24?<br><br>*-Record 99 if don't know/unsure*	0 = No<br>1 = Yes	Categorical variable	Access to blood glucose testing	Opinion
Q39	Would you recommend the selected place(s) in Q24 to others?<br><br>*-Record 99 if don't know/unsure*	1 = Not recommended<br>2 = Recommend with reservations<br>3 = Recommend<br>4 = Highly recommend	Categorical ordinal	Patient satisfaction of care services	Opinion

peer review only

For peer review only - http://bmjopen.bmj.com/site/about/guidelines.xhtml

SECTION 3c: HEALTH CARE UTILIZATION FOR DIABETES AND HYPERTENSION					
**Q.N**	**Description and Questions**	**Response**	**Type of Variable**	**Field**	**Ref:**
Q43c	How long have you lived with diabetes?<br><br>*-Record 99 if don't know/unsure and 88 if refuse*<br>*-Less than a year is rounded up to one year*<br>*-Standard rounded up formula is applied.*	________Years	Quantitative concrete	Duration DM and HT	Opinion
Q44c	Where were you first diagnosed as having diabetes?<br><br>*-Record 99 if don't know and 88 if refuse*	1= National hospital (PP)<br>2= Provincial hospital (RH)<br>3= District hospital (RH)<br>4= Health centre<br>5= Health post<br>6= Provincial rehabilitation centre (PRC) or Community-based rehabilitation (CBR)<br>7= Other public; specify:<br>8= Private hospital<br>9= Private clinic<br>10= Private pharmacy<br>11= Home/Office of trained health worker/nurse<br>12= Visit of trained health worker/nurse<br>13= Other private medical; specify:<br>14= Shop selling drugs/market<br>15= Kru Khmer/ Magician	Categorical Nominal	Diagnosis DM (CoC)	Opinion

For peer review only - http://bmjopen.bmj.com/site/about/guidelines.xhtml

		16= Monk/religious leader<br>17= Traditional birth attendant<br>18= Oversee medical service<br>19= MoPoTsyo<br>20= Other; specify:			
Q45c	Where did you first seek advice or treatment for diabetes after being diagnosed?<br><br>*-Record 99 if don't know and 88 if refuse*	1= National hospital (PP)<br>2= Provincial hospital (RH)<br>3= District hospital (RH)<br>4= Health centre<br>5= Health post<br>6= Provincial rehabilitation centre (PRC) or Community-based rehabilitation (CBR)<br>7= Other public; specify:<br>8= Private hospital<br>9= Private clinic<br>10= Private pharmacy<br>11= Home/Office of trained health worker/nurse<br>12= Visit of trained health worker/nurse<br>13= Other private medical; specify:<br>14= Shop selling drugs/market<br>15= Kru Khmer/ Magician<br>16= Monk/religious leader<br>17= Traditional birth attendant	Categorical Nominal	Link to care DM (CoC)	CDHS (Adapted for disease specific)

For peer review only - http://bmjopen.bmj.com/site/about/guidelines.xhtml

		18= Oversee medical service<br>19= MoPoTsyo<br>20= Other; specify:			
Q46c	How long have you lived with hypertension?<br><br>*-Record 99 if don't know/unsure and 88 if refuse*<br>*-Less than a year is rounded up to one year*<br>*-Standard rounded up formula is applied.*	________Years	Quantitative concrete	Durati on DM and HT	Opinion
Q47c	Where were you first diagnosed as having hypertension?<br><br>*-Record 99 if don't know and 88 if refuse*	1= National hospital (PP)<br>2= Provincial hospital (RH)<br>3= District hospital (RH)<br>4= Health centre<br>5= Health post<br>6= Provincial rehabilitation centre (PRC) or Community-based rehabilitation (CBR)<br>7= Other public; specify:<br>8= Private hospital<br>9= Private clinic<br>10= Private pharmacy<br>11= Home/Office of trained health worker/nurse<br>12= Visit of trained health worker/nurse<br>13= Other private medical; specify:<br>14= Shop selling drugs/market	Categorical Nominal	Diagn osis HT (CoC)	Opinion

For peer review only - http://bmjopen.bmj.com/site/about/guidelines.xhtml

		15= Kru Khmer/ Magician<br>16= Monk/religious leader<br>17= Traditional birth attendant<br>18= Oversee medical service<br>19= MoPoTsyo<br>20= Other; specify:			
Q48c	Where did you first seek advice or treatment for hypertension after being diagnosed?<br><br>*-Record 99 if don't know and 88 if refuse*	1= National hospital (PP)<br>2= Provincial hospital (RH)<br>3= District hospital (RH)<br>4= Health centre<br>5= Health post<br>6= Provincial rehabilitation centre (PRC) or Community-based rehabilitation (CBR)<br>7= Other public; specify:<br>8= Private hospital<br>9= Private clinic<br>10= Private pharmacy<br>11= Home/Office of trained health worker/nurse<br>12= Visit of trained health worker/nurse<br>13= Other private medical; specify:<br>14= Shop selling drugs/market<br>15= Kru Khmer/ Magician<br>16= Monk/religious leader	Categorical Nominal	Link to care HT (CoC)	CDHS (Adapted for disease specific)

For peer review only - http://bmjopen.bmj.com/site/about/guidelines.xhtml

		17= Traditional birth attendant<br>18= Oversee medical service<br>19= MoPoTsyo<br>20= Other; specify:			
Q49c	Did you go to other places for follow up treatment/care for your hypertensive and diabetes conditions?<br><br>*-Record 88 if refuse*<br>*-If NO, please skip Q50c*	0 = No<br>1 = Yes	Categorical binary	Trust DM and HT	CDHS
Q50c	If yes to Q49c, where else did you go to get follow up treatment/care for both conditions?	1= National hospital (PP)<br>2= Provincial hospital (RH)<br>3= District hospital (RH)<br>4= Health centre<br>5= Health post<br>6= Provincial rehabilitation centre (PRC) or Community-based rehabilitation (CBR)<br>7= Other public; specify:<br>8= Private hospital<br>9= Private clinic<br>10= Private pharmacy<br>11= Home/Office of trained health worker/nurse<br>12= Visit of trained health worker/nurse<br>13= Other private medical; specify:<br>14= Shop selling drugs/market	Categorical Nominal	Link to care DM and HT (CoC)	CDHS (Adapted for disease specific)

For peer review only - http://bmjopen.bmj.com/site/about/guidelines.xhtml

		15= Kru Khmer/ Magician<br>16= Monk/religious leader<br>17= Traditional birth attendant<br>18= Oversee medical service<br>19= MoPoTsyo<br>20= Other; specify:			
Q51c	Did you get treatment/care for both conditions in the past 12 months?<br><br>*-Record 88 if refuse*<br>*-If NO, please skip Q52c-69c*	0 = No<br>1 = Yes	Categorical binary	Retain in care DM and HT (CoC)	CDHS (Adapted for disease specific)
Q52c	Are you currently receiving any of the following treatment/advices for both conditions prescribed by a doctor or other health care worker?<br><br>Q. 52c.1. Insulin [_____]<br>Q. 52c.2. Drugs (medication) that you have taken in the past two weeks [_____]<br>Q. 52c.3. Special prescribed diet [_____]<br>Q. 52c.4. Advice or treatment to lose weight [_____]<br>Q. 52c.5. Advice or treatment to stop smoking<br>Q. 52c.6. Advice to reduce salt intake<br>Q. 52c.7. Advice to start or do more physical exercise	0 = No 1= Yes	Categorical ordinal	In treatment for DM and HT (CoC)	STEPS Survey
Q53c	Have you had your blood glucose measured in the past 12 months?<br><br>*-Record 99 if don't know and 88 if refuse*	0 = No 1= Yes	Categorical ordinal	In treatment for DM (CoC)	Veerle's suggestion

For peer review only - http://bmjopen.bmj.com/site/about/guidelines.xhtml

Q54c	Have you had your HbA1c tested in the past 12 months?<br><br>*-Record 99 if don't know and 88 if refuse*	0 = No 1= Yes	Categorical ordinal	In treatment for DM (CoC)	Veerle's suggestion
Q55c	Have you had your blood cholesterol measured in the past 12 months?<br><br>*-Record 99 if don't know and 88 if refuse*	0 = No 1= Yes	Categorical ordinal	In treatment for HT (CoC)	Veerle's suggestion
Q56c	Where did you seek medical advice or treatment for your conditions in **the past 3 months?**<br><br>***-More than one answer can be selected*.**<br>*-Data collectors can use probes to help respondents determine the types of health facilities in the Response Column.*<br>*-Record 99 if don't know and 88 if refuse*<br>*-If 21=Nowhere, go to Q70c.*	1= National hospital (PP)<br>2= Provincial hospital (RH)<br>3= District hospital (RH)<br>4= Health centre<br>5= Health post<br>6= Provincial rehabilitation centre (PRC) or Community-based rehabilitation (CBR)<br>7= Other public; specify:<br>8= Private hospital<br>9= Private clinic<br>10= Private pharmacy<br>11= Home/Office of trained health worker/nurse<br>12= Visit of trained health worker/nurse<br>13= Other private medical; specify:<br>14= Shop selling drugs/market	Categorical Nominal	Care provider_3M	CDHS and GACD book

For peer review only

For peer review only - http://bmjopen.bmj.com/site/about/guidelines.xhtml

		15= Kru Khmer/ Magician<br>16= Monk/religious leader<br>17= Traditional birth attendant<br>18= Oversee medical service<br>19= MoPoTsyo<br>20= Other; specify:<br>21= Nowhere			
From Q57c-Q69c, it is a set of questions that are asked following choices selected in Q56c. If 2 or 3 choices were selected in Q56c, Q57c-Q69c would appear 2 or 3 times, accordingly.					
Q57c	How many times did you visit the selected place**(s)** in Q56c in the past three months?	________ times	Quantitative discrete	Number of visits	GACD
Q58c	How much in total was spent on the treatment at the selected place(s) in Q56c?<br><br>*-Record 99 if don't know and 88 if refuse*	0 = free/no cost<br>1 = in kind<br>2 = _________Riels OR _________ USD	-Categorical nominal<br>-Quantitative Continuous	Cost of treatment	CDHS
Q59c	How did you pay for the treatment cost at the selected place(s) in Q56c?<br><br>*-Record 99 if don't know and 88 if refuse*	1= Health Equity Fund<br>2= Voucher<br>3= Fee Exemption<br>4= NGO<br>5= National Social Security Fund<br>6= Community-Based Health Insurance<br>7= Health Insurance through Employer<br>8= Other Privately	-Categorical nominal	Payment method	GADC adapted to context specific

For peer review only - http://bmjopen.bmj.com/site/about/guidelines.xhtml

		Purchased Commercial Health Insurance<br>9= Wage/income<br>10= Loan/ Ton Tin<br>11= Sale of Assets<br>12= Gift from Relative<br>13= Savings<br>14= Other			
Q60c	How much in total was spent on transport to go to and return from the selected place(s) in Q56c?<br><br>*-Record 99 if don't know and 88 if refuse*	0 = free/no cost<br>1 = in kind<br>2 = ________Riels<br>OR<br>________ USD	-Categorical nominal<br>-<br>Quantitative Continuous	Cost of transport	CDHS
Q61c	On average how many hours do you spend to get treatment/advices from the selected place(s) in Q56c?<br><br>*-Record 99 if don't know and 88 if refuse*	________Hours	Quantitative continuous	Time spending	GACD book (Adapted)
Q62c	How satisfied are you with the effect of your {treatment/care} at the selected place(s) in Q56c?<br><br>*-Record 99 if don't know/unsure*	0 = Very satisfied<br>1 = Satisfied<br>2 = Neither satisfied nor dissatisfied<br>3 = Dissatisfied<br>4 = Very dissatisfied	Categorical ordinal	Patient satisfaction of care service	SAPS 2006
Q63c	How satisfied are you with the explanations the {doctor/other health professional} has given you about the results of your {treatment/care} at the selected place(s) in Q56c?<br><br>*-Record 99 if don't know/unsure*	0 = Very satisfied<br>1 = Satisfied<br>2 = Neither satisfied nor dissatisfied<br>3 = Dissatisfied<br>4 = Very d issatisfied	Categorical ordinal	Patient satisfaction of care service	SAPS 2006

For peer review only - http://bmjopen.bmj.com/site/about/guidelines.xhtml

Q64c	The {doctor/other health professional} at the selected place(s) in Q56c was very careful to check everything when examining you.<br><br>*-Record 99 if don't know/unsure*	0 = Strongly agree<br>1 = Agree<br>2 = Not sure<br>3 = Disagree<br>4 = Strongly disagree	Categorical ordinal	Patient satisfaction of care service	SAPS 2006
Q65c	At the selected place(s) in Q56c, how satisfied were you with the choices you had in decisions affecting your health care?<br><br>*-Record 99 if don't know/unsure*	0 = Very satisfied<br>1 = Satisfied<br>2 = Neither satisfied nor dissatisfied<br>3 = Dissatisfied<br>4 = Very dissatisfied	Categorical ordinal	Patient satisfaction of care service	SAPS 2006
Q66c	How much of the time did you feel respected by the {doctor/other health professional} at the selected place(s) in Q56c?<br><br>*-Record 99 if don't know/unsure*	0 = All of the time<br>1 = Most of the time<br>2 = About half the time<br>3 = Some of the time<br>4 = None of the time	Categorical ordinal	Patient satisfaction of care service	SAPS 2006
Q67c	At the selected place(s) in Q56c, the time you had with the {doctor/other health professional} was too short.<br><br>*-Record 99 if don't know/unsure*	0 = Strongly agree<br>1 = Agree<br>2 = Not sure<br>3 = Disagree<br>4 = Strongly disagree	Categorical ordinal	Patient satisfaction of care service	SAPS 2006
Q68c	Are you satisfied with the care you received in the selected place(s) in Q56c?<br><br>*-Record 99 if don't know/unsure*	0 = Very satisfied<br>1 = Satisfied<br>2 = Neither satisfied nor dissatisfied<br>3 = Dissatisfied<br>4 = Very dissatisfied	Categorical ordinal	Patient satisfaction of care service	SAPS 2006

For peer review only - http://bmjopen.bmj.com/site/about/guidelines.xhtml

Q69c	Would you recommend the selected place(s) in Q56c to others?<br><br>*-Record 99 if don't know/unsure*	1 = Not recommended<br>2 = Recommend with reservations<br>3 = Recommend<br>4 = Highly recommend	Categorical ordinal	Patient satisfaction of care services	Opinion
Q70c	Have you ever been told by a doctor that you have eyes problems?<br><br>*-Record 99 if don't know/unsure*	0 = No<br>1 = Yes	Quantitative discrete	Complication-DM and HT	Cambodia national guideline
Q71c	Have you ever been told by a doctor that you have kidney problems?<br><br>*-Record 99 if don't know/unsure*	0 = No<br>1 = Yes	Quantitative discrete	Complication-DM and HT	Cambodia national guideline
Q72c	Have you ever been told by a doctor that you have lost the sensation of your peripheral membrane, such as foot, hand, arm?<br><br>*-Record 99 if don't know/unsure*	0 = No<br>1 = Yes	Quantitative discrete	Complication-DM and HT	Cambodia national guideline
	In **the past 3 months**, besides seeking medical advice or treatment for your hypertensive and diabetes condition, have you sought medical treatment or advice for other illnesses or conditions?<br><br>If No, go to Section 4.	0 = No<br>1 = Yes			

For peer review only - http://bmjopen.bmj.com/site/about/guidelines.xhtml

Q24	Where did you seek medical advice or treatment for that illness in the past 3 months?<br><br>*-More than one answer can be selected.*<br>*-Data collectors can use probes to help respondents determine the types of health facilities in the Response Column.*<br>*-Record 99 if don't know and 88 if refuse*	1= National hospital (PP)<br>2= Provincial hospital (RH)<br>3= District hospital (RH)<br>4= Health centre<br>5= Health post<br>6= Provincial rehabilitation centre (PRC) or Community-based rehabilitation (CBR)<br>7= Other public; specify:<br>8= Private hospital<br>9= Private clinic<br>10= Private pharmacy<br>11= Home/Office of trained health worker/nurse<br>12= Visit of trained health worker/nurse<br>13= Other private medical; specify:<br>14= Shop selling drugs/market<br>15= Kru Khmer/ Magician<br>16= Monk/religious leader<br>17= Traditional birth attendant<br>18= Oversee medical service<br>19= Other; specify:	Categorical Nominal	Care provider_3M	CDHS and GACD book
From Q25-Q39, it is a set of questions that are asked following choices selected in Q24. If 2 or 3 choices were selected in Q24, Q25-Q39 would appear 2 or 3 times, accordingly.					

For peer review only - http://bmjopen.bmj.com/site/about/guidelines.xhtml

Q25	How many times did you visit the selected place**(s)** in Q24 in the past three months?	_______ times	Quantitative discrete	Numb er of visits	GACD
Q26	How much in total was spent on the treatment at the selected place(s) in Q24?<br><br>-*Record 99 if don't know and 88 if refuse*	0 = free/no cost<br>1 = in kind<br>2 = ________Riels	-Categorical nominal<br>-Quantitative Continuous	Cost of treatm ent	CDHS
Q27	How did you pay for the treatment cost at the selected place(s) in Q24?<br><br>-*Record 99 if don't know and 88 if refuse*	1= Health Equity Fund<br>2= Voucher<br>3= Fee Exemption<br>4= NGO<br>5= National Social Security Fund<br>6= Community-Based Health Insurance<br>7= Health Insurance through Employer<br>8= Other Privately Purchased Commercial Health Insurance<br>9= Wage/income<br>10= Loan/ Ton Tin<br>11= Sale of Assets<br>12= Gift from Relative<br>13= Savings<br>14= Other	-Categorical nominal	Paym ent metho d	GADC adapted to context specific

For peer review only - http://bmjopen.bmj.com/site/about/guidelines.xhtml

Q28	How much in total was spent on transport to go to and return from the selected place(s) in Q24?<br><br>*-Record 99 if don't know and 88 if refuse*	0 = free/no cost<br>1 = in kind<br>2 = ________Riels	-Categorical nominal<br>-Quantitative Continuous	Cost of transport	CDHS
Q29	On average how many hours do you spend to get treatment/advices from the selected place(s) in Q24?<br><br>*-Record 99 if don't know and 88 if refuse*	________Hours	Quantitative continuous	Time spending	GACD book (Adapted)
Q30	How satisfied are you with the effect of your {treatment/care} at the selected place(s) in Q24?<br><br>*-Record 99 if don't know/unsure*	0 = Very satisfied<br>1 = Satisfied<br>2 = Neither satisfied nor dissatisfied<br>3 = Dissatisfied<br>4 = Very dissatisfied	Categorical ordinal	Patient satisfaction of care services	SAPS 2006
Q31	How satisfied are you with the explanations the {doctor/other health professional} has given you about the results of your {treatment/care} at the selected place(s) in Q24?<br><br>*-Record 99 if don't know/unsure*	0 = Very satisfied<br>1 = Satisfied<br>2 = Neither satisfied nor dissatisfied<br>3 = Dissatisfied<br>4 = Very dissatisfied	Categorical ordinal	Patient satisfaction of care services	SAPS 2006
Q32	The {doctor/other health professional} at the selected place(s) in Q24 was very careful to check everything when examining you.<br><br>*-Record 99 if don't know/unsure*	0 = Strongly agree<br>1 = Agree<br>2 = Not sure<br>3 = Disagree<br>4 = Strongly disagree	Categorical ordinal	Patient satisfaction of care services	SAPS 2006

For peer review only - http://bmjopen.bmj.com/site/about/guidelines.xhtml

Q33	At the selected place(s) in Q24, how satisfied were you with the choices you had in decisions affecting your health care?<br><br>*-Record 99 if don't know/unsure*	0 = Very satisfied<br>1 = Satisfied<br>2 = Neither s atisfied nor dissatisfied<br>3 = Dissatisfied<br>4 = Very dissatisfied	Categorical ordinal	Patien t satisfa ction of care servic es	SAPS 2006
Q34	How much of the time did you feel respected by the {doctor/other health professional} at the selected place(s) in Q24?<br><br>*-Record 99 if don't know/unsure*	0 = All of the time<br>1 = Most of the time<br>2 = About half the time<br>3 = Some of the time<br>4 = None of the time	Categorical ordinal	Patien t satisfa ction of care servic es	SAPS 2006
Q35	At the selected place(s) in Q24, the time you had with the {doctor/other health professional} was too short.<br><br>*-Record 99 if don't know/unsure*	0 = Strongly agree<br>1 = Agree<br>2 = Not sure<br>3 = Disagree<br>4 = Strongly disagree	Categorical ordinal	Patien t satisfa ction of care servic es	SAPS 2006
Q36	Are you satisfied with the care you received in the selected place(s) in Q24?<br><br>*-Record 99 if don't know/unsure*	0 = Very satisfied<br>1 = Satisfied<br>2 = Neither s atisfied nor dissatisfied<br>3 = Dissatisfied<br>4 = Very dissatisfied	Categorical ordinal	Patien t satisfa ction of care servic es	SAPS 2006
Q37	Did you get your blood pressure measured at the selected place(s) in Q24?<br><br>*-Record 99 if don't know/unsure*	0 = No<br>1 = Yes	Categorical variable	Acces s to blood pressu re testing	Opinion

For peer review only - http://bmjopen.bmj.com/site/about/guidelines.xhtml

Q38	Did you get your blood glucose tested at the selected place(s) in Q24?<br><br>*-Record 99 if don't know/unsure*	0 = No<br>1 = Yes	Categorical variable	Access to blood glucose testing	Opinion
Q39	Would you recommend the selected place(s) in Q24 to others?<br><br>*-Record 99 if don't know/unsure*	1 = Not recommended<br>2 = Recommend with reservations<br>3 = Recommend<br>4 = Highly recommend	Categorical ordinal	Patient satisfaction of care services	Opinion

peer review only

For peer review only - http://bmjopen.bmj.com/site/about/guidelines.xhtml

For peer review only

For peer review only - http://bmjopen.bmj.com/site/about/guidelines.xhtml

Annexure 4. Arithmetic and the geometric mean of medical cost by participant characteristics in Cambodia, 2020

Table S1. Arithmetic mean of medical cost by participant characteristics in Cambodia, 2020

Variable	**Overall**	**No T2D/HTN**	**HTN**	**T2D**	**T2D plus HTN**
	Mean [95% CI]	Mean [95% CI]	Mean [95% CI]	Mean [95% CI]	Mean [95% CI]
Sex					
Male	16.8 [15.2–18.5]	16.8 [14.7–18.9]	15.5 [12.3–18.6]	25.5 [15.8–35.2]	17.0 [10.2–23.7]
Female	19.5 [18.3–20.6]	22.6 [20.8–24.4]	14.3 [12.7–15.8]	21.1 [16.0–26.2]	22.2 [18.9–25.5]
P-value	0.015	<0.001	0.480	0.393	0.212
Age in years					
40–49	19.0 [16.8–21.2]	20.6 [18.1–23.2]	20.6 [18.1–23.2]	10.3 [6.5–14.0]	19.7 [7.1–32.4]
50–59	19.1 [17.5–20.7]	82.8 [73.0–92.6]	20.7 [18.3–23.2]	14.2 [12.0–16.5]	24.5 [17.6–31.3]
60+	18.2 [16.8–19.7]	82.1 [72.8–91.4]	20.5 [18.2–22.9]	15.4 [13.4–17.4]	21.1 [13.8–28.4]
P-value	0.698	0.995	0.120	0.704	0.850
Educational level					
No schooling	19.1 [17.4–20.9]	20.6 [17.9–23.3]	16.2 [13.8–18.7]	22.8 [14.8–30.7]	22.1 [15.8–28.5]
Primary	17.9 [16.7–19.1]	20.3 [18.5–22.1]	12.9 [11.1–14.7]	20.5 [15.1–26.0]	19.9 [16.7–23.2]
Secondary/higher	21.1 [18.0–24.3]	22.0 [17.9–26.2]	16.9 [11.5–22.2]	28.6 [8.8–48.4]	26.8 [14.3–39.2]
P-value	0.091	0.726	0.054	0.506	0.365
Having NSSF membership					
No	18.8 [17.8–19.7]	20.7 [19.2–22.1]	14.5 [13.1–16.0]	22.9 [18.1–27.6]	21.9 [18.8–25.0]
Yes	17.5 [13.2–21.8]	20.4 [13.9–26.9]	14.7 [7.0–22.4]	13.8 [1.6–26.0]	11.8 [4.7–18.8]
P-value	0.5604	0.937	0.961	0.339	0.076
Having HEF membership					
No	19.4 [18.3–20.5]	21.4 [19.8–23.0]	14.9 [13.3–16.5]	24.1 [18.8–29.3]	22.6 [19.4–25.9]
Yes	15.8 [13.8–17.8]	17.6 [14.6–20.5]	13.2 [10.2–16.2]	14.3 [11.0–18.6]	14.9 [7.5–22.3]
P-value	0.004	0.035	0.360	0.096	0.058
Household socio-economic class					
Poorest	20.2 [17.7–22.8]	23.9 [20.1–27.6]	13.6 [10.3–16.9]	18.0 [6.6–29.4]	23.6 [14.8–32.4]
Poor	16.9 [14.8–18.9]	17.8 [15.0–20.6]	14.7 [11.4–18.0]	23.2 [11.3–35.1]	14.8 [6.0–23.7]
Medium	18.2 [16.2–20.2]	19.8 [16.9–22.6]	14.7 [11.4–18.0]	18.8 [12.5–25.0]	21.2 [14.4–28.1]
Rich	20.0 [17.8–22.2]	23.0 [19.4–26.5]	15.6 [12.5–18.6]	23.4 [10.3–36.6]	21.5 [16.8–26.3]
Richest	18.2 [16.2–20.2]	19.2 [16.4–22.0]	14.0 [11.0–17.0]	26.8 [15.8–37.7]	24.1 [17.4–30.8]
P-value	0.171	0.034	0.919	0.687	0.427
Sector					
Private	20.5 [19.3–21.6]	23.0 [21.4–24.7]	15.7 [14.0–17.3]	24.9 [18.3–31.6]	22.0 [18.0–26.0]
Public	12.0 [10.3–13.7]	9.8 [7.4–12.2]	10.6 [7.7–13.6]	18.5 [13.2–23.8]	20.4 [15.6–25.2]
Both	21.7 [17.4–26.1]	24.8 [19.5–30.1]	9.4 [5.7–13.1]	—	20.5 [10.6–30.4]

P-value	<0.001	<0.001	0.013	0.164	0.878
Care model					
Coexisting	16.7 [14.7–18.7]	17.9 [15.1–20.6]	13.8 [10.8–16.9]	20.0 [11.8–28.2]	23.3 [12.1–34.6]
Community-based	22.6 [20.3–25.0]	24.5 [21.1–27.9]	18.9 [15.1–22.7]	22.3 [12.7–32.0]	27.2 [19.2–35.2]
Health center-based (high)	18.6 [16.4–20.7]	20.7 [17.5–24.0]	14.8 [11.7–17.8]	19.7 [9.2–30.1]	22.8 [14.6–30.9]
Health center-based (low)	15.7 [13.9–17.5]	17.6 [14.8–20.4]	11.0 [8.8–13.2]	24.1 [13.1–35.2]	19.0 [15.1–22.9]
Hospital-based	20.3 [17.9–22.7]	22.8 [19.4–26.2]	14.8 [10.9–18.7]	23.3 [12.1–34.6]	18.1 [12.0–24.1]
P-value	<0.001	0.006	0.011	0.896	0.2695

Abbreviation CI, confidence interval

Note: Health center-based (high) means the OD with high coverage (six out of nine) of health centers with the WHO PEN; Health center-based (low) means the OD with low coverage (six out of 25) of health centers with the WHO PEN

Table S2. Geometric mean of medical cost by participant characteristics in Cambodia, 2020

Variable	**Overall**	**No T2D/HTN**	**HTN**	**T2D**	**T2D plus HTN**
	Mean [95% CI]	Mean [95% CI]	Mean [95% CI]	Mean [95% CI]	Mean [95% CI]
Sex					
Male	6.9 [6.2–7.8]	6.5 [5.6–7.5]	7.0 [5.7–8.6]	14.7 [8.8–24.7]	8.5 [4.2–17.0]
Female	8.5 [7.9–9.1]	8.9 [8.0–10.0]	6.8 [6.1–7.6]	12.5 [9.4–16.5]	13.9 [11.1–17.5]
P-value	**0.004**	**<0.001**	0.79	0.548	**0.104**
Age in years					
40–49	7.9 [6.8–9.0]	8.2 [7.0–9.7]	8.2 [7.0–9.7]	5.0 [3.7–6.8]	13.2 [8.3–20.9]
50–59	8.4 [7.6–9.4]	28.2 [23.6–33.6]	7.9 [6.7–9.2]	7.6 [6.4–8.9]	15.2 [10.6–21.9]
60+	7.7 [7.0–8.5]	28.4 [23.7–33.9]	7.9 [6.8–9.3]	6.9 [6.0–7.8]	11.1 [7.1–17.3]
P-value	0.480	0.91	**0.09**	0.509	0.384
Educational level					
No schooling	7.9 [7.0–8.8]	7.4 [6.2–8.8]	7.6 [6.4–8.9]	13.0 [8.1–20.8]	11.6 [7.4–18.2]
Primary	8.0 [7.4–8.7]	8.3 [7.4–9.4]	6.4 [5.7–7.3]	12.6 [9.2–17.3]	12.9 [9.8–17.0]
Secondary/higher	8.2 [6.8–9.9]	8.0 [6.2–10.4]	6.6 [4.7–9.4]	15.7 [7.0–35.1]	17.2 [9.2–32.2]
P-value	0.920	0.510	0.299	0.842	0.600
Having NSSF membership					
No	8.1 [7.6–8.6]	8.0 [7.3–8.8]	7.0 [6.3–7.7]	13.6 [10.6–17.4]	13.5 [10.8–16.9]
Yes	6.8 [5.0–9.2]	7.9 [5.2–12.0]	5.3 [3.0–9.2]	7.6 [1.5–37.8]	6.3 [2.2–18.1]
P-value	**0.2351**	0.937	0.234	0.261	**0.071**
Having HEF membership					
No	8.8 [8.2–9.4]	8.7 [7.9–9.7]	7.4 [6.6–8.2]	14.1 [7.4–20.9]	16.1 [13.5–19.3]
Yes	5.4 [4.6–6.3]	5.5 [4.4–6.9]	5.1 [4.0–6.7]	8.6 [4.5–16.3]	4.1 [1.7–9.9]
P-value	**<0.001**	**<0.001**	**0.004**	**0.114**	**<0.001**
Household socio-economic class					

For peer review only

Poorest	6.8 [5.8–8.0]	7.3 [5.8–9.2]	5.4 [4.1–6.9]	8.4 [3.9–18.3]	11.1 [5.5–22.4]
Poor	7.4 [6.5–8.5]	7.4 [6.1–8.9]	7.1 [5.7–8.8]	13.6 [7.2–25.9]	6.6 [3.2–13.7]
Medium	8.4 [7.3–9.6]	8.2 [6.7–10.0]	7.4 [6.0–9.1]	12.6 [7.9–20.1]	12.5 [7.5–20.8]
Rich	9.4 [8.2–10.8]	9.6 [7.8–11.9]	7.9 [6.5–9.6]	14.1 [6.8–28.9]	15.8 [11.1–22.5]
Richest	8.2 [7.2–9.3]	7.8 [6.5–9.4]	6.7 [5.5–8.3]	16.5 [10.5–25.7]	19.0 [14.4–25.1]
P-value	**0.022**	0.366	**0.133**	0.538	**0.054**
Sector					
Private	9.8 [9.2–10.5]	10.2 [9.2–11.2]	8.4 [7.6–9.2]	15.0 [11.2–20.2]	15.5 [12.6–19.0]
Public	3.5 [3.0–4.1]	2.5 [2.0–3.1]	3.1 [2.4–4.1]	10.7 [7.0–16.3]	9.5 [5.9–15.2]
Both	13.9 [10.8–17.9]	16.2 [12.2–21.5]	6.9 [4.0–12.1]	—	20.3 [12.7–32.4]
P-value	**<0.001**	**<0.001**	**<0.001**	**0.176**	**0.084**
Care model					
Coexisting	7.3 [6.3–8.5]	7.5 [6.2–9.2]	6.0 [4.7–7.6]	12.8 [6.9–23.5]	17.5 [10.8–28.3]
Community-based	10.8 [9.4–12.3]	10.4 [8.5–12.8]	9.7 [7.9–11.8]	16.5 [10.8–25.2]	20.5 [14.3–29.2]
Health center-based (high)	8.5 [7.5–9.7]	8.6 [7.0–10.5]	7.4 [6.1–9.0]	11.4 [7.1–18.2]	15.0 [9.5–23.9]
Health center-based (low)	6.4 [5.6–7.3]	5.9 [4.8–7.2]	5.6 [4.6–6.9]	13.7 [6.9–27.4]	13.9 [9.5–20.1]
Hospital-based	7.9 [6.7–9.2]	8.5 [6.9–10.5]	6.3 [4.8–8.1]	17.5 [10.8–28.3]	7.3 [4.2–12.8]
P-value	**<0.001**	**0.002**	**0.003**	0.911	**0.015**

Abbreviation CI, confidence interval

Note: Health center-based (high) means the OD with high coverage (six out of nine) of health centers with the WHO PEN; Health center-based (low) means the OD with low coverage (six out of 25) of health centers with the WHO PEN

For peer review only

STROBE Statement—Checklist of items that should be included in reports of ***cross-sectional studies***

	Item No	Recommendation	Page No
**Title and abstract**	1	(*a*) Indicate the study's design with a commonly used term in the title or the abstract	1
		(*b*) Provide in the abstract an informative and balanced summary of what was done and what was found	2-3
**Introduction**			
Background/rationale	2	Explain the scientific background and rationale for the investigation being reported	5
Objectives	3	State specific objectives, including any prespecified hypotheses	7
**Methods**			
Study design	4	Present key elements of study design early in the paper	10
Setting	5	Describe the setting, locations, and relevant dates, including periods of recruitment, exposure, follow-up, and data collection	10-14
Participants	6	(*a*) Give the eligibility criteria, and the sources and methods of selection of participants	12
Variables	7	Clearly define all outcomes, exposures, predictors, potential confounders, and effect modifiers. Give diagnostic criteria, if applicable	13-14
Data sources/ measurement	8*	For each variable of interest, give sources of data and details of methods of assessment (measurement). Describe comparability of assessment methods if there is more than one group	10, 13, 14
Bias	9	Describe any efforts to address potential sources of bias	12
Study size	10	Explain how the study size was arrived at	12-13
Quantitative variables	11	Explain how quantitative variables were handled in the analyses. If applicable, describe which groupings were chosen and why	15-16
Statistical methods	12	(*a*) Describe all statistical methods, including those used to control for confounding	15-16
		(*b*) Describe any methods used to examine subgroups and interactions	15-16
		(*c*) Explain how missing data were addressed	NA
		(*d*) If applicable, describe analytical methods taking account of sampling strategy	NA
		(*e*) Describe any sensitivity analyses	NA
**Results**			
Participants	13*	(a) Report numbers of individuals at each stage of study—eg numbers potentially eligible, examined for eligibility, confirmed eligible, included in the study, completing follow-up, and analysed	17
		(b) Give reasons for non-participation at each stage	17, 12, 13
		(c) Consider use of a flow diagram	13
Descriptive data	14*	(a) Give characteristics of study participants (eg demographic, clinical, social) and information on exposures and potential confounders	17
		(b) Indicate number of participants with missing data for each variable of interest	17
Outcome data	15*	Report numbers of outcome events or summary measures	18-19, 22

For peer review only

Main results	16	(*a*) Give unadjusted estimates and, if applicable, confounder-adjusted estimates and their precision (eg, 95% confidence interval). Make clear which confounders were adjusted for and why they were included	19-20, 23-24
		(*b*) Report category boundaries when continuous variables were categorized	NA
		(*c*) If relevant, consider translating estimates of relative risk into absolute risk for a meaningful time period	NA
Other analyses	17	Report other analyses done—eg analyses of subgroups and interactions, and sensitivity analyses	NA
**Discussion**			
Key results	18	Summarise key results with reference to study objectives	25
Limitations	19	Discuss limitations of the study, taking into account sources of potential bias or imprecision. Discuss both direction and magnitude of any potential bias	27-28
Interpretation	20	Give a cautious overall interpretation of results considering objectives, limitations, multiplicity of analyses, results from similar studies, and other relevant evidence	25-28
Generalisability	21	Discuss the generalisability (external validity) of the study results	27
**Other information**			
Funding	22	Give the source of funding and the role of the funders for the present study and, if applicable, for the original study on which the present article is based	29

*Give information separately for exposed and unexposed groups.

Note: An Explanation and Elaboration article discusses each checklist item and gives methodological background and published examples of transparent reporting. The STROBE checklist is best used in conjunction with this article (freely available on the Web sites of PLoS Medicine at http://www.plosmedicine.org/, Annals of Internal Medicine at http://www.annals.org/, and Epidemiology at http://www.epidem.com/). Information on the STROBE Initiative is available at www.strobe-statement.org.

For peer review only
